# Direct Minisci-Type C–H Amidation of Purine Bases

**DOI:** 10.1021/acs.orglett.2c03206

**Published:** 2022-10-26

**Authors:** David T. Mooney, Peter R. Moore, Ai-Lan Lee

**Affiliations:** † Institute of Chemical Sciences, Heriot-Watt University, Edinburgh, EH14 4AS Scotland, U.K.; ‡ Early Chemical Development, Pharmaceutical Sciences, R&D BioPharmaceuticals, AstraZeneca, Macclesfield, SK10 2NA England, U.K.

## Abstract

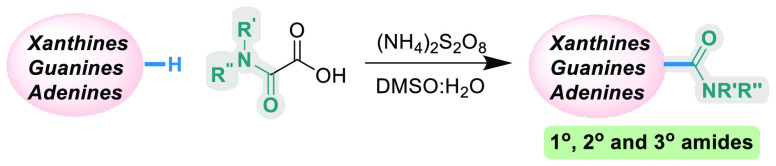

A method for the C–H carboxyamidation of purines has been developed that is capable of directly installing primary, secondary, and tertiary amides. Previous Minisci-type investigations on purines were limited to alkylations and arylations. Herein, we present the first method for the direct C–H amidation of a wide range of purines: xanthine, guanine, and adenine structures, including guanosine- and adenosine-type nucleosides. The Minisci-type reaction is also metal-free, cheap, operationally simple, scalable, and applicable to late-stage functionalizations of biologically important molecules.

Purine bases and nucleosides are the most widely occurring *N-*heterocycles in nature.^1^ In addition to forming the structural units in DNA and RNA, purines are also significant components of important biomolecules such as ATP, GTP, cAMP, CoA, and NADH and thus have important applications in biological and pharmaceutical chemistry.^1^ Methods for the direct C–H functionalization of purines, especially methods capable of late-stage functionalizations, would therefore be of great interest for the rapid synthesis of purine base analogues and derivatives. However, studies focused on Minisci-type C–H functionalizations^2^ of purines have so far been limited to C-6 alkylations^3^ and arylations^4^ on only the purine core **1** (rather than the wider class of purines) using silver-mediated strategies (Scheme 1A).^5^ There are currently no direct C–H amidation strategies focused on purine base motifs. Nevertheless, amidated purines have shown various activities such as nematocidal,^6^ PI3K^7^ and metalloproteinase^8^ inhibitor, anticancer,^9^ and antiviral^10^ activities and have been studied as ratiometric sensors^11^ and postconditioning agents.^12^ Amidated adenine UK-432097 was also exploited to map the internal structure of the A_2A_ adenosine receptor in detail by behaving as a conformationally selective agonist.^13^

**Scheme 1:**
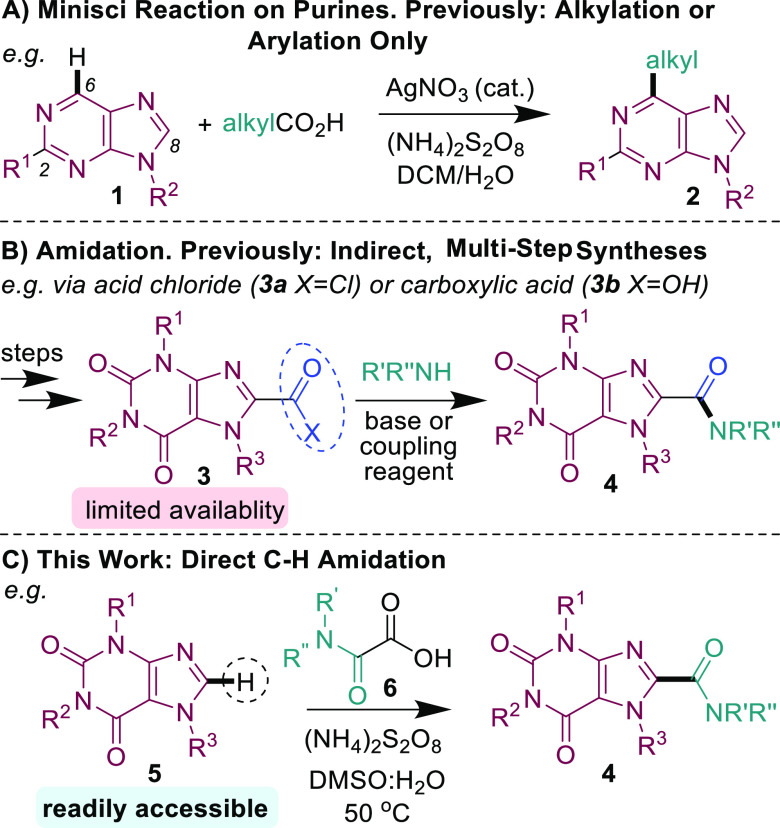
Functionalization of Purines

Previous methods of accessing C-amidated purine structures typically required lengthy multistep prefunctionalization of purines to the acid chloride^6^ or carboxylic acid reactant^9b,11,14^ (Scheme 1B).^5f,15^ Such reactants are often of limited availability and are synthesized in two or three steps from the parent purine.^9a,14a^ We herein present the first method for the direct C–H carboxyamidation of purine bases for the functionalization of a wide range of xanthine, guanine, and adenine structures, including guanosine- and adenosine-type nucleosides and many well-known drug molecules with these motifs (Scheme 1C). Furthermore, the reaction is metal-free, operationally simple, and scalable.

While several Minisci-type^16^ C–H amidations^17^ of *N*-heteroarenes via a carbamoyl radical were developed recently, including transition-metal-catalyzed,^18,19^ visible-light-mediated,^20^ electrocatalytic protocols^5f^ and the metal-free methodology pioneered by our group,^21−23^ none were tested on purines except for two reports that tested caffeine as part of the substrate scope studies.^20b,21a^ Decarboxylative photocatalytic conditions with caffeine and oxamic acids^24^**6** gave only a poor 42% yield of the product,^19b^ and **5a** also performed very poorly under our original metal-free general conditions (entry 1, Table 1). It is therefore clear that purines are much more challenging substrates than the (iso)quinoline structures typically employed for Minisci-type method development studies, and this limitation should be addressed due to the aforementioned importance of purines.

**Table 1:**
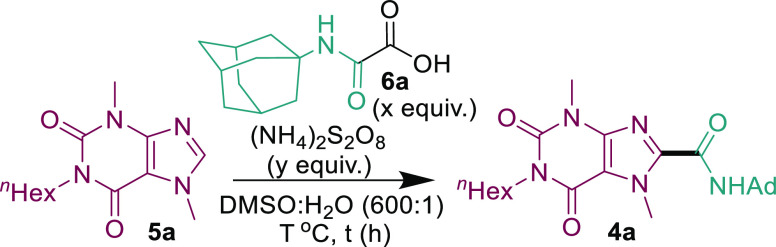
Selected Optimization Studies^a^

entry	*T*	*x*	*y*	*t*	conc. (M)	**5a** (%)	**4a** (%)
1	40	2	3	16	0.30	54	39
2	50	2	6	18	0.30	55	32
3	50	2	6	18	0.15	0	(71)^b^
4	40	2	6	18	0.15	8	75 (66)^b^
5	50	2	3	18	0.15	7	79
6	50	2	4	18	0.15	3	80
7	50	2	5	18	0.15	0	84
8	50	1.5	5	18	0.15	6	79
9	50	3	5	18	0.15	1	69
10	50	2	5	2	0.15	24	68
11	50	2	5	4	0.15	3	76
12	50	2	5	6	0.15	0	91 (83)^b^
13	rt	2	5	18	0.15	80	9
14	30	2	5	18	0.15	50	39
15	60	2	5	18	0.15	0	71

a The reactions were performed with 0.2 mmol **5a**. Yields were determined by ^1^H NMR analysis using trimethoxybenzene as internal standard unless otherwise stated.

b Isolated yield in parentheses.

We thus commenced our optimization with model substrate **5a** (Table 1), and dilution appeared to be the most important factor for improving the yield (entries 1 and 2 vs 3). Although the reaction proceeded well at 40 °C, performing the reaction at 50 °C appeared to give a slightly better conversion (entries 3 and 4). The conversion also improved slightly with more equivalents of persulfate (entries 5–7). Decreasing or increasing the equivalents of oxamic acid **6a** was detrimental to the reaction yield (entries 7–9), and a reaction time of 6 h was sufficient for full conversion with **5a** (entries 9–12). Entries 13 and 14 demonstrate that the temperature needs to be at least 40 °C for appreciable reactivity. Control reactions show that the reaction works equally well in the dark, requires persulfate for reactivity, and works best in wet DMSO (see the SI).

With the optimized conditions in hand, we began our investigation with the xanthine^25^ substrate scope (Scheme 2). Unlike that of the model substrate **5a** (83% **4a**), reactions with other substrates were not always complete within 6 h, so a more general reaction time of 18 h was adopted (e.g., **4b** from pentoxifylline). A second observation was that use of 3 equiv rather than 5 equiv of (NH_4_)_2_S_2_O_8_ tended to produce better yields (**4b**, **4e**–**g**, and **4i**). In cases where 5 equiv of (NH_4_)_2_S_2_O_8_ gave better yields, the increase was marginal (e.g., 88% vs 93% **4c**; 51% vs 56% **4d**), so 3 equiv of (NH_4_)_2_S_2_O_8_ and 18 h were adopted as the revised general optimized conditions.

**Scheme 2:**
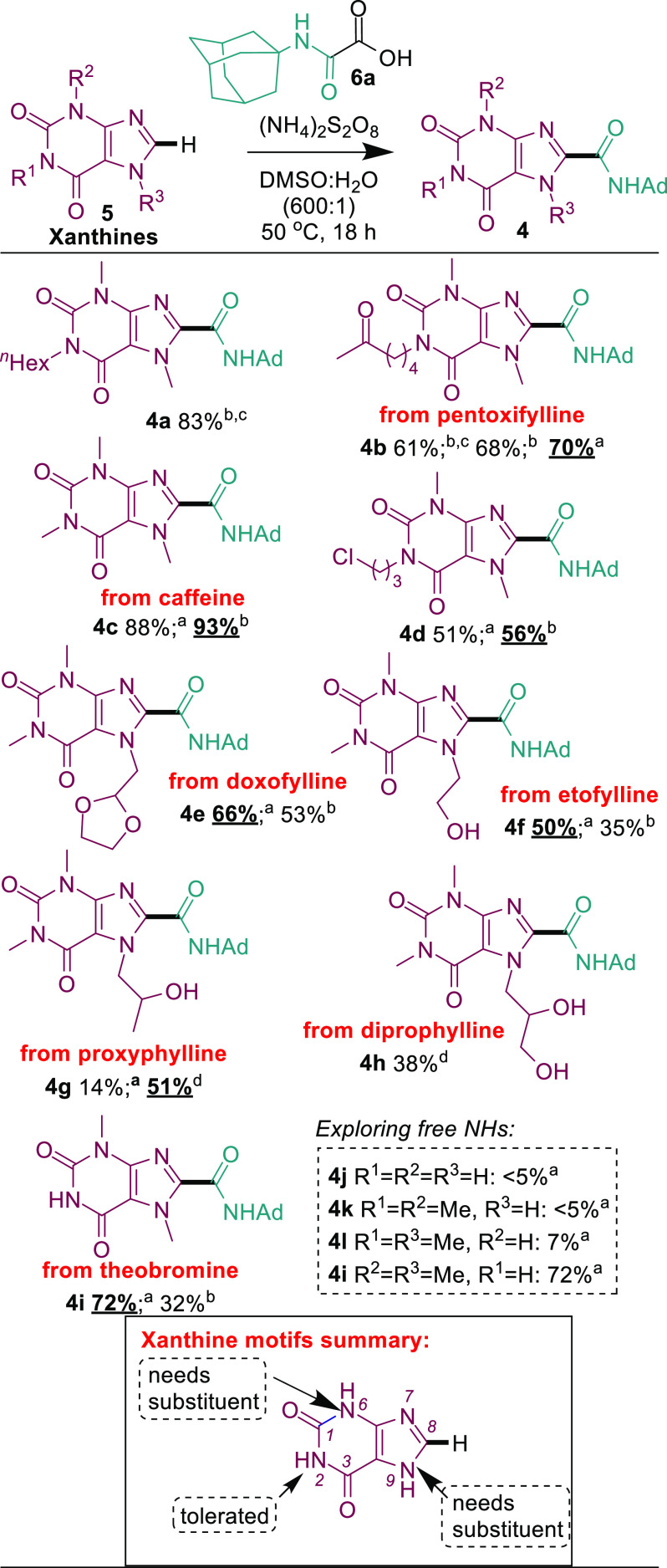
Xanthine Scope The reaction was performed with 3 equiv of (NH_4_)_2_S_2_O_8_. The reaction was performed with 5 equiv of (NH_4_)_2_S_2_O_8_. The reaction time was 6 h. The reaction temperature was 40 °C. Isolated yield reported using 0.2 mmol **5** and 0.4 mmol **6**.

We were pleased to find that the reactions showed functional group tolerance with ketone (70% **4b**), chloro (56% **4d**), acetal (66% **4e**), alcohol (50% **4f** and 51% **4g**), and diol (38% **4h**). The position of the free alcohol matters; while etofylline produced **4f** in a 50% yield under the standard conditions, proxyphylline gave a complex mixture (14% **4g**) under the same conditions. Pleasingly, running the reaction at a lower temperature of 40 °C solved the issue (51% **4g**). The successful reaction with theobromine (72% **4i**) but poor results with **4j**–**l** show that N-6 and N-9 need to be substituted for good yields, while a free N-2 is tolerated (Scheme 2).

Next, we investigated the more challenging guanine and adenine substrates for which there were no previously reported C–H amidations (Scheme 3). Although unprotected guanine itself yielded no desired product, acylated guanine produced **8a** in a moderate 31% yield, highlighting the need for protection at the C-2 amino group. This is exemplified again by the reaction with the antiviral drug acyclovir. The unprotected acyclovir was amidated in only a 11% yield, but the yield improved significantly to 65% upon the protection of the C-2 amino group (**8b**). Ac-protected antiviral ganciclovir was also amidated in a good yield (84% **8c**). Pleasingly, protected guanosine substrates also amidated smoothly (56% **8d** and 70% **8e**). While unprotected adenine itself yielded no desired product, benzoyl protection of the C-6 amino group improved the reactivity, and diamidated **10a** was formed in a 42% yield. Similarly, acylated adenosine was amidated twice at the C-2 and C-8 positions (39% **10a**). When C-2 is Cl- or F-substituted (e.g., the chemotherapy drug fludarabine), monoamidation occurs at C-8 (53% **10**c or 61% **10d**, respectively), while bromo-substitution at C-8 leads to monoamidation at C-2 in a 40% yield (**10e**). The tolerance to the Cl and Br functionalities in **10c** and **10e**, respectively, is pleasing as it provides an opportunity for further elaboration. Our substrate investigations also show that −NH_2_ needs to be protected in both guanine and adenine motifs, and yields are also improved by substitution at N-9 for guanine motifs (Scheme 3).

**Scheme 3:**
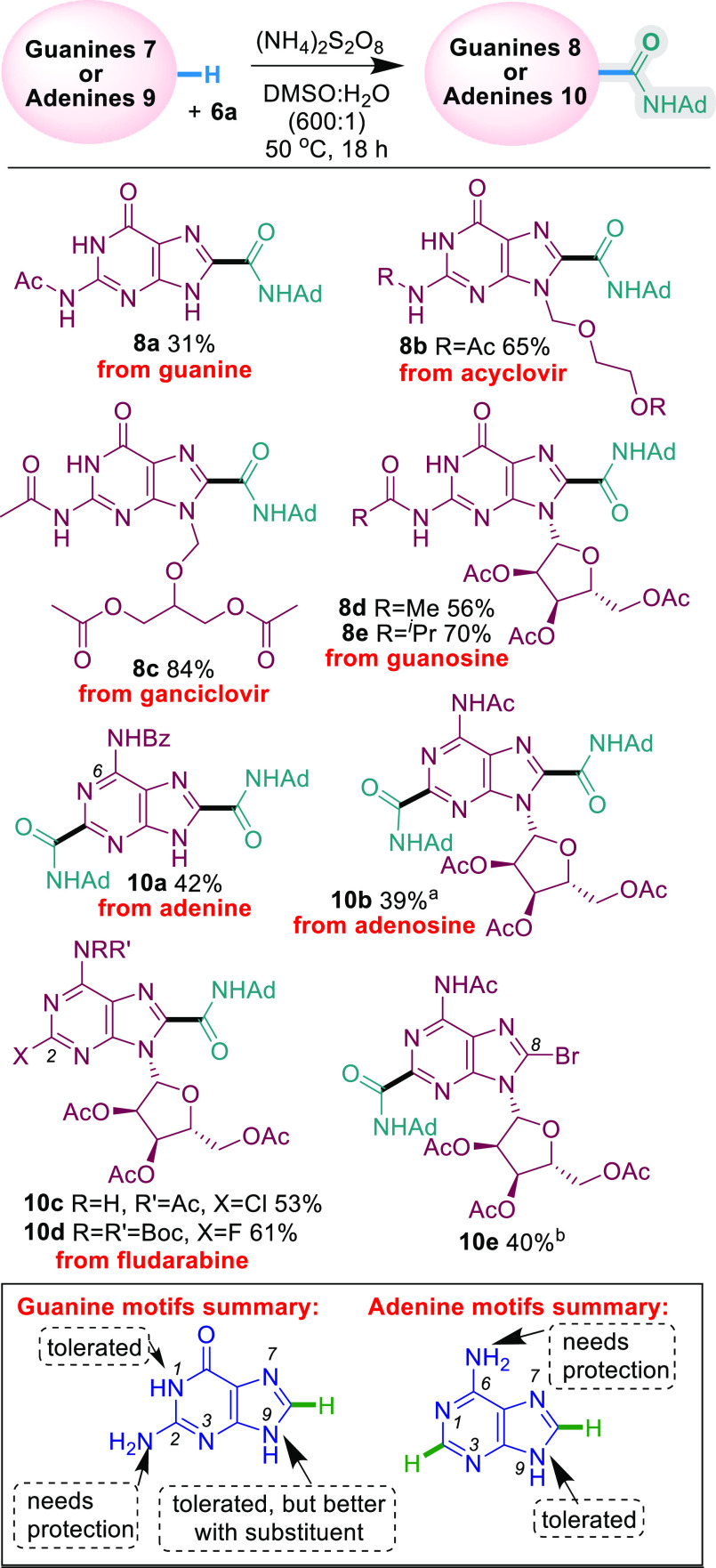
Guanine and Adenine Scope <5% **10** when the amounts of **6a** and reagents were doubled. At 70 °C; 32% at 50 °C. Isolated yields are reported for reactions performed with 0.2 mmol **7**/**9**, 0.4 mmol **6a**, and 0.6 mmol (NH_4_)_2_S_2_O_8_ unless otherwise stated.

Next, the oxamic acid scope was investigated (Scheme 4). A series of secondary amides with aliphatic substituents could be installed readily (47% to quantitative **4c** and **4m**–**q**). Amidation with aromatic substituents works better with electron-donating aryls (52–56%, **4r**-**s**), with the Ph- and Cl-substituted substrates suffering from low reactivities even at 70 °C (**4t** and **4u**, respectively). This is presumably due to the lower nucleophilicity of the corresponding carbamoyl radicals. Benzyl substitution was tolerated (73% **4v**), and a benzylic stereogenic center did not racemize under the reaction conditions (81% **4w**). Pleasingly, the CF_3_ moiety was also tolerated (73% **4x**). Tertiary amides were also installed smoothly (53–82% **4y**-**4ae**), including various ring-sizes (**4y**–**aa**), sterically hindering substituents (**4ab**), and F substituents (**4ad**). Finally, a primary amide was also readily installed (60%, **4af**).

**Scheme 4:**
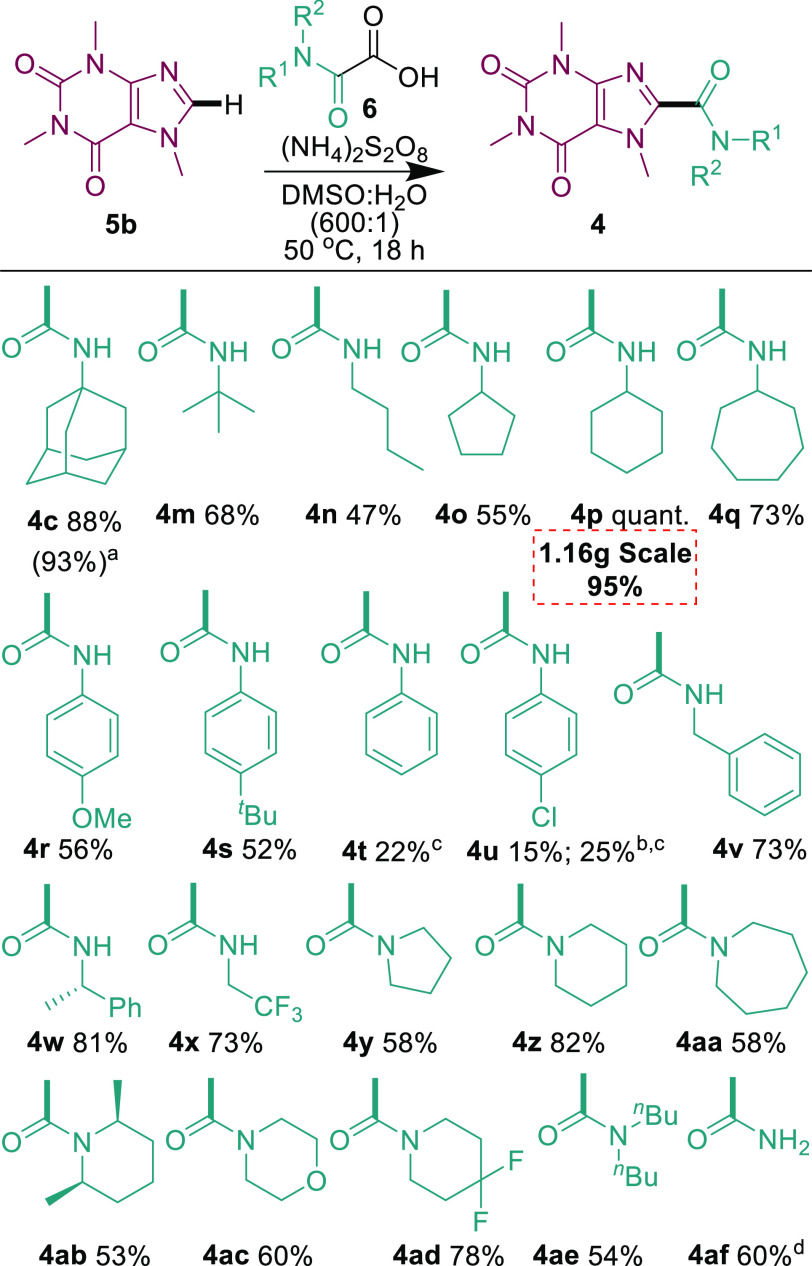
Oxamic Acid Scope The reaction was performed with 5 equiv of (NH_4_)_2_S_2_O_8_. Conversion based on starting material. The reaction was performed at 70 °C. **5a** was used due to solubility issues of product with **5b**. Isolated yields are reported for reactions performed with 0.2 mmol **5**, 0.4 mmol **6**, and 0.6 mmol (NH_4_)_2_S_2_O_8_ unless otherwise stated.

It should be noted that this operationally simple reaction is readily scaled up, with the 1.16 g scale reaction yielding 95% **4p**. As a comparison, previous methods for accessing **4p** involve amide formation with the C-8 acid chloride-functionalized caffeine **3a**, which is not commercially available and is made from the carboxylic acid derivative **3b**.^6^**3b** is one of the very few C-8 acid-functionalized purines that is commercially available; even then, it is only available from a very limited number of suppliers, many on demand (∼£1232 for 5 g).^26^ In contrast, the unfunctionalized substrate **5b** required for our method is widely available at a substantially reduced cost of £942 per 25 kg,^27^ i.e., 7 × 10^3^ times cheaper than **3b**. This showcases the significant advantage of being able to directly C–H amidate purines compared to previously available methods.

Based on previous literature precedent,^21,28^ the proposed mechanism is presented in Scheme 5. Deprotonation of **6** by the purine base (e.g., **5**) forms salt **I**. Meanwhile, the persulfate anion decomposes in a process accelerated by the DMSO solvent^29^ to give the oxidizing persulfate radical anion **II** (*E*_ox_ = +2.5–3.1 V vs SHE).^30^ SET occurs between **II** and the carboxylate anion **I** (*E*_ox_ = +1.32 V vs SCE)^31^ to produce the carboxylate radical,^32^ which then decarboxylates to release CO_2_ and the carbamoyl radical **III**.^17,33^ Radical **III** then adds to the activated purine substrate to produce **IV**. H radical abstraction by SO_4_^–**•**^ or SET via persulfate should furnish product **4**.

**Scheme 5:**
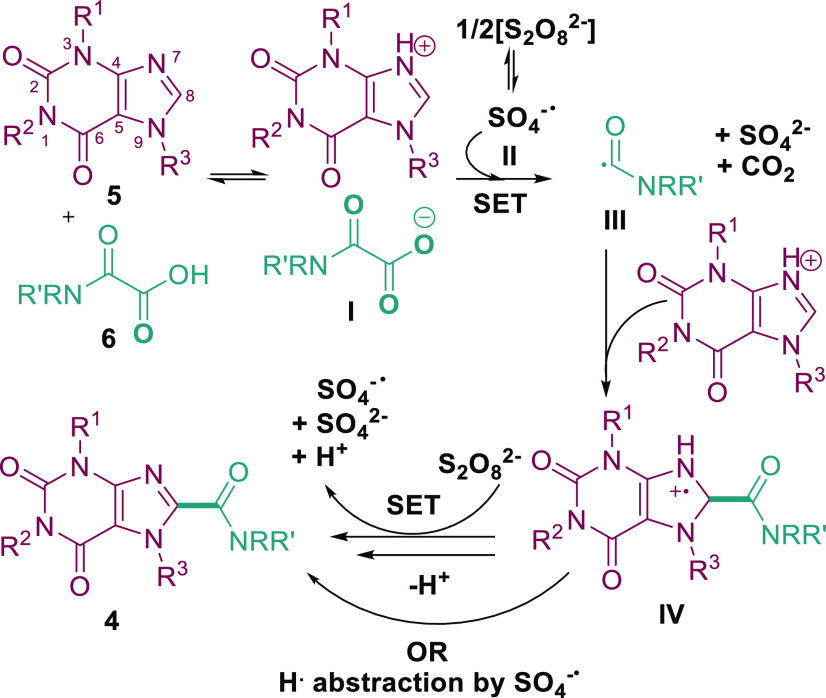
Proposed Mechanism

Finally, further modifications of the amidated purines were demonstrated (Scheme 6). Nitrile **11** was easily accessed from the primary amide **4af**, and the deprotection of **8e** to form amidated guanosine **12** was also straightforward. The tolerance of the Minisci amidation to halogen substituents also provides an opportunity for further elaboration via cross-couplings, as exemplified by the Suzuki coupling of **10c** to form **13**.

**Scheme 6:**
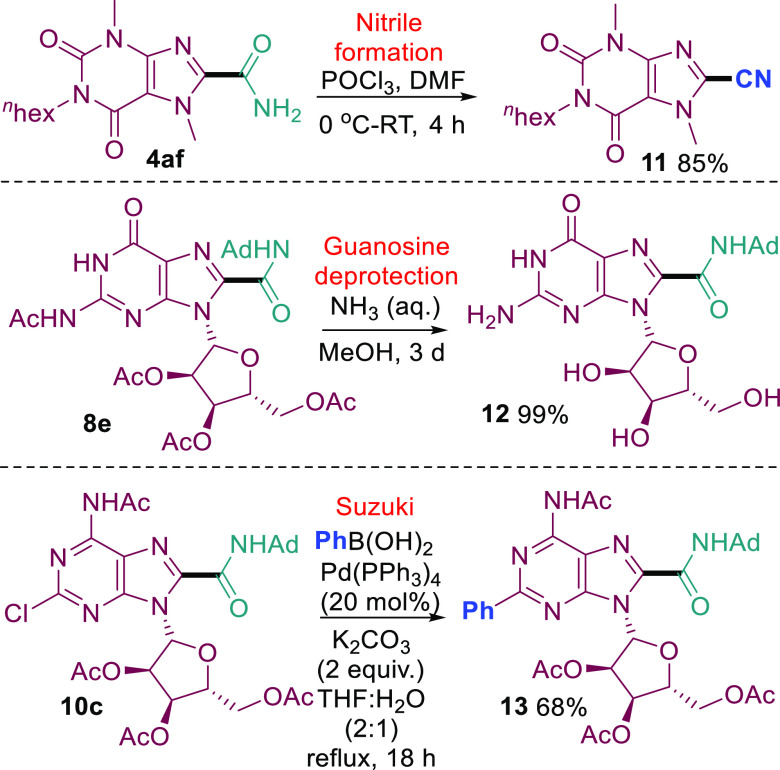
Further Modifications

In conclusion, the direct C–H carboxyamidation of a wide range of purine bases including xanthines, guanines, and adenines has been achieved for the first time, which should enable the facile synthesis of amidated analogues and derivatives of purine bases.

## Notes

The data underlying this study are available in the published article and its online Supporting Information. RAW data are available at 10.17861/2affc410-bad7-4f7a-9924-c5f516a6718f.

## Supporting Information Available

Optimization studies, all experimental details, characterization and copies of NMR data (PDF)
