# Supplementary material for: Direct Minisci-Type C–H Amidation of Purine Bases
Source: Org Lett. 2022 Oct 26;24(43):8008–13. doi: 10.1021/acs.orglett.2c03206 (PMC9641672; doi:10.1021/acs.orglett.2c03206)
Supplement: Supplementary file 1 — ol2c03206_si_001.pdf [file ol2c03206_si_001.pdf]

Supporting Information

Direct Minisci-type C-H Amidation of Purine Bases

David T. Mooney,[a] Peter R. Moore[b] and Ai-Lan Lee [a,*]

[a]Institute of Chemical Sciences, Heriot-Watt University, Edinburgh EH14 4AS, Scotland, U.K.

[b]Early Chemical Development, Pharmaceutical Sciences, R&D BioPharmaceuticals, AstraZeneca, Macclesfield SK10 2NA, U.K.

Table of Contents

1. General Experimental…… S-2
2. General Procedures…… S-3
3. Reaction Optimisation…… S-4
4. Starting Material Synthesis…… S-6
5. Product Characterisation…… S-12
6. NMR Spectra…… S-43
7. References…… S-94

1.General Experimental

Reagents were purchased from commercially available sources. All solvents employed were obtained from commercial sources. Reactions were heated using aluminium heating blocks. ^{1}H, ^{13}C and ^{19}F Nuclear Magnetic Resonances were recorded on Bruker® AV300 or AV400 (^{1}H NMR at 300 MHz or 400 MHz respectively, ^{13}C NMR at 75 MHz or 100 MHz respectively and ^{19}F NMR at 376 MHz) spectrometers with chemical shifts given in parts per million (ppm), employing DMSO-d_6, methanol-d_4 or chloroform-*d* as the solvent with residual $(CHD_2)CD_3SO$ (δ = 2.50), CHD_2OD (δ = 3.31) or $CHCl_3$ (δ = 7.26) as a standard reference peak, respectively. ^{13}C Nuclear Magnetic Resonances were recorded with total proton decoupling. The chemical shifts are reported relative to chloroform-*d* (δ = 77.16), methanol-d_4 (δ = 49.00) or DMSO-d_6 (δ = 39.52) as standard reference peaks. *J* values are given in Hz and br, s, d, t, q, quin, sextuplet, sept, multiplet respectively, or a combination of these. High resolution mass spectrometric (HRMS) data were reported with ion mass/charge (m/z) ratios as values in atomic mass units. High-Resolution Mass Spectra were recorded using a micrOTOF instrument under ESI conditions by the analytical services at the University of Edinburgh. Infrared spectra were obtained on Perkin-Elmer Spectrum 100 FT-IR Universal ATR Sampling Accessory, deposited neatly to a diamond/ZnSe plate. Column chromatography was carried out using Matrix silica gel 60 from Fluorochem. TLC was performed using Merck silica gel 60 F254 and visualised by UV (254 nm) and/or stained using aqueous acidic $KMnO_4$. “Ad” abbreviation in structures refers to 1-adamantyl.

2.General Procedures

General Procedure for Oxamic Acid Synthesis:

General Procedure A: Ethyl oxalyl chloride (11 mmol, 1.1 equiv.) was added dropwise to a solution of the desired amine (10 mmol, 1 equiv.) and triethylamine (11 mmol, 1.1 equiv.) at 0 ^oC. The reaction mixture was then allowed to warm to room temperature and stirred at room temperature for 3 h. 1 M HCl (aq.) (20 mL) was then added and the organic phase separated. The aqueous phase was then extracted with CH_2Cl_2 (3 x 30 mL) and the organic phases combined, washed with brine (70 mL), dried over Na_2SO_4 and the solvent removed *in vacuo*. The oily residue was then carried through to the next step without further purification. 1 M NaOH (aq.) (50 mL, 50 mmol, 5 equiv.) was added and the mixture stirred overnight at 30 ^oC or starting material consumption was monitored *via* TLC. The mixture was then acidified with 1 M HCl (aq.) and the product extracted with CH_2Cl_2 (3 x 30 mL). The organic phases were then combined, washed with brine (80 mL) and dried over Na_2SO_4, filtered, the solvent was then removed *in vacuo* to afford the product

General Procedures for Carbamoylation Reaction:

General Procedure B: A 4 mL vial equipped with a stirrer bar was purged with argon for 30 seconds, then sealed tightly. Purine (0.20 mmol, 1 equiv.), oxamic acid (0.40 mmol, 2 equiv.) and ammonium persulfate (0.60 mmol, 3 equiv.) were added quickly to the 4 mL screw top vial equipped with a magnetic stirrer bar, and then resealed. Separately, a Schlenk tube containing 600:1 DMSO:H_2O solvent mixture was purged with argon balloon for 10-15 mins. The 600:1 DMSO:H_2O mixture (1.3 mL/2.16 μL) was added to the 4 mL vial, and argon was blown into the opening for approx. 30 seconds. The vial was quickly sealed tight and stirred at 40 – 50 ^oC for 6 – 18 h. The reaction mixture was then diluted with CH_2Cl_2 (15 mL) and washed with sat. $NaHCO_3$ solution (80 mL). The aqueous phase was then extracted with CH_2Cl_2 (3 x 10 mL). The combined organic layers were then washed with brine (80 mL), dried over Na_2SO_4, filtered and concentrated *in vacuo* to give the crude product.

3.Reaction Optimisation

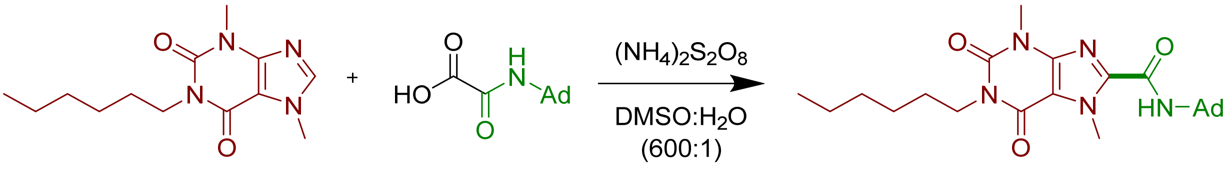

Entry	Temp. (°C)	Equiv. of 6a	Equiv. of $(NH_4)_2S_2O_8$	M (mol/L)	Rxn Time (h)	Remaining 5a (%)	Yield of 4a (%)[a]	Notes
1	50	2	6	0.15	18	0	(71)[b]	
2	40	2	6	0.15	18	8	75 (66)[b]	
3	50	2	3	0.15	18	7	79	
4	50	2	4	0.15	18	3	80	
5	50	2	5	0.15	18	0	84	
6	50	1.5	5	0.15	18	6	79	
7	50	3	5	0.15	18	1	69	
8	50	2	5	0.15	2	24	68	
9	50	2	5	0.15	4	3	76	
10	50	2	5	0.15	6	0	91 (83)[b]	
11	rt	2	5	0.15	18	80	9	
12	30	2	5	0.15	18	50	39	
13	50	2	5	0.15	6	0	86	In the dark
14	50	2	5	0.15	6	3	70	Dry DMSO
15	50	2	5	0.15	6	76	0	H_2O
16	50	2	5	0.15	6	99	0	MeCN
17	50	2	5	0.15	6	98	0	Acetone
18	50	2	5	0.15	6	55	35	DMSO:MeCN (1:1)
19	50	2	5	0.15	6	86	0	DMSO:H_2O (1:9)
20	50	2	5	0.15	6	76	10	DMSO:H_2O:MeCN (1:2:3)
21	50	2	5	0.15	6	63	14	$Na_2S_2O_8$ instead of $(NH_4)_2S_2O_8$
22	50	2	5	0.15	6	74	13	$K_2S_2O_8$ instead of $(NH_4)_2S_2O_8$
23	50	2	-	0.15	6	95	0	No persulfate
24	40	2	3	0.30	18	54	39	
25	50	2	6	0.30	18	55	32	

0.2 mmol of **5a**. [a]Determined by ^{1}H NMR analysis using trimethoxybenzene as internal standard, unless otherwise stated. [b]Isolated yield in parentheses.

Some studies were also carried out on 9-benzyl-9*H*-purine (**SI7**), but yields were generally low for this particular substrate, for example:

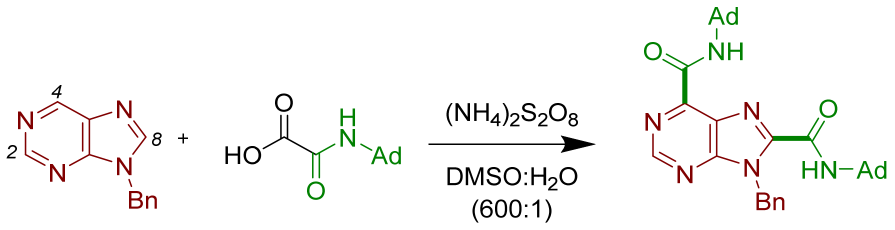

SI7 **6a** **SI8**

Entry	Temp. (°C)	Equiv. of 6a	Equiv. of $(NH_4)_2S_2O_8$	M (mol/L)	Rxn Time (h)	Remaining SI7 (%)	Yield of SI8 (%)[a]	Notes
1	50	2	3	0.15	6	0	21	Reaction selective for C4 and C8; no reaction at C2. No selectivity observed between C4 and C8.
2	50	4	6	0.15	18	0	27	Reaction selective for C4 and C8; no reaction at C2. No selectivity observed between C4 and C8.

0.2 mmol of **SI7**. [a]Isolated yield in parentheses.

In cases where reported reaction yields were below 50% (for example **8a**, **10a**, **10b**, **10e** and **4h**) only the product was observed in the crude NMR after work-up. No starting material or other identifiable by-products were isolated or detected by TLC or column chromatography.

4. Starting Material Synthesis

Preparation of Oxamic Acids

All oxamic acids were synthesised previously in the group following procedure A.[1, 2, 3, 4]

Preparation of Purines

(2*R*,3*R*,4*R*,5*R*)-2-(2-Acetamido-6-oxo-1,6-dihydro-9*H*-purin-9-yl)-5-(acetoxymethyl)tetrahydrofuran-3,4-diyl diacetate (SI1)

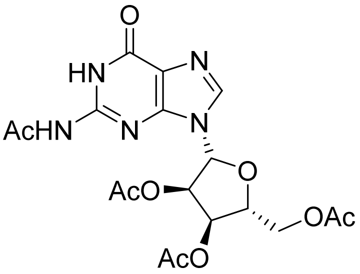

Guanosine (5.0957 g, 18.0 mmol, 1 equiv.), acetic anhydride (20 mL, 212.0 mmol, 12 equiv.) and 4-dimethylaminopyridine (2.3134 g, 18.0 mmol, 1 equiv.) were dissolved in pyridine (41 mL). The reaction mixture was then heated to 70 °C and left with continuous stirring for 3 h. After 3 h, the reaction was placed in an ice bath and methanol (20 mL) was added dropwise. The solvent was then reduced in *vacuo* and the reaction diluted in DCM (100 mL) and washed with water (100 mL). The aqueous was extracted with DCM (3x50 mL), the organic layers combined, washed with sat. sodium bicarbonate (150 mL), 1 M citric acid (2x150 mL), brine (2x150 mL), dried over sodium sulphate, filtered and the solvent removed in *vacuo* to afford (2*R*,3*R*,4*R*,5*R*)-2-(2-acetamido-6-oxo-1,6-dihydro-9*H*-purin-9-yl)-5-(acetoxymethyl)tetrahydrofuran-3,4-diyl diacetate **SI1** as an orange solid (5.0609 g, 11.16 mmol, 62%). ^{1}H NMR (300 MHz, Chloroform-*d*) δ 12.09 (s, 1H, NH), 9.93 (s, 1H, NH), 7.76 (s, 1H, Ar-H), 5.97 – 5.88 (m, 2H, CH_2), 5.65 (t, *J* = 4.5 Hz, 1H, CH), 4.55 (dd, *J* = 11.5, 4.5 Hz, 1H, CH), 4.45 – 4.37 (m, 1H, CH), 4.32 (dd, *J* = 11.5, 6.0 Hz, 1H, CH), 2.31 (s, 3H, CH_3), 2.10 (s, 3H, CH_3), 2.05 (s, 3H, CH_3), 2.04 (s, 3H, CH_3). ^{13}C NMR (75 MHz, $CDCl_3$) δ 172.7 (C), 171.3 (C), 169.8 (C), 169.5 (C), 155.7 (C), 147.9 (2xC, overlapping), 138.6 (CH), 122.5 (C), 87.5 (CH), 80.1 (CH), 72.8 (CH), 71.0 (CH), 63.3 (CH_2), 24.4 (CH_3), 20.9 (CH_3), 20.6 (CH_3), 20.5 (CH_3). ν_{max}/cm^{-1} 3146, 2940, 1745, 1674, 1608, 1557, 1481, 1403. M.p. = 95 – 98 °C. HRMS (ESI-TOF) m/z: $[M + H]^+$ Calcd for $C_{18}H_{21}N_5O_9H$ 452.1412; Found 452.1412. $[\alpha]_D^{20.2}$ -64 (c 1.00, $CHCl_3$). Data consistent with literature.[5]

(2*R*,3*R*,4*R*,5*R*)-2-(6-Acetamido-9*H*-purin-9-yl)-5-(acetoxymethyl)tetrahydrofuran-3,4-diyl diacetate (SI2)

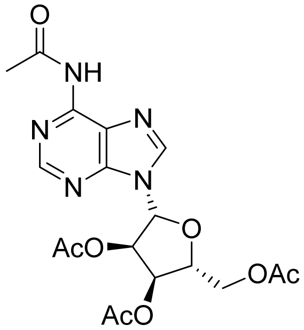

Adenosine (1.0055 g, 3.75 mmol, 1 equiv.) and acetic anhydride (3.6 mL, 37.5 mmol, 10 equiv.) were dissolved in pyridine (7.5 mL). The reaction mixture was then heated to 60 °C and left with continuous stirring for 24 h. After 24 h, the reaction mixture was allowed to cool to room temperature and ethanol (15 mL) was added to quench the reaction. The solvent was then removed in *vacuo* to afford a white foam. The foam was then dissolved in methanol (10 mL) and imidazole (205.7 mg, 3 mmol, 0.8 equiv.) was added. The reaction mixture was then left with continuous stirring at room temperature for 6 h. After 6 h, the reaction was diluted in ethyl acetate (80 mL) and washed with brine (4x80 mL). The organic layer was then dried over sodium sulphate, filtered and the solvent removed in *vacuo* to afford (2*R*,3*R*,4*R*,5*R*)-2-(6-acetamido-9*H*-purin-9-yl)-5-(acetoxymethyl)tetrahydrofuran-3,4-diyl diacetate **SI2** as a white foam (1.0915 g, 2.513 mmol, 67%). ^{1}H NMR (300 MHz, Chloroform-*d*) δ 8.69 (s, 1H, Ar-H), 8.58 (s, 1H, NH), 8.13 (s, 1H, Ar-H), 6.21 (d, J = 5.5 Hz, 1H, CH), 5.96 (t, J = 5.5 Hz, 1H, CH), 5.67 (dd, J = 5.5, 4.5 Hz, 1H, CH), 4.50 – 4.34 (m, 3H, CH and CH_2), 2.64 (s, 3H, CH_3), 2.16 (s, 3H, CH_3), 2.12 (s, 3H, CH_3), 2.08 (s, 3H, CH_3). ^{13}C NMR (75 MHz, $CDCl_3$) δ 170.6 (C), 170.5 (C), 169.7 (C), 169.5 (C), 152.8 (CH), 151.1 (C), 149.5 (C), 141.3 (CH), 122.4 (C), 86.7 (CH), 80.6 (CH), 73.3 (CH), 70.8 (CH), 63.2 (CH_2), 25.9 (CH_3), 20.9 (CH_3), 20.7 (CH_3), 20.5 (CH_3). HRMS (ESI-TOF) m/z: $[M + H]^+$ Calcd for $C_{18}H_{21}N_5O_8H$ 436.1463; Found 436.1463. $[\alpha]_D^{21.2}$ -28 (c 1.00, $CHCl_3$). Data consistent with literature.[6]

(2*R*,3*R*,4*R*,5*R*)-2-(Acetoxymethyl)-5-(2-isobutyramido-6-oxo-1,6-dihydro-9*H*-purin-9-yl)tetrahydrofuran-3,4-diyl diacetate (SI3)

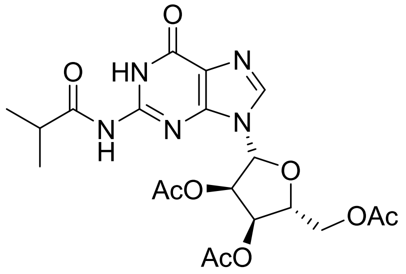

N-Isobutyrylguanosine (425.7 mg, 1.2 mmol, 1 equiv.), acetic anhydride (0.45 mL, 4.8 mmol, 4 equiv.) and 4-dimethylaminopyridine (74.3 mg, 0.6 mmol, 0.5 equiv.) were dissolved in MeCN (6 mL) and the reaction mixture left with continuous stirring at room temperature for 22 h. After 22 h, the solvent was removed in *vacuo* and the reaction diluted in DCM (15 mL) and washed with water (30 mL). The aqueous was then extracted with DCM (3x10 mL), the organic layers were combined, washed with sat. sodium bicarbonate (50 mL), brine (50 mL), dried over sodium sulphate, filtered and the solvent removed in *vacuo* to afford (2*R*,3*R*,4*R*,5*R*)-2-(acetoxymethyl)-5-(2-isobutyramido-6-oxo-1,6-dihydro-9*H*-purin-9-yl)tetrahydrofuran-3,4-diyl diacetate **SI3** as a colourless oil (321.2 mg, 0.672 mmol, 56%). ^{1}H NMR (400 MHz, Chloroform-*d*) δ 12.04 (s, 1H, NH), 8.94 (s, 1H, NH), 7.70 (s, 1H, Ar-H), 5.95 – 5.88 (m, 2H, 2xCH), 5.78 (t, J = 4.5 Hz, 1H, CH), 4.63 – 4.56 (m, 1H, CH), 4.50 – 4.42 (m, 2H, CH_2), 2.71 (hept, J = 7.0 Hz, 1H, CH), 2.14 (s, 3H, CH_3), 2.08 (s, 3H, CH_3), 2.07 (s, 3H, CH_3), 1.29 (d, J = 7.0 Hz, 3H, CH_3), 1.28 (d, J = 7.0 Hz, 3H, CH_3). ^{13}C NMR (101 MHz, $CDCl_3$) δ 178.9 (C), 171.5 (C), 169.9 (C), 169.5 (C), 155.5 (C), 147.9 (C), 147.6 (C), 138.4 (CH), 122.8 (C), 87.5 (CH), 80.1 (CH), 73.1 (CH), 71.1 (CH), 63.3 (CH_2), 36.7 (CH), 21.0 (CH_3), 20.7 (CH_3), 20.5 (CH_3), 19.1 (CH_3), 19.0 (CH_3). HRMS (ESI-TOF) m/z: $[M + H]^+$ Calcd for $C_{20}H_{25}N_5O_9H$ 480.1752; Found 480.1748. $[\alpha]_D^{21.3}$ -76 (c 1.00, $CHCl_3$). Data consistent with literature.[7]

(2*R*,3*R*,4*R*,5*R*)-2-(6-Acetamido-2-chloro-9*H*-purin-9-yl)-5-(acetoxymethyl)tetrahydrofuran-3,4-diyl diacetate (SI4)

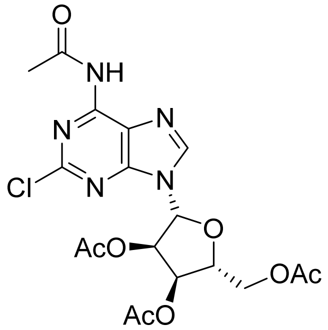

2-Chloroadenosine (457.4 mg, 1.5 mmol, 1 equiv.), acetic anhydride (2.3 mL) and sodium acetate (128.1 mg, 1.5 mmol, 1 equiv.) were added to a round bottom flask and left with continuous stirring at 80 ºC for 40 h. After 40 h, the solvent was removed in *vacuo* and the reaction diluted in ethyl acetate (10 mL) and washed with water (20 mL). The aqueous was then extracted with ethyl acetate (3x10 mL), the organic layers were combined, washed with sat. sodium bicarbonate (2x50 mL), brine (50 mL), dried over sodium sulphate, filtered and the solvent removed in *vacuo* to afford an orange oil. The crude was then purified *via* column chromatography (50:50, DCM:ethyl acetate) to afford (2*R*,3*R*,4*R*,5*R*)-2-(6-acetamido-2-chloro-9*H*-purin-9-yl)-5-(acetoxymethyl)tetrahydrofuran-3,4-diyl diacetate **SI4** as a colourless oil (158.3 mg, 0.345mmol, 23%). R_f= 0.18 (50:50, DCM:ethyl acetate). ^{1}H NMR (400 MHz, Chloroform-*d*) δ 8.78 (s, 1H, NH), 8.19 (s, 1H, Ar-H), 6.20 (d, *J* = 6.0 Hz, 1H, CH), 5.81 (t, *J* = 6.0 Hz, 1H, CH), 5.59 (dd, *J* = 6.0, 4.0 Hz, 1H, CH), 4.46 (td, *J* = 4.0, 3.0 Hz, 1H, CH), 4.41 (dd, *J* = 4.0, 2.0 Hz, 2H, CH_2), 2.67 (s, 3H, CH_3), 2.17 (s, 3H, CH_3), 2.14 (s, 3H, CH_3), 2.08 (s, 3H, CH_3). ^{13}C NMR (101 MHz, $CDCl_3$) δ 170.9 (C), 170.4 (C), 169.7 (C), 169.5 (C), 154.1 (C), 152.4 (C), 150.1 (C), 141.6 (CH), 121.0 (C), 86.3 (CH), 80.9 (CH), 73.4 (CH), 70.8 (CH), 63.1 (CH_2), 26.0 (CH_3), 20.9 (CH_3), 20.7 (CH_3), 20.5 (CH_3).ν_{max}/cm^{-1} 3240, 3126, 2941, 1742, 1706, 1606, 1581, 1521. HRMS (ESI-TOF) m/z: $[M + H]^+$ Calcd for $C_{18}H_{20}N_5O_8{}^{35}ClH$ 470.1073; Found 470.1079.

(2*R*,3*R*,4*R*,5*R*)-2-(Acetoxymethyl)-5-(6-(bis(tert-butoxycarbonyl)amino)-2-fluoro-9*H*-purin-9-yl)tetrahydrofuran-3,4-diyl diacetate (SI5)

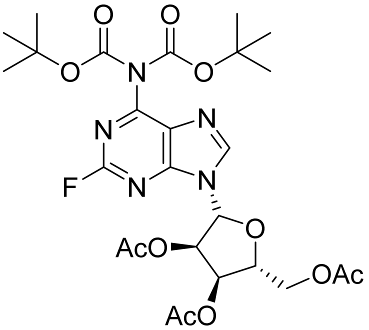

Fludarabine (106.0 mg, 0.375 mmol, 1 equiv.), acetic anhydride (0.58 mL) and sodium acetate (29.5 mg, 0.375 mmol, 1 equiv.) were added to a round bottom flask and left with continuous stirring at 80 °C for 18 h. After 18 h, the solvent was removed in *vacuo* and the reaction diluted in DCM (10 mL) and washed with water (20 mL). The aqueous was then extracted with DCM (3x10 mL), the organic layers were combined, washed with sat. sodium bicarbonate (30 mL), brine (30 mL), dried over sodium sulphate, filtered and the solvent removed in *vacuo* to afford a white solid. The solid was then dissolved in THF (1.5 mL) and 4-dimethylaminopyridine (4.62 mg, 0.375 mmol, 1 equiv.) was added. The reaction mixture was then allowed to cool to 0 °C and di-tert-butyldicarbonate (0.2 mL) was added. The reaction mixture was then allowed to warm to room temperature and left with continuous stirring for 18 h. After 18 h, the reaction was quenched with water (10 mL). The aqueous was extracted with ethyl acetate (3x10 mL), the organic layers were combined, washed with brine (40 mL), dried over sodium sulphate, filtered and the solvent removed in *vacuo* to afford an orange oil. The crude was then purified *via* column chromatography (50:50, hexane:ethyl acetate) to afford (2*R*,3*R*,4*R*,5*R*)-2-(acetoxymethyl)-5-(6-(bis(tert-butoxycarbonyl)amino)-2-fluoro-9*H*-purin-9-yl)tetrahydrofuran-3,4-diyl diacetate **SI5** as an orange oil (171.9 mg, 0. 281 mmol, 75%). R_f = 0.26 (50:50, hexane:ethyl acetate). ^{1}H NMR (400 MHz, Chloroform-*d*) δ 8.24 (s, 1H, Ar-H), 6.56 (d, *J* = 4.5 Hz, 1H, CH), 5.50 (dd, *J* = 5.0, 3.0 Hz, 1H, CH), 5.37 (dd, *J* = 4.0, 3.0 Hz, 1H, CH), 4.46 (d, *J* = 5.5 Hz, 2H, CH_2), 4.28 (ddd, *J* = 5.5, 5.0, 4.0 Hz, 1H, CH), 2.18 (s, 3H, CH_3), 2.13 (s, 3H, CH_3), 1.91 (s, 3H, CH_3), 1.46 (s, 18H, 6xCH_3). ^{13}C NMR (101 MHz, $CDCl_3$) δ 170.6 (C), 169.7 (C), 168.5 (C), 158.0 (d, *J* = 218.0 Hz, CF), 154.6 (d, *J* = 17.0 Hz, C), 152.4 (d, *J* = 17.0 Hz, C), 149.9 (C), 143.8 (d, *J* = 3.0 Hz, CH), 126.7 (d, *J* = 5.0 Hz, C), 84.5 (C), 83.5 (CH), 80.5 (CH), 75.8 (CH), 74.9 (CH), 62.8 (CH_2), 27.9 (CH_3), 20.90 (CH_3), 20.85 (CH_3),

20.4 (CH_3). ν_{max}/cm^{-1} 2980, 2937, 1792, 1745, 1606, 1592, 1506, 1476. HRMS (ESI-TOF) m/z: $[M + H]^+$ Calcd for $C_{26}H_{34}N_5O_{11}FH$ 612.2312; Found 612.2308.

(2*R*,3*R*,4*R*,5*R*)-2-(6-Acetamido-8-bromo-9*H*-purin-9-yl)-5-(acetoxymethyl)tetrahydrofuran-3,4-diyl diacetate (SI6)

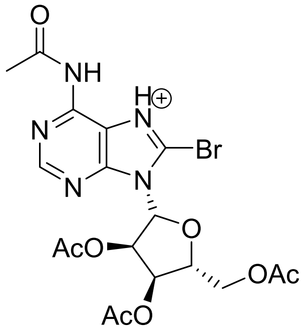

(2*R*,3*R*,4*S*,5*R*)-2-(6-Amino-8-bromo-9*H*-purin-9-yl)-5-(hydroxymethyl)tetrahydrofuran-3,4-diol (519.8 mg, 1.5 mmol, 1 equiv.), acetic anhydride (2.4 mL) and sodium acetate (122.0 mg, 1.5 mmol, 1 equiv.) were added to a round bottom flask and left with continuous stirring at 80 °C for 19 h. After 19 h, the solvent was removed in *vacuo* and the reaction diluted in DCM (20 mL) and washed with water (30 mL). The aqueous was then extracted with DCM (3x10 mL), the organic layers were combined, washed with sat. sodium bicarbonate (30 mL), brine (50 mL), dried over sodium sulphate, filtered and the solvent removed in *vacuo* to afford a brown oil. The crude was then purified *via* column chromatography (90:10, DCM:ethyl acetate) to afford (2*R*,3*R*,4*R*,5*R*)-2-(6-acetamido-8-bromo-9*H*-purin-9-yl)-5-(acetoxymethyl)tetrahydrofuran-3,4-diyl diacetate **SI6** as a colourless oil (137.9 mg, 0.465 mmol, 31%). R_f = 0.08 (90:10, DCM:ethyl acetate). ^1H NMR (400 MHz, Chloroform-*d*) δ 9.40 (s, 1H, NH), 8.38 (s, 1H, NH), 8.34 (s, 1H, Ar-H), 6.22 (dd, *J* = 6.0, 4.0 Hz, 1H, CH), 6.12 (d, *J* = 4.0 Hz, 1H, CH), 5.80 (t, *J* = 6.0 Hz, 1H, CH), 4.49 (dd, *J* = 11.5, 3.5 Hz, 1H, CH), 4.38 – 4.25 (m, 2H, CH_2), 2.28 (s, 3H, CH_3), 2.14 (s, 3H, CH_3), 2.11 (s, 3H, CH_3), 2.07 (s, 3H, CH_3). ^{13}C NMR (101 MHz, $CDCl_3$) δ 170.8 (C), 170.0 (C), 169.8 (C), 169.7 (C), 150.8 (2xC), 150.5 (CH), 137.5 (C), 108.8 (C), 84.5 (CH), 79.5 (CH), 72.0 (CH), 70.7 (CH), 63.4 (CH_2), 24.1 (CH_3), 20.9 (CH_3), 20.7 (2xCH_3). ν_{max}/cm^{-1} 3314, 3206, 3134, 3028, 2972, 1744, 1692, 1626, 1594, 1549, 1525, 1472. $[\alpha]_D^{22.6}$ +8 (c 1.00, $CHCl_3$). HRMS (ESI-TOF) m/z: $[M + Na]^+$ Calcd for $C_{18}H_{20}N_5O_8{}^{79}BrNa$ 537.2738; Found 537.2733.

5. Product Characterisation

***N*-(Adamantan-1-yl)-1-hexyl-3,7-dimethyl-2,6-dioxo-2,3,6,7-tetrahydro-1*H*-purine-8-carboxamide (4a)**

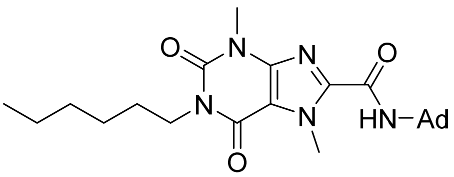

General procedure B was followed: 1-hexyltheobromide (53.0 mg, 0.20 mmol, 1 equiv.), 2-((adamantan-1-yl)amino)-2-oxoacetic acid (89.9 mg, 0.40 mmol, 2 equiv.) and $(NH_4)S_2O_8$ (230.6 mg, 1.00 mmol, 5 equiv.) in DMSO:H_2O, 600:1 (1.3/2.16 mL/µL) at 50 ºC for 6 h. The crude product was then purified *via* column chromatography (80:20 hexane/ethyl acetate) to afford *N*-((3*s*,5*s*,7*s*)-adamantan-1-yl)-1-hexyl-3,7-dimethyl-2,6-dioxo-2,3,6,7-tetrahydro-1*H*-purine-8-carboxamide **4a** as a white solid (73.0 mg, 0.165 mmol, 83%). R_f = 0.56 (80:20 hexane/ethyl acetate). ^{1}H NMR (400 MHz, Chloroform-*d*) δ 7.15 (s, 1H, NH), 4.37 (s, 3H, CH_3), 4.02 – 3.96 (m, 2H, CH_2), 3.54 (s, 3H, CH_3), 2.13 (app. s, 9H, 3xCH and 3xCH_2), 1.73 (app. s, 6H, 3xCH_2), 1.67 – 1.60 (m, 2H, CH_2), 1.40 – 1.27 (m, 6H, 3xCH_2), 0.87 (t, *J* = 7.1 Hz, 3H, CH_3). ^{13}C NMR (101 MHz, $CDCl_3$) δ 157.2 (C), 155.5 (C), 151.4 (C), 146.1 (C), 141.7 (C), 110.2 (C), 52.7 (C), 41.7 (CH_2), 41.5 (CH_2), 36.4 (CH_2), 34.7 (CH_3), 31.6 (CH_2), 29.8 (CH_3), 29.5 (CH), 28.0 (CH_2), 26.7 (CH_2), 22.6 (CH_2), 14.1 (CH_3). ν_{max}/cm^{-1} 3389, 2908, 2851, 2252, 1709, 1662, 1603, 1524. M.p. = 149 – 151 ºC. HRMS (ESI-TOF) m/z: $[M + H]^+$ Calcd for $C_{24}H_{35}N_5O_3H$ 442.2813; Found 442.2810.

***N*-(Adamantan-1-yl)-3,7-dimethyl-2,6-dioxo-1-(5-oxohexyl)-2,3,6,7-tetrahydro-1*H*-purine-8-carboxamide (4b)**

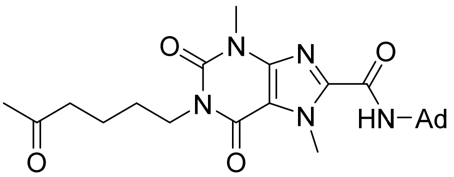

General procedure B was followed: 3,7-dimethyl-1-(5-oxohexyl)-3,7-dihydro-1*H*-purine-2,6-dione (56.3 mg, 0.20 mmol, 1 equiv.), 2-((adamantan-1-yl)amino)-2-oxoacetic acid (89.4 mg, 0.40 mmol, 2 equiv.) and $(NH_4)S_2O_8$ (137.4 mg, 0.60 mmol, 3 equiv.) in DMSO:H_2O, 600:1 (1.3/2.16 mL/µL) at 50 ºC for 18 h. The crude product was then purified *via* column chromatography (60:40 hexane/ethyl acetate) to afford *N*-((3*s*,5*s*,7*s*)-adamantan-1-yl)-3,7-

dimethyl-2,6-dioxo-1-(5-oxohexyl)-2,3,6,7-tetrahydro-1*H*-purine-8-carboxamide **4b** as a white solid (63.4 mg, 0.139 mmol, 70%). R_f = 0.34 (60:40 hexane/ethyl acetate). ^{1}H NMR (400 MHz, Chloroform-*d*) δ 7.13 (s, 1H, NH), 4.35 (s, 3H, CH_3), 3.98 (t, *J* = 7.0 Hz, 2H, CH_2), 3.52 (s, 3H, CH_3), 2.48 (t, *J* = 7.0 Hz, 2H, CH_2), 2.15 – 2.10 (m, 12H, 1xCH_3, 3xCH and 3xCH_2), 1.71 (app. s, 6H, 3xCH_2), 1.67 – 1.59 (m, 4H, 2xCH_2). ^{13}C NMR (101 MHz, $CDCl_3$) δ 208.7 (C), 157.2 (C), 155.5 (C), 151.4 (C), 146.1 (C), 141.8 (C), 110.1 (C), 52.8 (C), 43.2 (CH_2), 41.5 (CH_2), 41.1 (CH_2), 36.4 (CH_2), 34.7 (CH_3), 30.0 (CH_3), 29.8 (CH_3), 29.5 (CH), 27.5 (CH_2), 21.0 (CH_2). ν_{max}/cm^{-1} 3388, 2907, 2850, 2251, 1708, 1660, 1602, 1524, 1476. M.p. = 157 – 160 °C. HRMS (ESI-TOF) m/z: $[M + H]^+$ Calcd for $C_{24}H_{33}N_5O_4H$ 456.3605; Found 456.2595.

_N_-(Adamantan-1-yl)-1,3,7-trimethyl-2,6-dioxo-2,3,6,7-tetrahydro-1*H*-purine-8-carboxamide (4c)

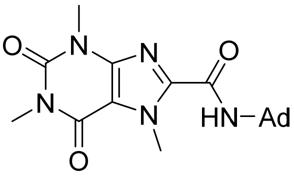

General procedure B was followed: caffeine (39.5 mg, 0.20 mmol, 1 equiv.), 2-((adamantan-1-yl)amino)-2-oxoacetic acid (89.0 mg, 0.40 mmol, 2 equiv.) and $(NH_4)S_2O_8$ (228.3 mg, 1.00 mmol, 5 equiv.) in DMSO:H_2O, 600:1 (1.3/2.16 mL/μL) at 50 °C for 18 h. The crude product was then purified *via* column chromatography (70:30 hexane/ethyl acetate) to afford *N*-((3*s*,5*s*,7*s*)-adamantan-1-yl)-1,3,7-trimethyl-2,6-dioxo-2,3,6,7-tetrahydro-1*H*-purine-8-carboxamide **4c** as a white solid (68.7 mg, 0.185 mmol, 93%). R_f = 0.45 (70:30 hexane/ethyl acetate). ^{1}H NMR (400 MHz, Chloroform-*d*) δ 7.15 (s, 1H, NH), 4.38 (s, 3H, CH_3), 3.55 (s, 3H, CH_3), 3.40 (s, 3H, CH_3), 2.13 (app. s, 9H, 3xCH and 3xCH_2), 1.73 (app. s, 6H, 3xCH_2). ^{13}C NMR (101 MHz, $CDCl_3$) δ 157.3 (C), 155.7 (C), 151.7 (C), 146.1 (C), 141.8 (C), 110.2 (C), 52.8 (C), 41.6 (CH_2), 36.4 (CH_2), 34.8 (CH_3), 29.9 (CH_3), 29.5 (CH), 28.2 (CH_3). ν_{max}/cm^{-1} 3382, 2950, 2904, 2850, 2253, 1714, 1677, 1654, 1595, 1525. M.p. = 280 – 283 °C. HRMS (ESI-TOF) m/z: $[M + H]^+$ Calcd for $C_{19}H_{25}N_5O_3H$ 372.2030; Found 372.2033.

_N_-(Adamantan-1-yl)-1-(3-chloropropyl)-3,7-dimethyl-2,6-dioxo-2,3,6,7-tetrahydro-1*H*-purine-8-carboxamide (4d)

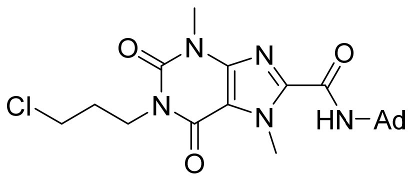

General procedure B was followed: 1-(3-chloropropyl)-3,7-dimethyl-3,7-dihydro-1*H*-purine-2,6-dione (51.6 mg, 0.20 mmol, 1 equiv.), 2-((adamantan-1-yl)amino)-2-oxoacetic acid (89.2 mg, 0.40 mmol, 2 equiv.) and $(NH_4)S_2O_8$ (227.9 mg, 1.00 mmol, 5 equiv.) in DMSO:H_2O, 600:1 (1.3/2.16 mL/µL) at 50 °C for 18 h. The crude product was then purified *via* column chromatography (80:20 hexane/ethyl acetate) to afford *N*-((3*s*,5*s*,7*s*)-adamantan-1-yl)-1-(3-chloropropyl)-3,7-dimethyl-2,6-dioxo-2,3,6,7-tetrahydro-1*H*-purine-8-carboxamide **4d** as a white solid (48.2 mg, 0.112 mmol, 56%). R_f = 0.33 (80:20 hexane/ethyl acetate). ^{1}H NMR (300 MHz, Chloroform-*d*) δ 7.15 (s, 1H, NH), 4.37 (s, 3H, CH_3), 4.16 (t, *J* = 7.0 Hz, 2H, CH_2), 3.60 (t, *J* = 7.0 Hz, 2H, CH_2), 3.55 (s, 3H, CH_3), 2.21 – 2.10 (m, 11H, 3xCH, 4xCH_2), 1.73 (app. s, 6H, 3xCH_2). ^{13}C NMR (75 MHz, $CDCl_3$) δ 157.2 (C), 155.5 (C), 151.4 (C), 146.3 (C), 142.0 (C), 110.1 (C), 52.8 (C), 42.7 (CH_2), 41.6 (CH_2), 39.6 (CH_2), 36.4 (CH_2), 34.8 (CH_3), 31.3 (CH_2), 29.9 (CH_3), 29.5 (CH). ν_{max}/cm^{-1} 3394, 3356, 2908, 2848, 1711, 1663, 1597, 1530, 1476. M.p. = 182 – 185 °C. HRMS (ESI-TOF) m/z: $[M + H]^+$ Calcd for $C_{21}H_{28}{}^{35}Cl_1N_5O_3H$ 434.1953; Found 434.1957.

7-((1,3-Dioxolan-2-yl)methyl)-*N*-(adamantan-1-yl)-1,3-dimethyl-2,6-dioxo-2,3,6,7-tetrahydro-1*H*-purine-8-carboxamide (4e)

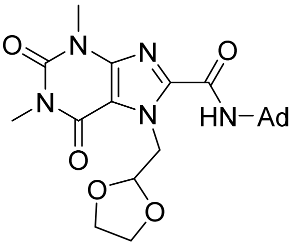

General procedure B was followed: 7-((1,3-dioxolan-2-yl)methyl)-1,3-dimethyl-3,7-dihydro-1*H*-purine-2,6-dione (53.7 mg, 0.20 mmol, 1 equiv.), 2-((adamantan-1-yl)amino)-2-oxoacetic acid (90.4 mg, 0.40 mmol, 2 equiv.) and $(NH_4)S_2O_8$ (136.9 mg, 0.60 mmol, 3 equiv.) in DMSO:H_2O, 600:1 (1.3/2.16 mL/µL) at 50 °C for 18 h. The crude product was then purified *via* column chromatography (70:30 hexane/ethyl acetate) to afford **7**-((1,3-dioxolan-2-yl)methyl)-*N*-((3*s*,5*s*,7*s*)-adamantan-1-yl)-1,3-dimethyl-2,6-dioxo-2,3,6,7-tetrahydro-1*H*-purine-8-carboxamide **4e** as a white solid (58.7 mg, 0.132 mmol, 66%). R_f = 0.27 (70:30 hexane/ethyl acetate). ^{1}H NMR (300 MHz, Chloroform-*d*) δ 7.11 (s, 1H, NH), 5.41 (t, *J* = 4.8 Hz, 1H, CH), 5.08 (d, *J* = 4.8 Hz, 2H, CH_2), 4.02 – 3.93 (m, 2H, CH_2), 3.92 – 3.83 (m, 2H, CH_2), 3.56 (s, 3H, CH_3), 3.41 (s, 3H, CH_3), 2.13 (app. s, 9H, 3xCH and 3xCH_2), 1.73 (app. s, 6H, 3xCH_2). ^{13}C NMR (75 MHz, $CDCl_3$) δ 157.1 (C), 155.5 (C), 151.7 (C), 146.2 (C), 142.4 (C), 109.9 (C), 101.9 (CH), 65.2 (CH_2), 52.8 (C), 48.3 (CH_2), 41.5 (CH_2), 36.4 (CH_2), 30.0

(CH_3), 29.5 (CH), 28.3 (CH_3). ν_{max}/cm^{-1} 3387, 2908, 2851, 2250, 1709, 1666, 1605, 1526, 1462. M.p. = 191 – 194 °C. HRMS (ESI-TOF) m/z: $[M + H]^+$ Calcd for $C_{22}H_{29}N_5O_5H$ 444.2255; Found 444.2241.

_N_-(Adamantan-1-yl)-7-(2-hydroxyethyl)-1,3-dimethyl-2,6-dioxo-2,3,6,7-tetrahydro-1_H_-purine-8-carboxamide (4f)

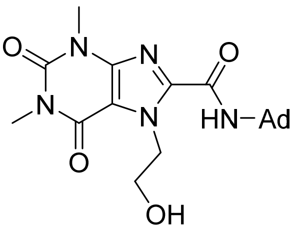

General procedure B was followed: 7-(2-hydroxyethyl)-1,3-dimethyl-3,7-dihydro-1*H*-purine-2,6-dione (45.4 mg, 0.20 mmol, 1 equiv.), 2-((adamantan-1-yl)amino)-2-oxoacetic acid (89.9 mg, 0.40 mmol, 2 equiv.) and $(NH_4)S_2O_8$ (137.6 mg, 0.60 mmol, 3 equiv.) in DMSO:H_2O, 600:1 (1.3/2.16 mL/µL) at 50 °C for 18 h. The crude product was then purified *via* column chromatography (60:40 hexane/ethyl acetate) to afford *N*-((3*s*,5*s*,7*s*)-adamantan-1-yl)-7-(2-hydroxyethyl)-1,3-dimethyl-2,6-dioxo-2,3,6,7-tetrahydro-1*H*-purine-8-carboxamide **4f** as a white solid (40.5 mg, 0.100 mmol, 50%). R_f = 0.24 (60:40 hexane/ethyl acetate). ^{1}H NMR (300 MHz, Chloroform-*d*) δ 7.21 (s, 1H, NH), 5.06 (t, *J* = 5.0 Hz, 2H, CH_2), 4.02 (app. q, *J* = 6.0 Hz, 2H, CH_2), 3.57 (s, 3H, CH_3), 3.41 (s, 3H, CH_3), 3.21 (t, *J* = 6.0 Hz, 1H, OH), 2.12 (app. s, 9H, 3xCH and 3x CH_2), 1.73 (app. s, 6H, 3xCH_2). ^{13}C NMR (75 MHz, $CDCl_3$) δ 157.7 (C), 156.1 (C), 151.5 (C), 146.4 (C), 142.4 (C), 110.1 (C), 62.9 (CH_2), 53.1 (C), 49.1 (CH_2), 41.5 (CH_2), 36.4 (CH_2), 30.0 (CH_3), 29.5 (CH), 28.4 (CH_3). ν_{max}/cm^{-1} 3466 (br), 3386, 2908, 2851, 2250, 1709, 1664, 1603, 1528, 1465. M.p. = 179 – 182 °C. HRMS (ESI-TOF) m/z: $[M + H]^+$ Calcd for $C_{20}H_{27}N_5O_4H$ 402.2136; Found 402.2156.

_N_-(Adamantan-1-yl)-7-(2-hydroxypropyl)-1,3-dimethyl-2,6-dioxo-2,3,6,7-tetrahydro-1_H_-purine-8-carboxamide (4g)

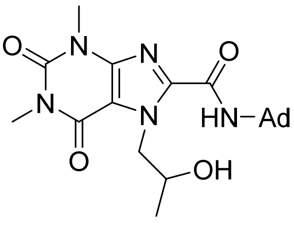

General procedure B was followed: 7-(2-hydroxypropyl)-1,3-dimethyl-3,7-dihydro-1*H*-purine-2,6-dione (47.8 mg, 0.20 mmol, 1 equiv.), 2-((adamantan-1-yl)amino)-2-oxoacetic acid (89.4 mg, 0.40 mmol, 2 equiv.) and $(NH_4)S_2O_8$ (137.1 mg, 0.60 mmol, 3 equiv.) in DMSO:H_2O, 600:1 (1.3/2.16 mL/μL) at 40 °C for 18 h. The crude product was then purified *via* column chromatography (60:40 hexane/ethyl acetate) to afford *N*-((3*s*,5*s*,7*s*)-adamantan-1-yl)-7-(2-hydroxypropyl)-1,3-dimethyl-2,6-dioxo-2,3,6,7-tetrahydro-1*H*-purine-8-carboxamide **4g** as a white solid (42.1 mg, 0.102 mmol, 51%). R_f = 0.28 (60:40 hexane/ethyl acetate). ^{1}H NMR (400 MHz, Chloroform-*d*) δ 7.24 (s, 1H, NH), 4.95 (dd, *J* = 13.5, 3.5 Hz, 1H, C*H*H, 4.85 (dd, *J* = 13.5, 8.5 Hz, 1H, CH*H*), 4.20 – 4.09 (m, 1H, CH), 3.57 (s, 3H, CH_3), 3.42 – 3.39 (m, 4H, OH and CH_3), 2.18 – 2.10 (m, 9H. 3xCH and 3xCH_2), 1.73 (app. s, 6H, 3x CH_2), 1.31 (d, *J* = 6.0 Hz, 3H, CH_3). ^{13}C NMR (101 MHz, $CDCl_3$) δ 157.8 (C), 156.2 (C), 151.5 (C), 146.3 (C), 142.4 (C), 110.3 (C), 68.3 (CH), 53.2 (C), 53.1 (CH_2), 41.5 (CH_2), 36.4 (CH_2), 30.0 (CH_3), 29.5 (CH), 28.4 (CH_3), 21.5 (CH_3). ν_{max}/cm^{-1} 3465 (br), 2909, 2851, 1710, 1669, 1603, 1528, 1467. M.p. = 205 – 208 °C. HRMS (ESI-TOF) m/z: $[M + H]^+$ Calcd for $C_{21}H_{29}N_5O_4H$ 416.2292; Found 416.2293.

N-(Adamantan-1-yl)-7-(2,3-dihydroxypropyl)-1,3-dimethyl-2,6-dioxo-2,3,6,7-tetrahydro-1*H*-purine-8-carboxamide (4h)

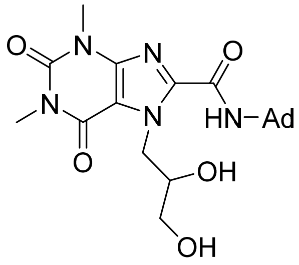

General procedure B was followed: 7-(2,3-dihydroxypropyl)-1,3-dimethyl-3,7-dihydro-1H-purine-2,6-dione (51.0 mg, 0.20 mmol, 1 equiv.), 2-((adamantan-1-yl)amino)-2-oxoacetic acid (89.7 mg, 0.40 mmol, 2 equiv.) and $(NH_4)S_2O_8$ (136.8 mg, 0.60 mmol, 3 equiv.) in DMSO:H_2O, 600:1 (1.3/2.16 mL/μL) at 40 °C for 18 h. The crude product was then purified *via* column chromatography (10:90 hexane/ethyl acetate) to afford *N*-((*3s,5s,7s*)-adamantan-1-yl)-7-(2,3-dihydroxypropyl)-1,3-dimethyl-2,6-dioxo-2,3,6,7-tetrahydro-1*H*-purine-8-carboxamide 4h as a white solid (32.5 mg, 0.076 mmol, 38%). R_f = 0.33 (10:90 hexane/ethyl acetate). ^{1}H NMR (300 MHz, Chloroform-*d*) δ 7.28 (s, 1H, NH), 5.03 (app. t, *J* = 5.0 Hz, 2H, CH_2), 4.18 – 4.07 (m, 2H, CH_2), 3.63 – 3.56 (m, 5H, OH, CH, CH_3), 3.42 (s, 3H, CH_3), 3.18 (t, *J* = 7.0 Hz, 1H, OH), 2.18 – 2.10 (m, 9H, 3xCH and 3xCH_2), 1.73 (t, *J* = 3.0 Hz, 6H, 3xCH_2).

^{13}C NMR (75 MHz, $CDCl_3$) δ 157.9 (C), 156.6 (C), 151.3 (C), 146.6 (C), 142.6 (C), 110.4 (C), 71.8 (CH), 63.2 (CH_2), 53.4 (C), 48.8 (CH_2), 41.4 (CH_2), 36.3 (CH_2), 30.1 (CH_3), 29.5 (CH), 28.5 (CH_3). ν_{max}/cm^{-1} 3465 (br), 3381, 2909, 2851, 1708, 1665, 1603, 1530, 1466. M.p. = 180 – 183 °C. HRMS (ESI-TOF) m/z: $[M + H]^+$ Calcd for $C_{21}H_{29}N_5O_5H$ 432.2241; Found 432.2259.

_N_-(Adamantan-1-yl)-3,7-dimethyl-2,6-dioxo-2,3,6,7-tetrahydro-1_H_-purine-8-carboxamide (4i)

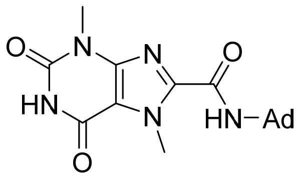

General procedure B was followed: theobromide (35.9 mg, 0.20 mmol, 1 equiv.), 2-((adamantan-1-yl)amino)-2-oxoacetic acid (89.4 mg, 0.40 mmol, 2 equiv.) and $(NH_4)S_2O_8$ (136.5 mg, 0.60 mmol, 3 equiv.) in DMSO:H_2O, 600:1 (1.3/2.16 mL/μL) at 50 °C for 18 h. The crude product was then purified *via* column chromatography (60:40 hexane/ethyl acetate) to afford *N*-((3*s*,5*s*,7*s*)-adamantan-1-yl)-3,7-dimethyl-2,6-dioxo-2,3,6,7-tetrahydro-1*H*-purine-8-carboxamide **4i** as a white solid (51.5 mg, 0.144 mmol, 72%). R_f = 0.23 (60:40 hexane/ethyl acetate). ^1H NMR (300 MHz, Chloroform-*d*) δ 8.03 (s, 1H, NH), 7.14 (s, 1H, NH), 4.35 (s, 3H, CH_3), 3.52 (s, 3H, CH_3), 2.13 (app. s, 9H, 3xCH and 3xCH_2), 1.73 (t, J = 3.0 Hz, 6H, 3xCH_2). ^{13}C NMR (75 MHz, $CDCl_3$) δ 157.1 (C), 154.9 (C), 150.9 (C), 148.0 (C), 142.4 (C), 110.4 (C), 52.9 (C), 41.6 (CH_2), 36.4 (CH_2), 34.9 (CH_3), 29.5 (CH), 29.2 (CH_3). ν_{max}/cm^{-1} 3390, 3169, 3062, 2907, 2850, 1715, 1674, 1597, 1531, 1477. M.p. = 286 °C decomposed. HRMS (ESI-TOF) m/z: $[M + H]^+$ Calcd for $C_{18}H_{23}N_5O_3H$ 358.1874; Found 358.1879.

_N_-(Adamantan-1-yl)-1,7-dimethyl-2,6-dioxo-2,3,6,7-tetrahydro-1_H_-purine-8-carboxamide (4l)

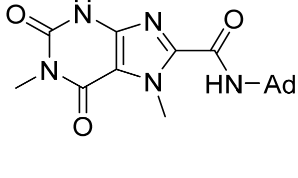

General procedure B was followed: 1,7-dimethylxanthine (36.5 mg, 0.20 mmol, 1 equiv.), 2-((adamantan-1-yl)amino)-2-oxoacetic acid (89.4 mg, 0.40 mmol, 2 equiv.) and $(NH_4)S_2O_8$ (137.0 mg, 0.60 mmol, 3 equiv.) in DMSO:H_2O, 600:1 (1.3/2.16 mL/µL) at 50 °C for 18 h. The crude product was then purified *via* column chromatography (60:40 hexane/ethyl acetate) to afford *N*-((3*s*,5*s*,7*s*)-adamantan-1-yl)-1,7-dimethyl-2,6-dioxo-2,3,6,7-tetrahydro-1*H*-purine-8-carboxamide **4l** as a white solid (2.7 mg, 0.014 mmol, 7%). R_f = 0.34 (60:40 hexane/ethyl acetate). ^{1}H NMR (300 MHz, Chloroform-*d*) δ 8.47 (s, 1H, NH), 7.08 (s, 1H, NH), 4.37 (s, 3H, CH_3), 3.39 (s, 3H, CH_3), 2.16 – 2.08 (m, 9H, 3xCH and 3xCH_2), 1.72 (app. s, 6H, 3xCH_2). ^{13}C NMR (101 MHz, $CDCl_3$) δ 157.1 (C), 156.0 (C), 151.2 (C), 143.9 (C), 142.2 (C), 141.8 (C), 52.9 (C), 41.6 (CH_2), 36.4 (CH_2), 34.7 (CH_3), 29.5 (CH), 27.8 (CH_3). ν_{max}/cm^{-1} 3317, 3206, 2907, 2850, 1726, 1676, 1643, 1534, 1479. M.p. = 183 – 186 °C. HRMS (ESI-TOF) m/z: $[M + H]^+$ Calcd for $C_{18}H_{23}N_5O_3H$ 358.1874; Found 358.1872.

2-Acetamido-*N*-(adamantan-1-yl)-6-oxo-6,9-dihydro-1*H*-purine-8-carboxamide (8a)

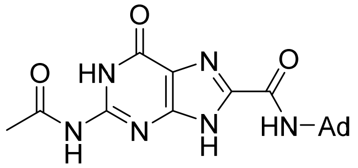

General procedure B was followed: *N*-(6-oxo-6,9-dihydro-1*H*-purin-2-yl)acetamide (38.8 mg, 0.20 mmol, 1 equiv.), 2-((adamantan-1-yl)amino)-2-oxoacetic acid (89.5 mg, 0.40 mmol, 2 equiv.) and $(NH_4)S_2O_8$ (137.1 mg, 0.60 mmol, 3 equiv.) in DMSO:H_2O, 600:1 (1.3/2.16 mL/µL) at 50 °C for 18 h. The crude product was then purified *via* column chromatography (98.5:1.5 DCM/methanol) to afford 2-acetamido-*N*-((3*s*,5*s*,7*s*)-adamantan-1-yl)-6-oxo-6,9-dihydro-1*H*-purine-8-carboxamide **8a** as a white solid (23.1 mg, 0.060 mmol, 31%). R_f = 0.19 (98.5:1.5 DCM/methanol). ^{1}H NMR (300 MHz, Chloroform-*d*) δ 14.40 (s, 1H, NH), 12.49 (s, 1H, NH), 11.74 (s, 1H, NH), 7.51 (s, 1H, NH), 2.50 (s, 3H, CH_3), 2.21 – 2.10 (m, 9H, 3xCH and 3xCH_2), 1.79 – 1.63 (m, 6H, 3xCH and 3xCH_2). ^{13}C NMR (101 MHz, DMSO) δ 173.5 (C), 157.1 (C), 155.0 (C), 148.9 (C), 148.4 (C), 144.6 (C), 69.8 (C), 54.9 (C), 51.6 (CH), 35.9 (CH_2), 28.8 (CH_2), 23.7 (CH_3). ν_{max}/cm^{-1} 3362, 3338, 3158, 3091, 2907, 2850, 1725, 1699, 1662, 1607, 1583, 1543. M.p. = >300 °C. HRMS (ESI-TOF) m/z: $[M + H]^+$ Calcd for $C_{18}H_{22}N_6O_3H$ 371.1826; Found 371.1835.

2-((2-Acetamido-8-(((3*s*,5*s*,7*s*)-adamantan-1-yl)carbamoyl)-6-oxo-1,6-dihydro-9*H*-purin-9-yl)methoxy)ethyl acetate (8b)

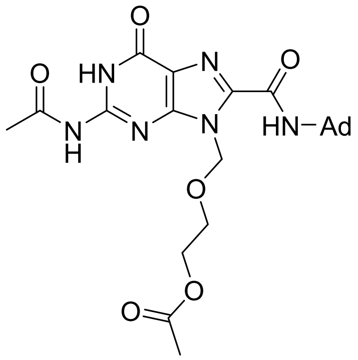

General procedure B was followed: 2-((2-acetamido-6-oxo-1,6-dihydro-9*H*-purin-9-yl)methoxy)ethyl acetate (61.1 mg, 0.20 mmol, 1 equiv.), 2-((adamantan-1-yl)amino)-2-oxoacetic acid (89.4 mg, 0.40 mmol, 2 equiv.) and $(NH_4)S_2O_8$ (137.9 mg, 0.60 mmol, 3 equiv.) in DMSO:H_2O, 600:1 (1.3/2.16 mL/μL) at 50 ºC for 18 h. The crude product was then purified *via* column chromatography (20:80 hexane/ethyl acetate) to afford 2-((2-acetamido-8-(((3*s*,5*s*,7*s*)-adamantan-1-yl)carbamoyl)-6-oxo-1,6-dihydro-9*H*-purin-9-yl)methoxy)ethyl acetate **8b** as a white solid (62.9 mg, 0.130 mmol, 65%). R_f = 0.20 (20:80 hexane/ethyl acetate). ^{1}H NMR (300 MHz, Chloroform-*d*) δ 12.01 (s, 1H, NH), 8.65 (s, 1H, NH), 7.38 (s, 1H, NH), 6.03 (s, 2H, CH_2), 4.28 – 4.20 (m, 2H, CH_2), 3.88 – 3.81 (m, 2H, CH_2), 2.31 (s, 3H, CH_3), 2.10 (app. s, 9H, 3xCH and 3xCH_2), 2.03 (s, 3H, CH_3), 1.71 (app. s, 6H, 3xCH_2). ^{13}C NMR (75 MHz, $CDCl_3$) δ 171.7 (C), 171.6 (C), 157.6 (C), 155.8 (C), 150.7 (C), 148.2 (C), 141.0 (C), 119.8 (C), 73.2 (CH_2), 67.3 (CH_2), 63.5 (CH_2), 53.0 (C), 41.6 (CH_2), 36.4 (CH_2), 29.6 (CH), 24.5 (CH_3), 21.2 (CH_3). ν_{max}/cm^{-1} 3343, 3203, 2907, 2852, 1750, 1699, 1682, 1661, 1616, 1567. M.p. = 256 – 259 ºC. HRMS (ESI-TOF) m/z: $[M + H]^+$ Calcd for $C_{23}H_{30}N_6O_6H$ 487.2300; Found 487.2318.

2-((2-Acetamido-8-((adamantan-1-yl)carbamoyl)-6-oxo-1,6-dihydro-9H-purin-9-yl)methoxy)propane-1,3-diyl diacetate (8c)

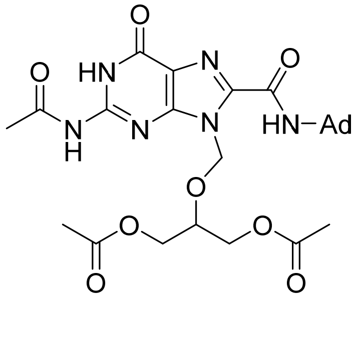

General procedure B was followed: 2-((2-acetamido-6-oxo-1,6-dihydro-9*H*-purin-9-yl)methoxy)propane-1,3-diyl diacetate (76.7 mg, 0.20 mmol, 1 equiv.), 2-((adamantan-1-yl)amino)-2-oxoacetic acid (89.3 mg, 0.40 mmol, 2 equiv.) and $(NH_4)S_2O_8$ (138.0 mg, 0.60 mmol, 3 equiv.) in DMSO:H_2O, 600:1 (1.3/2.16 mL/µL) at 50 ºC for 18 h. The crude product was then purified *via* column chromatography (20:80 hexane/ethyl acetate) to afford 2-((2-acetamido-8-(((3s,5s,7s)-adamantan-1-yl)carbamoyl)-6-oxo-1,6-dihydro-9H-purin-9-yl)methoxy)propane-1,3-diyl diacetate **8c** as a colourless oil (93.4 mg, 0.168 mmol, 84%). R_f = 0.21 (20:80 hexane/ethyl acetate). ^{1}H NMR (300 MHz, Chloroform-*d*) δ 11.99 (s, 1H, NH), 8.84 (s, 1H, NH), 7.37 (s, 1H, NH), 6.10 (s, 2H, CH_2), 4.35 – 4.26 (m, 1H, CH), 4.23 – 4.10 (m, 4H, 2xCH_2), 2.31 (s, 3H, CH_3), 2.10 (app. s, 9H, 3xCH and 3xCH_2), 2.01 (s, 6H, 2xCH_3), 1.71 (app. s, 6H, 3xCH_2). 13C NMR (75 MHz, $CDCl_3$) δ 171.7 (C), 171.1 (2xC), 157.5 (C), 155.8 (C), 150.7 (C), 148.3 (C), 140.7 (C), 119.7 (C), 74.8 (CH), 73.1 (CH_2), 63.6 (2xCH_2), 53.0 (C), 41.6 (CH_2), 36.4 (CH_2), 29.6 (CH), 24.5 (CH_3), 21.0 (2xCH_3). ν_{max}/cm^{-1} 3331, 3150, 2913, 2853, 1744, 1669, 1609, 1557, 1524. HRMS (ESI-TOF) m/z: $[M + H]^+$ Calcd for $C_{26}H_{34}N_6O_8H$ 559.2511; Found 559.2533.

(2*R*,3*R*,4*R*,5*R*)-2-(2-Acetamido-8-((adamantan-1-yl)carbamoyl)-6-oxo-1,6-dihydro-9*H*-purin-9-yl)-5-(acetoxymethyl)tetrahydrofuran-3,4-diyl diacetate (8d)

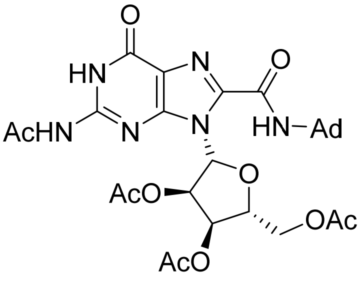

General procedure B was followed: (2*R*,3*R*,4*R*,5*R*)-2-(2-acetamido-6-oxo-1,6-dihydro-9*H*-purin-9-yl)-5-(acetoxymethyl)tetrahydrofuran-3,4-diyl diacetate (90.5 mg, 0.20 mmol, 1 equiv.), 2-((adamantan-1-yl)amino)-2-oxoacetic acid (90.3 mg, 0.40 mmol, 2 equiv.) and $(NH_4)S_2O_8$ (137.1 mg, 0.60 mmol, 3 equiv.) in DMSO:H_2O, 600:1 (1.3/2.16 mL/µL) at 50 ºC for 18 h. The crude product was then purified *via* column chromatography (99:1 DCM/methanol) to afford (2*R*,3*R*,4*R*,5*R*)-2-(2-acetamido-8-(((3*S*,5*S*,7*S*)-adamantan-1-yl)carbamoyl)-6-oxo-1,6-dihydro-9*H*-purin-9-yl)-5-(acetoxymethyl)tetrahydrofuran-3,4-diyl diacetate **8d** as a white solid (70.2 mg, 0.112 mmol, 56%). R_f = 0.21 (99:1 DCM/methanol). ^{1}H NMR (400 MHz, Chloroform-*d*) δ 12.00 (s, 1H, NH), 9.24 (s, 1H, NH), 7.53 (d, *J* = 3.5 Hz,

1H, CH), 7.41 (s, 1H, NH), 6.11 – 6.02 (m, 2H, CH_2), 4.73 (dd, J = 11.5, 5.5 Hz, 1H, CH), 4.43 (app. q, J = 6.0 Hz, 1H, CH), 4.28 (dd, J = 11.5, 6.0 Hz, 1H, CH), 2.33 (s, 3H, CH_3), 2.12 (s, 3H, CH_3), 2.10 (app. s, 12H, CH_3, 3xCH and 3xCH_2), 2.02 (s, 3H, CH_3), 1.70 (app. s, 6H, 3xCH_2). ^{13}C NMR (101 MHz, $CDCl_3$) δ 172.2 (C), 171.7 (C), 170.4 (C), 169.9 (C), 157.5 (C), 155.8 (C), 149.2 (C), 147.8 (C), 140.7 (C), 120.2 (C), 87.9 (CH), 78.9 (CH), 73.3 (CH), 71.1 (CH), 63.1 (CH_2), 53.0 (C), 41.5 (CH_2), 36.4 (CH_2), 29.6 (CH), 24.4 (CH_3), 21.0 (CH_3), 20.8 (CH_3), 20.7 (CH_3). ν_{max}/cm^{-1} 3368, 3168, 2909, 2852, 1750, 1724, 1674, 1608, 1559, 1524. M.p. = 143 – 146 °C. HRMS (ESI-TOF) m/z: $[M + H]^+$ Calcd for $C_{29}H_{36}N_6O_{10}H$ 629.2566; Found 629.2567. $[\alpha]_D^{18.5}$ -68 (c 1.00, $CHCl_3$).

(2*R*,3*R*,4*R*,5*R*)-2-(Acetoxymethyl)-5-(8-((adamantan-1-yl)carbamoyl)-2-isobutyramido-6-oxo-1,6-dihydro-9*H*-purin-9-yl)tetrahydrofuran-3,4-diyl diacetate (8e)

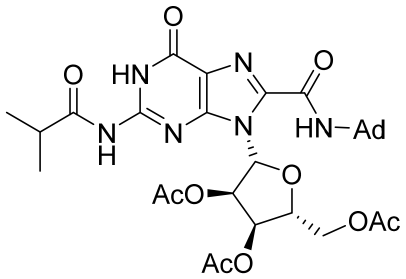

General procedure B was followed: (2*R*,3*R*,4*R*,5*R*)-2-(acetoxymethyl)-5-(2-isobutyramido-6-oxo-1,6-dihydro-9*H*-purin-9-yl)tetrahydrofuran-3,4-diyl diacetate (95.8 mg, 0.20 mmol, 1 equiv.), 2-((adamantan-1-yl)amino)-2-oxoacetic acid (89.7 mg, 0.40 mmol, 2 equiv.) and $(NH_4)S_2O_8$ (136.6 mg, 0.60 mmol, 3 equiv.) in DMSO:H_2O, 600:1 (1.3/2.16 mL/μL) at 50 °C for 18 h. The crude product was then purified *via* column chromatography (99:1 DCM/methanol) to afford (2*R*,3*R*,4*R*,5*R*)-2-(acetoxymethyl)-5-(8-(((3*S*,5*S*,7*S*)-adamantan-1-yl)carbamoyl)-2-isobutyramido-6-oxo-1,6-dihydro-9*H*-purin-9-yl)tetrahydrofuran-3,4-diyl diacetate **8e** as a colourless oil (92.2 mg, 0.140 mmol, 70%). R_f = 0.15 (99:1 DCM/methanol). ^{1}H NMR (400 MHz, Chloroform-*d*) δ 12.09 (s, 1H, NH), 9.00 (s, 1H, NH), 7.49 (d, J = 3.0 Hz, 1H, CH), 7.42 (s, 1H, NH), 6.19 (dd, J = 7.0, 5.5 Hz, 1H, CH), 6.12 (dd, J = 5.5, 3.0 Hz, 1H, CH), 4.62 (dd, J = 12.0, 5.0 Hz, 1H, CH), 4.43 (ddd, J = 7.0, 6.0, 5.0 Hz, 1H, CH), 4.29 (dd, J = 12.0, 6.0 Hz, 1H, CH), 2.71 (sept, J = 7.0 Hz, 1H, CH), 2.12 (s, 3H, CH_3), 2.11 (s, 3H, CH_3), 2.11 (app. s, 9H, 3xCH and 3xCH_2), 1.99 (s, 3H, CH_3), 1.71 (app. s, 6H, 3xCH_2), 1.32 (d, J = 7.0 Hz, 3H, CH_3), 1.30 (d, J = 7.0 Hz, 3H, CH_3). ^{13}C NMR (101 MHz, $CDCl_3$) δ 179.1 (C), 171.4 (C), 170.5 (C), 169.9 (C), 157.5 (C), 155.7 (C), 149.3 (C), 148.1 (C), 140.6 (C), 120.1

(C), 87.9 (CH), 78.5 (CH), 73.3 (CH), 70.9 (CH), 62.9 (CH_2), 53.0 (C), 42.8 (CH), 41.5 (CH_2), 36.4 (CH_2), 29.6 (CH), 20.9 (CH_3), 20.8 (CH_3), 20.7 (CH_3), 19.0 (CH_3). ν_{max}/cm^{-1} 3169, 2907, 2852, 1746, 1669, 1605, 1559, 1522, 1425. HRMS (ESI-TOF) m/z: $[M + H]^+$ Calcd for $C_{31}H_{40}N_6O_{10}H$ 657.2879; Found 657.2877. $[\alpha]_D^{21.6}$ -52 (c 1.00, $CHCl_3$).

***N*²,*N*⁸-Di(adamantan-1-yl)-6-benzamido-9*H*-purine-2,8-dicarboxamide (10a)**

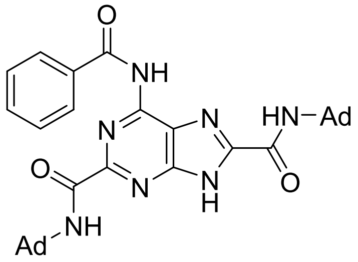

General procedure B was followed: *N*-(9*H*-purin-6-yl)benzamide (47.7 mg, 0.20 mmol, 1 equiv.), 2-((adamantan-1-yl)amino)-2-oxoacetic acid (89.5 mg, 0.40 mmol, 2 equiv.) and $(NH_4)S_2O_8$ (137.6 mg, 0.60 mmol, 3 equiv.) in DMSO:H_2O, 600:1 (1.3/2.16 mL/µL) at 50 ºC for 18 h. The crude product was then purified *via* column chromatography (70:30 DCM/ethyl acetate) to afford N^2,N^8-di((3*s*,5*s*,7*s*)-adamantan-1-yl)-6-benzamido-9*H*-purine-2,8-dicarboxamide **10a** as an off-white solid (49.8 mg, 0.084 mmol, 42%). R_f = 0.28 (70:30 DCM/ethyl acetate). ^{1}H NMR (400 MHz, Chloroform-*d*) δ 8.81 (s, 1H, NH), 8.02 – 7.98 (m, 2H, Ar-H), 7.68 – 7.65 (m, 1H, Ar-H), 7.60 – 7.54 (m, 2H, Ar-H), 7.41 (s, 1H, NH), 2.24 – 2.11 (m, 18H, 6xCH and 6xCH_2), 1.75 (app. s, 12H, 6xCH_2). ^{13}C NMR (101 MHz, $CDCl_3$) δ 166.0 (C), 164.2 (C), 164.0 (C), 156.4 (C), 153.2 (C), 150.3 (C), 144.4 (C), 140.6 (C), 133.8 (CH), 129.4 (CH), 127.9 (CH), 70.7 (C), 53.1 (2xC overlapping), 41.5 (CH_2), 41.2 (CH_2), 36.4 (2xCH_2 overlapping), 29.6 (2xCH overlapping). ν_{max}/cm^{-1} 3368, 2907, 2850, 1669, 1611, 1527, 1491, 1456, 1420. M.p. = 190 ºC decomposed. HRMS (ESI-TOF) m/z: $[M + H]^+$ Calcd for $C_{34}H_{39}N_7O_3H$ 594.3187; Found 594.3173.

(2*R*,3*R*,4*R*,5*R*)-2-(6-Acetamido-2,8-bis((adamantan-1-yl)carbamoyl)-9*H*-purin-9-yl)-5-(acetoxymethyl)tetrahydrofuran-3,4-diyl diacetate (10b)

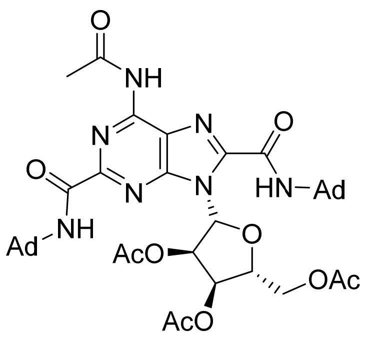

General procedure B was followed: (2*R*,3*R*,4*R*,5*R*)-2-(6-acetamido-9*H*-purin-9-yl)-5-(acetoxymethyl)tetrahydrofuran-3,4-diyl diacetate (87.9 mg, 0.20 mmol, 1 equiv.), 2-((adamantan-1-yl)amino)-2-oxoacetic acid (89.3 mg, 0.40 mmol, 2 equiv.) and $(NH_4)S_2O_8$ (137.2 mg, 0.60 mmol, 3 equiv.) in DMSO:H_2O, 600:1 (1.3/2.16 mL/μL) at 50 °C for 18 h. The crude product was then purified *via* column chromatography (98:2 DCM/methanol) to afford (2*R*,3*R*,4*R*,5*R*)-2-(6-acetamido-2,8-bis(((3*S*,5*S*,7*S*)-adamantan-1-yl)carbamoyl)-9*H*-purin-9-yl)-5-(acetoxymethyl)tetrahydrofuran-3,4-diyl diacetate **10b** as a colourless oil (61.8 mg, 0.078 mmol, 39%). R_f = 0.47 (96:4 DCM/methanol). ^{1}H NMR (300 MHz, Chloroform-*d*) δ 8.42 (s, 1H, NH), 7.63 (s, 1H, NH), 7.39 (d, *J* = 4.0 Hz, 1H, CH), 7.35 (s, 1H, NH), 6.31 (dd, *J* = 6.5, 4.0 Hz, 1H, CH), 5.82 (t, *J* = 6.5 Hz, 1H, CH), 4.65 – 4.57 (m, 1H, CH), 4.44 – 4.33 (m, 2H, CH_2), 2.76 (s, 3H, CH_3), 2.17 – 2.13 (m, 21H, CH_3, 6xCH and 6xCH_2), 2.07 (s, 3H, CH_3), 2.01 (s, 3H, CH_3), 1.75 (d, *J* = 3.3 Hz, 12H, 6xCH_2). ^{13}C NMR (75 MHz, $CDCl_3$) δ 170.7 (C), 170.5 (C), 169.8 (C), 169.7 (C), 160.3 (C), 156.6 (C), 153.9 (C), 152.7 (C), 149.6 (C), 145.6 (C), 120.2 (C), 88.5 (CH), 79.9 (CH), 72.9 (CH), 70.8 (CH), 63.2 (CH_2), 53.2 (C), 52.3 (C), 41.5 (2xCH_2 overlapping), 36.5 (CH_2), 36.4 (CH_2), 29.6 (CH), 29.5 (CH), 26.1 (CH_3), 20.9 (CH_3), 20.7 (CH_3), 20.6 (CH_3). ν_{max}/cm^{-1} 3331, 2908, 2852, 1749, 1658, 1608, 1586, 1522, 1454. HRMS (ESI-TOF) m/z: $[M + H]^+$ Calcd for $C_{40}H_{51}N_7O_{10}H$ 790.3770; Found 790.3787. $[\alpha]_D^{19.1}$ +8 (c 1.00, $CHCl_3$).

(2*R*,3*R*,4*R*,5*R*)-2-(6-Acetamido-8-((adamantan-1-yl)carbamoyl)-2-chloro-9*H*-purin-9-yl)-5-(acetoxymethyl)tetrahydrofuran-3,4-diyl diacetate (10c)

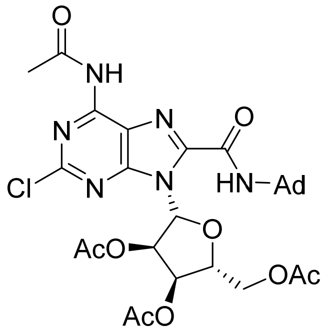

General procedure B was followed: (2*R*,3*R*,4*R*,5*R*)-2-(6-acetamido-2-chloro-9*H*-purin-9-yl)-5-(acetoxymethyl)tetrahydrofuran-3,4-diyl diacetate (90.0 mg, 0.20 mmol, 1 equiv.), 2-((adamantan-1-yl)amino)-2-oxoacetic acid (89.7 mg, 0.40 mmol, 2 equiv.) and $(NH_4)S_2O_8$ (137.1 mg, 0.60 mmol, 3 equiv.) in DMSO:H_2O, 600:1 (1.3/2.16 mL/μL) at 50 °C for 18 h. The crude product was then purified *via* column chromatography (95:5 DCM/ethyl acetate) to afford (2*R*,3*R*,4*R*,5*R*)-2-(6-acetamido-8-(((3*S*,5*S*,7*S*)-adamantan-1-yl)carbamoyl)-2-chloro-9*H*-purin-9-yl)-5-(acetoxymethyl)tetrahydrofuran-3,4-diyl diacetate **10c** as a colourless oil (66.5 mg, 0.106 mmol, 53%). R_f = 0.16 (95:5 DCM/ethyl acetate). ^{1}H NMR (400 MHz, Chloroform-*d*) δ 8.30 (s, 1H, NH), 7.39 (d, *J* = 4.0 Hz, 1H, CH), 7.28 (s, 1H, NH), 6.10 (dd, *J* = 6.5, 4.0 Hz, 1H, CH), 5.86 (t, *J* = 6.5 Hz, 1H, CH), 4.58 – 4.49 (m, 1H, CH), 4.39 – 4.31 (m, 2H, CH_2), 2.73 (s, 3H, CH_3), 2.18 – 2.11 (m, 12H, CH_3, 3xCH and 3xCH_2), 2.09 (s, 3H, CH_3), 2.07 (s, 3H, CH_3), 1.73 (t, *J* = 3.0 Hz, 6H, 3xCH_2). ^{13}C NMR (101 MHz, $CDCl_3$) δ 171.0 (C), 170.8 (C), 169.8 (C), 169.7 (C), 156.7 (C), 154.9 (C), 153.2 (C), 150.6 (C), 144.2 (C), 118.5 (C), 88.5 (CH), 79.9 (CH), 73.1 (CH), 70.3 (CH), 63.0 (CH_2), 53.2 (C), 41.5 (CH_2), 36.4 (CH_2), 29.5 (CH), 26.1 (CH_3), 20.9 (CH_3), 20.7 (2xCH_3, overlapping). ν_{max}/cm^{-1} 3284, 2910, 2851, 2255, 1746, 1683, 1607, 1584, 1530. HRMS (ESI-TOF) m/z: $[M + H]^+$ Calcd for $C_{29}H_{35}N_6O_9{}^{35}ClH$ 647.2227; Found 647.2232. $[\alpha]_D^{22.4}$ +20 (c 1.00, $CHCl_3$).

(2*R*,3*R*,4*R*,5*R*)-2-(Acetoxymethyl)-5-(8-((adamantan-1-yl)carbamoyl)-6-(bis(tert-butoxycarbonyl)amino)-2-fluoro-9*H*-purin-9-yl)tetrahydrofuran-3,4-diyl diacetate (10d)

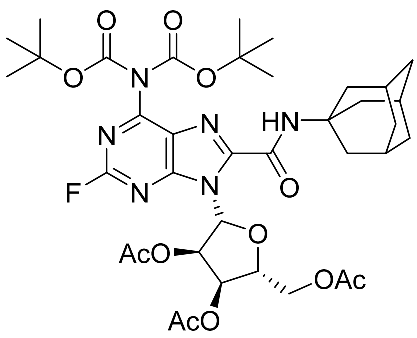

General procedure B was followed: (2*R*,3*R*,4*R*,5*R*)-2-(acetoxymethyl)-5-(6-(bis(tert-butoxycarbonyl)amino)-2-fluoro-9*H*-purin-9-yl)tetrahydrofuran-3,4-diyl diacetate (156.2 mg, 0.25 mmol, 1 equiv.), 2-((adamantan-1-yl)amino)-2-oxoacetic acid (111.2 mg, 0.50 mmol, 2 equiv.) and $(NH_4)S_2O_8$ (172.4 mg, 0.75 mmol, 3 equiv.) in DMSO:H_2O, 600:1 (1.3/2.16 mL/µL) at 50 °C for 18 h. The crude product was then purified *via* column chromatography (70:30 hexane/ethyl acetate) to afford (2*R*,3*R*,4*R*,5*R*)-2-(acetoxymethyl)-5-(8-(((3*S*,5*S*,7*S*)-adamantan-1-yl)carbamoyl)-6-(bis(tert-butoxycarbonyl)amino)-2-fluoro-9*H*-purin-9-yl)tetrahydrofuran-3,4-diyl diacetate **10d** as a colourless oil (119.7 mg, 0.153 mmol, 61%). R_f = 0.21 (70:30 hexane/ethyl acetate). ^{1}H NMR (400 MHz, Chloroform-*d*) δ 7.82 (d, *J* = 7.0 Hz, 1H, CH), 7.30 (s, 1H, NH), 5.96 (dd, *J* = 7.5, 6.0 Hz, 1H, CH), 5.74 (dd, *J* = 7.0, 6.0 Hz, 1H, CH), 4.68 (dd, *J* = 12.0, 3.5 Hz, 1H, C*H*H), 4.59 (dd, *J* = 12.0, 7.5 Hz, 1H, CH*H*), 4.24 (td, *J* = 7.5, 3.5 Hz, 1H, CH), 2.15 – 2.12 (m, 6H, CH_3 and 3xCH), 2.10 (s, 3H, CH_3), 2.08 (d, *J* = 3.0 Hz, 6H, 3xCH_2), 1.72 (t, *J* = 3.0 Hz, 6H, 3xCH_2), 1.60 (s, 3H, CH_3), 1.48 (s, 18H, 6xCH_3). ^{13}C NMR (101 MHz, $CDCl_3$) δ 170.8 (C), 170.0 (C), 169.1 (C), 158.2 (d, *J* = 218.6 Hz, CF), 156.6 (C), 156.1 (d, *J* = 17.0 Hz, C), 153.6 (d, *J* = 17.0 Hz, C), 149.8 (C), 145.7 (d, *J* = 3.0 Hz, C), 124.4 (d, *J* = 5.0 Hz, C), 84.7 (C), 84.2 (CH), 78.7 (CH), 75.8 (CH), 75.3 (CH), 63.5 (CH_2), 53.2 (C), 41.5 (CH_2), 36.3 (CH_2), 29.5 (CH), 27.9 (CH_3), 21.0 (CH_3), 20.9 (CH_3), 20.1 (CH_3). ^{19}F NMR (376 MHz, Chloroform-*d*) δ -47.12 (s, CF). ν_{max}/cm^{-1} 3387, 2981, 2910, 2853, 2258, 1792, 1746, 1687, 1606, 1527, 1486, 1454. HRMS (ESI-TOF) m/z: [M + H]$^+$ Calcd for $C_{37}H_{49}N_6O_{12}H$ 789.3465; Found 789.3465. $[\alpha]_D^{23.0}$ -20 (c 1.00, $CHCl_3$).

(2*R*,3*R*,4*R*,5*R*)-2-(6-Acetamido-2-((adamantan-1-yl)carbamoyl)-8-bromo-9*H*-purin-9-yl)-5-(acetoxymethyl)tetrahydrofuran-3,4-diyl diacetate (10e)

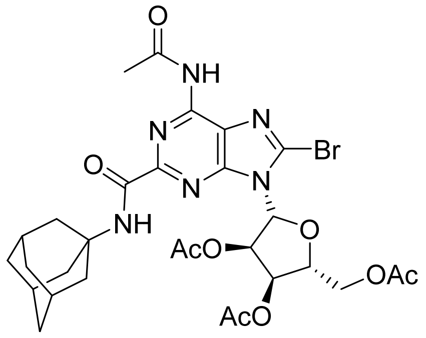

General procedure B was followed: (2*R*,3*R*,4*R*,5*R*)-2-(6-acetamido-8-bromo-9*H*-purin-9-yl)-5-(acetoxymethyl)tetrahydrofuran-3,4-diyl diacetate (95.6 mg, 0.19 mmol, 1 equiv.), 2-((adamantan-1-yl)amino)-2-oxoacetic acid (83.0 mg, 0.38 mmol, 2 equiv.) and $(NH_4)S_2O_8$ (126.6 mg, 0.56 mmol, 3 equiv.) in DMSO:H_2O, 600:1 (1.3/2.16 mL/µL) at 70 °C for 18 h. The crude product was then purified *via* column chromatography (50:50 DCM/ethyl acetate) to afford (2*R*,3*R*,4*R*,5*R*)-2-(6-acetamido-2-(((1*s*,3*S*)-adamantan-1-yl)carbamoyl)-8-bromo-9*H*-purin-9-yl)-5-(acetoxymethyl)tetrahydrofuran-3,4-diyl diacetate **10e** as a colourless oil (43.7 mg, 0.076 mmol, 40%). R_f = 0.37 (50:50 DCM/ethyl acetate). ^{1}H NMR (400 MHz, Chloroform-*d*) δ 7.38 (s, 1H, NH), 6.24 (dd, *J* = 6.0, 4.5 Hz, 1H, CH), 6.15 (d, *J* = 4.5 Hz, 1H, NH), 5.67 (t, *J* = 6.0 Hz, 1H, CH), 4.49 (dd, *J* = 12.0, 3.5 Hz, 1H, C*H*H), 4.34 (td, *J* = 6.0, 3.5 Hz, 2H, 2xCH), 4.25 (dd, *J* = 12.0, 6.0 Hz, 1H, CH*H*), 2.27 (s, 3H, CH_3), 2.15 – 2.12 (m, 12H, CH_3, 3xCH and 3xCH_2), 2.10 (s, 3H, CH_3), 2.06 (s, 3H, CH_3), 1.73 (app. s, 6H, 3xCH_2). ^{13}C NMR (101 MHz, $CDCl_3$) δ 170.7 (C), 170.0 (C), 169.7 (C), 169.6 (C), 160.4 (C), 151.1 (C), 151.0 (C), 150.6 (C), 137.9 (C), 109.8 (C), 84.4 (CH), 79.4 (CH), 71.3 (CH), 70.7 (CH), 63.2 (CH_2), 52.5 (C), 41.4 (CH_2), 36.5 (CH_2), 29.6 (CH), 24.2 (CH_3), 20.9 (CH_3), 20.7 (CH_3), 20.6 (CH_3). ν_{max}/cm^{-1} 3315, 2909, 2853, 1744, 1704, 1631, 1593, 1518, 1479, 1371. HRMS (ESI-TOF) m/z: $[M + H]^+$ Calcd for $C_{30}H_{40}N_6O_{10}{}^{79}BrH$ 733.5213; Found 733.5209. $[\alpha]_D^{22.7}$ +12 (c 1.00, $CHCl_3$).

***N*-(*tert*-Butyl)-1,3,7-trimethyl-2,6-dioxo-2,3,6,7-tetrahydro-1*H*-purine-8-carboxamide (4m)**

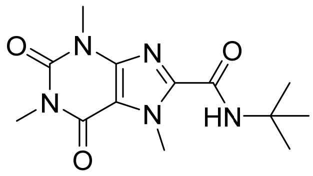

General procedure B was followed: caffeine (39.2 mg, 0.20 mmol, 1 equiv.), 2-(tert-butylamino)-2-oxoacetic acid (60.6 mg, 0.40 mmol, 2 equiv.) and $(NH_4)S_2O_8$ (137.6 mg, 0.60 mmol, 3 equiv.) in DMSO:H_2O, 600:1 (1.3/2.16 mL/µL) at 50 °C for 18 h. The crude product was then purified *via* column chromatography (70:30 hexane/ethyl acetate) to afford *N*-(tert-butyl)-1,3,7-trimethyl-2,6-dioxo-2,3,6,7-tetrahydro-1*H*-purine-8-carboxamide **4m** as a white solid (39.8 mg, 0.136 mmol, 68%). R_f = 0.16 (70:30 hexane/ethyl acetate). ^{1}H NMR (300 MHz, Chloroform-*d*) δ 4.39 (s, 3H, CH_3), 3.56 (s, 3H, CH_3), 3.40 (s, 3H, CH_3), 1.48 (s, 9H, 3xCH_3). ^{13}C NMR (75 MHz, $CDCl_3$) δ 157.5 (C), 155.7 (C), 151.7 (C), 146.1 (C), 141.8 (C), 110.2 (C), 52.0 (C), 34.8 (CH_3), 29.9 (CH_3), 28.8 (CH_3), 28.2 (CH_3). ν_{max}/cm^{-1} 3392, 2974, 2874, 1712, 1678, 1656, 1600, 1522. M.p. = 174 °C decomposed. HRMS (ESI-TOF) m/z: [M + H]$^+$ Calcd for $C_{13}H_{19}N_5O_3H$ 294.1561; Found 294.1574.

***N*-Butyl-1,3,7-trimethyl-2,6-dioxo-2,3,6,7-tetrahydro-1*H*-purine-8-carboxamide (4n)**

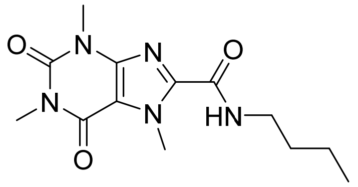

General procedure B was followed: caffeine (39.2 mg, 0.20 mmol, 1 equiv.), 2-(butylamino)-2-oxoacetic acid (59.1 mg, 0.40 mmol, 2 equiv.) and $(NH_4)S_2O_8$ (137.7 mg, 0.60 mmol, 3 equiv.) in DMSO:H_2O, 600:1 (1.3/2.16 mL/µL) at 50 °C for 18 h. The crude product was then purified *via* column chromatography (60:40 hexane/ethyl acetate) to afford *N*-butyl-1,3,7-trimethyl-2,6-dioxo-2,3,6,7-tetrahydro-1*H*-purine-8-carboxamide **4n** as a white solid (27.5 mg, 0.094 mmol, 47%). R_F = 0.16 (60:40 hexane/ethyl acetate). ^{1}H NMR (300 MHz, Chloroform-*d*) δ 7.38 (s, 1H, NH), 4.40 (s, 3H, CH_3), 3.55 (s, 3H, CH_3), 3.46 – 3.38 (m, 5H, CH_2 and CH_3), 1.65 – 1.56 (m, 2H, CH_2), 1.49 – 1.35 (m, 2H, CH_2), 0.96 (t, *J* = 7.5 Hz, 3H, CH_3). ^{13}C NMR (75 MHz, $CDCl_3$) δ 158.0 (C), 155.6 (C), 151.5 (C), 146.2 (C), 141.2 (C), 110.1 (C), 39.1 (CH_2), 34.5 (CH_3), 31.6 (CH_2), 29.7 (CH_3), 28.1 (CH_3), 20.1 (CH_2), 13.7 (CH_3). ν_{max}/cm^{-1} 3363,

2953, 2873, 1706, 1652, 1602, 1525, 1476. M.p. = 115 – 118 ºC. HRMS (ESI-TOF) m/z: [M + H]+ Calcd for $C_{13}H_{19}N_5O_3H$ 294.1561; Found 294.1572.

***N*-Cyclopentyl-1,3,7-trimethyl-2,6-dioxo-2,3,6,7-tetrahydro-1*H*-purine-8-carboxamide (4o)**

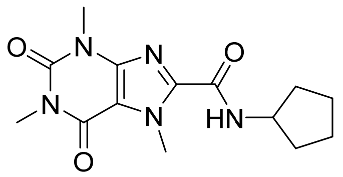

General procedure B was followed: caffeine (39.5 mg, 0.20 mmol, 1 equiv.), 2-(cyclopentylamino)-2-oxoacetic acid (63.5 mg, 0.40 mmol, 2 equiv.) and $(NH_4)S_2O_8$ (142.6 mg, 0.60 mmol, 3 equiv.) in DMSO:H_2O, 600:1 (1.3/2.16 mL/µL) at 50 ºC for 18 h. The crude product was then purified *via* column chromatography (70:30 hexane/ethyl acetate) to afford *N*-cyclopentyl-1,3,7-trimethyl-2,6-dioxo-2,3,6,7-tetrahydro-1*H*-purine-8-carboxamide **4o** as a white solid (33.6 mg, 0.110 mmol, 55%). R_f = 0.18 (70:30 hexane/ethyl acetate). ^{1}H NMR (400 MHz, Chloroform-*d*) δ 7.30 (d, *J* = 8.0 Hz, 1H, NH), 4.40 (s, 3H, CH_3), 4.38 – 4.28 (m, 1H, CH), 3.57 (s, 3H, CH_3), 3.41 (s, 3H, CH_3), 2.14 – 2.04 (m, 2H, CH_2), 1.81 – 1.64 (m, 4H, 2xCH_2), 1.61 – 1.50 (m, 2H, CH_2). ^{13}C NMR (101 MHz, $CDCl_3$) δ 157.8 (C), 155.7 (C), 151.7 (C), 146.3 (C), 141.3 (C), 110.2 (C), 51.3 (CH), 34.7 (CH_3), 33.2 (CH_2), 30.0 (CH_3), 28.3 (CH_3), 24.0 (CH_2). ν_{max}/cm^{-1} 3409, 2946, 1705, 1674, 1655, 1597, 1521, 1468. M.p. = 185 – 188 ºC. HRMS (ESI-TOF) m/z: [M + H]+ Calcd for $C_{14}H_{19}N_5O_3H$ 306.1561; Found 306.1571.

***N*-Cyclohexyl-1,3,7-trimethyl-2,6-dioxo-2,3,6,7-tetrahydro-1*H*-purine-8-carboxamide (4p)**

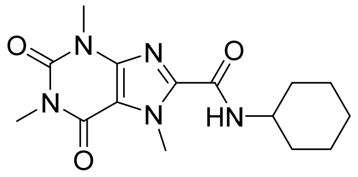

General procedure B was followed: caffeine (38.9 mg, 0.20 mmol, 1 equiv.), 2-(cyclohexylamino)-2-oxoacetic acid (68.0 mg, 0.40 mmol, 2 equiv.) and $(NH_4)S_2O_8$ (142.5 mg, 0.60 mmol, 3 equiv.) in DMSO:H_2O, 600:1 (1.3/2.16 mL/µL) at 50 ºC for 18 h. The crude product was then purified *via* column chromatography (70:30 hexane/ethyl acetate) to afford

N-cyclohexyl-1,3,7-trimethyl-2,6-dioxo-2,3,6,7-tetrahydro-1*H*-purine-8-carboxamide **4p** as a white solid (64.0 mg, 0.200 mmol, quant.).

1.169 g Scale:

Caffeine (1.1691 g, 6.00 mmol, 1 equiv.), 2-(cyclohexylamino)-2-oxoacetic acid (2.0754 g, 12.00 mmol, 2 equiv.) and $(NH_4)S_2O_8$ (4.1014 g, 18.00 mmol, 3 equiv.) were added to an oven-dried, Ar backfilled 100 mL round bottom flask. Separately, in another 100 mL round bottom flask, a mixture of DMSO (40 mL) and H_2O (60 µL) was degassed *via* sparging with Ar for approximately 2 h. The DMSO:H_2O, 600:1 (40/60 mL/µL) mixture was then added to the round bottom flask containing the reaction components and the resulting mixture stirred at 50 ºC for 18 h. The crude product was then purified *via* column chromatography (70:30 hexane/ethyl acetate) to afford *N*-cyclohexyl-1,3,7-trimethyl-2,6-dioxo-2,3,6,7-tetrahydro-1*H*-purine-8-carboxamide **4p** as a white solid (1.8203 g, 5.70 mmol, 95%).

R_f = 0.25 (70:30 hexane/ethyl acetate). ^{1}H NMR (400 MHz, Chloroform-*d*) δ 4.40 (s, 3H, CH_3), 3.95 – 3.84 (m, 1H, CH), 3.57 (s, 3H, CH_3), 3.41 (s, 3H, CH_3), 2.04 – 1.96 (m, 2H, CH_2), 1.78 (dt, J = 13.0, 4.0 Hz, 2H, CH_2), 1.67 (dt, J = 13.0, 4.0 Hz, 1H, C*H*H), 1.49 – 1.17 (m, 5H, CH*H* and 2xCH_2). ^{13}C NMR (101 MHz, $CDCl_3$) δ 157.3 (C), 155.7 (C), 151.7 (C), 146.3 (C), 141.4 (C), 110.2 (C), 48.6 (CH), 34.7 (CH_3), 33.1 (CH_2), 30.0 (CH_3), 28.3 (CH_3), 25.6 (CH_2), 25.0 (CH_2). ν_{max}/cm^{-1} 3281, 2936, 2847, 1708, 1662, 1601, 1531, 1476. M.p. = 200 – 203 ºC. HRMS (ESI-TOF) m/z: [M + H]$^+$ Calcd for $C_{15}H_{21}N_5O_3H$ 320.1717; Found 320.1724.

***N*-Cycloheptyl-1,3,7-trimethyl-2,6-dioxo-2,3,6,7-tetrahydro-1*H*-purine-8-carboxamide (4q)**

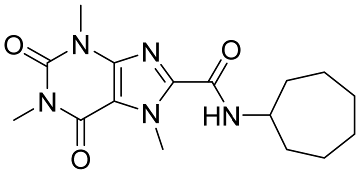

General procedure B was followed: caffeine (39.6 mg, 0.20 mmol, 1 equiv.), 2-(cycloheptylamino)-2-oxoacetic acid (73.6 mg, 0.40 mmol, 2 equiv.) and $(NH_4)S_2O_8$ (142.7 mg, 0.60 mmol, 3 equiv.) in DMSO:H_2O, 600:1 (1.3/2.16 mL/µL) at 50 ºC for 18 h. The crude product was then purified *via* column chromatography (70:30 hexane/ethyl acetate) to afford *N*-cycloheptyl-1,3,7-trimethyl-2,6-dioxo-2,3,6,7-tetrahydro-1*H*-purine-8-carboxamide **4q** as a white solid (48.7 mg, 0.146 mmol, 73%). R_f = 0.26 (70:30 hexane/ethyl acetate). ^{1}H NMR (400

MHz, Chloroform-*d*) δ 7.32 (d, *J* = 8.5 Hz, 1H, NH), 4.40 (s, 3H, CH_3), 4.12 – 4.02 (m, 1H, CH), 3.57 (s, 3H, CH_3), 3.41 (s, 3H, CH_3), 2.08 – 1.96 (m, 2H, CH_2), 1.75 – 1.52 (m, 10H, 5xCH_2). ^{13}C NMR (101 MHz, $CDCl_3$) δ 157.1 (C), 155.7 (C), 151.7 (C), 146.3 (C), 141.5 (C), 110.2 (C), 50.9 (CH), 35.1 (CH_2), 34.7 (CH_3), 30.0 (CH_3), 28.3 (CH_3), 28.1 (CH_2), 24.3 (CH_2). ν_{max}/cm^{-1} 3400, 2923, 2856, 1711, 1671, 1602, 1525, 1471. M.p. = 189 – 192 °C. HRMS (ESI-TOF) m/z: $[M + H]^+$ Calcd for $C_{16}H_{23}N_5O_3H$ 334.1874; Found 334.1881.

N-(4-Methoxyphenyl)-1,3,7-trimethyl-2,6-dioxo-2,3,6,7-tetrahydro-1*H*-purine-8-carboxamide (4r)

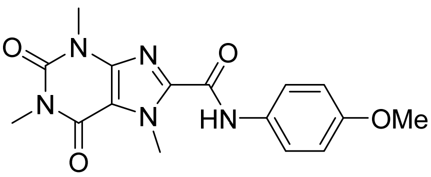

General procedure B was followed: caffeine (38.9 mg, 0.20 mmol, 1 equiv.), 2-((4-methoxyphenyl)amino)-2-oxoacetic acid (77.8 mg, 0.40 mmol, 2 equiv.) and $(NH_4)S_2O_8$ (136.9 mg, 0.60 mmol, 3 equiv.) in DMSO:H_2O, 600:1 (1.3/2.16 mL/µL) at 50 °C for 18 h. The crude product was then purified *via* column chromatography (99:1, DCM:methanol) to afford *N*-(4-methoxyphenyl)-1,3,7-trimethyl-2,6-dioxo-2,3,6,7-tetrahydro-1*H*-purine-8-carboxamide **4r** as a brown solid (38.1 mg, 0.112 mmol, 56%). R_f = 0.28 (99:1 DCM/methanol). ^{1}H NMR (300 MHz, Chloroform-*d*) δ 9.06 (s, 1H, NH), 7.59 (d, *J* = 9.0 Hz, 2H, Ar-H), 6.92 (d, *J* = 9.0 Hz, 2H, Ar-H), 4.46 (s, 3H, CH_3), 3.82 (s, 3H, CH_3), 3.61 (s, 3H, CH_3), 3.43 (s, 3H, CH_3). ^{13}C NMR (75 MHz, $CDCl_3$) δ 157.2 (C), 155.7 (C), 151.6 (C), 146.3 (C), 141.2 (C), 130.1 (C), 121.8 (CH), 114.7 (CH), 114.5 (CH), 110.7 (C), 55.7 (C), 34.9 (CH_3), 30.0 (CH_3), 28.3 (CH_3). ν_{max}/cm^{-1} 3354, 2970, 2261, 1738, 1708, 1677, 1658, 1595, 1550, 1510. M.p. = 218 – 221 °C. HRMS (ESI-TOF) m/z: $[M + H]^+$ Calcd for $C_{16}H_{17}N_5O_4H$ 344.1353; Found 344.1354.

***N*-(4-(*tert*-Butyl)phenyl)-1,3,7-trimethyl-2,6-dioxo-2,3,6,7-tetrahydro-1*H*-purine-8-carboxamide (4s)**

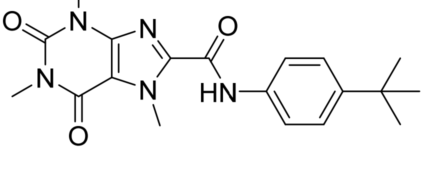

General procedure B was followed: caffeine (39.1 mg, 0.20 mmol, 1 equiv.), 2-((4-(tert-butyl)phenyl)amino)-2-oxoacetic acid (88.9 mg, 0.40 mmol, 2 equiv.) and $(NH_4)S_2O_8$ (142.8 mg, 0.60 mmol, 3 equiv.) in DMSO:H_2O, 600:1 (1.3/2.16 mL/μL) at 50 °C for 18 h. The crude product was then purified *via* column chromatography (85:15, hexane:ethyl acetate) to afford *N*-(4-(tert-butyl)phenyl)-1,3,7-trimethyl-2,6-dioxo-2,3,6,7-tetrahydro-1*H*-purine-8-carboxamide **4s** as a brown solid (38.5 mg, 0.104 mmol, 52%). R_f = 0.08 (85:15 hexane/ethyl acetate). ^{1}H NMR (300 MHz, Chloroform-*d*) δ 9.10 (s, 1H, NH), 7.59 (d, *J* = 9.0 Hz, 2H, Ar-H), 7.41 (d, *J* = 9.0 Hz, 2H, Ar-H), 4.47 (s, 3H, CH_3), 3.62 (s, 3H, CH_3), 3.43 (s, 3H, CH_3), 1.33 (s, 9H, 3xCH_3). ^{13}C NMR (75 MHz, $CDCl_3$) δ 155.8 (C), 155.7 (C), 151.6 (C), 148.4 (C), 146.2 (C), 141.1 (C), 134.3 (C), 126.2 (CH), 119.9 (CH), 110.7 (C), 34.9 (CH_3), 34.7 (C), 31.5 (CH_3), 30.0 (CH_3), 28.3 (CH_3). ν_{max}/cm^{-1} 3364, 2960, 1710, 1681, 1662, 1603, 1590, 1527. M.p. = 258 – 261 °C. HRMS (ESI-TOF) m/z: [M + H]$^+$ Calcd for $C_{19}H_{23}N_5O_3H$ 370.1874; Found 370.1866.

1,3,7-Trimethyl-2,6-dioxo-*N*-phenyl-2,3,6,7-tetrahydro-1*H*-purine-8-carboxamide (4t)

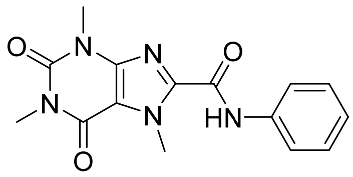

General procedure B was followed: caffeine (39.0 mg, 0.20 mmol, 1 equiv.), 2-oxo-2-(phenylamino)acetic acid (67.1 mg, 0.40 mmol, 2 equiv.) and $(NH_4)S_2O_8$ (137.2 mg, 0.60 mmol, 3 equiv.) in DMSO:H_2O, 600:1 (1.3/2.16 mL/μL) at 50 °C for 18 h. The crude product was then purified *via* column chromatography (70:30, hexane:ethyl acetate) to afford 1,3,7-trimethyl-2,6-dioxo-*N*-phenyl-2,3,6,7-tetrahydro-1*H*-purine-8-carboxamide **4t** as a white solid (13.6 mg, 0.044 mmol, 22%). R_f = 0.12 (70:30 hexane/ethyl acetate). ^{1}H NMR (300 MHz, Chloroform-*d*) δ 9.15 (s, 1H, NH), 7.68 (dd, *J* = 7.5, 1.0 Hz, 2H, 2xAr-H), 7.40 (t, *J* = 7.5 Hz, 2H, 2xAr-H), 7.20 (tt, *J* = 2.0, 1.0 Hz, 1H, Ar-H), 4.48 (s, 3H, CH_3), 3.63 (s, 3H, CH_3), 3.44 (s, 3H, CH_3). ^{13}C NMR (75 MHz, $CDCl_3$) δ 155.9 (C), 155.7 (C), 151.8 (C), 151.6 (C), 141.0 (C), 137.0 (C), 129.4 (CH), 125.3 (CH), 120.1 (CH), 118.6 (C), 34.9 (CH_3), 30.0 (CH_3), 28.3 (CH_3). ν_{max}/cm^{-1} 3380, 2955, 1709, 1677, 1658, 1602, 1535, 1443. M.p. = 232 – 235 °C. HRMS (ESI-TOF) m/z: [M + H]$^+$ Calcd for $C_{15}H_{15}N_5O_3H$ 314.1248; Found 314.1254.

N-Benzyl-1,3,7-trimethyl-2,6-dioxo-2,3,6,7-tetrahydro-1H-purine-8-carboxamide (4v)

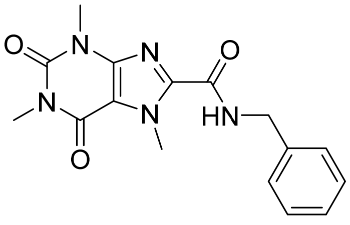

General procedure B was followed: caffeine (38.6 mg, 0.20 mmol, 1 equiv.), 2-(benzylamino)-2-oxoacetic acid (71.6 mg, 0.40 mmol, 2 equiv.) and $(NH_4)S_2O_8$ (142.5 mg, 0.60 mmol, 3 equiv.) in DMSO:H_2O, 600:1 (1.3/2.16 mL/μL) at 50 °C for 18 h. The crude product was then purified *via* column chromatography (70:30, hexane:ethyl acetate) to afford N-benzyl-1,3,7-trimethyl-2,6-dioxo-2,3,6,7-tetrahydro-1H-purine-8-carboxamide **4v** as a white solid (47.8 mg, 0.146 mmol, 73%). R_f = 0.14 (70:30 hexane/ethyl acetate). ^{1}H NMR (400 MHz, Chloroform-*d*) δ 7.72 (t, J = 6.0 Hz, 1H, NH), 7.40 – 7.28 (m, 5H, 5xAr-H), 4.62 (d, J = 6.0 Hz, 2H, CH_2), 4.43 (s, 3H, CH_3), 3.53 (s, 3H, CH_3), 3.41 (s, 3H, CH_3). ^{13}C NMR (101 MHz, $CDCl_3$) δ 158.1 (C), 155.7 (C), 151.6 (C), 146.3 (C), 141.0 (C), 137.5 (C), 129.0 (CH), 128.01 (2xCH), 127.98 (CH), 110.4 (C), 43.5 (CH_2), 34.7 (CH_3), 29.9 (CH_3), 28.3 (CH_3). ν_{max}/cm^{-1} 3369, 2953, 1705, 1654, 1599, 1533. M.p. = 210 – 213 °C. HRMS (ESI-TOF) m/z: $[M + H]^+$ Calcd for $C_{16}H_{17}N_5O_3H$ 328.1404; Found 328.1406.

(S)-1,3,7-Trimethyl-2,6-dioxo-N-(1-phenylethyl)-2,3,6,7-tetrahydro-1H-purine-8-carboxamide (4w)

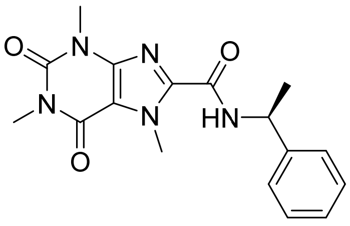

General procedure B was followed: caffeine (38.9 mg, 0.20 mmol, 1 equiv.), 2-oxo-2-(((*s*)-1-phenylethyl)amino)acetic acid (77.5 mg, 0.40 mmol, 2 equiv.) and $(NH_4)S_2O_8$ (136.4 mg, 0.60 mmol, 3 equiv.) in DMSO:H_2O, 600:1 (1.3/2.16 mL/μL) at 50 °C for 18 h. The crude product was then purified *via* column chromatography (60:40, hexane:ethyl acetate) to afford (S)-1,3,7-trimethyl-2,6-dioxo-N-(1-phenylethyl)-2,3,6,7-tetrahydro-1H-purine-8-carboxamide **4w** as a colourless oil (55.0 mg, 0.162 mmol, 81%). R_f = 0.28 (60:40 hexane/ethyl acetate). ^{1}H NMR (300 MHz, Chloroform-*d*) δ 7.62 (d, J = 8.0 Hz, 1H, NH), 7.43 – 7.27 (m, 5H, 5xAr-H), 5.24

(dq, J = 8.0, 7.0 Hz, 1H, CH), 4.39 (s, 3H, CH_3), 3.56 (s, 3H, CH_3), 3.41 (s, 3H, CH_3), 1.63 (d, J = 7.0 Hz, 3H, CH_3). ^{13}C NMR (75 MHz, $CDCl_3$) δ 157.4 (C), 155.7 (C), 151.7 (C), 146.3 (C), 142.7 (C), 141.1 (C), 129.0 (CH), 127.8 (CH), 126.3 (CH), 110.4 (C), 49.1 (CH), 34.7 (CH_3), 30.0 (CH_3), 28.3 (CH_3), 22.0 (CH_3). ν_{max}/cm^{-1} 3405, 2972, 1708, 1679, 1662, 1599, 1545, 1524, 1471. HRMS (ESI-TOF) m/z: $[M + H]^+$ Calcd for $C_{17}H_{19}N_5O_3H$ 342.1565; Found 342.1565. No racemisation of stereocentre observed: HPLC (CHIRALPAK IA, hexane/2-propanol: 95:5, flow rate: 1.0 mL min^{-1}, detection UV 275nm, 25 °C) t_R of major isomer: 37.662 min.

CSP-HPLC traces:

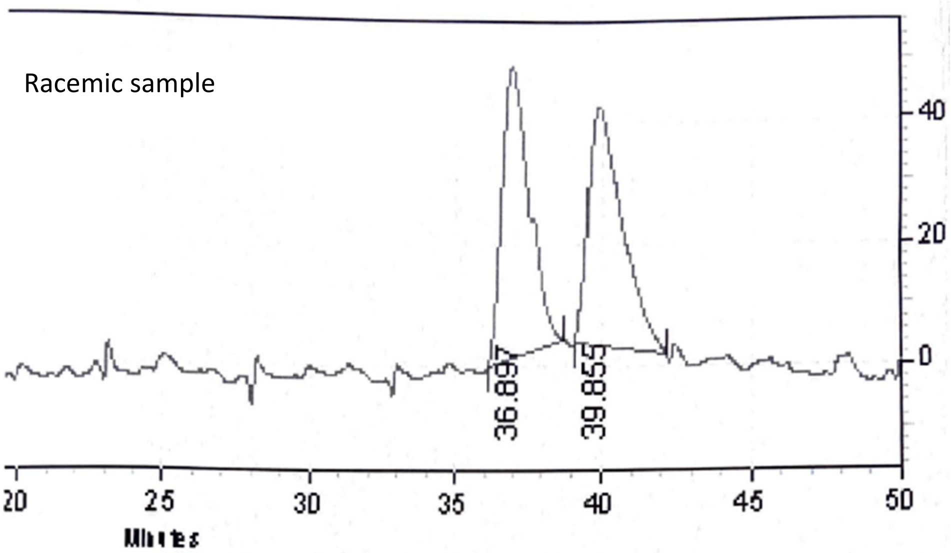

VWD: Signal A,
275 nm Results

Retention Time	Area	Area %	Height	Height %
36.897	52051816	50.08	798492	55.03
39.855	51893423	49.92	652648	44.97
Totals	103945239	100.00	1451140	100.00

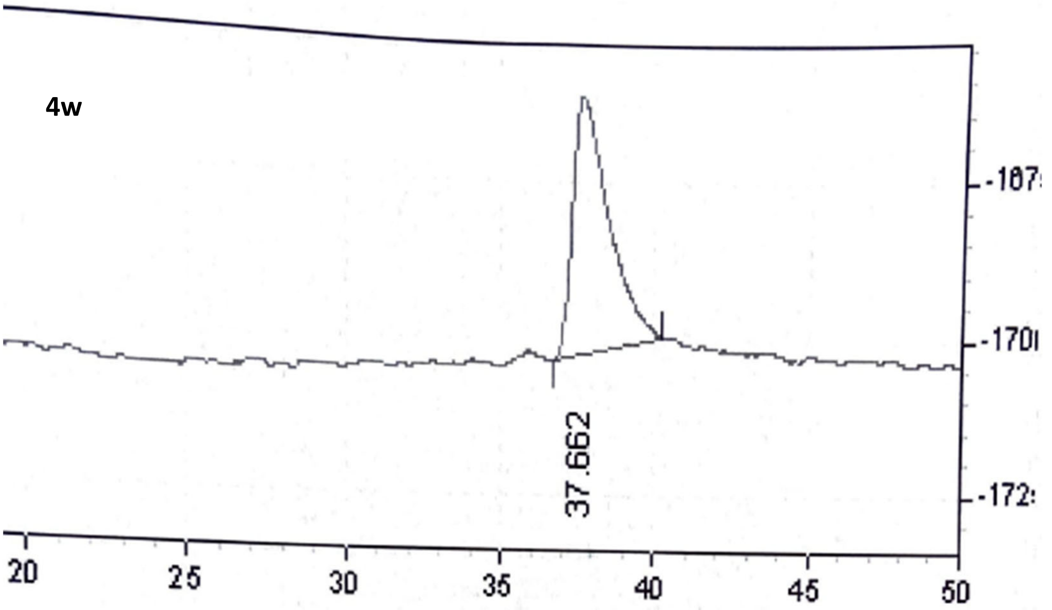

VWD: Signal A,
275 nm Results

Retention Time	Area	Area %	Height	Height %
37.662	55712723	100.00	682546	100.00
Totals	55712723	100.00	682546	100.00

1,3,7-Trimethyl-2,6-dioxo-*N*-(2,2,2-trifluoroethyl)-2,3,6,7-tetrahydro-1*H*-purine-8-carboxamide (4x)

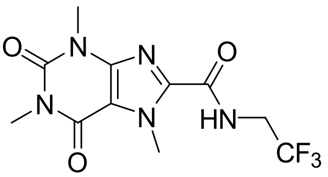

General procedure B was followed: caffeine (39.1 mg, 0.20 mmol, 1 equiv.), 2-oxo-2-((2,2,2-trifluoroethyl)amino)acetic acid (68.8 mg, 0.40 mmol, 2 equiv.) and $(NH_4)S_2O_8$ (137.2 mg, 0.60 mmol, 3 equiv.) in DMSO:H_2O, 600:1 (1.3/2.16 mL/µL) at 50 °C for 18 h. The crude product was then purified *via* column chromatography (50:50, hexane:ethyl acetate) to afford 1,3,7-trimethyl-2,6-dioxo-*N*-(2,2,2-trifluoroethyl)-2,3,6,7-tetrahydro-1*H*-purine-8-carboxamide **4x** as a white solid (45.5 mg, 0.142 mmol, 71%). R_f = 0.37 (50:50 hexane/ethyl acetate). ^{1}H NMR (400 MHz, Chloroform-*d*) δ 7.65 (t, *J* = 7.0 Hz, 1H, NH), 4.41 (s, 3H, CH_3), 4.08 (qd, *J* = 9.0, 7.0 Hz, 2H, CH_2), 3.57 (s, 3H, CH_3), 3.42 (s, 3H, CH_3). ^{13}C NMR (101 MHz, Chloroform-*d*) δ 158.2 (C), 155.7 (C), 151.6 (C), 146.3 (C), 139.8 (C), 123.9 (q, *J* = 278.6 Hz, CF_3), 110.8 (C), 40.6 (q, *J* = 35.4 Hz, CH_2), 34.8 (CH_3), 30.0 (CH_3), 28.3 (CH_3). ^{19}F NMR (376

MHz, Chloroform-*d*) δ -72.17 (t, *J* = 9.0 Hz, CF_3). ν_{max}/cm^{-1} 3372, 2945, 1709, 1678, 1658, 1603, 1527, 1475. M.p. = 190 – 193 ºC. HRMS (ESI-TOF) m/z: $[M + H]^+$ Calcd for $C_{11}H_{12}N_5O_3F_3H$ 320.0965; Found 320.0960.

1,3,7-Trimethyl-8-(pyrrolidine-1-carbonyl)-3,7-dihydro-1*H*-purine-2,6-dione (4y)

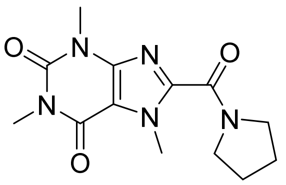

General procedure B was followed: caffeine (38.2 mg, 0.20 mmol, 1 equiv.), 2-oxo-2-(pyrrolidine-1-yl)acetic acid (58.2 mg, 0.40 mmol, 2 equiv.) and $(NH_4)S_2O_8$ (136.6 mg, 0.60 mmol, 3 equiv.) in DMSO:H_2O, 600:1 (1.3/2.16 mL/µL) at 50 ºC for 18 h. The crude product was then purified *via* column chromatography (40:60, hexane:ethyl acetate) to afford 1,3,7-trimethyl-8-(pyrrolidine-1-carbonyl)-3,7-dihydro-1*H*-purine-2,6-dione **4y** as a white solid (33.6 mg, 0.116 mmol, 58%). R*f* = 0.22 (40:60 hexane/ethyl acetate). ^{1}H NMR (300 MHz, Chloroform-*d*) δ 4.24 (s, 3H, CH_3), 3.90 (app. t, *J* = 6.5 Hz, 2H, CH_2), 3.66 (app. t, *J* = 6.5 Hz, 2H, CH_2), 3.56 (s, 3H, CH_3), 3.41 (s, 3H, CH_3), 2.02 – 1.92 (m, 4H, 2xCH_2). ^{13}C NMR (75 MHz, $CDCl_3$) δ 157.7 (C), 155.8 (C), 151.8 (C), 146.5 (C), 143.4 (C), 108.9 (C), 49.3 (CH_2), 47.1 (CH_2), 34.5 (CH_3), 29.8 (CH_3), 28.2 (CH_3), 26.6 (CH_2), 24.0 (CH_2). ν_{max}/cm^{-1} 2978, 2958, 2885, 1697, 1659, 1622, 1616, 1540. M.p. = 199 – 202 ºC. HRMS (ESI-TOF) m/z: $[M + H]^+$ Calcd for $C_{13}H_{17}N_5O_3H$ 292.1404; Found 292.1415.

1,3,7-Trimethyl-8-(piperidine-1-carbonyl)-3,7-dihydro-1*H*-purine-2,6-dione (4z)

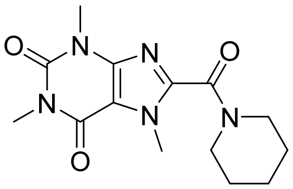

General procedure B was followed: caffeine (39.2 mg, 0.20 mmol, 1 equiv.), 2-oxo-2-(piperidin-1-yl)acetic acid (63.4 mg, 0.40 mmol, 2 equiv.) and $(NH_4)S_2O_8$ (136.3 mg, 0.60 mmol, 3 equiv.) in DMSO:H_2O, 600:1 (1.3/2.16 mL/µL) at 50 ºC for 18 h. The crude product was then purified *via* column chromatography (30:70, hexane:ethyl acetate) to afford 1,3,7-trimethyl-8-(piperidine-1-carbonyl)-3,7-dihydro-1*H*-purine-2,6-dione **4z** as a white solid (50.0 mg, 0.164 mmol, 82%). R*f* = 0.25 (30:70 hexane/ethyl acetate). ^{1}H NMR (300 MHz,

Chloroform-*d*) δ 4.07 (s, 3H, CH_3), 3.71 (t, *J* = 5.5 Hz, 2H, CH_2), 3.62 (t, *J* = 5.5 Hz, 2H, CH_2), 3.55 (s, 3H, CH_3), 3.40 (s, 3H, CH_3), 1.76 – 1.58 (m, 6H, 3xCH_2). ^{13}C NMR (75 MHz, $CDCl_3$) δ 158.2 (C), 155.6 (C), 151.8 (C), 146.9 (C), 144.1 (C), 108.5 (C), 48.5 (CH_2), 43.6 (CH_2), 33.7 (CH_3), 29.9 (CH_3), 28.2 (CH_3), 26.8 (CH_2), 25.7 (CH_2), 24.5 (CH_2). ν_{max}/cm^{-1} 2940, 2857, 2252, 1706, 1661, 1637, 1605, 1542, 1505, 1445. M.p. = 181 – 184 °C. HRMS (ESI-TOF) m/z: [M + H]$^+$ Calcd for $C_{14}H_{19}N_5O_3H$ 306.1561; Found 306.1569.

8-(Azepane-1-carbonyl)-1,3,7-trimethyl-3,7-dihydro-1*H*-purine-2,6-dione (4aa)

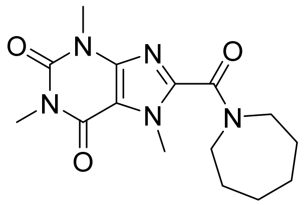

General procedure B was followed: caffeine (39.3 mg, 0.20 mmol, 1 equiv.), 2-(azepane-1-yl)-2-oxoacetic acid (69.0 mg, 0.40 mmol, 2 equiv.) and $(NH_4)S_2O_8$ (136.8 mg, 0.60 mmol, 3 equiv.) in DMSO:H_2O, 600:1 (1.3/2.16 mL/µL) at 50 °C for 18 h. The crude product was then purified *via* column chromatography (40:60, hexane:ethyl acetate) to afford 8-(azepane-1-carbonyl)-1,3,7-trimethyl-3,7-dihydro-1*H*-purine-2,6-dione **4aa** as a white solid (37.0 mg, 0.116 mmol, 58%). R*f* = 0.37 (40:60 hexane/ethyl acetate). ^{1}H NMR (300 MHz, Chloroform-*d*) δ 4.09 (s, 3H, CH_3), 3.72 – 3.62 (m, 4H, 2xCH_2), 3.56 (s, 3H, CH_3), 3.41 (s, 3H, CH_3), 1.90 – 1.73 (m, 4H, 2xCH_2), 1.68 – 1.57 (m, 4H, 2xCH_2). ^{13}C NMR (101 MHz, $CDCl_3$) δ 159.8 (C), 155.7 (C), 151.8 (C), 146.9 (C), 144.4 (C), 108.5 (C), 49.6 (CH_2), 47.1 (CH_2), 33.8 (CH_3), 29.9 (CH_3), 29.8 (CH_2), 28.2 (CH_3), 27.5 (CH_2), 27.0 (CH_2), 26.7 (CH_2). ν_{max}/cm^{-1} 3107, 2921, 2855, 1813, 1708, 1658, 1630, 1599, 1541. M.p. = 153 – 156 °C. HRMS (ESI-TOF) m/z: [M + H]$^+$ Calcd for $C_{15}H_{21}N_5O_3H$ 320.1724; Found 320.1724.

8-((2*R*,6*S*)-2,6-Dimethylpiperidine-1-carbonyl)-1,3,7-trimethyl-3,7-dihydro-1*H*-purine-2,6-dione (4ab)

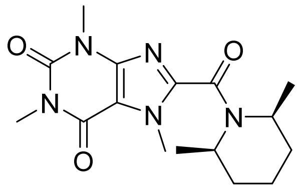

General procedure B was followed: caffeine (39.2 mg, 0.20 mmol, 1 equiv.), 2-((2*R*,6*S*)-2,6-dimethylpiperidin-1-yl)-2-oxoacetic acid (73.6 mg, 0.40 mmol, 2 equiv.) and $(NH_4)S_2O_8$ (142.5 mg, 0.60 mmol, 3 equiv.) in DMSO:H_2O, 600:1 (1.3/2.16 mL/µL) at 50 °C for 18 h. The crude product was then purified *via* column chromatography (50:50, hexane:ethyl acetate) to afford 8-((2*R*,6*S*)-2,6-dimethylpiperidine-1-carbonyl)-1,3,7-trimethyl-3,7-dihydro-1*H*-purine-2,6-dione **4ab** as a white solid (35.3 mg, 0.106 mmol, 53%). R_f = 0.26 (50:50 hexane/ethyl acetate). ^{1}H NMR (400 MHz, Chloroform-*d*) δ 4.87 (app. s, 1H, CH), 4.22 (app. s, 1H, CH), 4.02 (s, 3H, CH_3), 3.55 (s, 3H, CH_3), 3.41 (s, 3H, CH_3), 1.96 – 1.83 (m, 1H, C*H*H), 1.77 – 1.53 (m, 5H, 2xCH_2 and CH*H*), 1.36 (d, *J* = 7.0 Hz, 6H, 2xCH_3). ^{13}C NMR (101 MHz, $CDCl_3$) δ 159.6 (C), 155.6 (C), 151.8 (C), 147.2 (C), 145.3 (C), 108.1 (C), 49.7 (CH), 45.1 (CH), 33.5 (CH_3), 30.5 (CH_2), 29.9 (CH_3), 28.2 (CH_3), 22.5 (CH_2), 20.6 (CH_2), 14.2 (2xCH_3, overlapping). ν_{max}/cm^{-1} 2939, 2872, 1706, 1662, 1637, 1606, 1436. M.p. = 190 – 193 °C. HRMS (ESI-TOF) m/z: [M + H]$^+$ Calcd for $C_{16}H_{23}N_5O_3H$ 334.1871; Found 334.1871.

1,3,7-Trimethyl-8-(morpholine-4-carbonyl)-3,7-dihydro-1H-purine-2,6-dione (4ac)

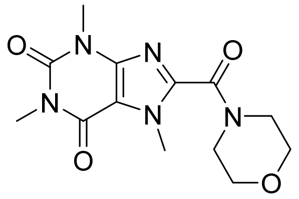

General procedure B was followed: caffeine (40.2 mg, 0.20 mmol, 1 equiv.), 2-morpholino-2-oxoacetic acid (65.5 mg, 0.40 mmol, 2 equiv.) and $(NH_4)S_2O_8$ (137.0 mg, 0.60 mmol, 3 equiv.) in DMSO:H_2O, 600:1 (1.3/2.16 mL/µL) at 50 °C for 18 h. The crude product was then purified *via* column chromatography (20:80, hexane:ethyl acetate) to afford 1,3,7-trimethyl-8-(morpholine-4-carbonyl)-3,7-dihydro-1H-purine-2,6-dione **4ac** as a white solid (36.6 mg, 0.120 mmol, 60%). R_f = 0.25 (20:80 hexane/ethyl acetate). ^{1}H NMR (400 MHz, Chloroform-*d*) δ 4.16 (s, 3H, CH_3), 3.91 (dd, *J* = 6.0, 4.5 Hz, 2H, CH_2), 3.80 (app. s, 4H, 2xCH_2), 3.75 (dd, *J* = 6.0, 4.0 Hz, 2H, CH_2), 3.55 (s, 3H, CH_3), 3.41 (s, 3H, CH_3). ^{13}C NMR (101 MHz, CDCl3) δ 158.2 (C), 155.6 (C), 151.7 (C), 146.6 (C), 142.6 (C), 109.0 (C), 67.2 (CH_2), 66.9 (CH_2), 48.0 (CH_2), 43.1 (CH_2), 34.2 (CH_3), 29.9 (CH_3), 28.3 (CH_3). ν_{max}/cm^{-1} 2959, 2921, 2856, 2252, 1704, 1658, 1604, 1543, 1506, 1441. M.p. = 229 – 232 °C. HRMS (ESI-TOF) m/z: [M + H]$^+$ Calcd for $C_{13}H_{17}N_5O_4H$ 308.1353; Found 308.1344.

8-(4,4-Difluoropiperidine-1-carbonyl)-1,3,7-trimethyl-3,7-dihydro-1*H*-purine-2,6-dione (4ad)

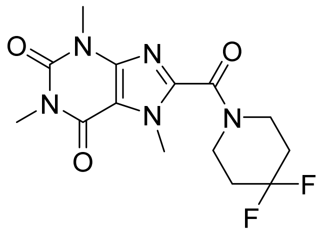

General procedure B was followed: caffeine (39.5 mg, 0.20 mmol, 1 equiv.), 2-(4,4-difluoropiperidin-1-yl)-2-oxoacetic acid (78.8 mg, 0.40 mmol, 2 equiv.) and $(NH_4)S_2O_8$ (137.2 mg, 0.60 mmol, 3 equiv.) in DMSO:H_2O, 600:1 (1.3/2.16 mL/μL) at 50 ºC for 18 h. The crude product was then purified *via* column chromatography (60:40, hexane:ethyl acetate) to afford 8-(4,4-difluoropiperidine-1-carbonyl)-1,3,7-trimethyl-3,7-dihydro-1*H*-purine-2,6-dione **4ad** as a white solid (53.4 mg, 0.156 mmol, 78%). R_F = 0.20 (60:40 hexane/ethyl acetate). ^{1}H NMR (400 MHz, Chloroform-*d*) δ 4.15 (s, 3H, CH_3), 3.95 (app. t, *J* = 6.0 Hz, 2H, CH_2), 3.89 (app. t, *J* = 6.0 Hz, 2H, CH_2), 3.55 (s, 3H, CH_3), 3.42 (s, 3H, CH_3), 2.22 – 2.05 (m, 4H, 2xCH_2). ^{13}C NMR (101 MHz, Chloroform-*d*) δ 158.2 (C), 155.6 (C), 151.7 (C), 146.6 (C), 142.4 (C), 121.5 (t, *J* = 242.8 Hz, CF_2), 109.2 (C), 44.3 (t, *J* = 5.5 Hz, CH_2), 39.8 (t, *J* = 5.5 Hz, CH_2), 34.9 (t, *J* = 24.0 Hz, CH_2), 34.2 (CH_3), 34.1 (t, *J* = 24.0 Hz, CH_2), 29.9 (CH_3), 28.3 (CH_3). ^{19}F NMR (376 MHz, Chloroform-*d*) δ -97.68 (p, *J* = 13.5 Hz, CF_2). ν_{max}/cm^{-1} 2950, 1707, 1662, 1605, 1545, 1505, 1445. M.p. = 220 – 223 ºC. HRMS (ESI-TOF) m/z: $[M + H]^+$ Calcd for $C_{14}H_{17}N_5O_3F_2H$ 342.1372; Found 342.1382.

***N*,*N*-Dibutyl-1,3,7-trimethyl-2,6-dioxo-2,3,6,7-tetrahydro-1*H*-purine-8-carboxamide (4ae)**

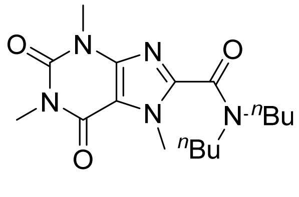

General procedure B was followed: caffeine (29.8 mg, 0.15 mmol, 1 equiv.), 2-(dibutylamino)-2-oxoacetic acid (59.5 mg, 0.30 mmol, 2 equiv.) and $(NH_4)S_2O_8$ (108.5 mg, 0.45 mmol, 3 equiv.) in DMSO:H_2O, 600:1 (1.3/2.16 mL/μL) at 50 ºC for 18 h. The crude product was then purified *via* column chromatography (70:30, hexane:ethyl acetate) to afford *N*,*N*-dibutyl-1,3,7-trimethyl-2,6-dioxo-2,3,6,7-tetrahydro-1*H*-purine-8-carboxamide **4ae** as a white solid (28.4

mg, 0.108 mmol, 54%). R$_f$ = 0.32 (60:40 hexane/ethyl acetate). ^{1}H NMR (300 MHz, Chloroform-*d*) δ 4.08 (s, 3H, CH_3), 3.55 (s, 3H, CH_3), 3.54 – 3.45 (m, 4H, 2xCH_2), 3.42 (s, 3H, CH_3), 1.72 – 1.58 (m, 4H, 2xCH_2), 1.47 – 1.33 (m, 2H, CH_2), 1.30 – 1.17 (m, 2H, CH_2), 0.98 (t, *J* = 7.3 Hz, 3H, CH_3), 0.87 (t, *J* = 7.3 Hz, 3H, CH_3). ^{13}C NMR (75 MHz, $CDCl_3$) δ 159.5 (C), 155.7 (C), 151.8 (C), 146.8 (C), 144.3 (C), 108.5 (C), 49.0 (CH_2), 46.3 (CH_2), 33.9 (CH_3), 31.3 (CH_2), 29.8 (CH_3), 29.7 (CH_2), 28.2 (CH_3), 20.5 (CH_2), 20.0 (CH_2), 14.0 (CH_3), 13.9 (CH_3). ν_{max}/cm^{-1} 2956, 2932, 2872, 1706, 1665, 1652, 1634, 1605, 1544, 1498. M.p. = 124 – 127 °C. HRMS (ESI-TOF) m/z: [M + H]$^+$ Calcd for $C_{17}H_{27}N_5O_3H$ 350.2187; Found 350.2184.

1-Hexyl-3,7-dimethyl-2,6-dioxo-2,3,6,7-tetrahydro-1*H*-purine-8-carboxamide (4af)

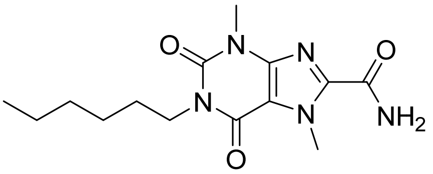

General procedure B was followed: 1-hexyltheobromide (52.5 mg, 0.20 mmol, 1 equiv.), oxamic acid (35.3 mg, 0.40 mmol, 2 equiv.) and $(NH_4)S_2O_8$ (136.9 mg, 0.60 mmol, 3 equiv.) in DMSO:H_2O, 600:1 (1.3/2.16 mL/μL) at 50 °C for 18 h. The crude product was then purified *via* column chromatography (40:60, hexane:ethyl acetate) to afford 1-hexyl-3,7-dimethyl-2,6-dioxo-2,3,6,7-tetrahydro-1*H*-purine-8-carboxamide **4af** as a white solid (36.9 mg, 0.120 mmol, 60%). R$_f$ = 0.29 (40:60 hexane/ethyl acetate). ^{1}H NMR (400 MHz, Chloroform-*d*) δ 7.22 (s, 1H, NH), 5.67 (s, 1H, NH), 4.39 (s, 3H, CH_3), 4.02 – 3.96 (m, 2H, CH_2), 3.55 (s, 3H, CH_3), 1.68 – 1.61 (m, 2H, CH_2), 1.41 – 1.28 (m, 6H, 3xCH_2), 0.88 (t, *J* = 7.0 Hz, 3H, CH_3). ^{13}C NMR (101 MHz, $CDCl_3$) δ 160.0 (C), 155.6 (C), 151.4 (C), 146.4 (C), 140.3 (C), 110.7 (C), 41.9 (CH_2), 34.8 (CH_3), 31.7 (CH_2), 29.8 (CH_3), 28.1 (CH_2), 26.8 (CH_2), 22.7 (CH_2), 14.2 (CH_3). ν_{max}/cm^{-1} 3472, 3406, 3277, 3199, 2954, 2933, 2855, 1716, 1690, 1661, 1621, 1602, 1541, 1502. M.p. = 165 – 168 °C. HRMS (ESI-TOF) m/z: [M + H]$^+$ Calcd for $C_{14}H_{21}N_5O_3H$ 308.1718; Found 308.1723.

1-Hexyl-3,7-dimethyl-2,6-dioxo-2,3,6,7-tetrahydro-1*H*-purine-8-carbonitrile (11)

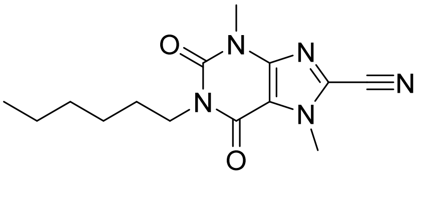

1-Hexyl-3,7-dimethyl-2,6-dioxo-2,3,6,7-tetrahydro-1*H*-purine-8-carboxamide **4ag** (10.7 mg, 0.0325 mmol, 1 equiv.) was dissolved in anhydrous DMF (0.26 mL) and allowed to cool to 0 °C with continuous stirring. Phosphoryl chloride (10 µL, 0.0975 mmol, 3 equiv.) was then added dropwise at 0 °C. After addition was complete the reaction was allowed to warm to room temperature and was left with continuous stirring for 4 h. After 4 h, the reaction was diluted in ethyl acetate (10 mL) and washed with water (20 mL). The aqueous was the extracted with ethyl acetate (3x10 mL), the organic layers combined, washed with water (2x40 mL), brine (50 mL) and dried over sodium sulphate. The solvent was the removed *in vacuo* to afford 1-hexyl-3,7-dimethyl-2,6-dioxo-2,3,6,7-tetrahydro-1*H*-purine-8-carbonitrile **11** as an off-white solid (8.0 mg, 0.0276 mmol, 85%). ^{1}H NMR (400 MHz, Chloroform-*d*) δ 4.16 (s, 3H, CH_3), 3.99 (t, *J* = 7.0 Hz, 2H, CH_2), 3.56 (s, 3H, CH_3), 1.70 – 1.58 (m, 2H, CH_2), 1.40 – 1.27 (m, 6H, 3xCH_2), 0.88 (t, *J* = 6.0 Hz, 3H, CH_3). ^{13}C NMR (101 MHz, $CDCl_3$) δ 154.7 (C), 151.1 (C), 147.6 (C), 124.8 (C), 110.0 (C), 109.7 (C), 42.1 (CH_2), 34.1 (CH_3), 31.6 (CH_2), 30.0 (CH_3), 28.0 (CH_2), 26.7 (CH_2), 22.7 (CH_2), 14.2 (CH_3). ν_{max}/cm^{-1} 2957, 2933, 2857, 2242, 1738, 1711, 1669, 1602, 1549, 1484. M.p. = 93 – 96 °C. HRMS (ESI-TOF) m/z: $[M + Na]^+$ Calcd for $C_{14}H_{19}N_5O_2Na$ 312.1431; Found 312.1426.

_N_-(Adamantan-1-yl)-2-amino-9-((2*R*,3*R*,4*S*,5*R*)-3,4-dihydroxy-5-(hydroxymethyl)tetrahydrofuran-2-yl)-6-oxo-6,9-dihydro-1*H*-purine-8-carboxamide (12)

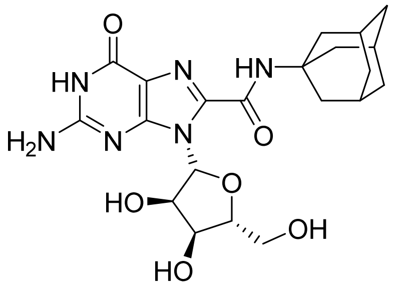

(2*R*,3*R*,4*R*,5*R*)-2-(2-Acetamido-8-(((3*S*,5*S*,7*S*)-adamantan-1-yl)carbamoyl)-6-oxo-1,6-dihydro-9*H*-purin-9-yl)-5-(acetoxymethyl)tetrahydrofuran-3,4-diyl diacetate **8e** (19.7 mg, 0.0315 mmol, 1 equiv.) was dissolved in conc. ammonia:methanol (1:1) (0.8 mL) and the reaction left stirring at room temperature for 17 h. After 17 h, the reaction was diluted in methanol (5 mL) and the solvent removed *in vacuo* to afford *N*-((3*S*,5*S*,7*S*)-adamantan-1-yl)-2-amino-9-((2*R*,3*R*,4*S*,5*R*)-3,4-dihydroxy-5-(hydroxymethyl)tetrahydrofuran-2-yl)-6-oxo-6,9-dihydro-1*H*-purine-8-carboxamide **12** as a white solid (14.4 mg, 0.0312 mmol, 99%). ^{1}H

NMR (400 MHz, DMSO-d_6) δ 7.61 (s, 1H, NH), 6.70 (d, *J* = 6.0 Hz, 1H, CH), 6.61 (s, 2H, NH_2), 5.22 (d, *J* = 6.0 Hz, 1H, CH), 4.95 – 4.85 (m, 3H, 3xOH), 4.19 (q, *J* = 5.0 Hz, 1H, CH), 3.80 (q, *J* = 4.5 Hz, 1H, CH), 3.66 (dt, *J* = 12.0, 4.5 Hz, 1H, C*H*H), 3.53 – 3.46 (m, 1H, CH*H*), 2.05 (s, 9H, 3xCH and 3xCH_2), 1.66 (app. s, 6H, 3xCH_2). ^{13}C NMR (101 MHz, DMSO) δ 157.9 (C), 156.9 (C), 154.0 (C), 152.6 (C), 138.8 (C), 115.7 (C), 89.0 (CH), 85.4 (CH), 71.1 (CH), 70.5 (CH), 62.3 (CH_2), 51.6 (C), 40.7 (CH_2), 35.9 (CH_2), 28.8 (CH). ν_{max}/cm^{-1} 3439, 3352, 3316, 3205, 2907, 2848, 2358, 2344, 2323, 1698, 1668, 1653, 1527. M.p. = >300 °C. HRMS (ESI-TOF) m/z: $[M + H]^+$ Calcd for $C_{21}H_{28}N_6O_6H$ 461.2143; Found 461.2144. $[\alpha]_D^{23.1}$ -8 (c 1.00, $CHCl_3$).

(2*R*,3*R*,4*R*,5*R*)-2-(6-Acetamido-8-((adamantan-1-yl)carbamoyl)-2-phenyl-9*H*-purin-9-yl)-5-(acetoxymethyl)tetrahydrofuran-3,4-diyl diacetate (13)

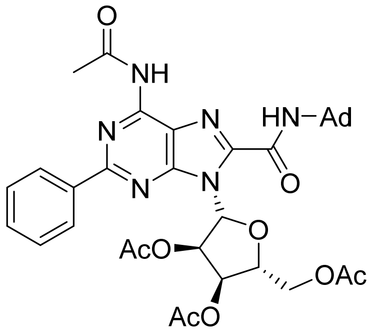

(2*R*,3*R*,4*R*,5*R*)-2-(6-Acetamido-8-(((3*S*,5*S*,7*S*)-adamantan-1-yl)carbamoyl)-2-chloro-9*H*-purin-9-yl)-5-(acetoxymethyl)tetrahydrofuran-3,4-diyl diacetate **10c** (15.3 mg, 0.024 mmol, 1 equiv.), phenylboronic acid (12.2 mg, 0.096 mmol, 4 equiv.), $Pd(PPh_3)_4$ (5.1 mg, 0.0048 mmol, 20 mol%) and K_2CO_3 (6.4 mg, 0.048 mmol, 2 equiv.) were dissolved in THF:H_2O (2:1) (0.45 mL) and the reaction mixture was heated at reflux for 19 h. After 19 h, the reaction was diluted in ethyl acetate (20 mL) and washed with water (20 mL). The aqueous was then extracted with ethyl acetate (3x10 mL), the organic layers were combined, washed with water (3x20 mL), brine (3x20 mL), dried over sodium sulphate and the solvent removed *in vacuo* to afford the crude. The crude was then purified *via* column chromatography (70:30, hexane:ethyl acetate) to afford (2*R*,3*R*,4*R*,5*R*)-2-(6-acetamido-8-(((3*S*,5*S*,7*S*)-adamantan-1-yl)carbamoyl)-2-phenyl-9*H*-purin-9-yl)-5-(acetoxymethyl)tetrahydrofuran-3,4-diyl diacetate **13** as a colourless oil (11.2 mg, 0. 016 mmol, 68%). R_f = 0.20 (70:30, hexane:ethyl acetate). ^{1}H NMR (400 MHz, Chloroform-*d*) δ 8.52 – 8.50 (m, 1H, Ar-H), 8.50 – 8.48 (m, 1H, CH), 8.39 (s, 1H, NH), 7.55 – 7.48 (m, 4H, 4xAr-H), 7.39 (s, 1H, NH), 6.44 (dd, *J* = 6.5, 3.0 Hz, 1H, CH), 6.22 (dd, *J* =

8.0, 6.5 Hz, 1H, CH), 4.49 (dd, *J* = 12.0, 3.0 Hz, 1H, C*H*H), 4.37 (ddd, *J* = 8.0, 5.0, 3.0 Hz, 1H, CH), 4.24 (dd, *J* = 12.0, 5.0 Hz, 1H, CH*H*), 2.91 (s, 3H, CH_3), 2.18 – 2.15 (m, 12H, CH_3, 3xCH and 3xCH_2), 2.13 (s, 3H, CH_3), 1.78 – 1.72 (m, 9H, CH_3 and 3xCH_2). ^{13}C NMR (101 MHz, $CDCl_3$) δ 171.4 (C), 170.8 (C), 169.9 (C), 169.6 (C), 160.9 (C), 157.2 (C), 153.1 (C), 149.8 (C), 143.7 (C), 137.4 (C), 131.2 (CH), 128.92 (CH), 128.88 (CH), 118.5 (C), 88.4 (CH), 79.0 (CH), 73.1 (CH), 69.7 (CH), 62.5 (CH_2), 53.1 (C), 41.6 (CH_2), 36.4 (CH_2), 29.6 (CH), 26.5 (CH_3), 20.7 (2xCH_3 overlapping), 20.6 (CH_3). ν_{max}/cm^{-1} 3350, 2965, 2910, 2851, 2360, 1750, 1706, 1681, 1601, 1576, 1528. HRMS (ESI-TOF) m/z: $[M + H]^+$ Calcd for $C_{35}H_{40}N_6O_9H$ 689.2930; Found 689.2930. $[\alpha]_D^{22.8}$ +8 (c 1.00, $CHCl_3$).

6.NMR Spectra

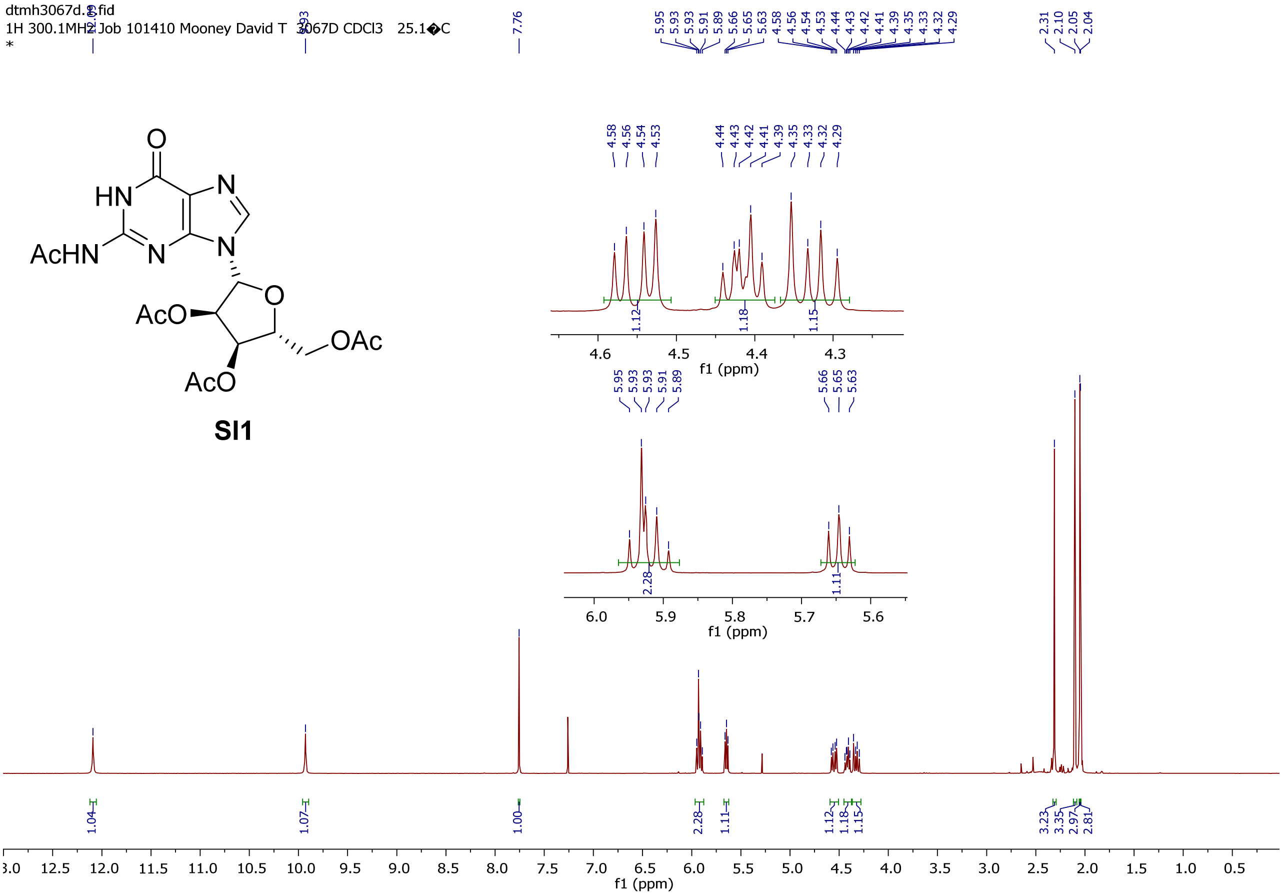

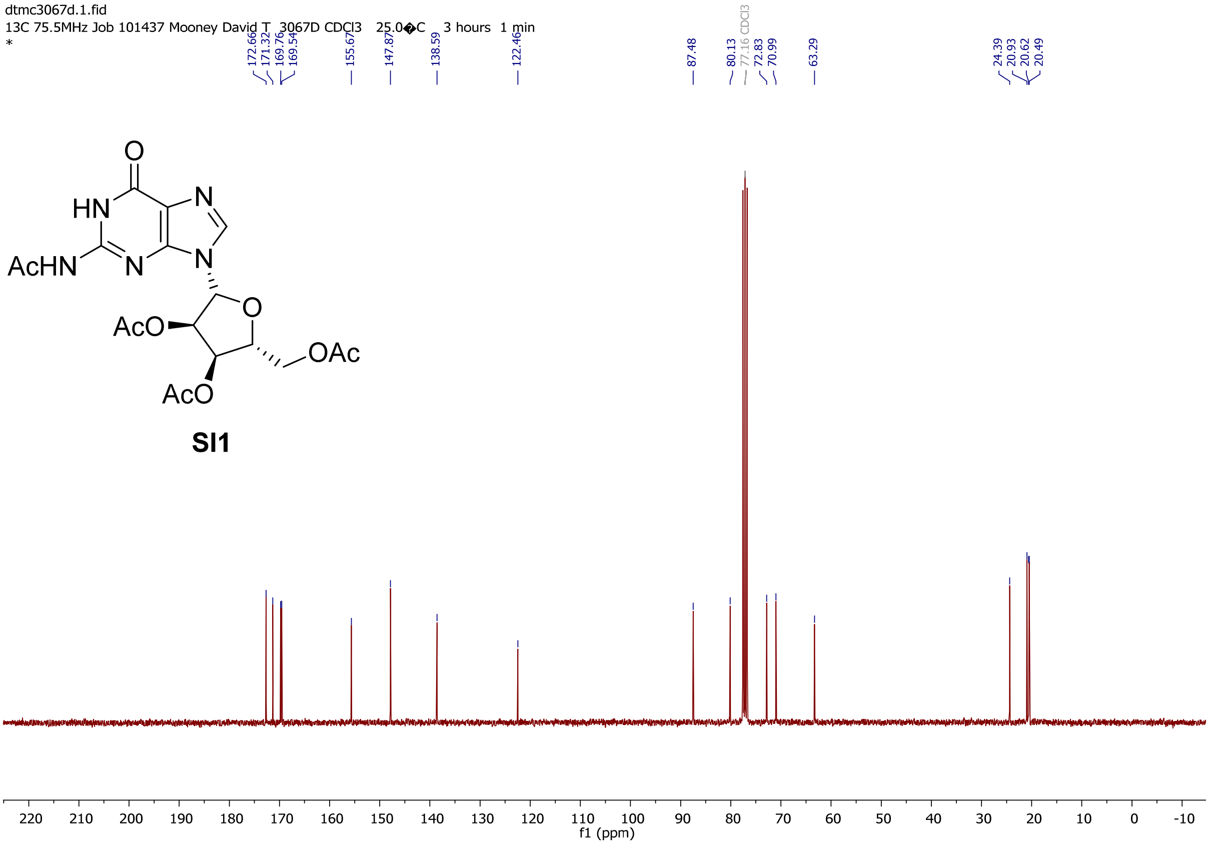

dtmh3091a.1.fid ^{1}H NMR, 400 MHz, CDCl$_3$

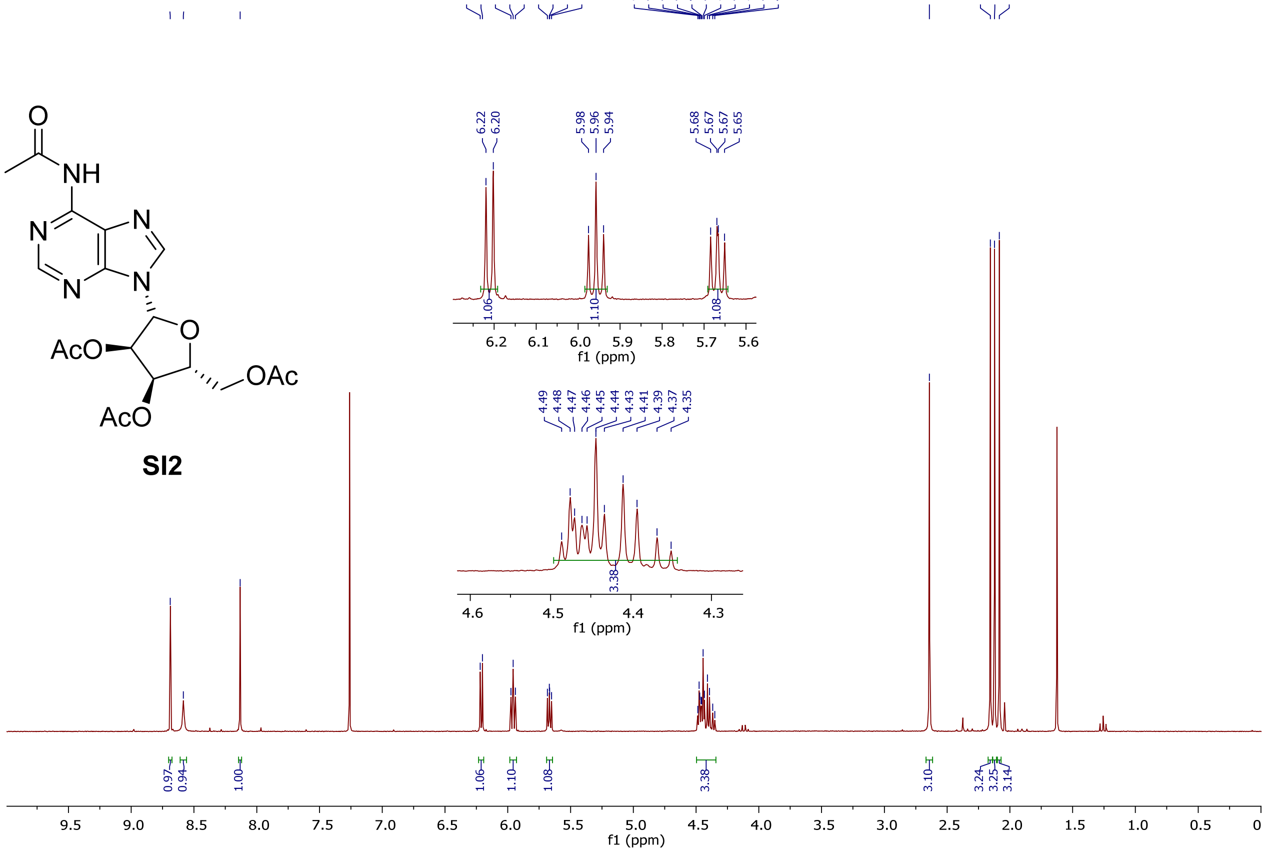

^{13}C NMR, 101 MHz, CDCl$_3$

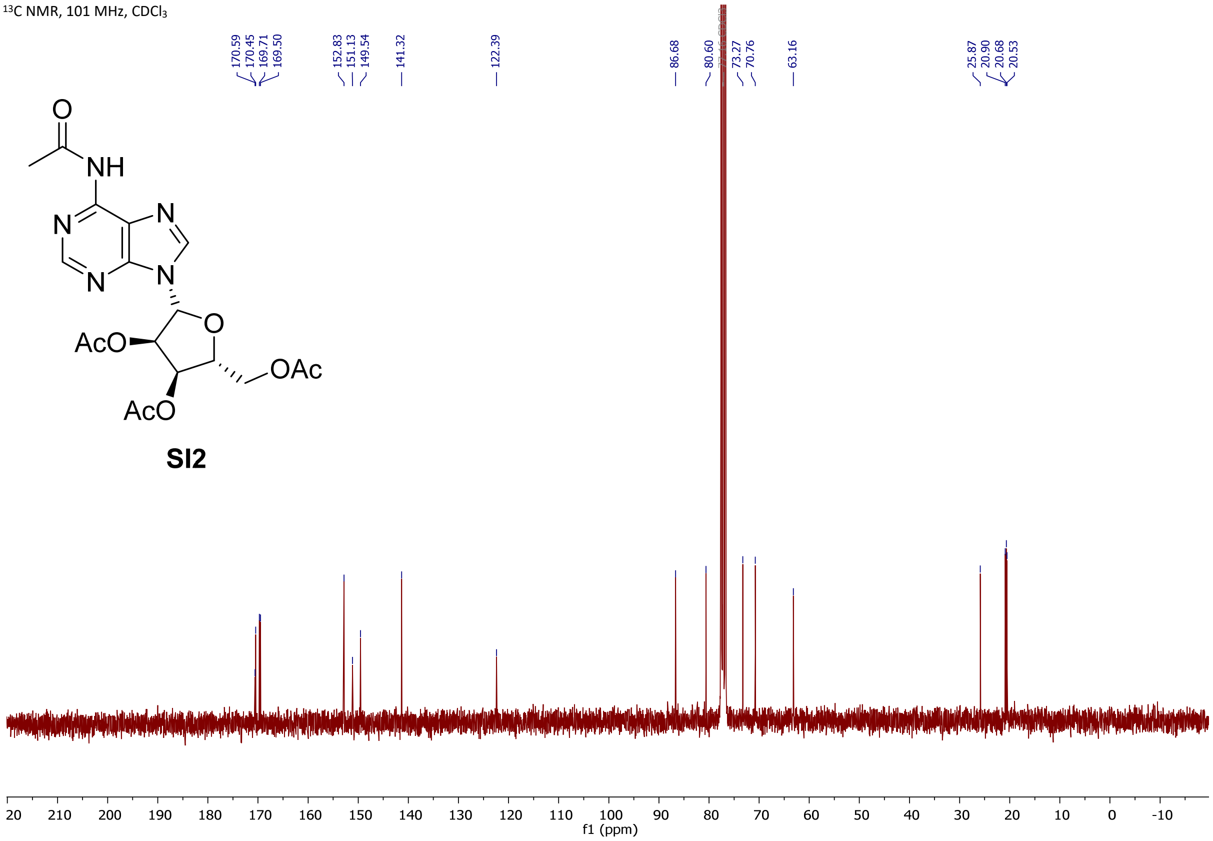

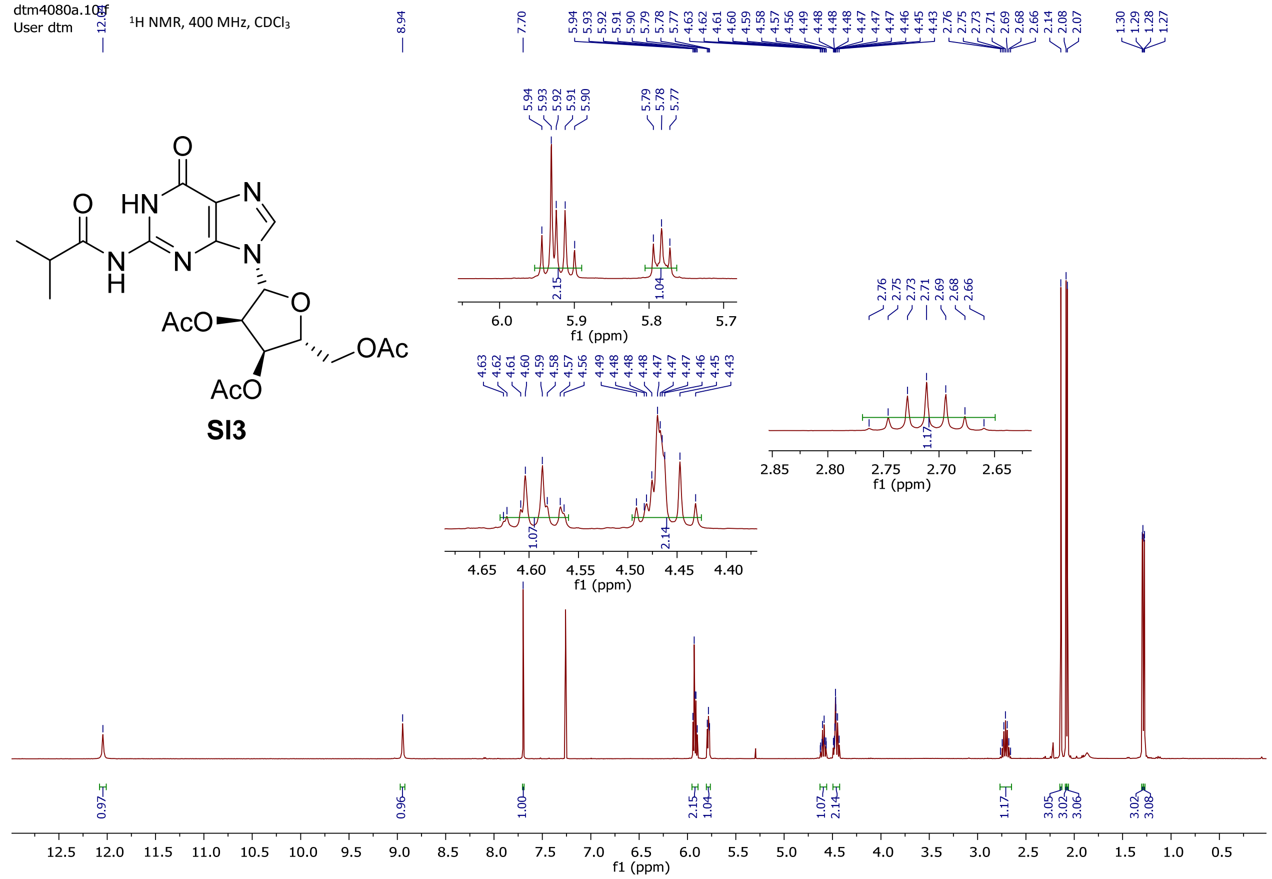
dtm4080a.10.fid
User dtm
¹H NMR, 400 MHz, CDCl₃
12.07
8.94
7.70
5.94 5.93 5.92 5.91 5.90 5.79 5.78 5.77 4.63 4.62 4.61 4.60 4.59 4.58 4.57 4.56 4.49 4.48 4.48 4.48 4.47 4.47 4.47 4.46 4.45 4.43 2.76 2.75 2.73 2.71 2.69 2.68 2.66 2.14 2.08 2.07 1.30 1.29 1.28 1.27
0.97 0.96 1.00 2.15 1.04 1.07 2.14 1.17 3.05 3.02 3.06 3.02 3.08
f1 (ppm)
SI3

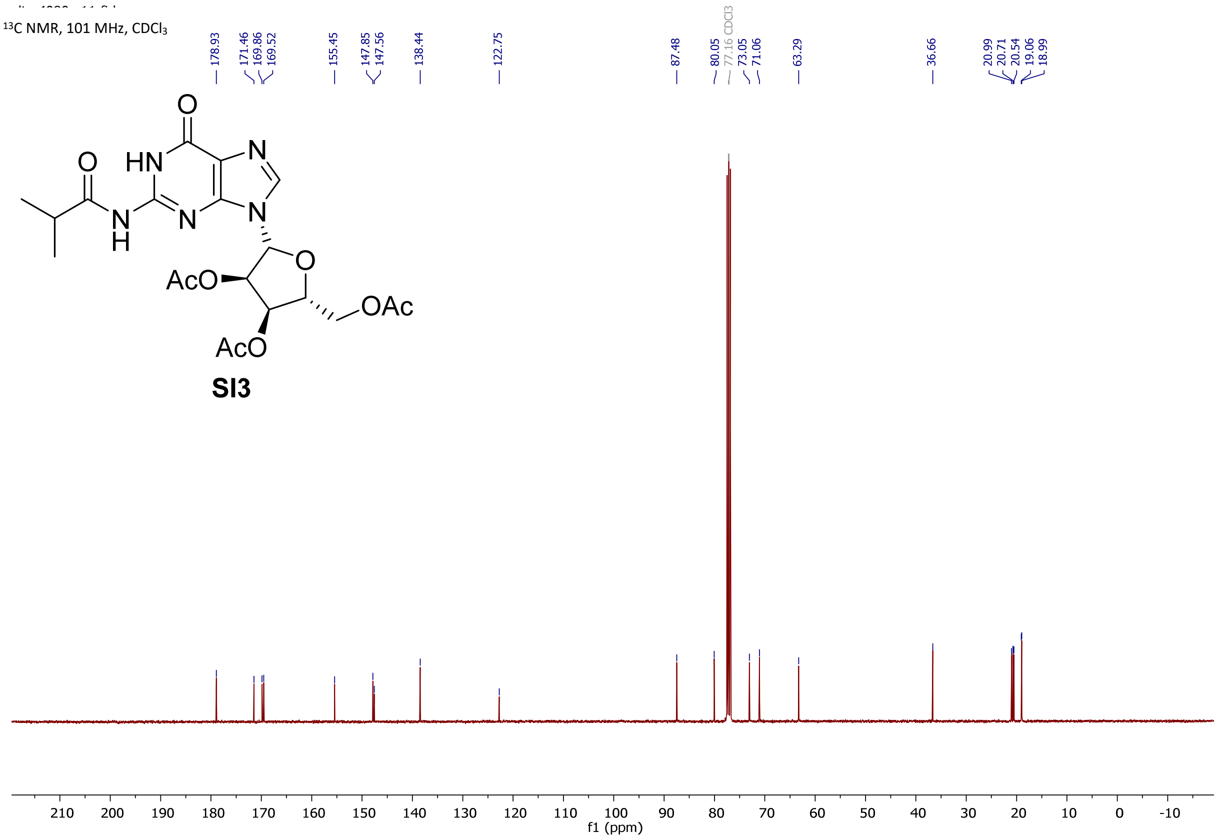
¹³C NMR, 101 MHz, CDCl₃
178.93
171.46 169.86 169.52
155.45
147.85 147.56
138.44
122.75
87.48
80.05
77.16 CDCl3
73.05 71.06
63.29
36.66
20.99 20.71 20.54 19.06 18.99
f1 (ppm)
SI3

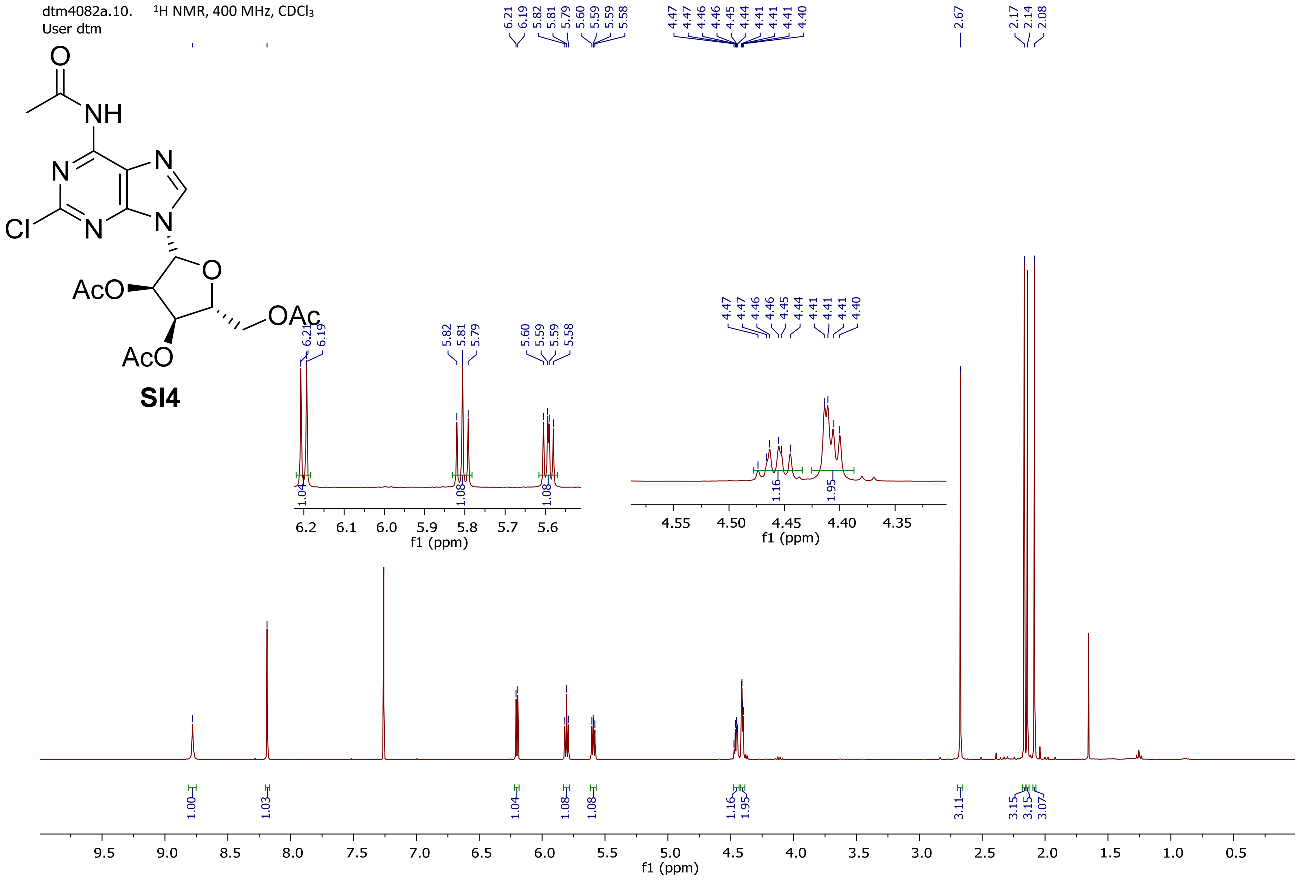
dtm4082a.10.
User dtm
¹H NMR, 400 MHz, CDCl₃
SI4
f1 (ppm)

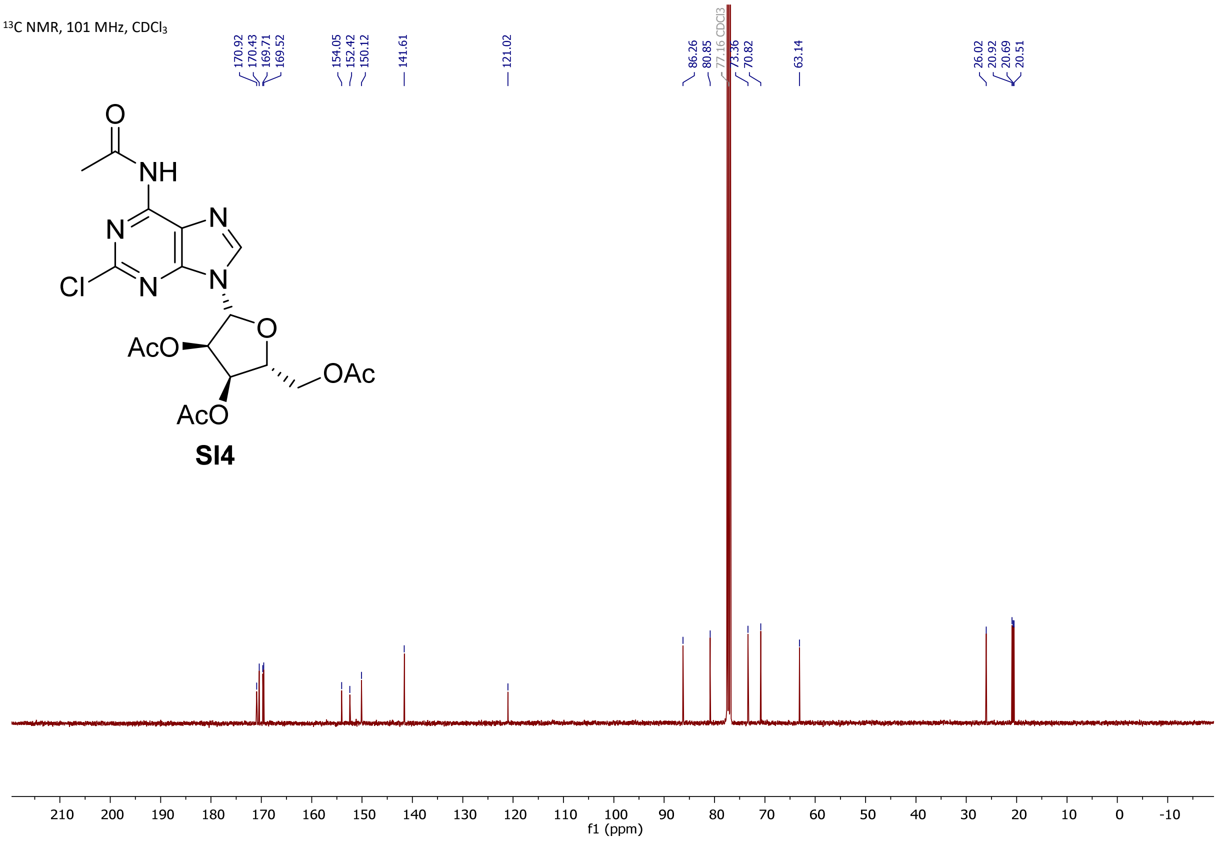
¹³C NMR, 101 MHz, CDCl₃
SI4
f1 (ppm)

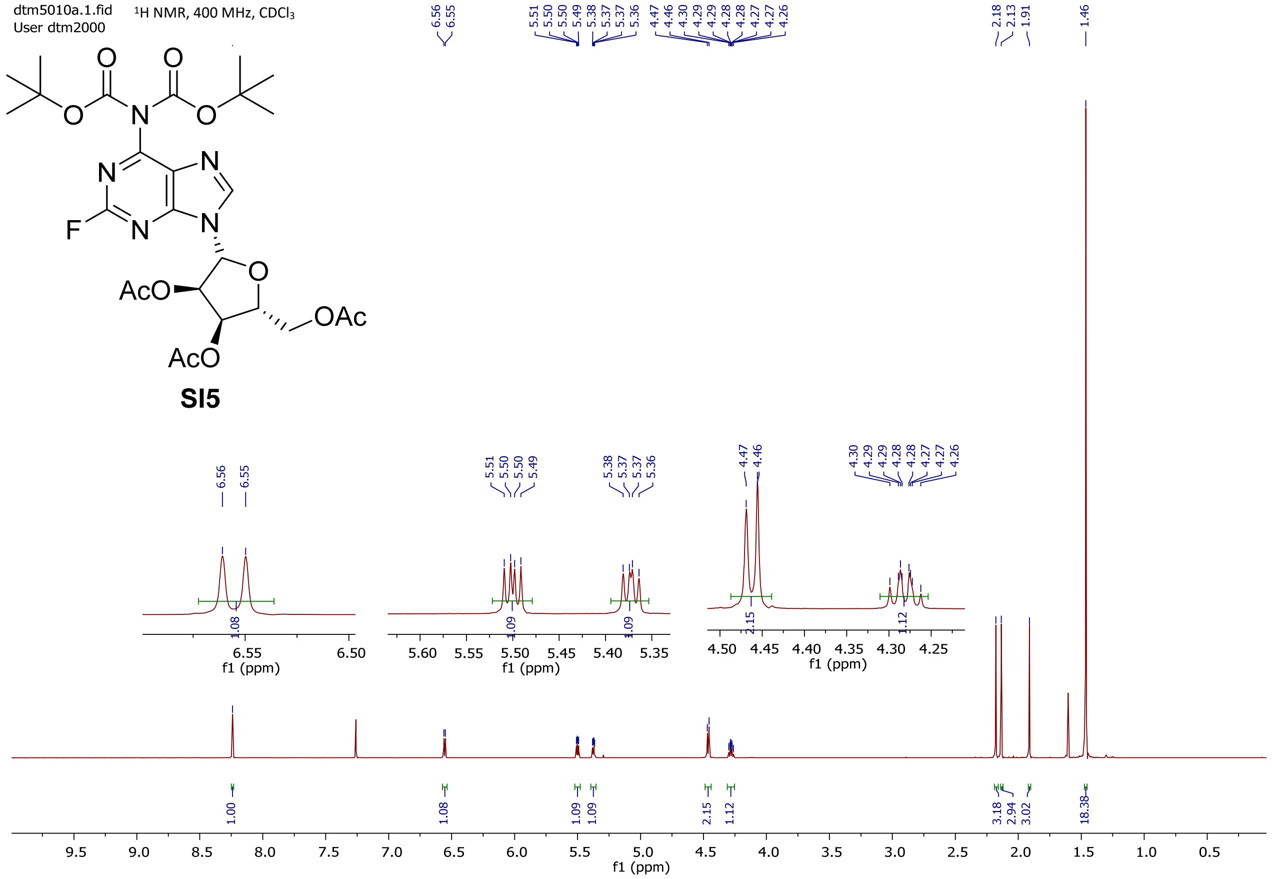
dtm5010a.1.fid
User dtm2000
¹H NMR, 400 MHz, CDCl₃
SI5
f1 (ppm)

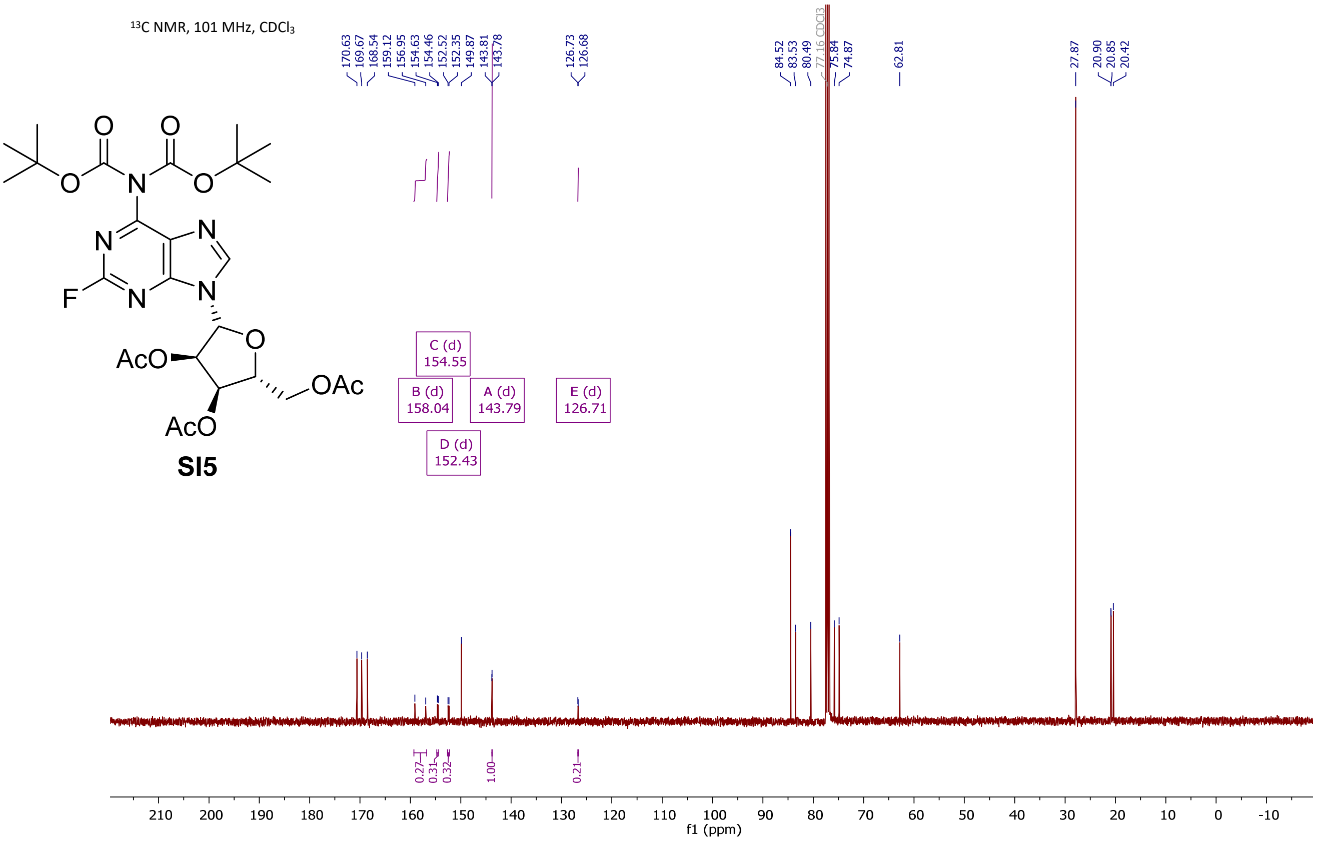
¹³C NMR, 101 MHz, CDCl₃
SI5
C (d) 154.55
B (d) 158.04
A (d) 143.79
E (d) 126.71
D (d) 152.43
f1 (ppm)

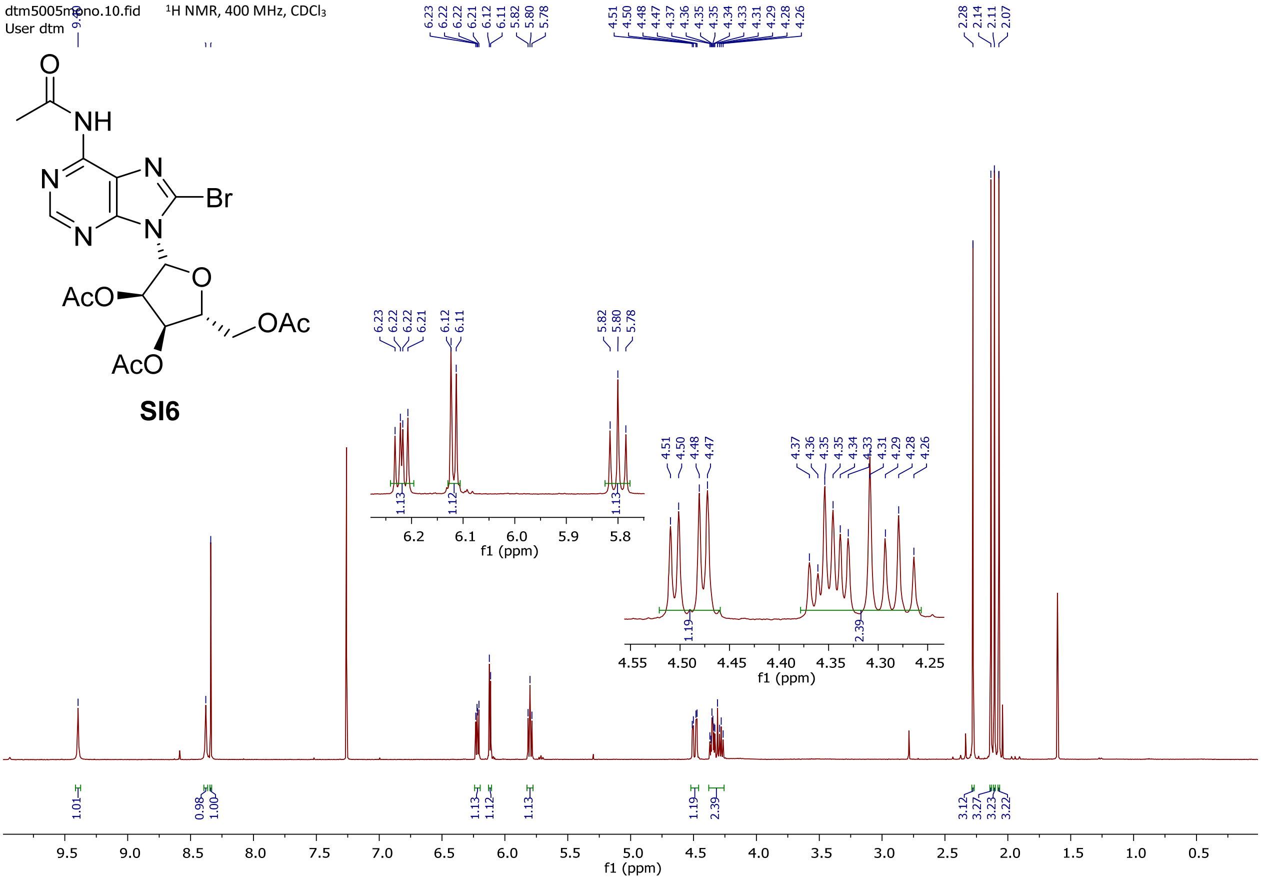
dtm5005mono.10.fid
User dtm
¹H NMR, 400 MHz, CDCl₃
SI6
6.23 6.22 6.22 6.21 6.12 6.11 5.82 5.80 5.78
4.51 4.50 4.48 4.47 4.37 4.36 4.35 4.35 4.34 4.33 4.31 4.29 4.28 4.26
2.28 2.14 2.11 2.07
f1 (ppm)

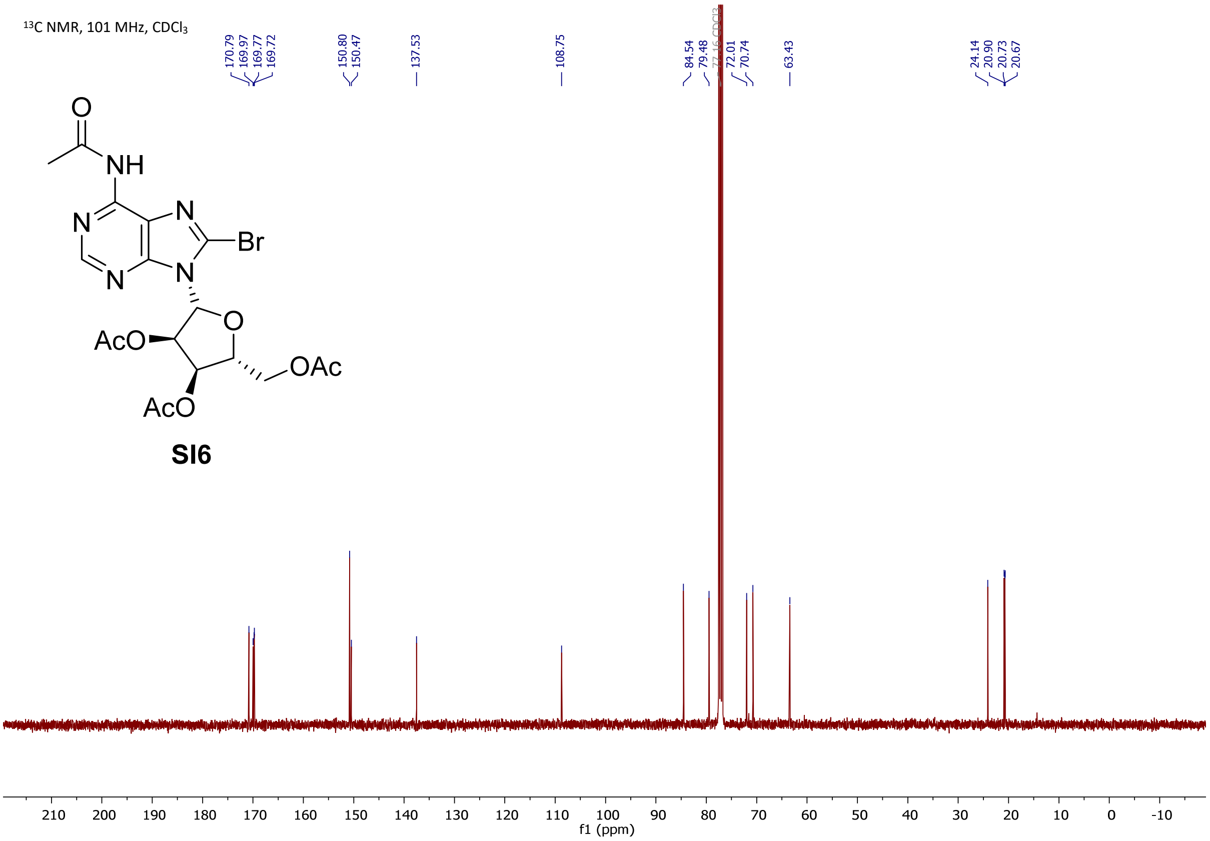
¹³C NMR, 101 MHz, CDCl₃
SI6
170.79 169.97 169.77 169.72
150.80 150.47
137.53
108.75
84.54 79.48 72.01 70.74
63.43
24.14 20.90 20.73 20.67
f1 (ppm)

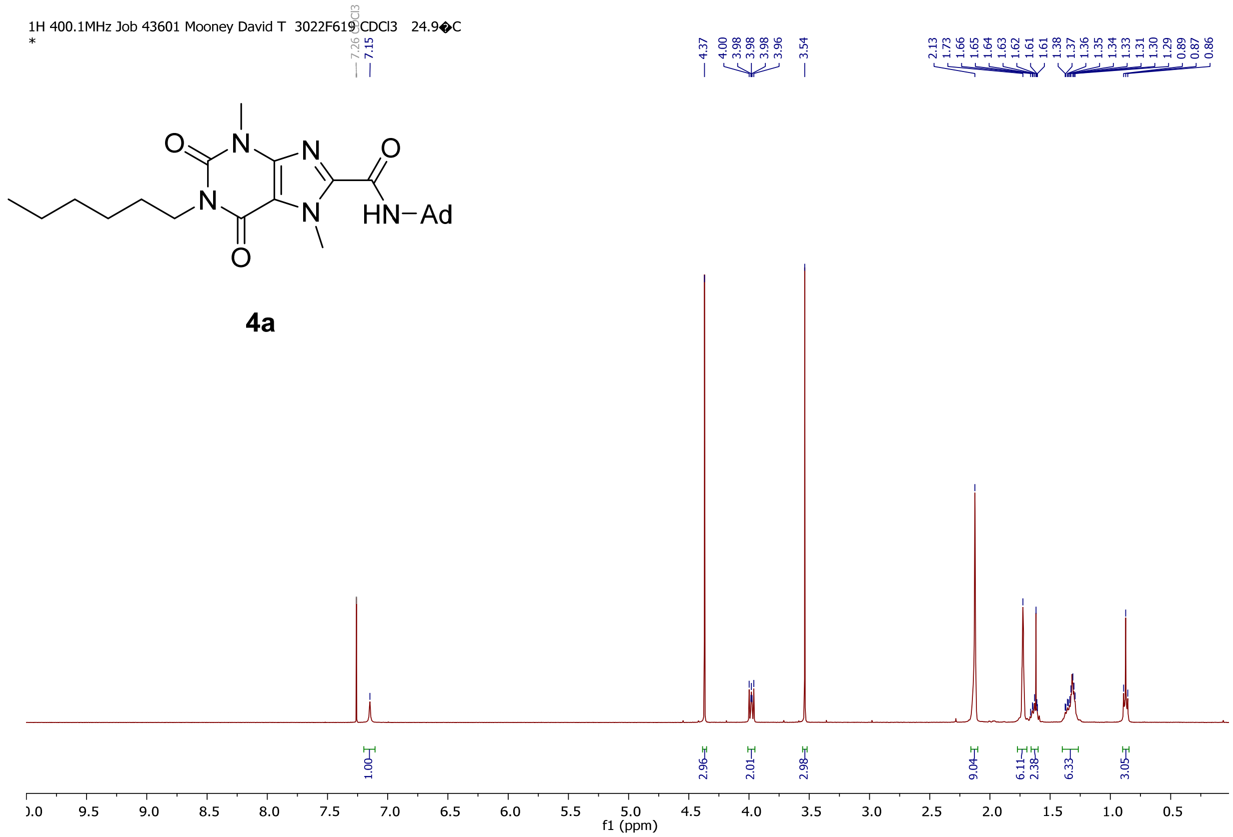
1H 400.1MHz Job 43601 Mooney David T 3022F619 CDCl3 24.9�C
*
7.26 CDCl3
7.15
4.37
4.00
3.98
3.98
3.98
3.96
3.54
2.13
1.73
1.66
1.65
1.64
1.63
1.62
1.61
1.61
1.38
1.37
1.36
1.35
1.34
1.33
1.31
1.30
1.29
0.89
0.87
0.86
O
N
N
O
N
N
HN−Ad
O
4a
1.00
2.96
2.01
2.98
9.04
6.11
2.38
6.33
3.05
f1 (ppm)

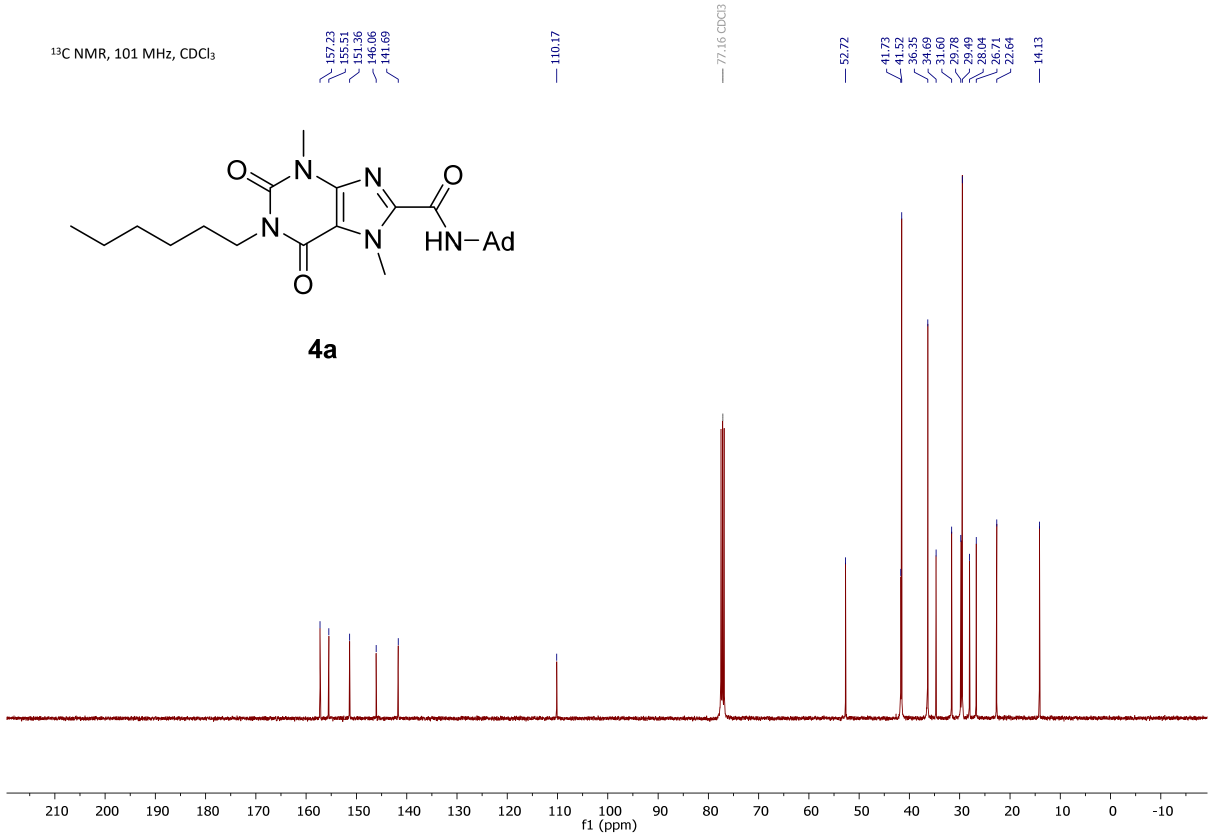
¹³C NMR, 101 MHz, CDCl₃
157.23
155.51
151.36
146.06
141.69
110.17
77.16 CDCl3
52.72
41.73
41.52
36.35
34.69
31.60
29.78
29.49
28.04
26.71
22.64
14.13
O
N
N
O
N
N
HN−Ad
O
4a
f1 (ppm)

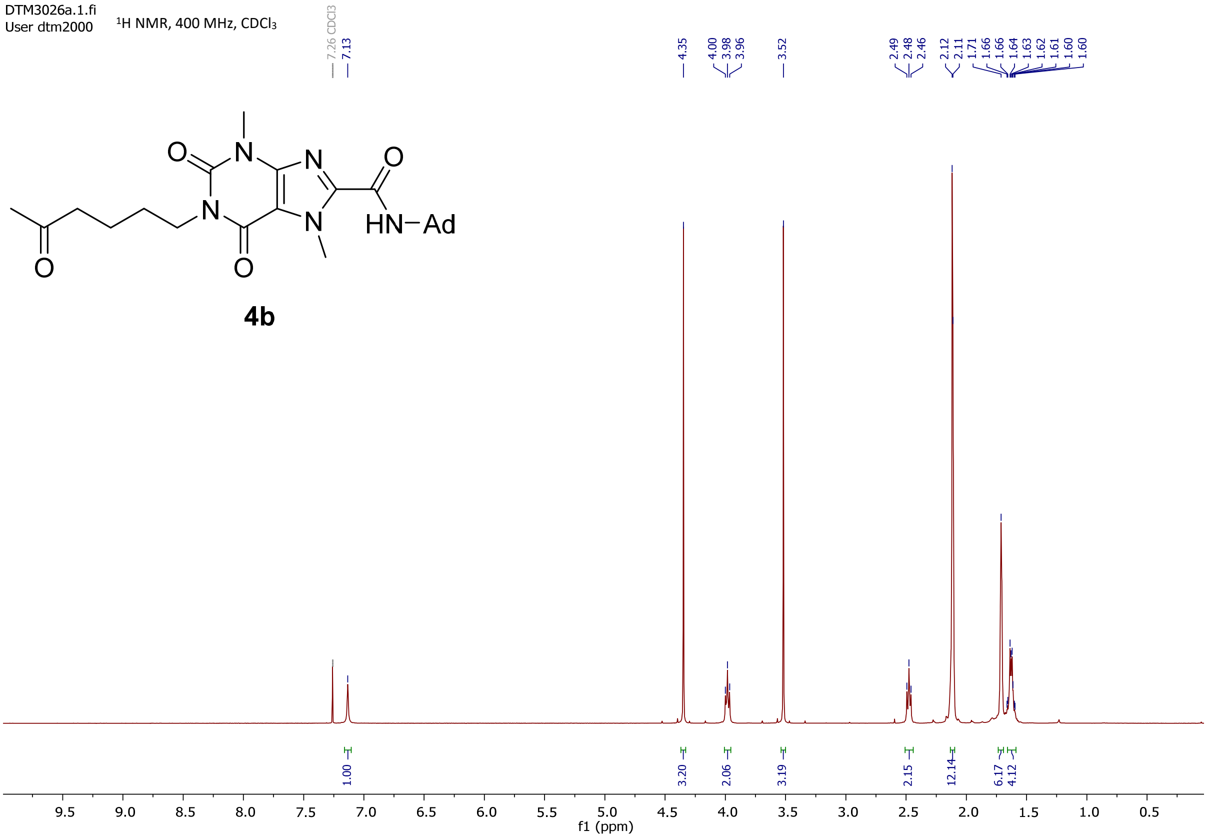
DTM3026a.1.fi
User dtm2000
1H NMR, 400 MHz, CDCl3
7.26 CDCl3
7.13
4.35
4.00
3.98
3.96
3.52
2.49
2.48
2.46
2.12
2.11
1.71
1.66
1.64
1.63
1.62
1.61
1.60
1.60
HN−Ad
4b
1.00
3.20
2.06
3.19
2.15
12.14
6.17
4.12
f1 (ppm)

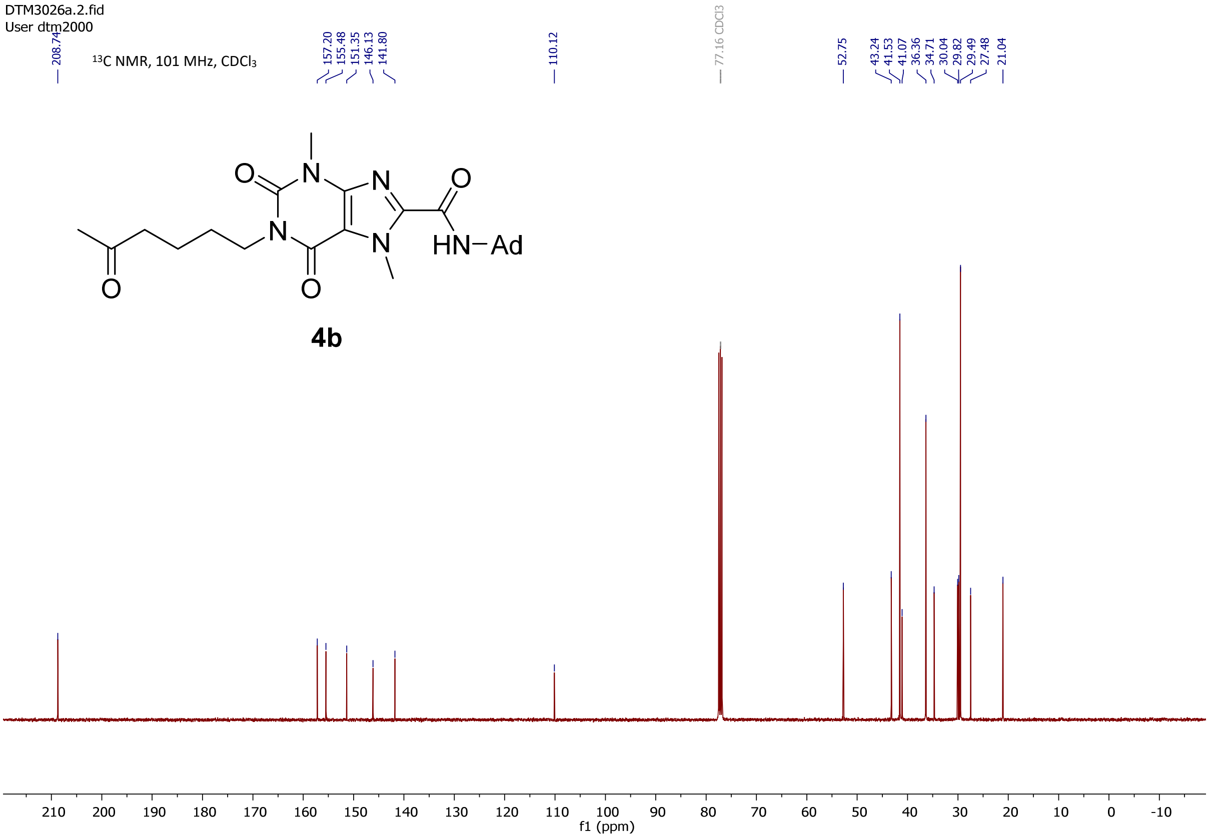
DTM3026a.2.fid
User dtm2000
13C NMR, 101 MHz, CDCl3
208.74
157.20
155.48
151.35
146.13
141.80
110.12
77.16 CDCl3
52.75
43.24
41.53
41.07
36.36
34.71
30.04
29.82
29.49
27.48
21.04
HN−Ad
4b
f1 (ppm)

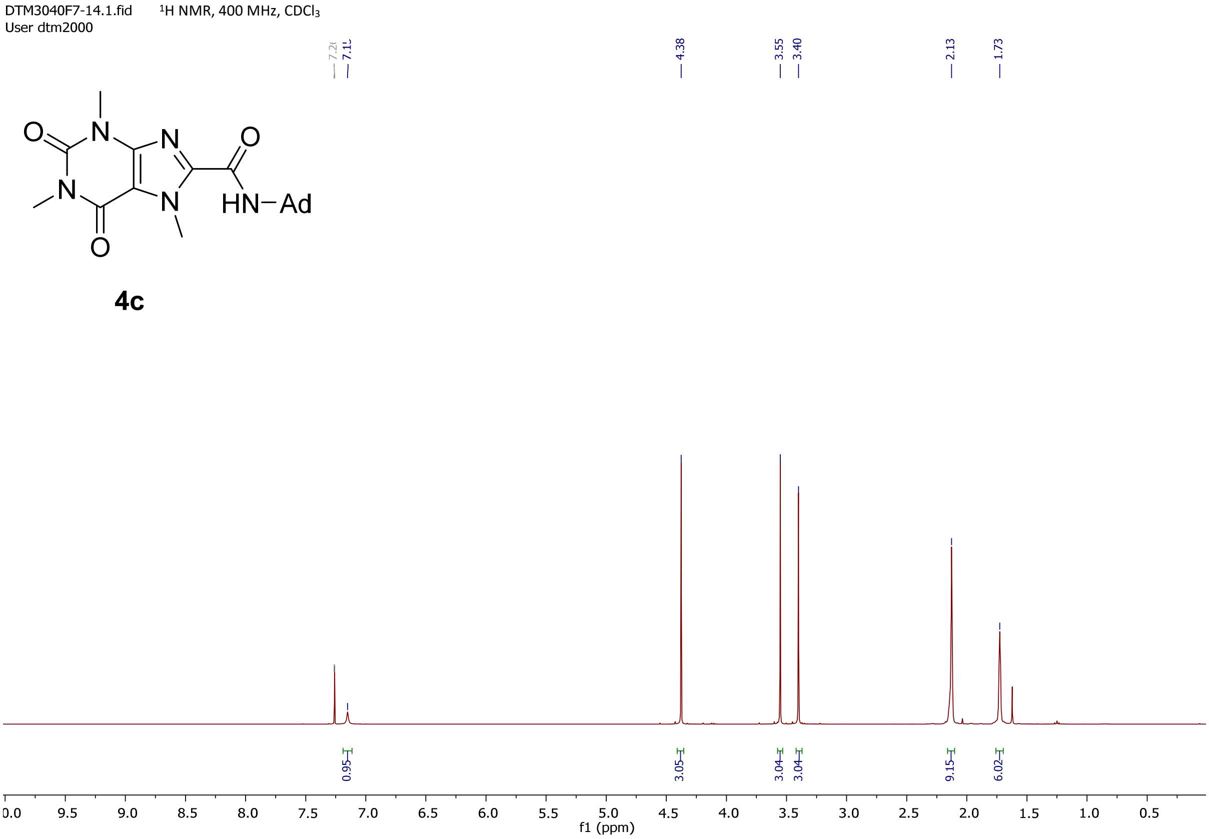
DTM3040F7-14.1.fid
^{1}H NMR, 400 MHz, $CDCl_3$
User dtm2000
4.38
3.55
3.40
2.13
1.73
O
N
N
O
N
N
O
HN−Ad
4c
0.95
3.05
3.04
3.04
9.15
6.02
9.5
9.0
8.5
8.0
7.5
7.0
6.5
6.0
5.5
5.0
4.5
4.0
3.5
3.0
2.5
2.0
1.5
1.0
0.5
f1 (ppm)

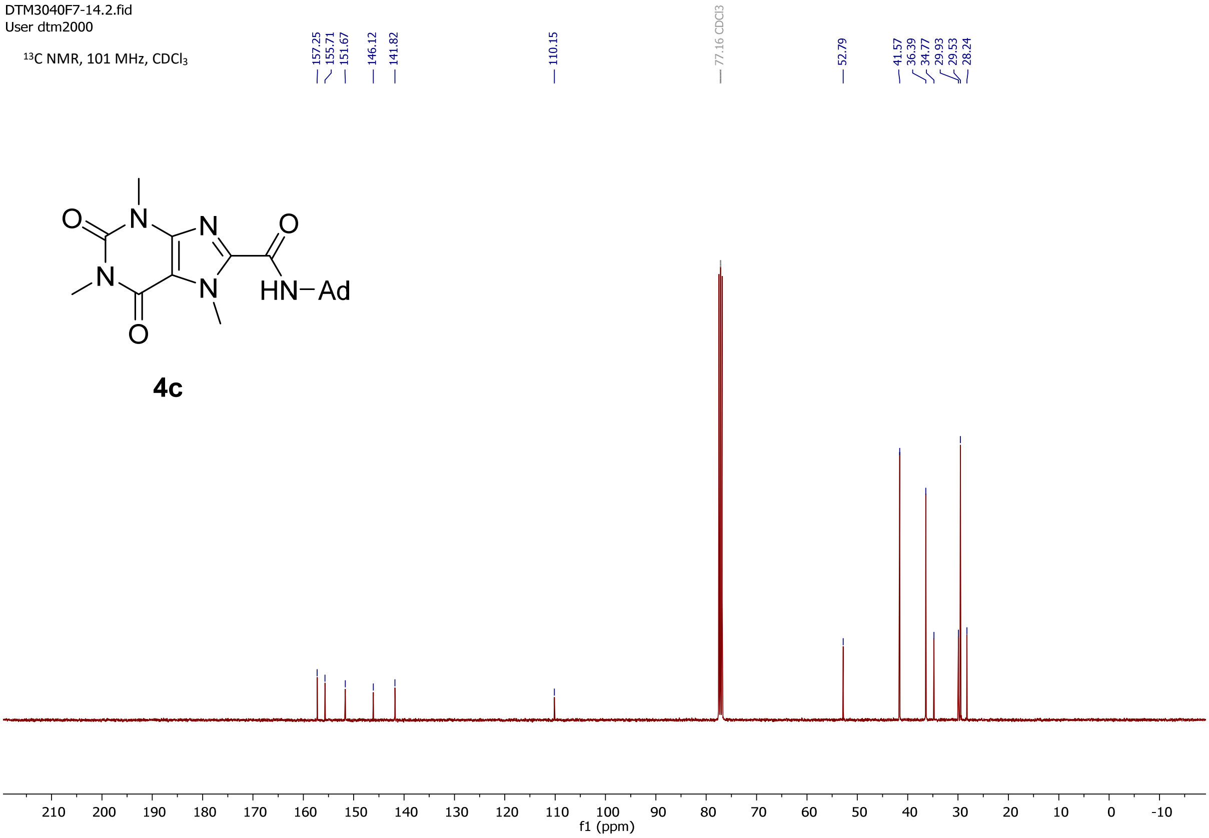
DTM3040F7-14.2.fid
User dtm2000
^{13}C NMR, 101 MHz, $CDCl_3$
157.25
155.71
151.67
146.12
141.82
110.15
77.16 CDCl3
52.79
41.57
36.39
34.77
29.93
29.53
28.24
O
N
N
O
N
N
O
HN−Ad
4c
210
200
190
180
170
160
150
140
130
120
110
100
90
80
70
60
50
40
30
20
10
0
-10
f1 (ppm)

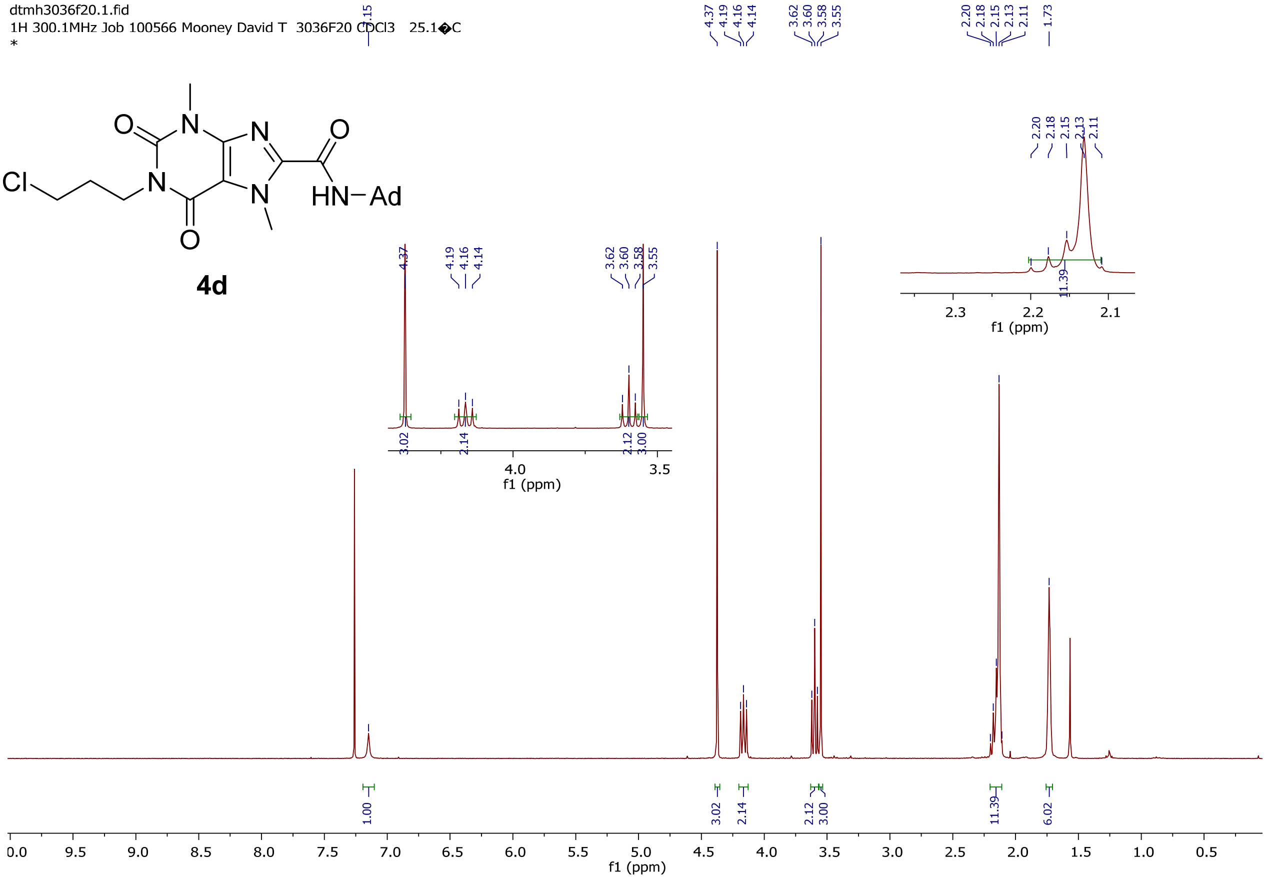
dtmh3036f20.1.fid
1H 300.1MHz Job 100566 Mooney David T 3036F20 CDCl3 25.1�C
*
7.15
4.37
4.19
4.16
4.14
3.62
3.60
3.58
3.55
2.20
2.18
2.15
2.13
2.11
1.73
Cl
N
N
N
N
O
O
O
HN−Ad
4d
3.02
2.14
2.12
3.00
4.0
3.5
f1 (ppm)
11.39
2.3
2.2
2.1
f1 (ppm)
1.00
6.02
0.0
9.5
9.0
8.5
8.0
7.5
7.0
6.5
6.0
5.5
5.0
4.5
4.0
3.5
3.0
2.5
2.0
1.5
1.0
0.5
f1 (ppm)

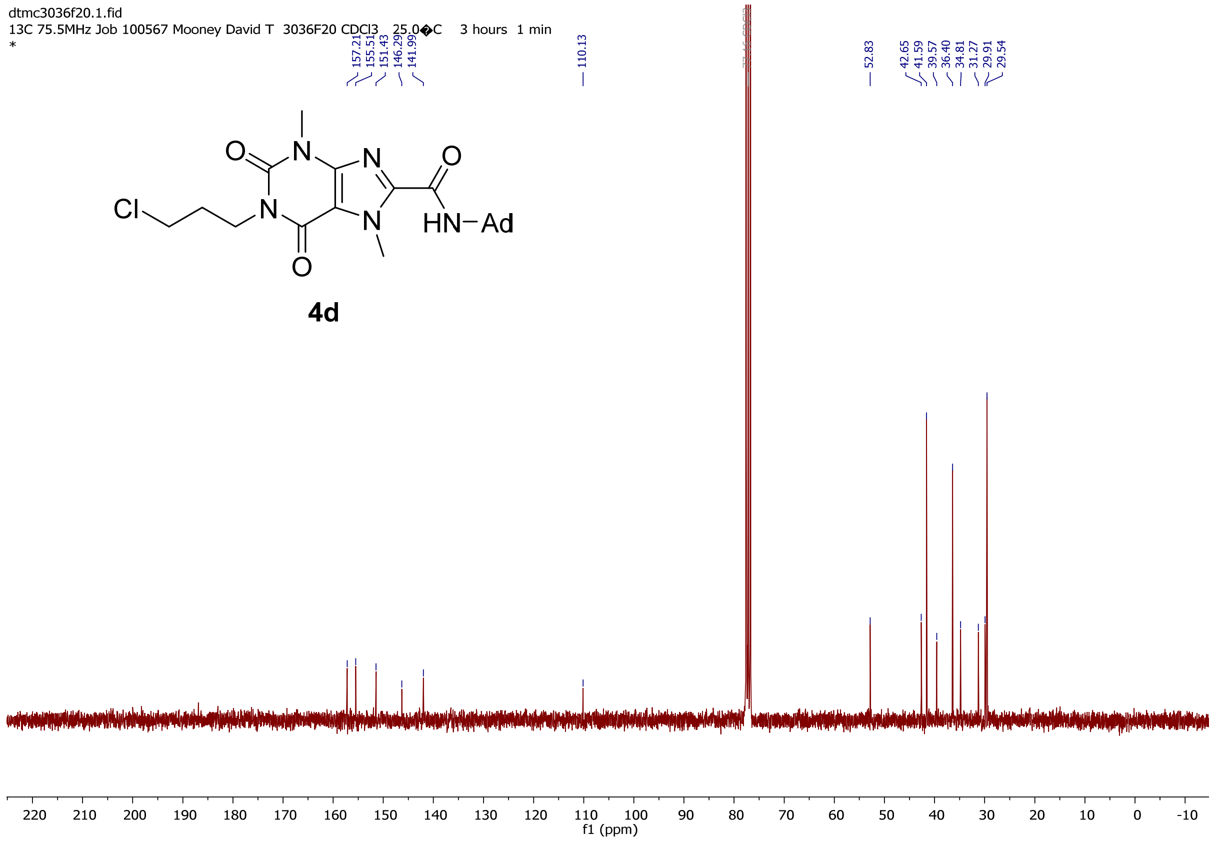
dtmc3036f20.1.fid
13C 75.5MHz Job 100567 Mooney David T 3036F20 CDCl3 25.0�C 3 hours 1 min
*
157.21
155.51
151.43
146.29
141.99
110.13
52.83
42.65
41.59
39.57
36.40
34.81
31.27
29.91
29.54
Cl
N
N
N
N
O
O
O
HN−Ad
4d
220
210
200
190
180
170
160
150
140
130
120
110
100
90
80
70
60
50
40
30
20
10
0
-10
f1 (ppm)

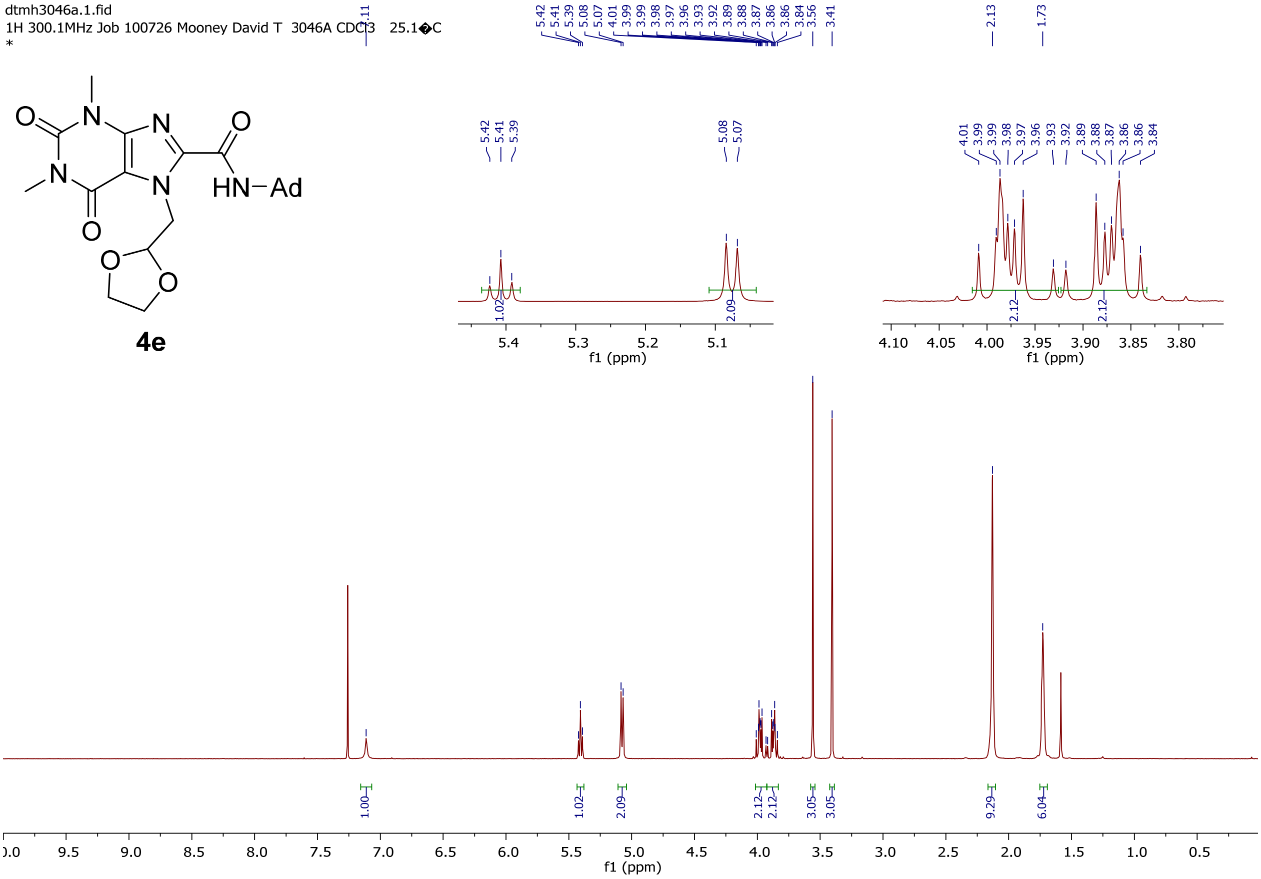
dtmh3046a.1.fid
1H 300.1MHz Job 100726 Mooney David T 3046A CDCl3 25.1�C
*
7.11
5.42 5.41 5.39 5.08 5.07 4.01 3.99 3.99 3.98 3.97 3.96 3.93 3.92 3.89 3.88 3.87 3.86 3.86 3.84 3.56 3.41
2.13
1.73
4e
1.00
1.02
2.09
2.12
2.12
3.05
3.05
9.29
6.04
f1 (ppm)

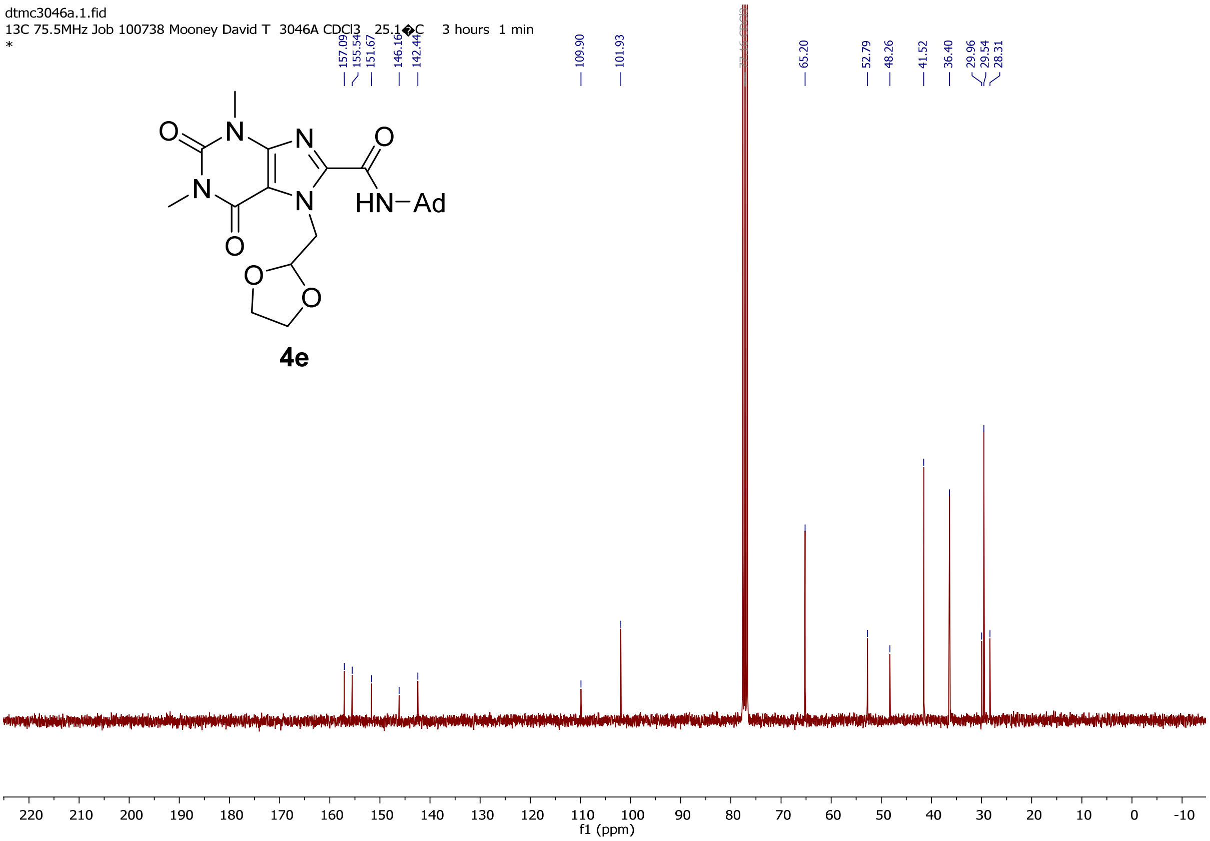
dtmc3046a.1.fid
13C 75.5MHz Job 100738 Mooney David T 3046A CDCl3 25.1�C 3 hours 1 min
*
157.09 155.54 151.67 146.16 142.44
109.90
101.93
65.20
52.79
48.26
41.52
36.40
29.96 29.54 28.31
4e
f1 (ppm)

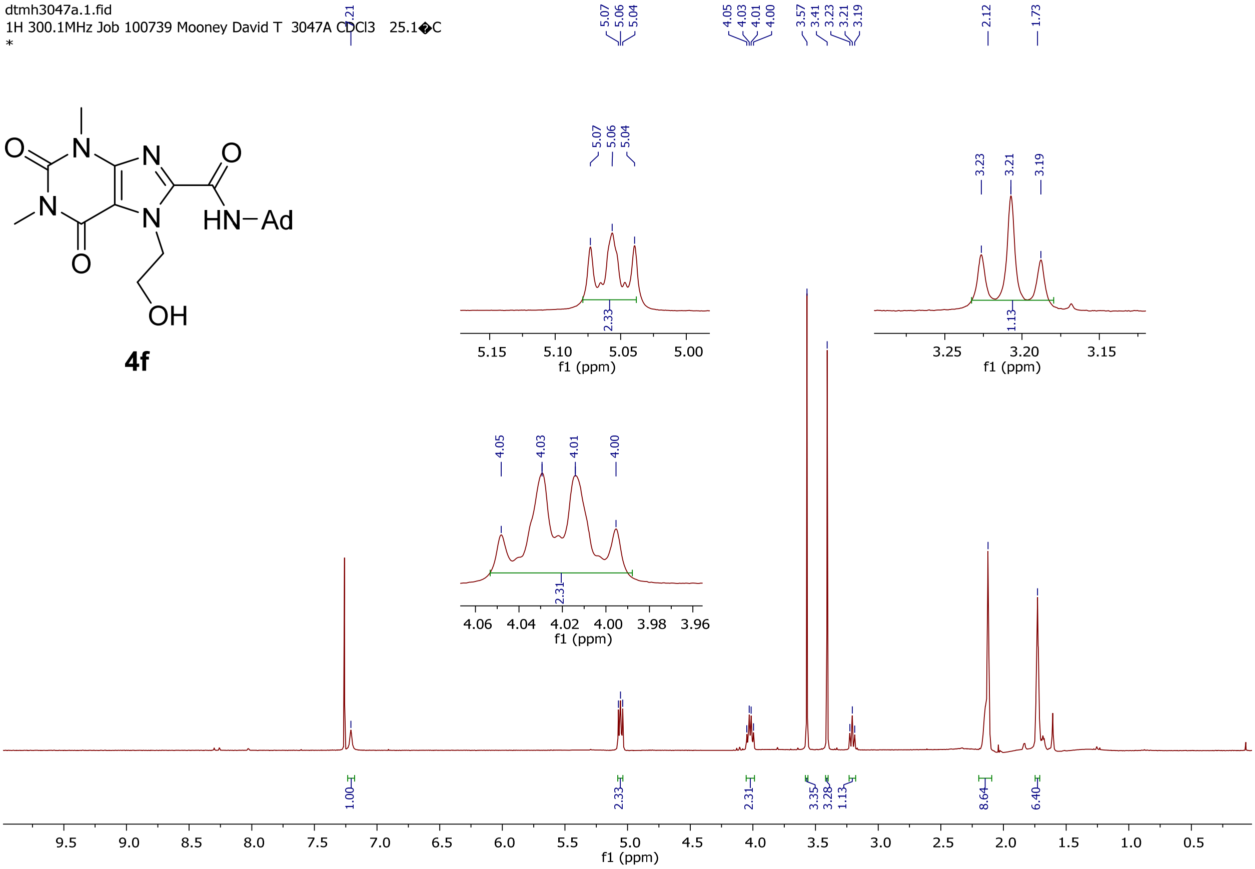
dtmh3047a.1.fid
1H 300.1MHz Job 100739 Mooney David T 3047A CDCl3 25.1�C
*
7.21
5.07
5.06
5.04
4.05
4.03
4.01
4.00
3.57
3.41
3.23
3.21
3.19
2.12
1.73
O
N
N
N
N
O
O
HN−Ad
OH
4f
2.33
1.13
2.31
1.00
2.33
2.31
3.35
3.28
1.13
8.64
6.40
f1 (ppm)

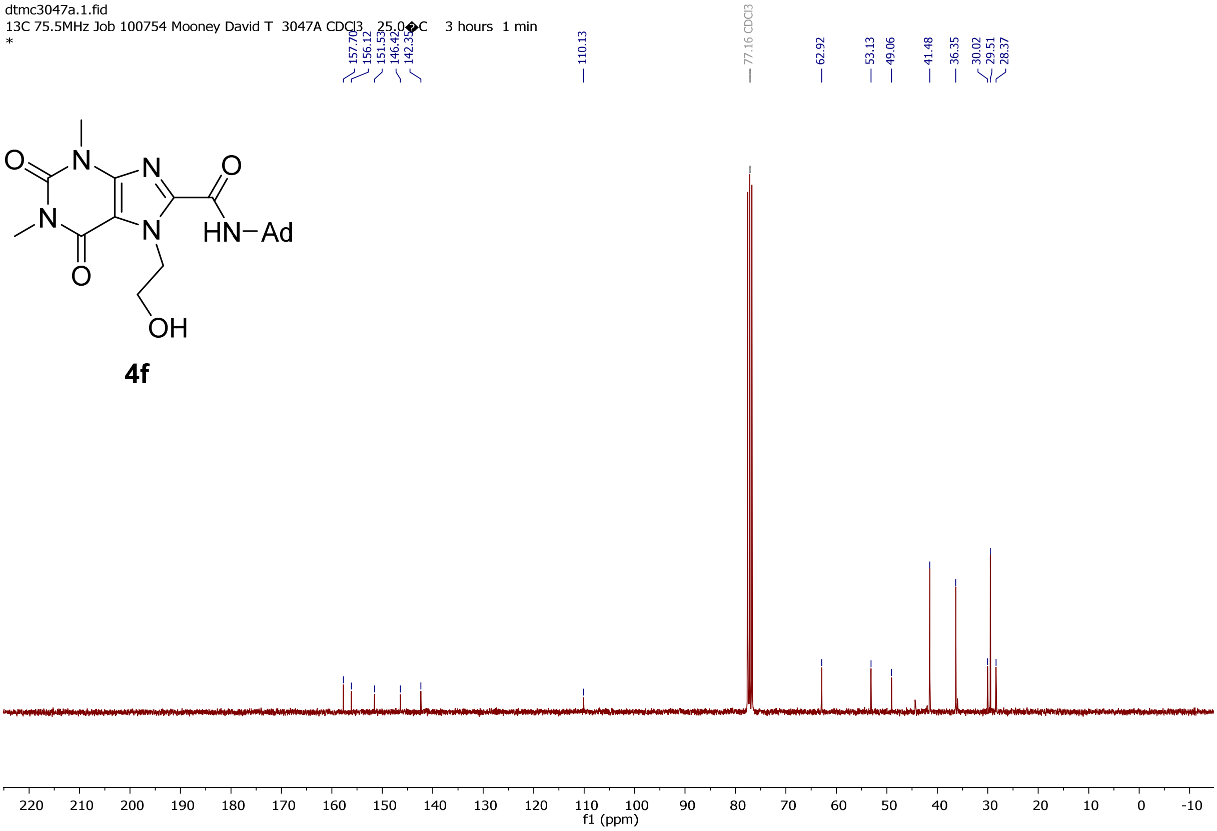
dtmc3047a.1.fid
13C 75.5MHz Job 100754 Mooney David T 3047A CDCl3 25.0�C 3 hours 1 min
*
157.70
156.12
151.53
146.42
142.35
110.13
77.16 CDCl3
62.92
53.13
49.06
41.48
36.35
30.02
29.51
28.37
O
N
N
N
N
O
O
HN−Ad
OH
4f
f1 (ppm)

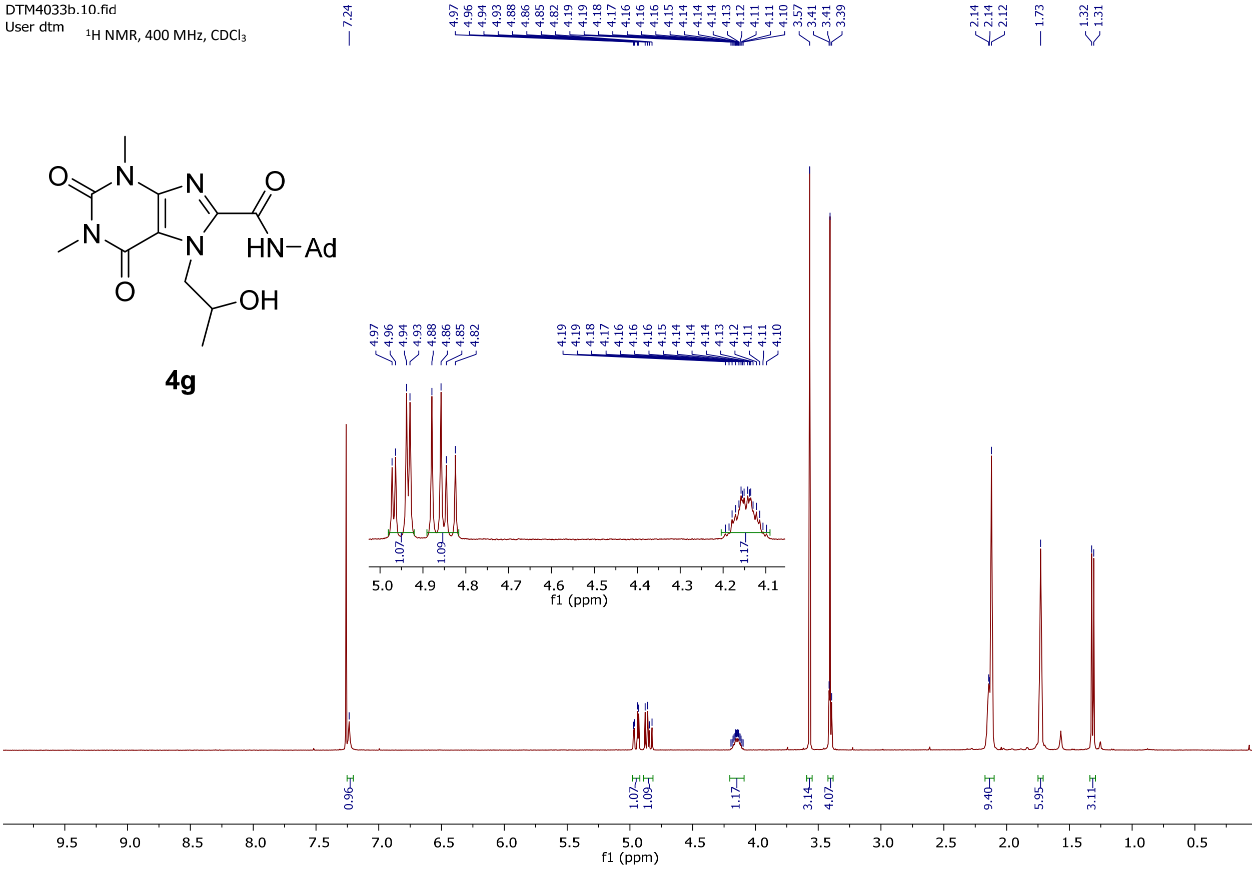
DTM4033b.10.fid
User dtm
¹H NMR, 400 MHz, CDCl₃
4g
HN−Ad
OH
7.24
4.97 4.96 4.94 4.93 4.88 4.86 4.85 4.82 4.19 4.19 4.18 4.17 4.16 4.16 4.16 4.15 4.14 4.14 4.14 4.13 4.12 4.11 4.11 4.10 3.57 3.41 3.41 3.39
2.14 2.14 2.12
1.73
1.32 1.31
0.96 1.07 1.09 1.17 3.14 4.07 9.40 5.95 3.11
f1 (ppm)

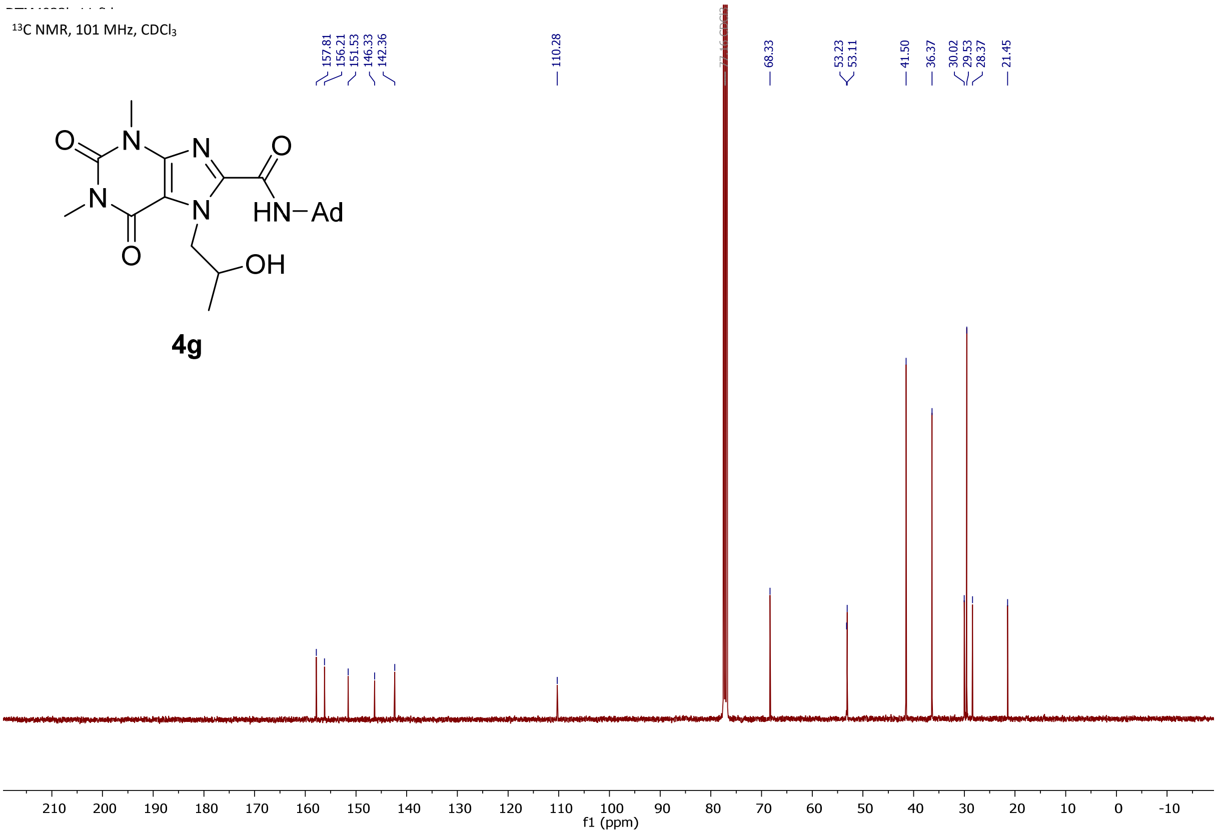
¹³C NMR, 101 MHz, CDCl₃
4g
HN−Ad
OH
157.81 156.21 151.53 146.33 142.36
110.28
68.33
53.23 53.11
41.50
36.37
30.02 29.53 28.37
21.45
f1 (ppm)

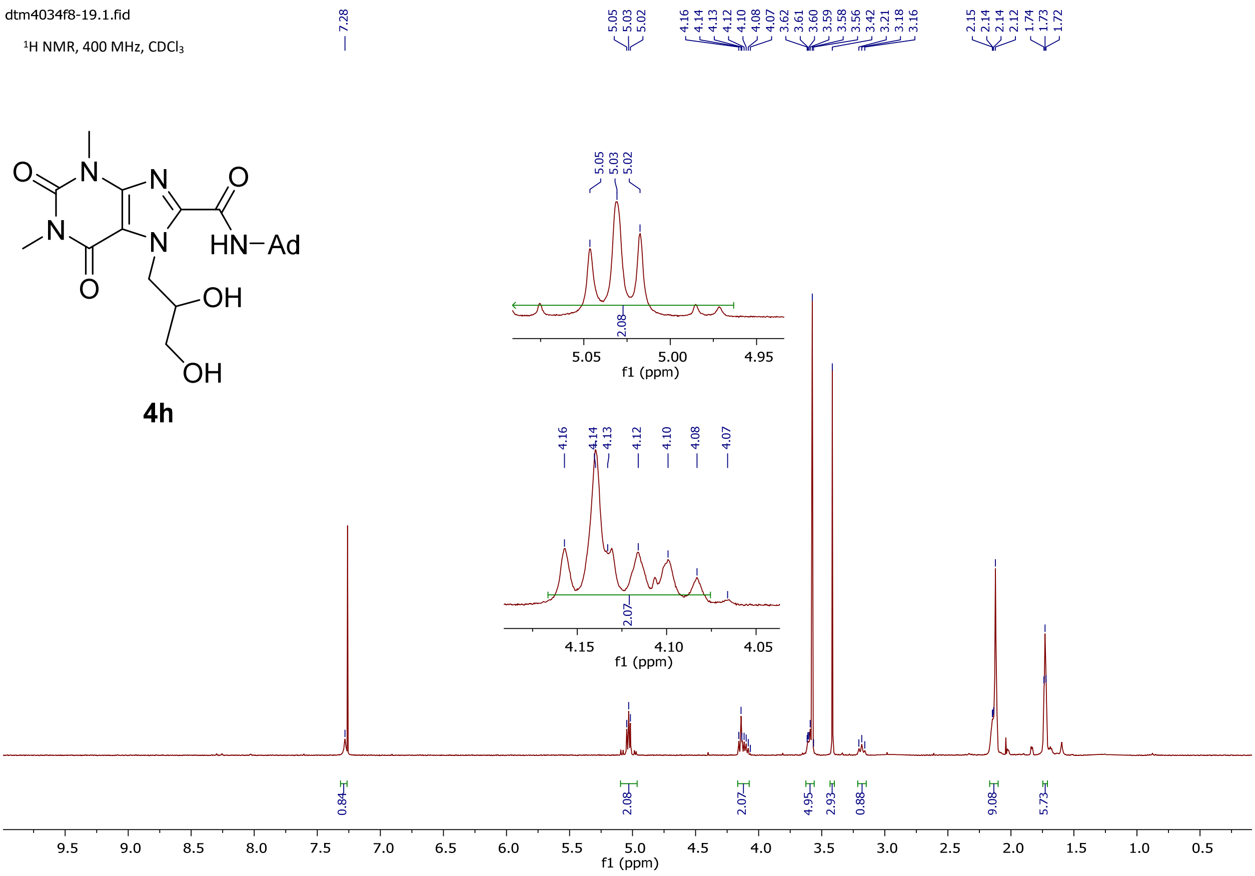
dtm4034f8-19.1.fid
¹H NMR, 400 MHz, CDCl₃
7.28
5.05
5.03
5.02
4.16
4.14
4.13
4.12
4.10
4.08
4.07
3.62
3.61
3.60
3.59
3.58
3.56
3.42
3.21
3.18
3.16
2.15
2.14
2.14
2.12
1.74
1.73
1.72
0.84
2.08
2.07
4.95
2.93
0.88
9.08
5.73
f1 (ppm)
O
N
N
N
N
O
O
HN−Ad
OH
OH
4h

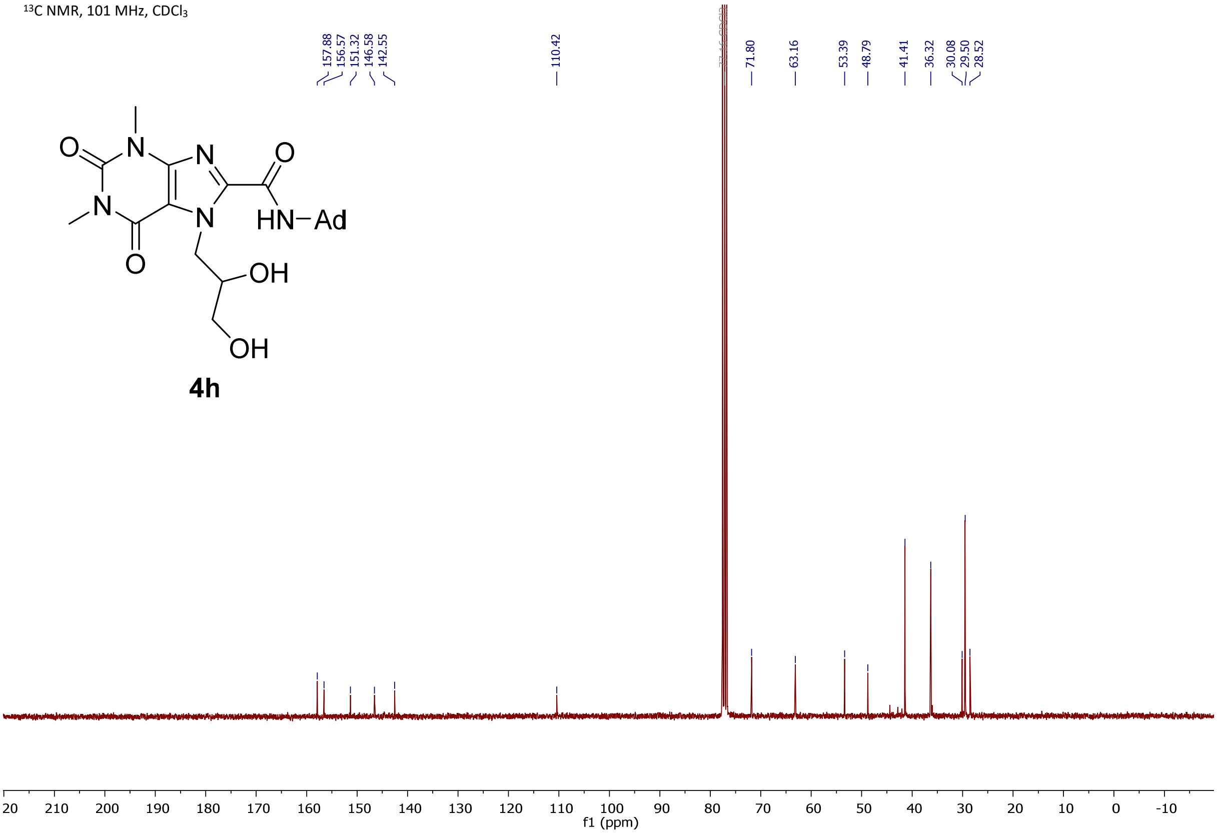
¹³C NMR, 101 MHz, CDCl₃
157.88
156.57
151.32
146.58
142.55
110.42
71.80
63.16
53.39
48.79
41.41
36.32
30.08
29.50
28.52
f1 (ppm)
O
N
N
N
N
O
O
HN−Ad
OH
OH
4h

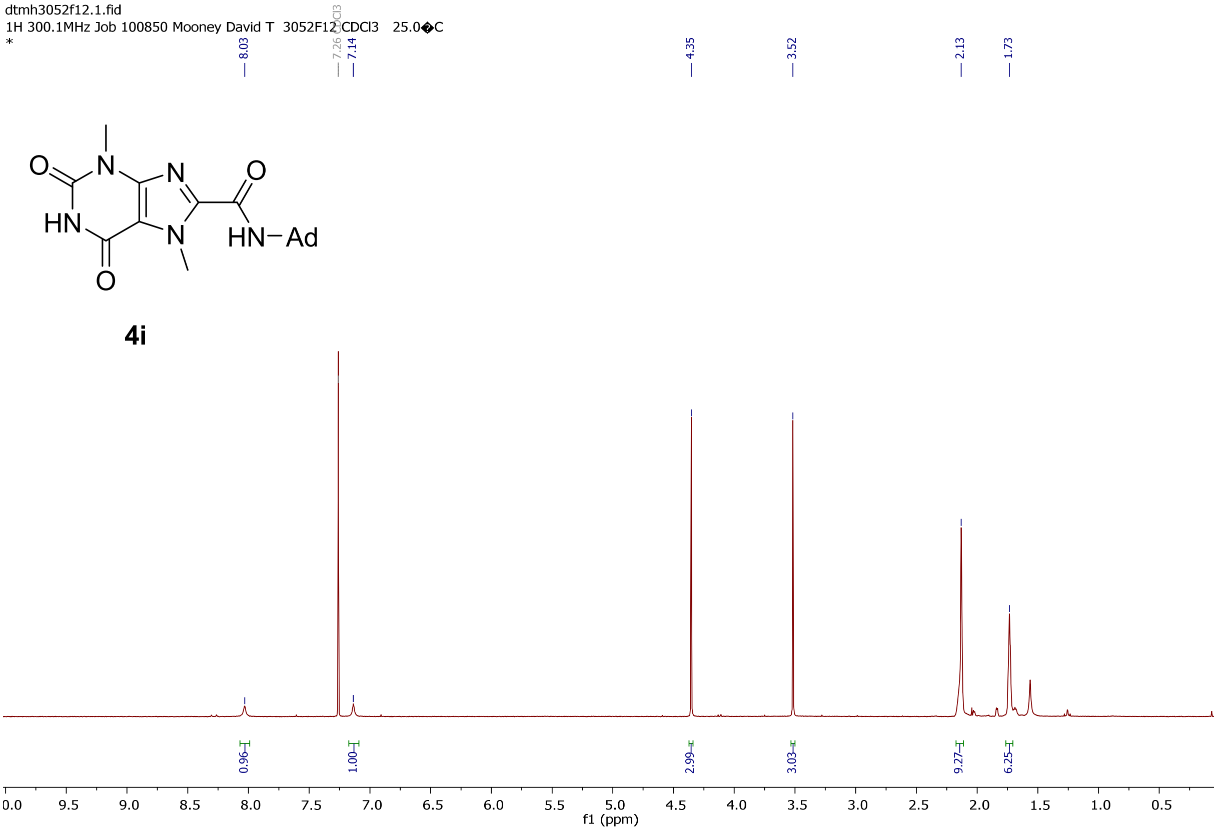
dtmh3052f12.1.fid
1H 300.1MHz Job 100850 Mooney David T 3052F12 CDCl3 25.0�C
*
8.03
7.26 CDCl3
7.14
4.35
3.52
2.13
1.73
4i
HN−Ad
0.96
1.00
2.99
3.03
9.27
6.25
f1 (ppm)

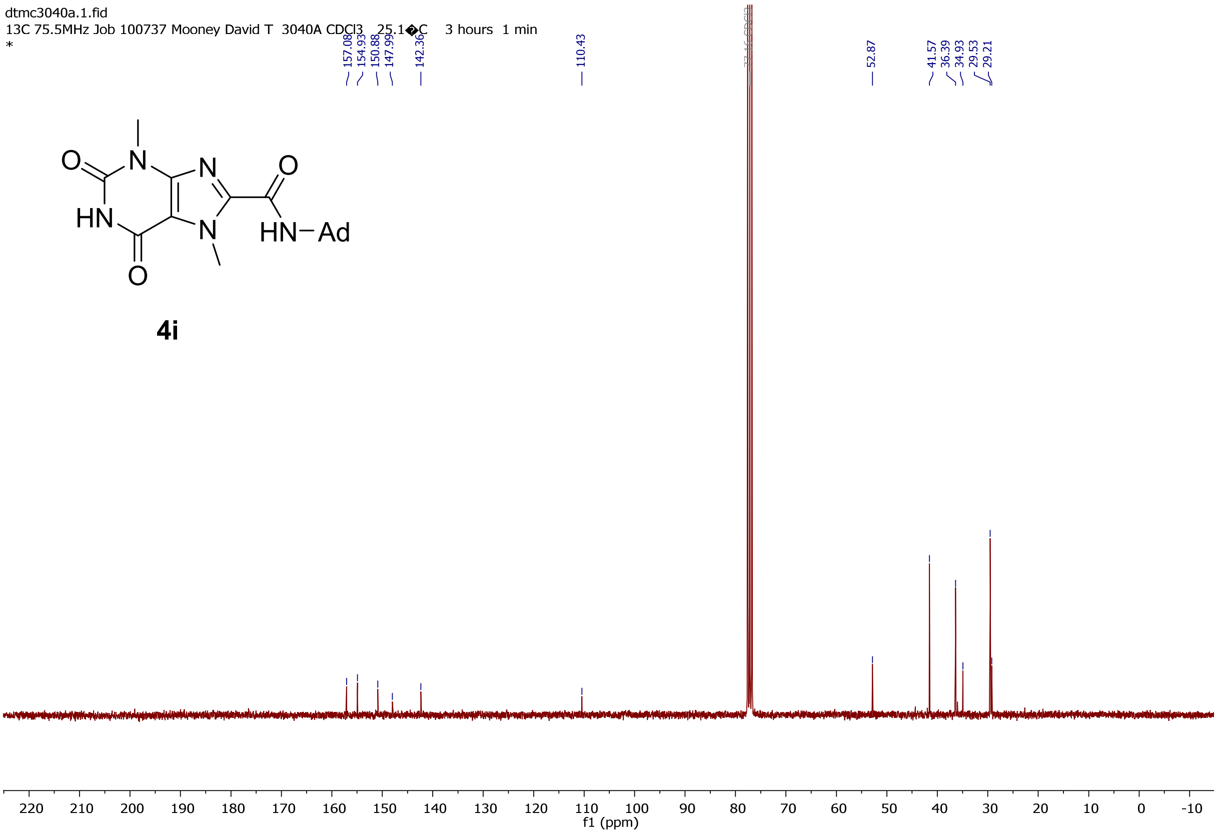
dtmc3040a.1.fid
13C 75.5MHz Job 100737 Mooney David T 3040A CDCl3 25.1�C 3 hours 1 min
*
157.08
154.93
150.88
147.99
142.36
110.43
52.87
41.57
36.39
34.93
29.53
29.21
4i
HN−Ad
f1 (ppm)

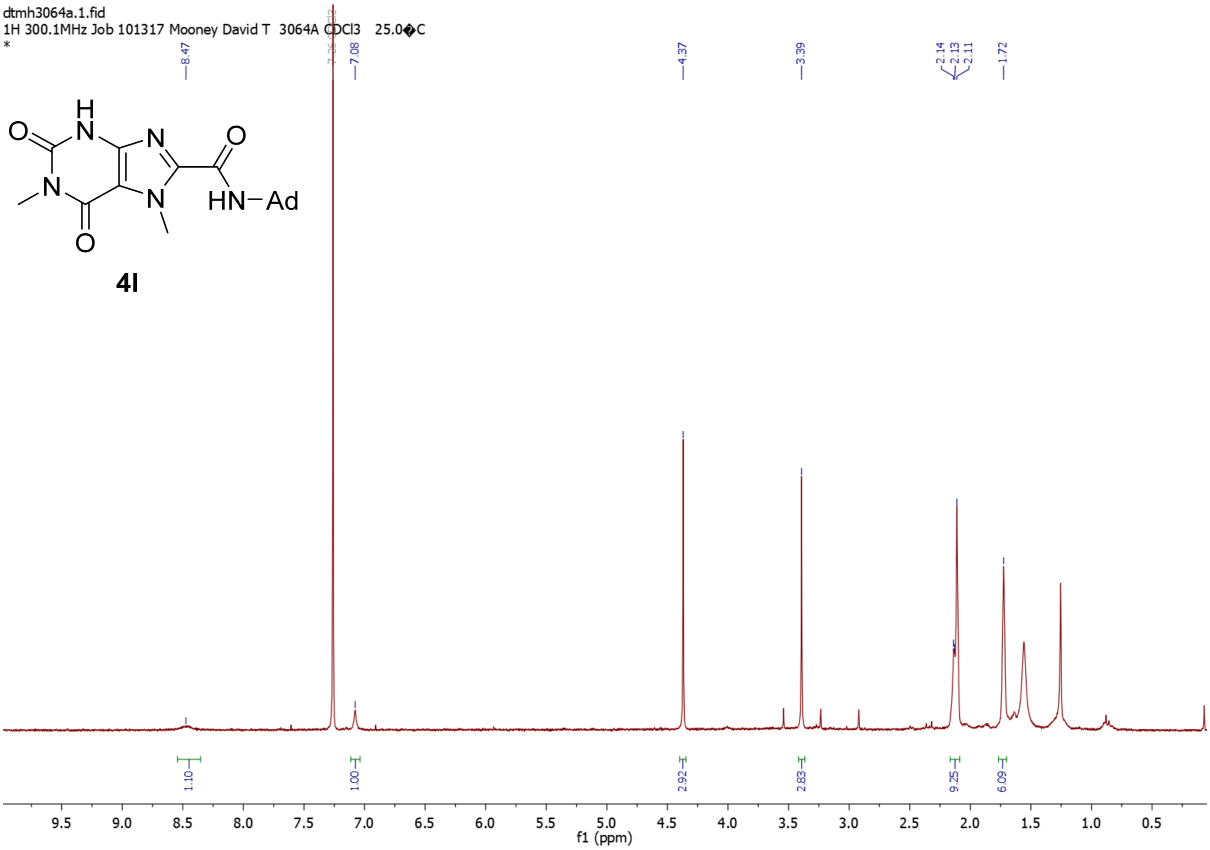
dtmh3064a.1.fid
1H 300.1MHz Job 101317 Mooney David T 3064A CDCl3 25.0�C
*
8.47
7.08
4.37
3.39
2.14
2.13
2.11
1.72
O
H
N
N
O
N
N
HN−Ad
O
4l
1.10
1.00
2.92
2.83
9.25
6.09
9.5
9.0
8.5
8.0
7.5
7.0
6.5
6.0
5.5
5.0
4.5
4.0
3.5
3.0
2.5
2.0
1.5
1.0
0.5
f1 (ppm)

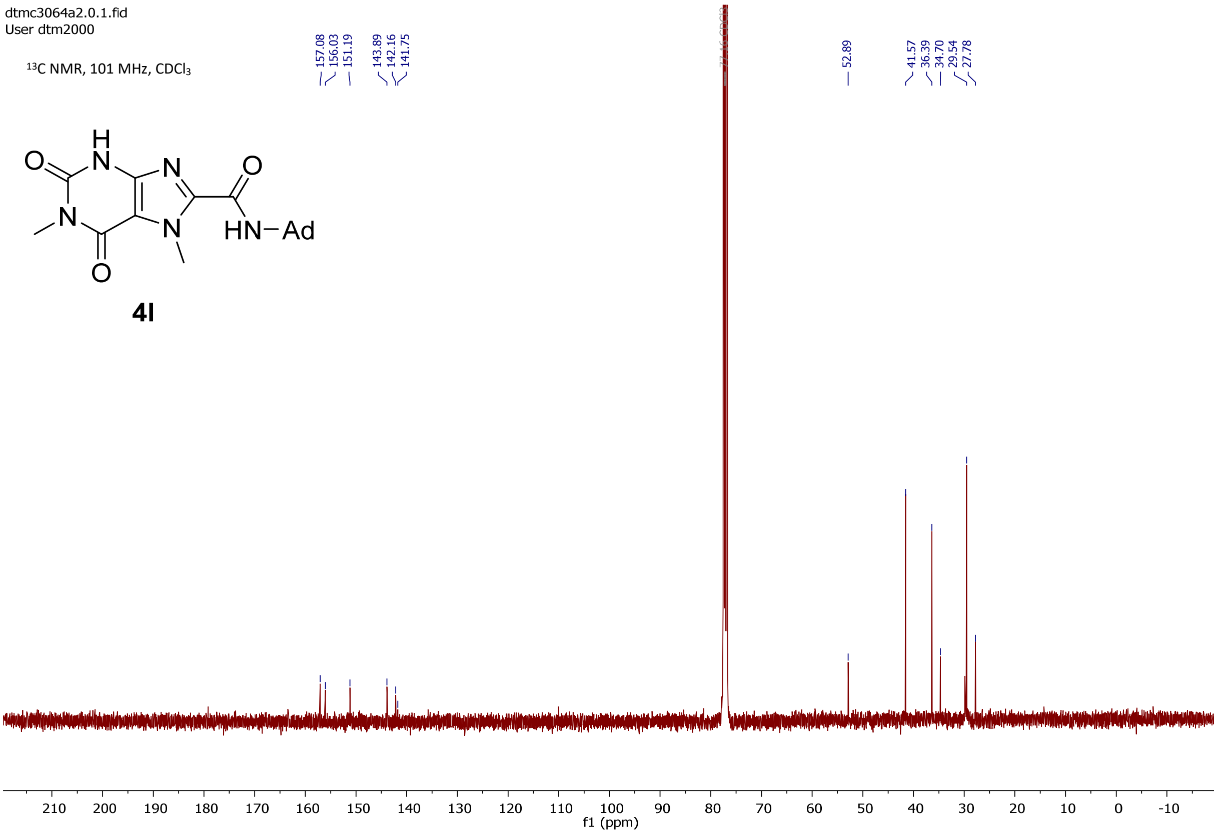
dtmc3064a2.0.1.fid
User dtm2000
13C NMR, 101 MHz, CDCl3
157.08
156.03
151.19
143.89
142.16
141.75
52.89
41.57
36.39
34.70
29.54
27.78
O
H
N
N
O
N
N
HN−Ad
O
4l
210
200
190
180
170
160
150
140
130
120
110
100
90
80
70
60
50
40
30
20
10
0
-10
f1 (ppm)

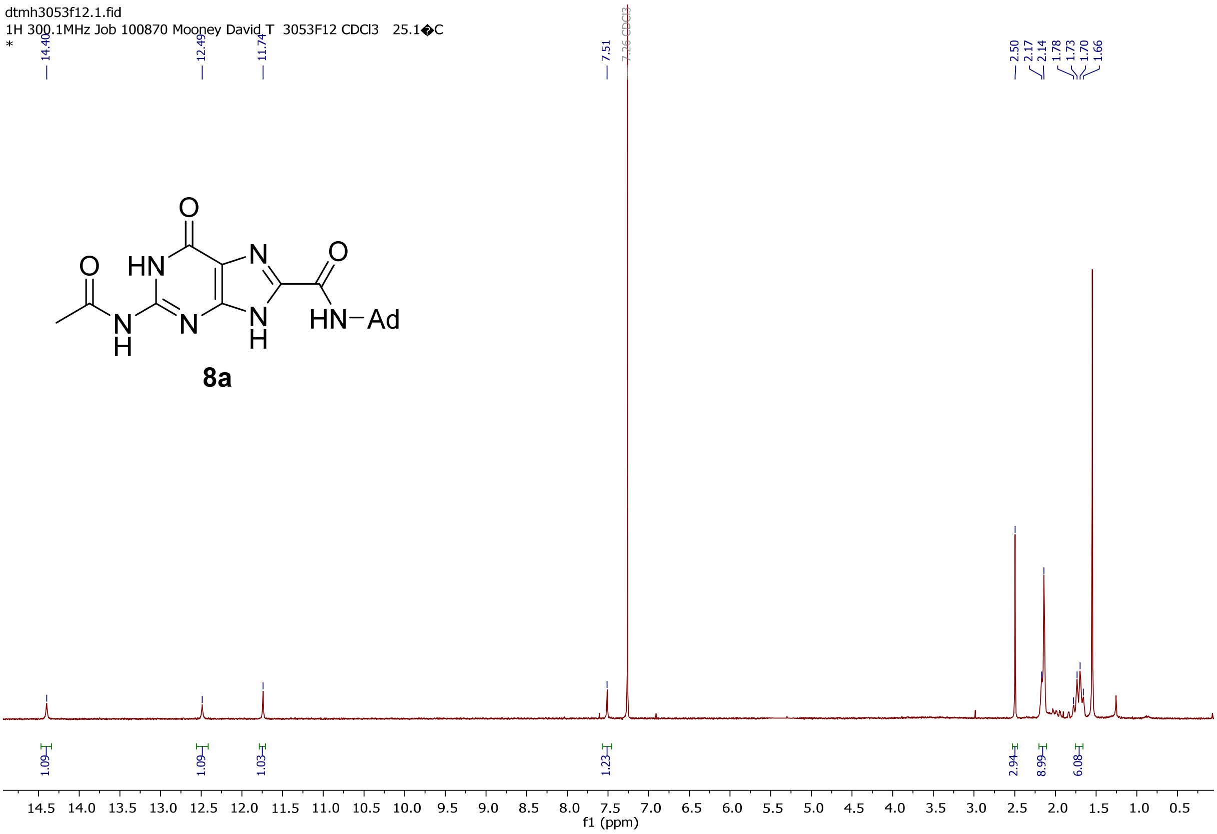
dtmh3053f12.1.fid
1H 300.1MHz Job 100870 Mooney David T 3053F12 CDCl3 25.1�C
*
14.40
12.49
11.74
7.51
7.26 CDCl3
2.50
2.17
2.14
1.78
1.73
1.70
1.66
O
HN
N
O
N
N
H
N
H
HN−Ad
8a
1.09
1.09
1.03
1.23
2.94
8.99
6.08
f1 (ppm)

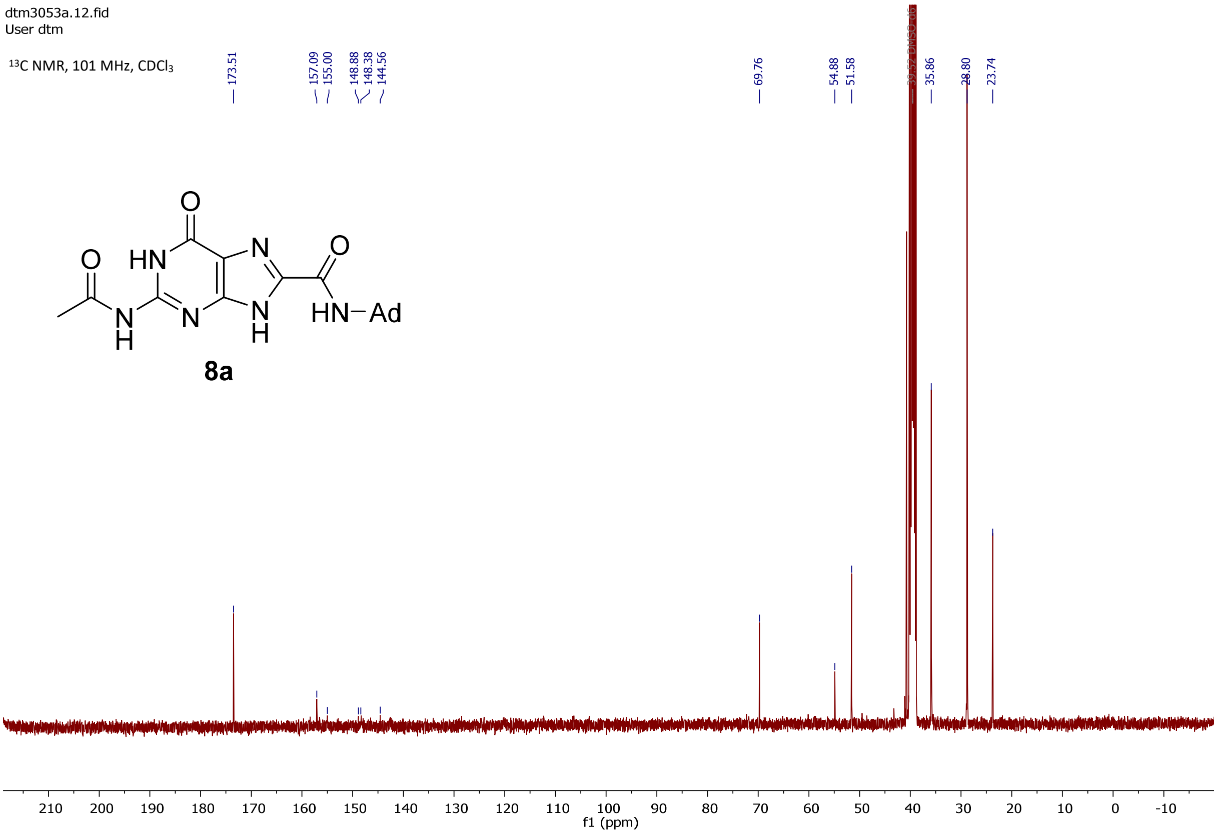
dtm3053a.12.fid
User dtm
13C NMR, 101 MHz, CDCl3
173.51
157.09
155.00
148.88
148.38
144.56
69.76
54.88
51.58
35.86
28.80
23.74
O
HN
N
O
N
N
H
N
H
HN−Ad
8a
f1 (ppm)

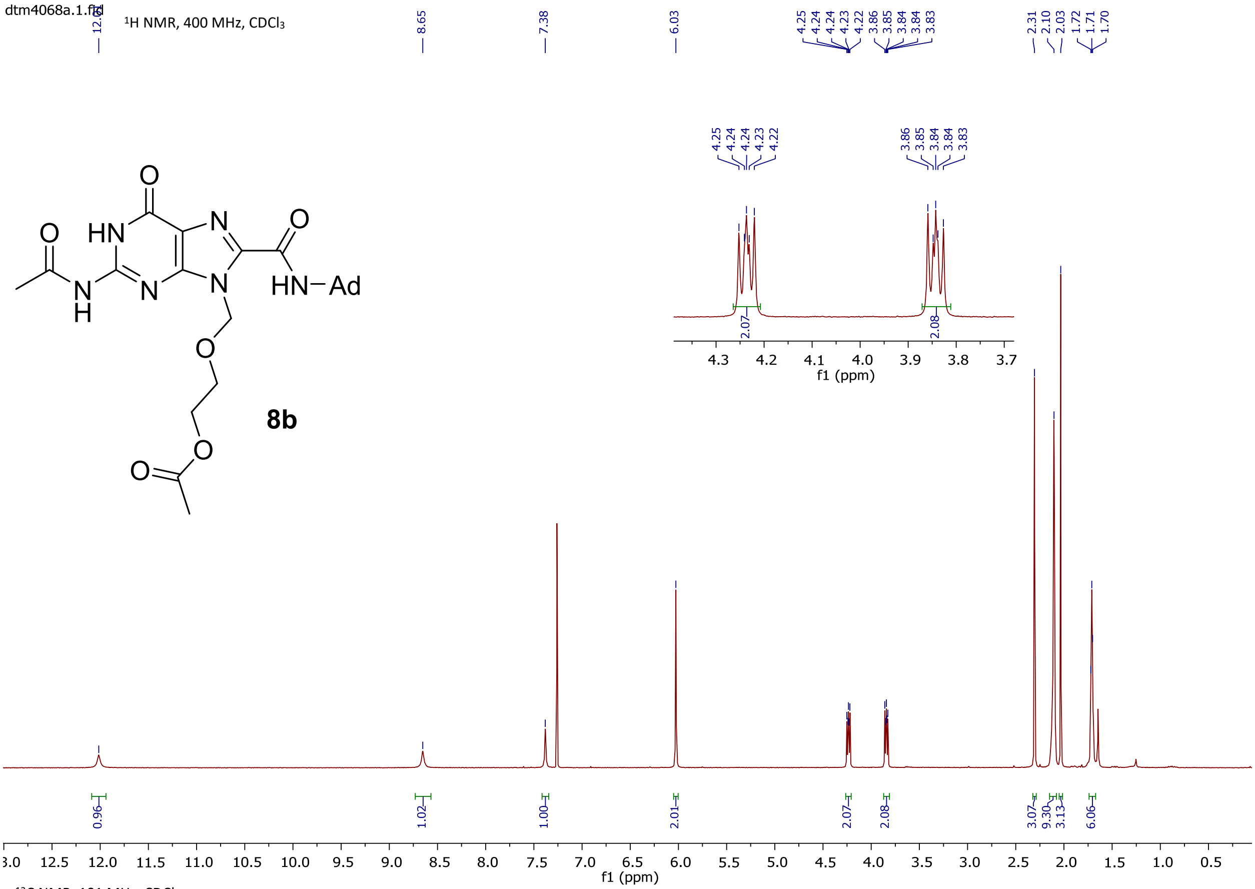
dtm4068a.1.fid
¹H NMR, 400 MHz, CDCl₃
12.04
8.65
7.38
6.03
4.25 4.24 4.24 4.23 4.22 3.85 3.85 3.84 3.84 3.83
2.31 2.10 2.03 1.72 1.71 1.70
4.25 4.24 4.24 4.23 4.22
3.86 3.85 3.84 3.84 3.83
2.07
2.08
4.3 4.2 4.1 4.0 3.9 3.8 3.7
f1 (ppm)
O
HN
N
O
N
N
N
H
HN−Ad
O
O
O
8b
0.96
1.02
1.00
2.01
2.07
2.08
3.07
9.30
3.13
6.06
13.0 12.5 12.0 11.5 11.0 10.5 10.0 9.5 9.0 8.5 8.0 7.5 7.0 6.5 6.0 5.5 5.0 4.5 4.0 3.5 3.0 2.5 2.0 1.5 1.0 0.5
f1 (ppm)

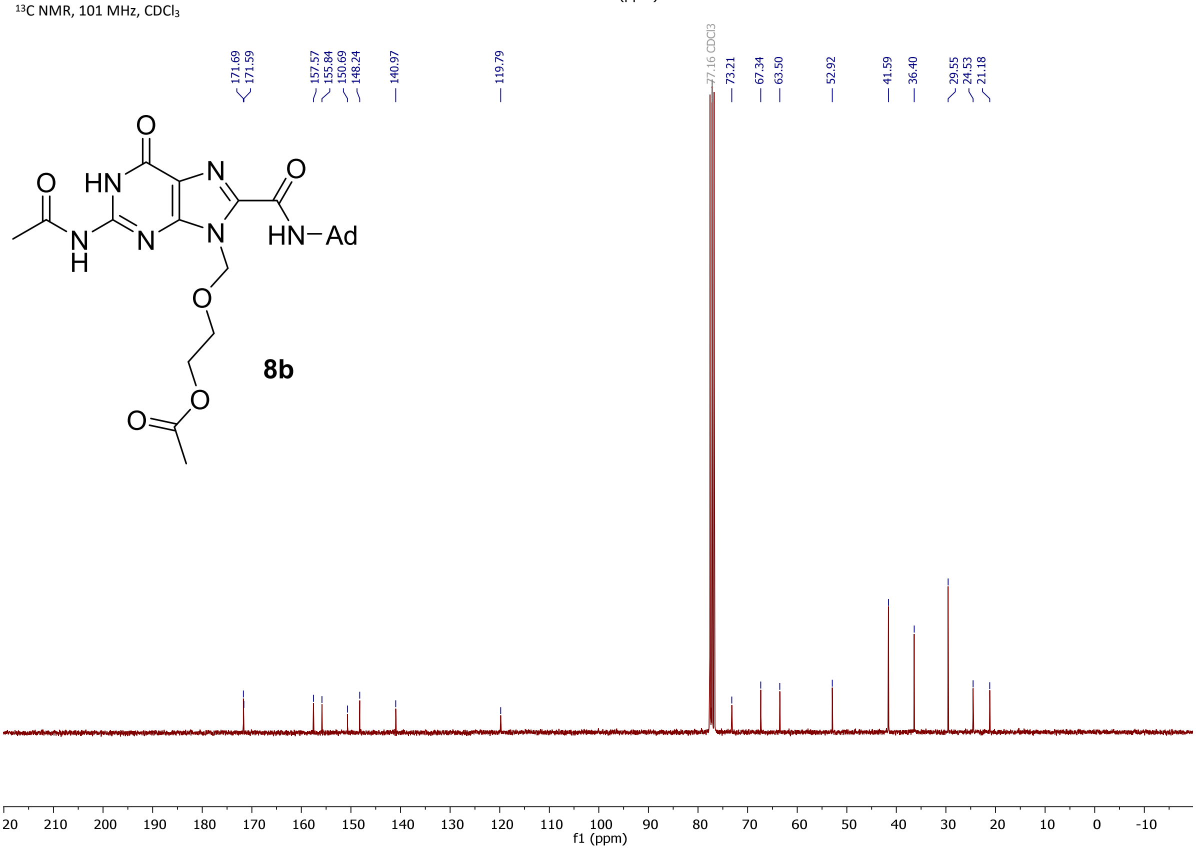
¹³C NMR, 101 MHz, CDCl₃
171.69
171.59
157.57
155.84
150.69
148.24
140.97
119.79
77.16 CDCl3
73.21
67.34
63.50
52.92
41.59
36.40
29.55
24.53
21.18
O
HN
N
O
N
N
N
H
HN−Ad
O
O
O
8b
220 210 200 190 180 170 160 150 140 130 120 110 100 90 80 70 60 50 40 30 20 10 0 -10
f1 (ppm)

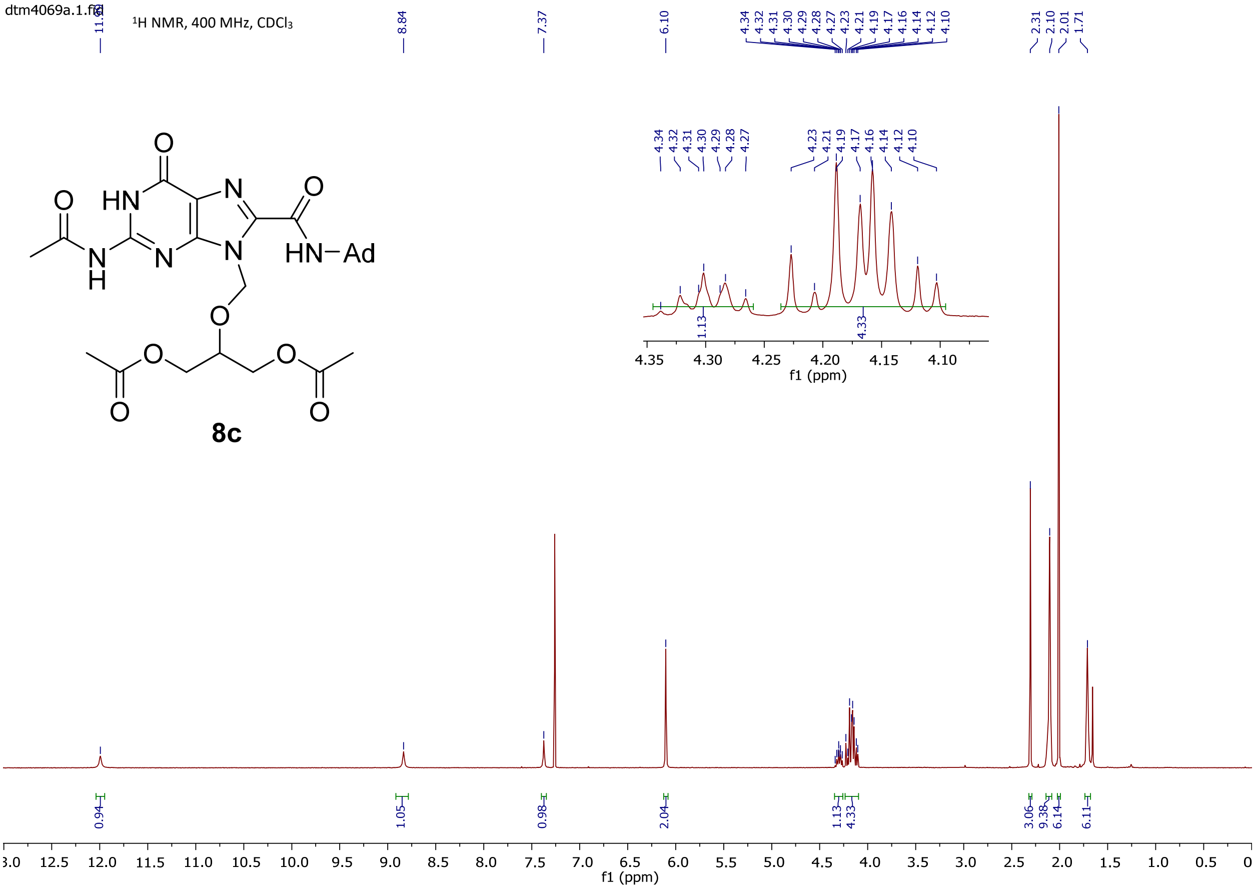
dtm4069a.1.fid
^1H NMR, 400 MHz, $CDCl_3$
11.61
8.84
7.37
6.10
4.34 4.32 4.31 4.30 4.29 4.28 4.27 4.23 4.21 4.19 4.17 4.16 4.14 4.12 4.10
2.31 2.10 2.01 1.71
8c
1.13
4.33
0.94
1.05
0.98
2.04
3.06 9.38 6.14
6.11
f1 (ppm)

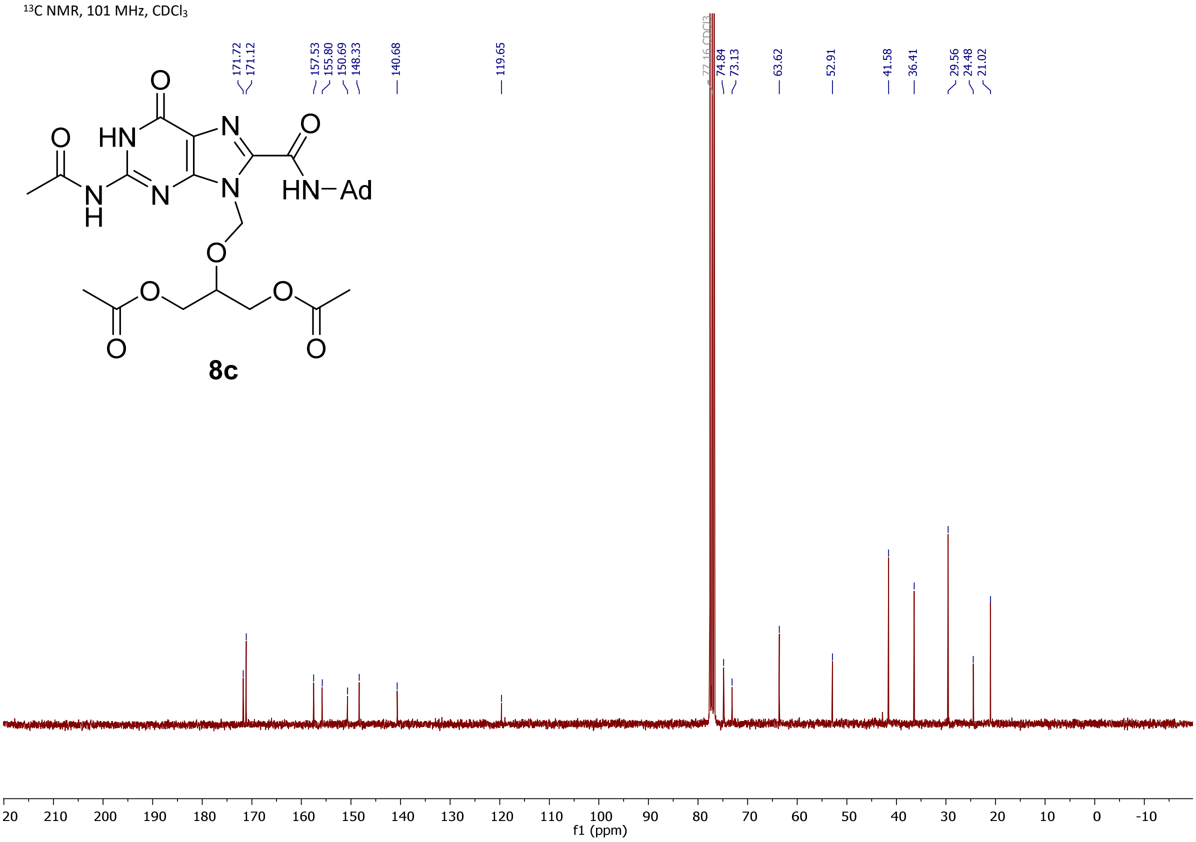
^{13}C NMR, 101 MHz, $CDCl_3$
171.72 171.12
157.53 155.80 150.69 148.33
140.68
119.65
77.16 CDCl3
74.84 73.13
63.62
52.91
41.58
36.41
29.56 24.48 21.02
8c
f1 (ppm)

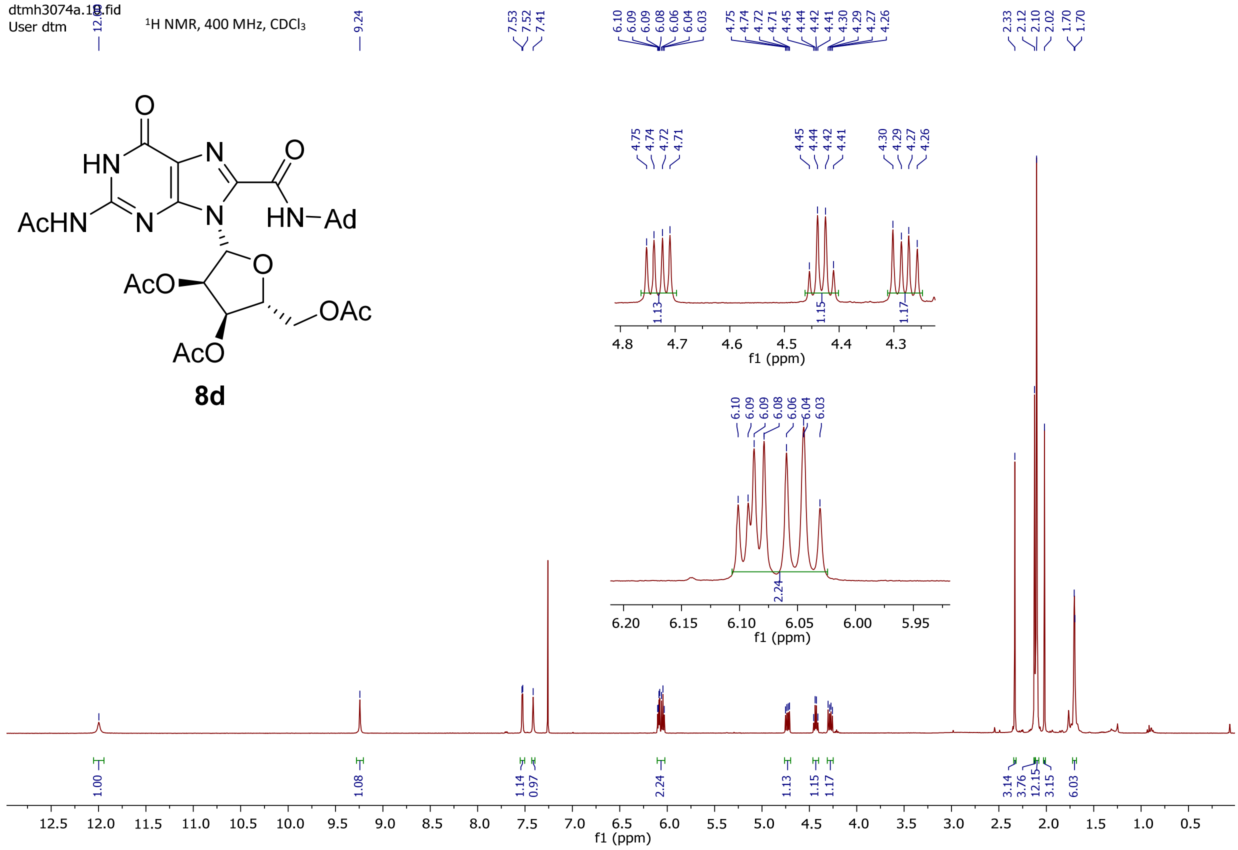
User dtm
¹H NMR, 400 MHz, CDCl₃
8d
12.00, 9.24, 7.53, 7.52, 7.41, 6.10, 6.09, 6.09, 6.08, 6.06, 6.04, 6.03, 4.75, 4.74, 4.72, 4.71, 4.45, 4.44, 4.42, 4.41, 4.30, 4.29, 4.27, 4.26, 2.33, 2.12, 2.10, 2.02, 1.70, 1.70
1.00, 1.08, 1.14, 0.97, 2.24, 1.13, 1.15, 1.17, 3.14, 3.76, 12.15, 3.15, 6.03
f1 (ppm)

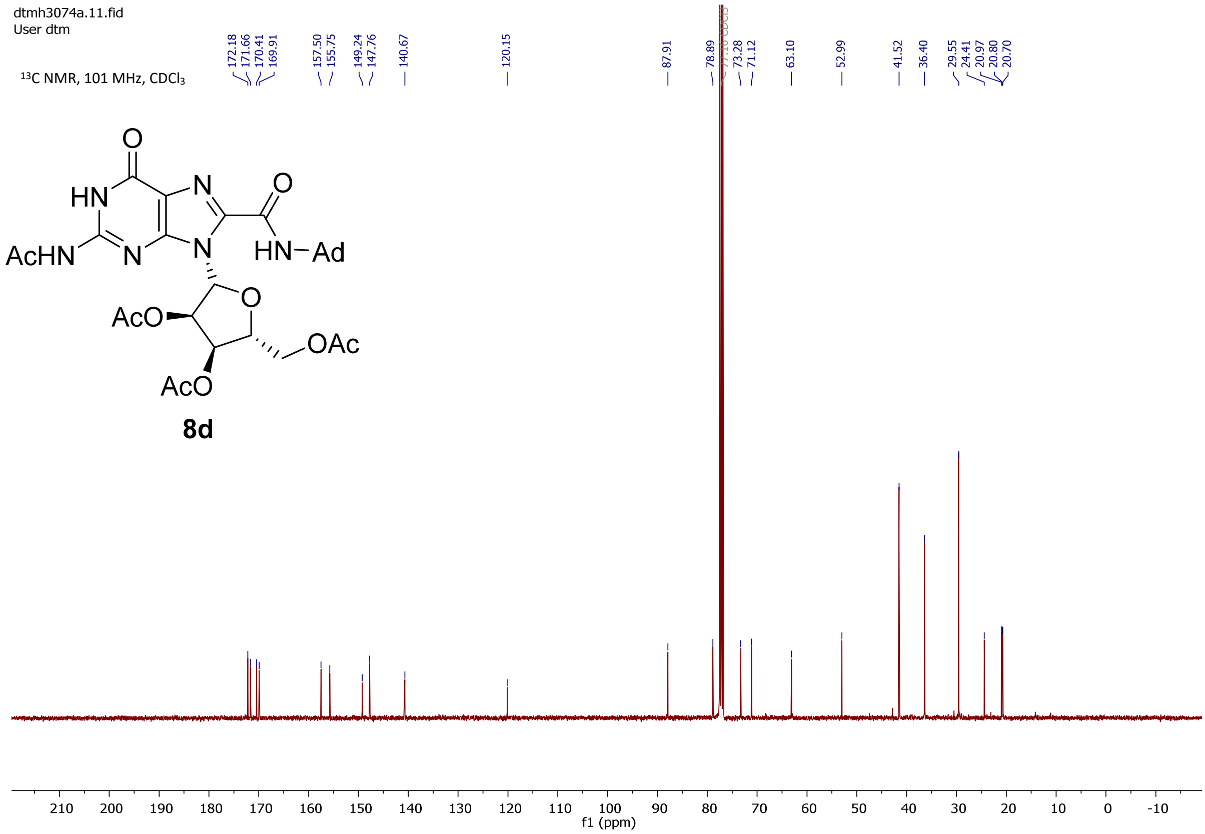
dtmh3074a.11.fid
User dtm
¹³C NMR, 101 MHz, CDCl₃
8d
172.18, 171.66, 170.41, 169.91, 157.50, 155.75, 149.24, 147.76, 140.67, 120.15, 87.91, 78.89, 73.28, 71.12, 63.10, 52.99, 41.52, 36.40, 29.55, 24.41, 20.97, 20.80, 20.70
f1 (ppm)

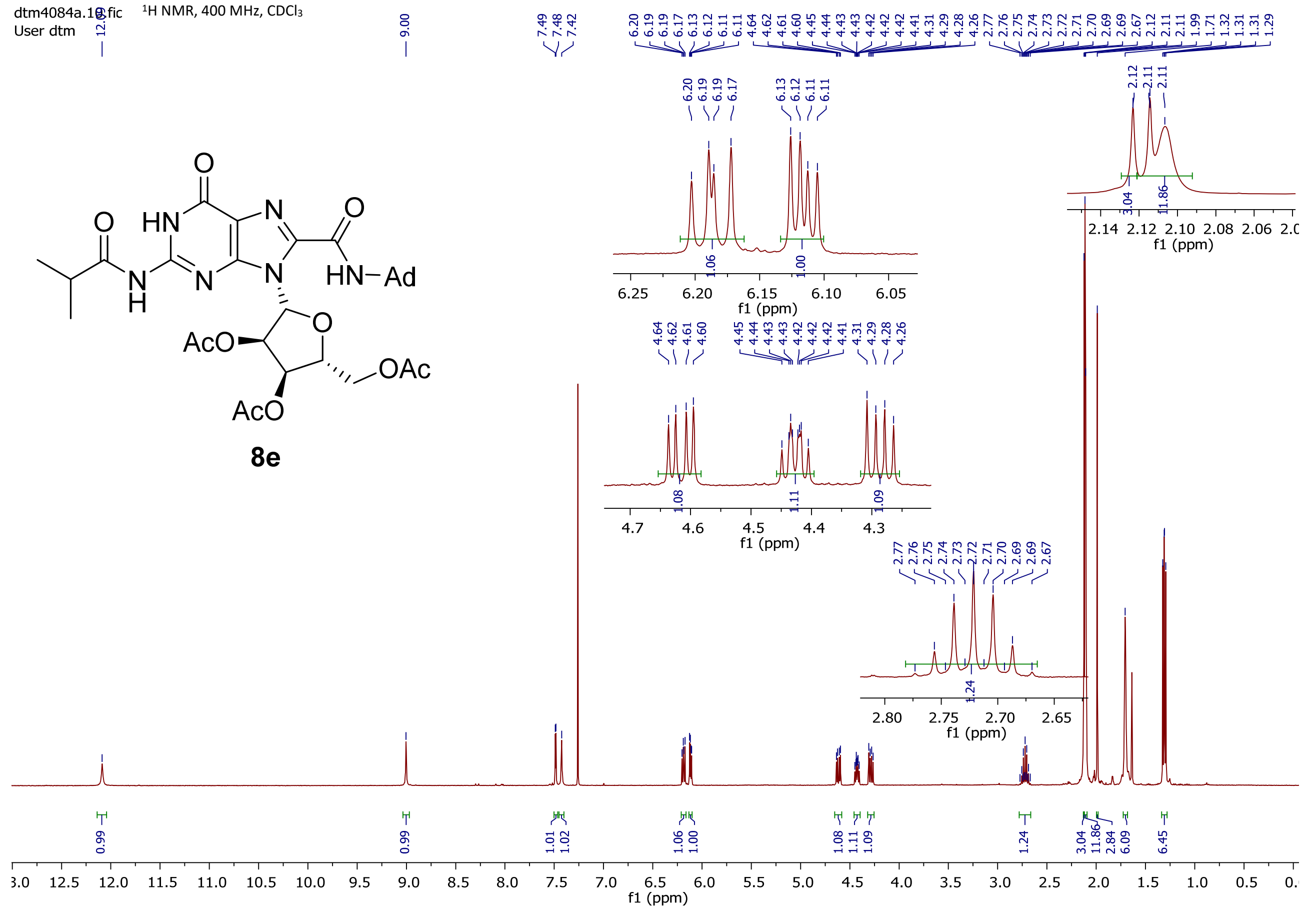
dtm4084a.10.fic
User dtm
¹H NMR, 400 MHz, CDCl₃
8e
f1 (ppm)

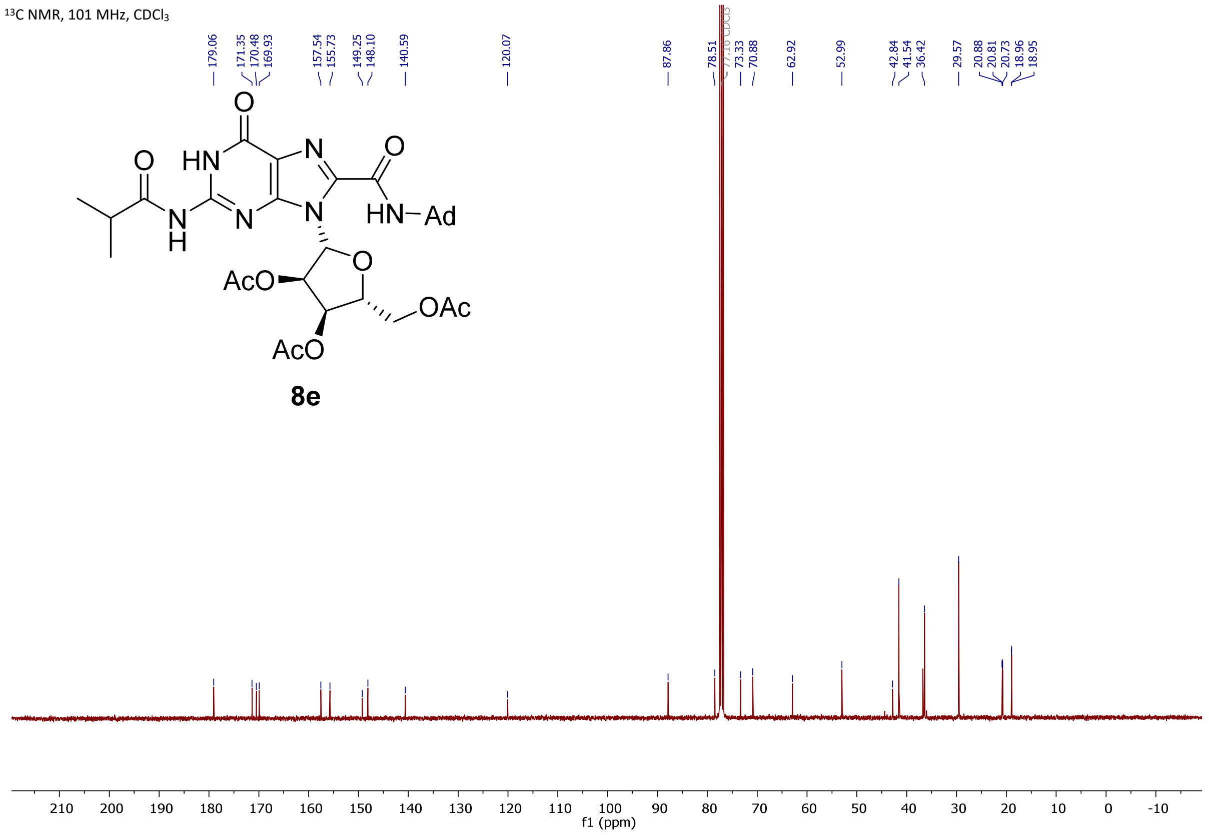
¹³C NMR, 101 MHz, CDCl₃
8e
f1 (ppm)

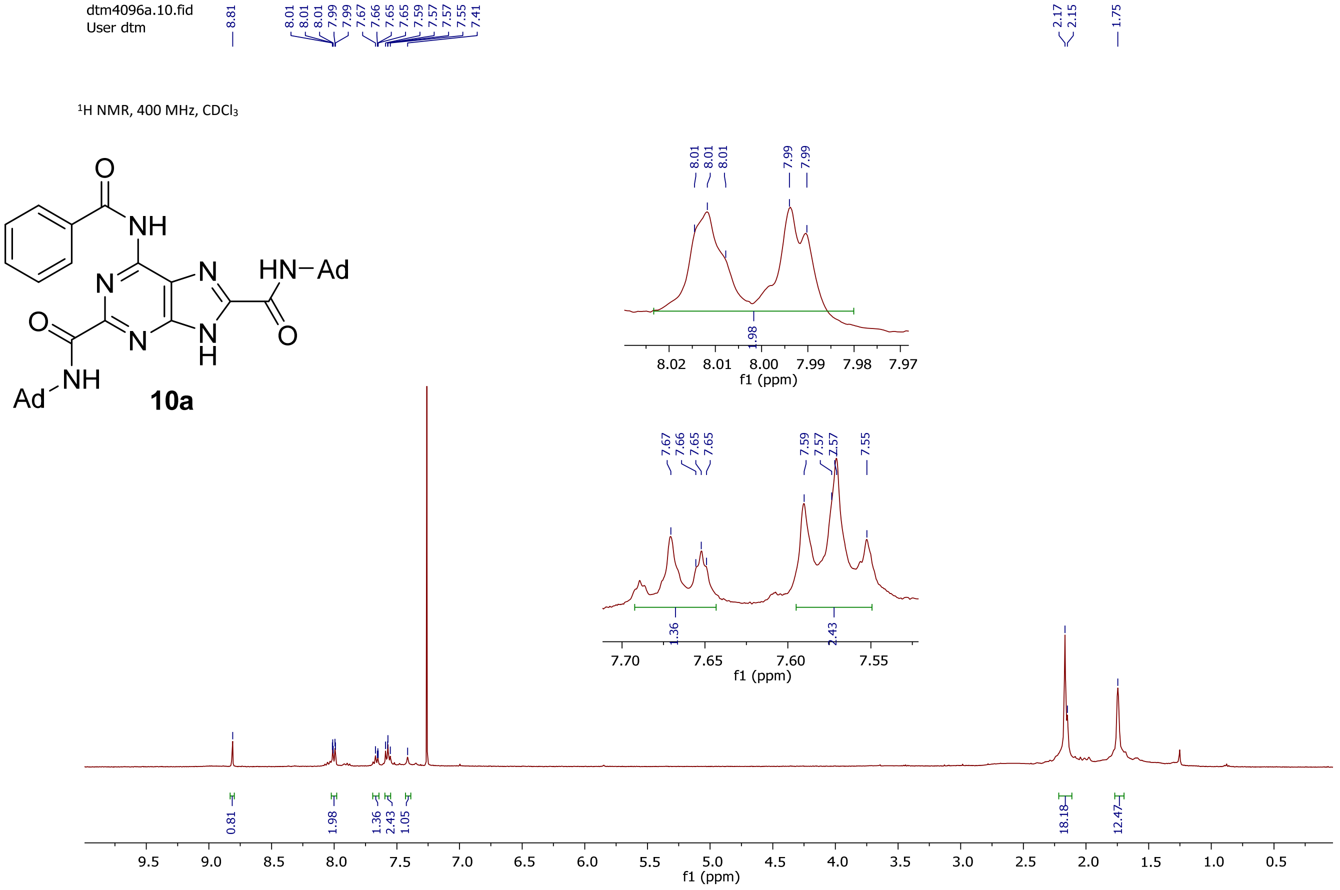
dtm4096a.10.fid
User dtm
¹H NMR, 400 MHz, CDCl₃
10a
HN−Ad
Ad
NH
f1 (ppm)

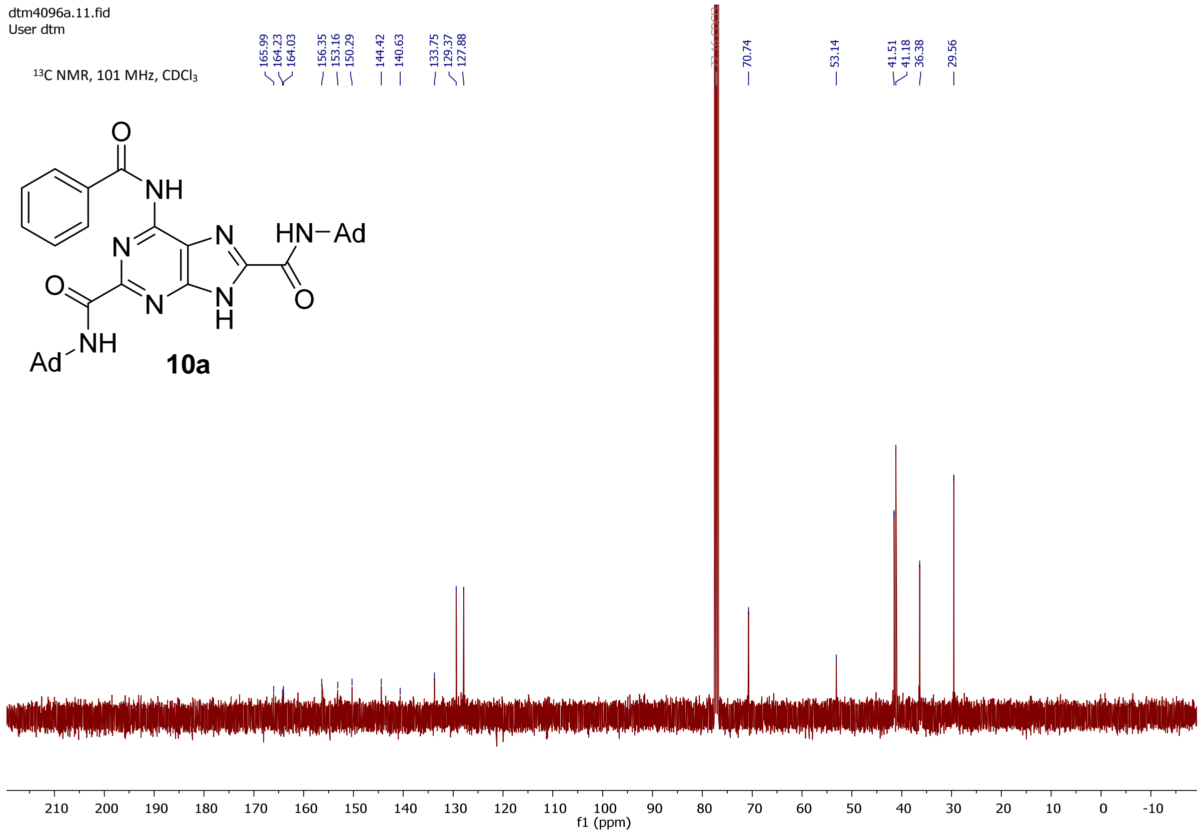
dtm4096a.11.fid
User dtm
¹³C NMR, 101 MHz, CDCl₃
165.99
164.23
164.03
156.35
153.16
150.29
144.42
140.63
133.75
129.37
127.88
70.74
53.14
41.51
41.18
36.38
29.56
10a
f1 (ppm)

dtm3099a.1.fid

¹H NMR, 400 MHz, CDCl₃

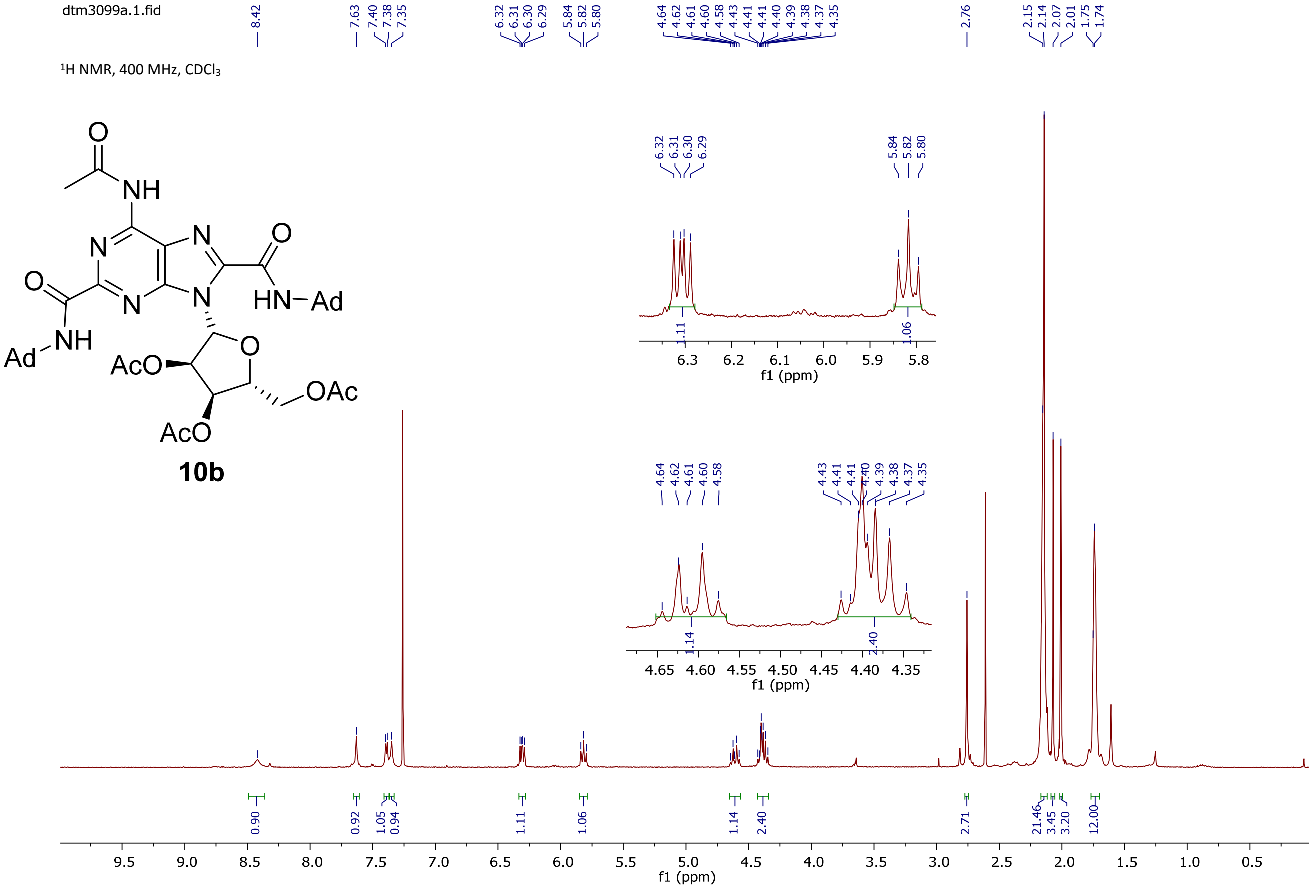

¹³C NMR, 101 MHz, CDCl₃

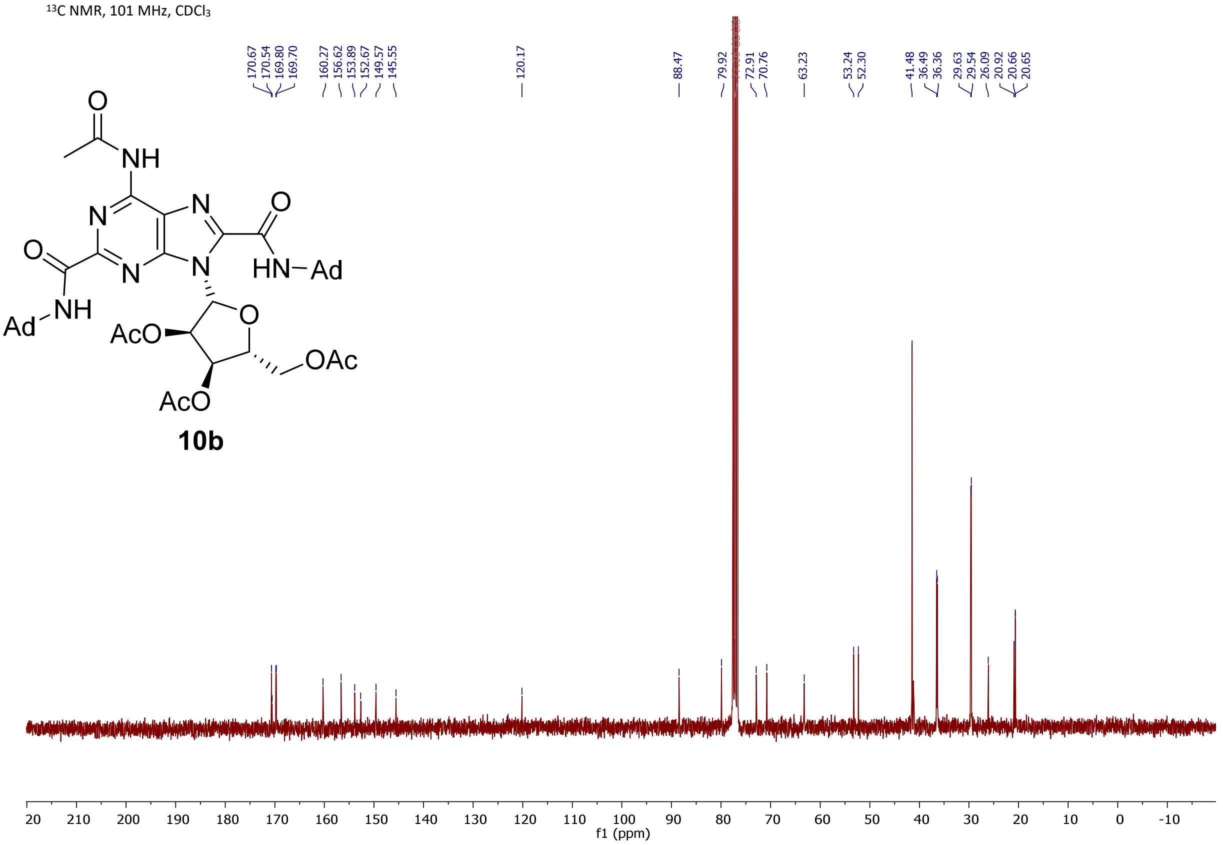

dtm4088a.10.fid
User dtm

^{1}H NMR, 400 MHz, $CDCl_3$

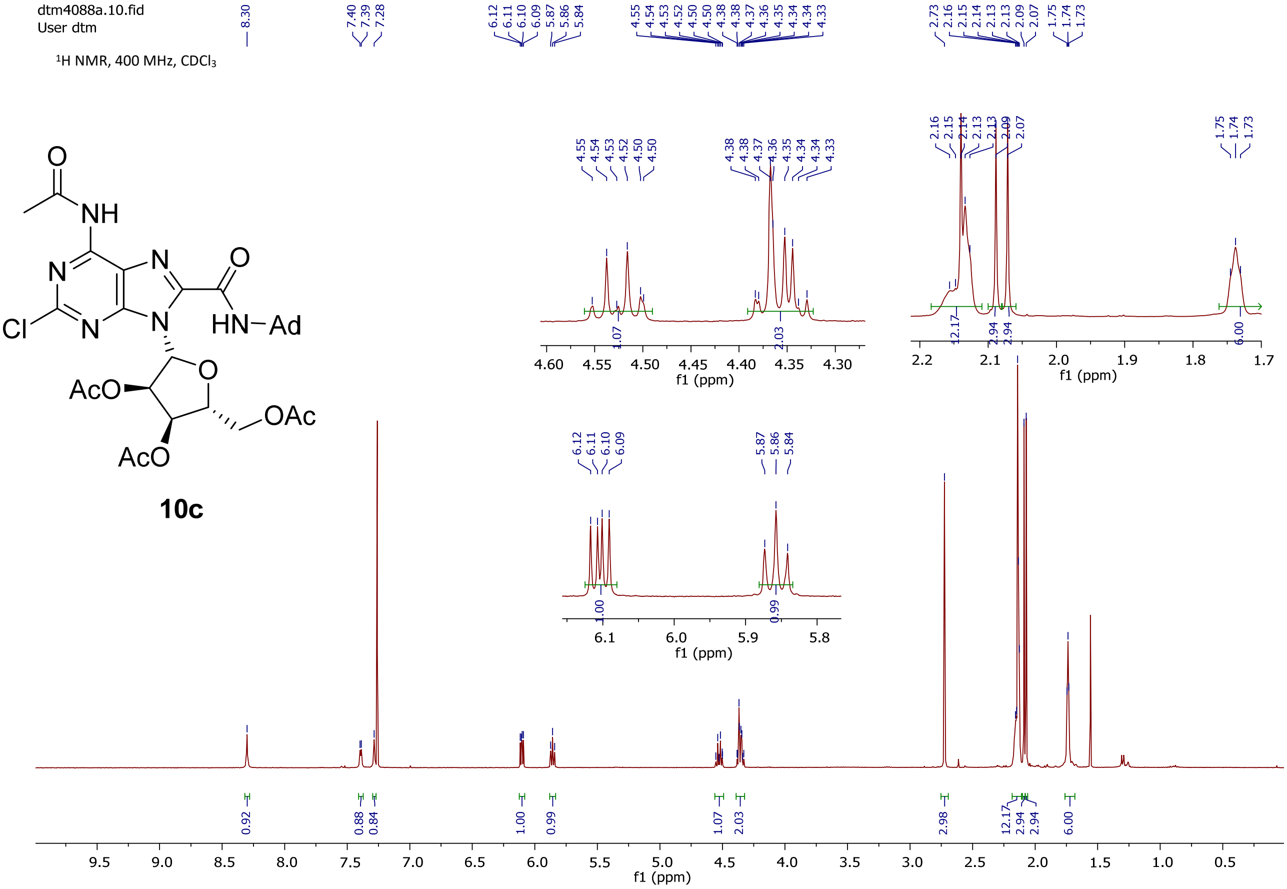

10c

^{13}C NMR, 101 MHz, $CDCl_3$

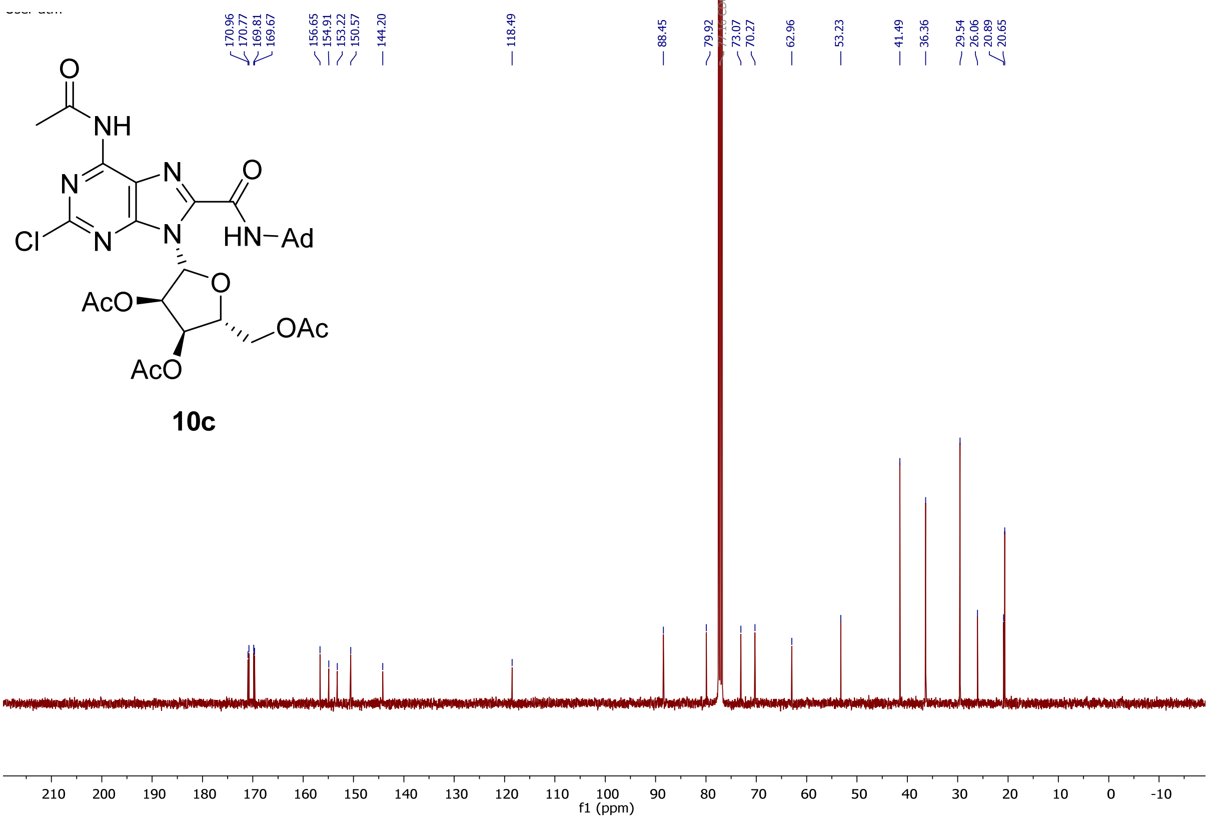

10c

dtm5013a.1.fid
User dtm2000

^{1}H NMR, 400 MHz, $CDCl_3$

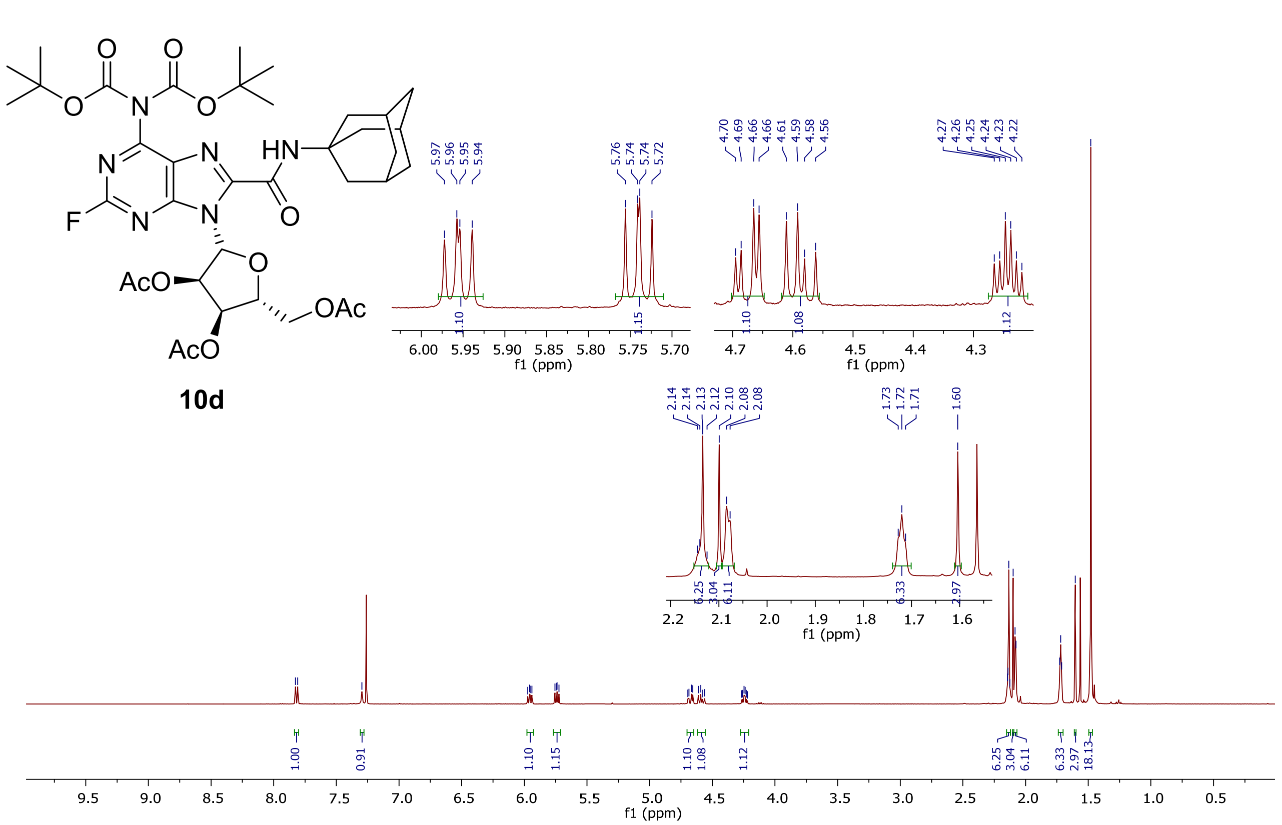

^{13}C NMR, 101 MHz, $CDCl_3$

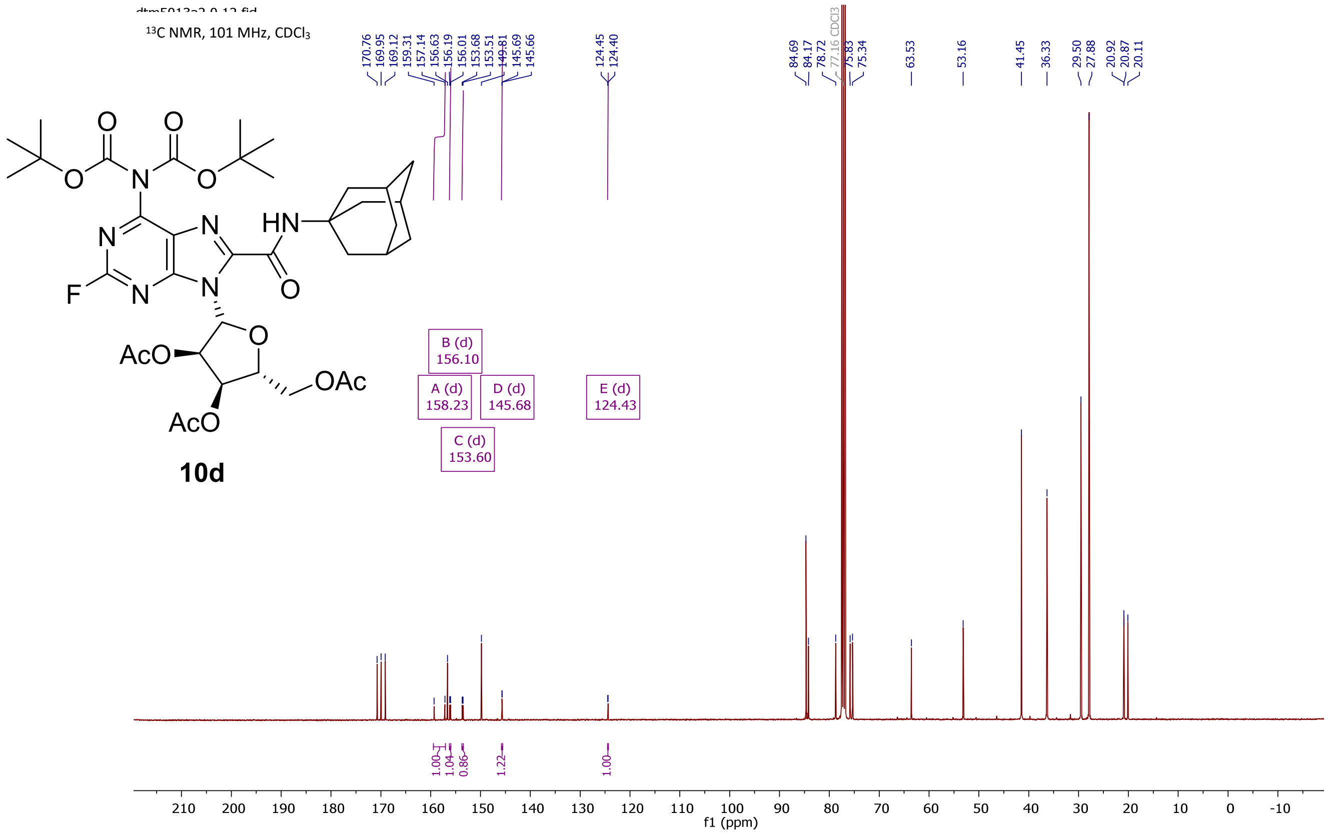

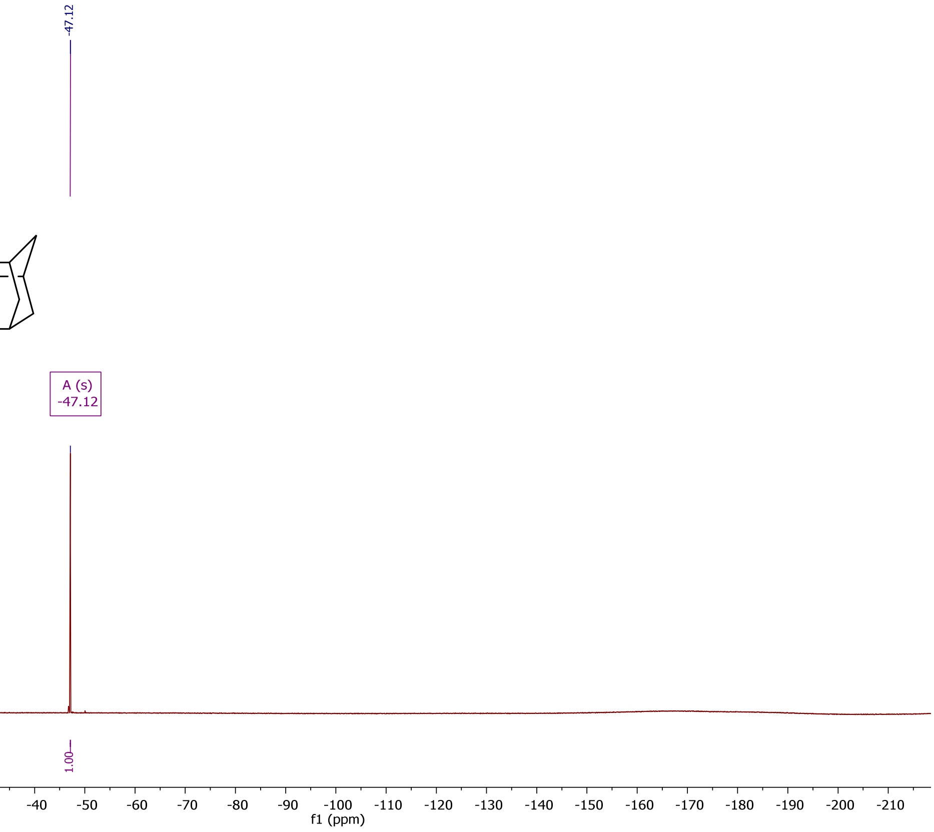
dtm5013a2.0.11.fid
User dtm
^{19}F NMR, 376 MHz, CDCl$_3$
-47.12
A (s)
-47.12
1.00
10 0 -10 -20 -30 -40 -50 -60 -70 -80 -90 -100 -110 -120 -130 -140 -150 -160 -170 -180 -190 -200 -210
f1 (ppm)

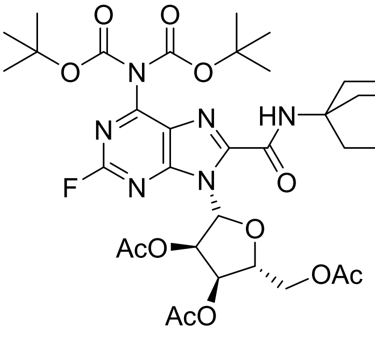

10d

DTM5007A.10.fid
User dtm

^{1}H NMR, 400 MHz, $CDCl_3$

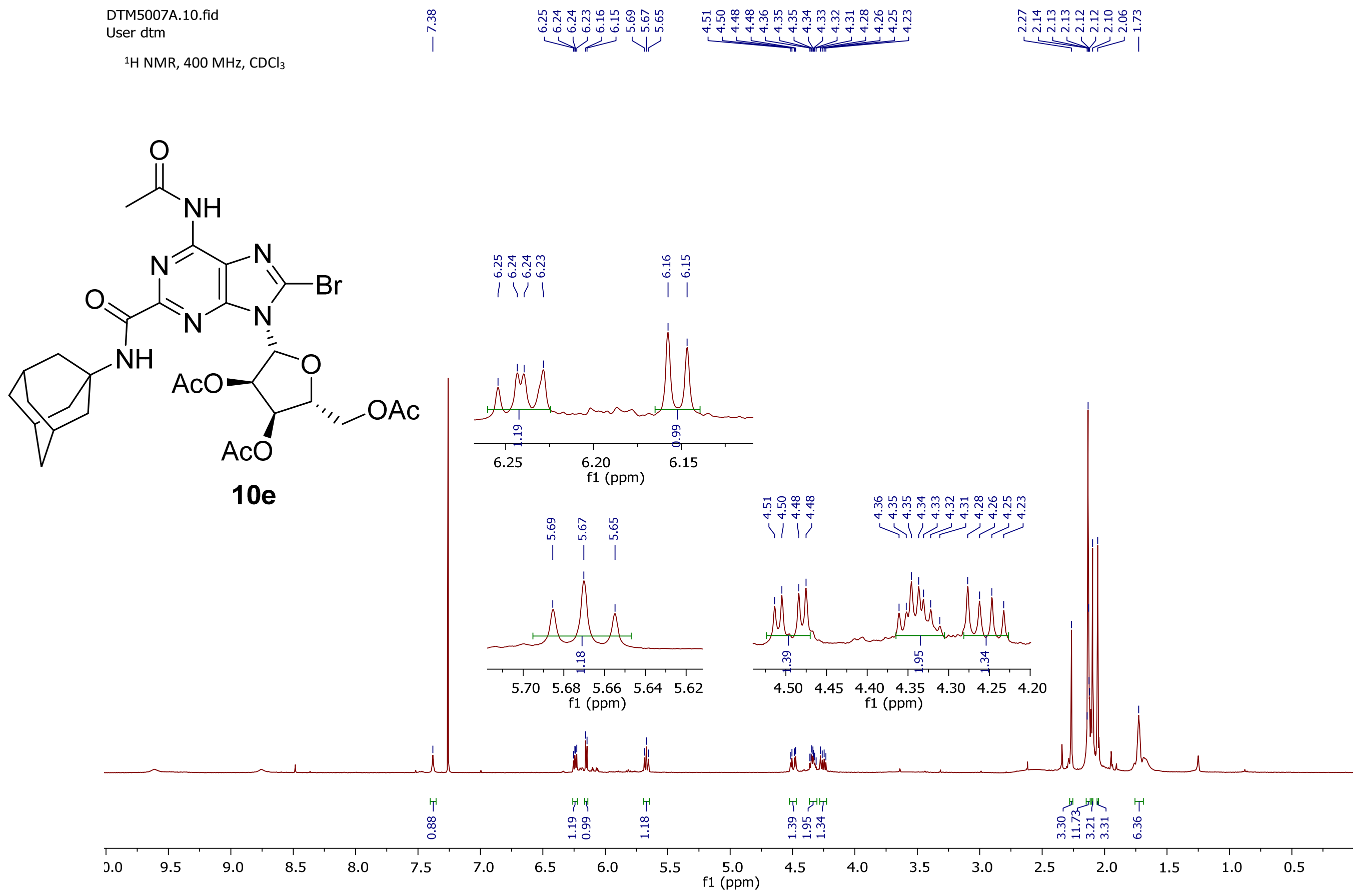

10e

^{13}C NMR, 101 MHz, $CDCl_3$

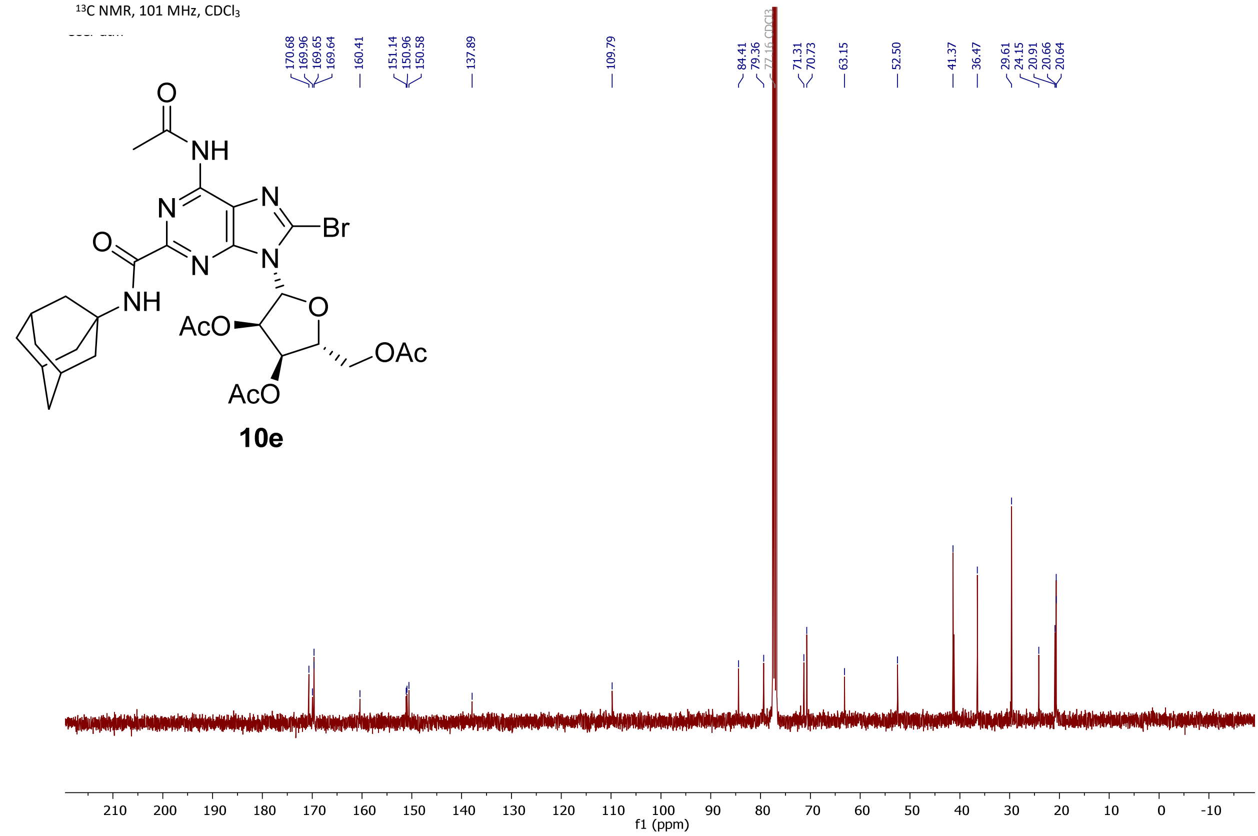

10e

dtmh3096f11-19.1.fid

^{1}H NMR, 400 MHz, CDCl$_3$

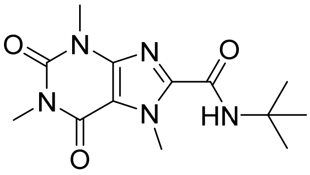

4m

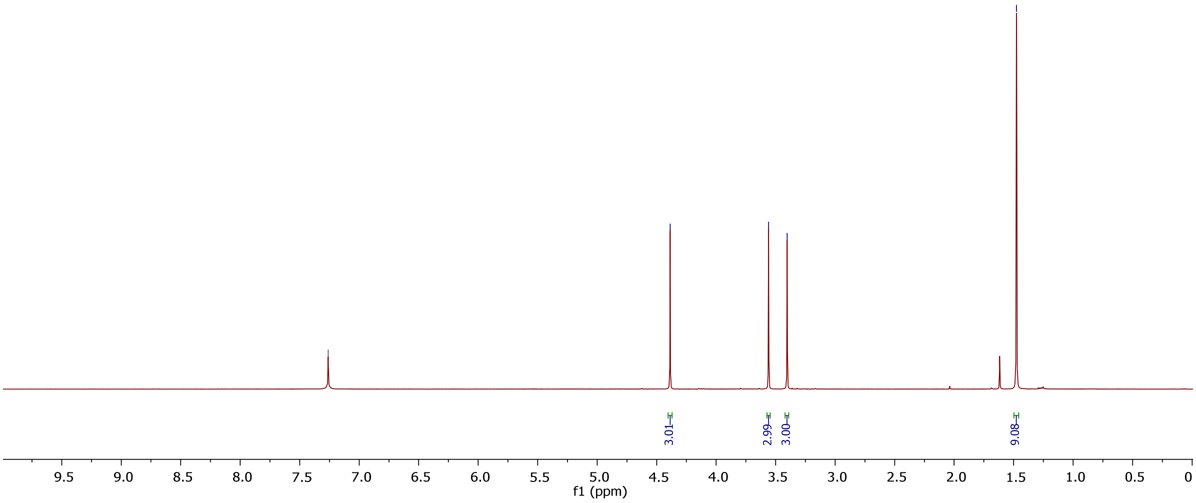

dtmc3096f11-19.5.fid

^{13}C NMR, 101 MHz, CDCl$_3$

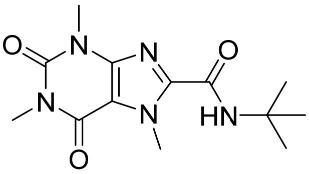

4m

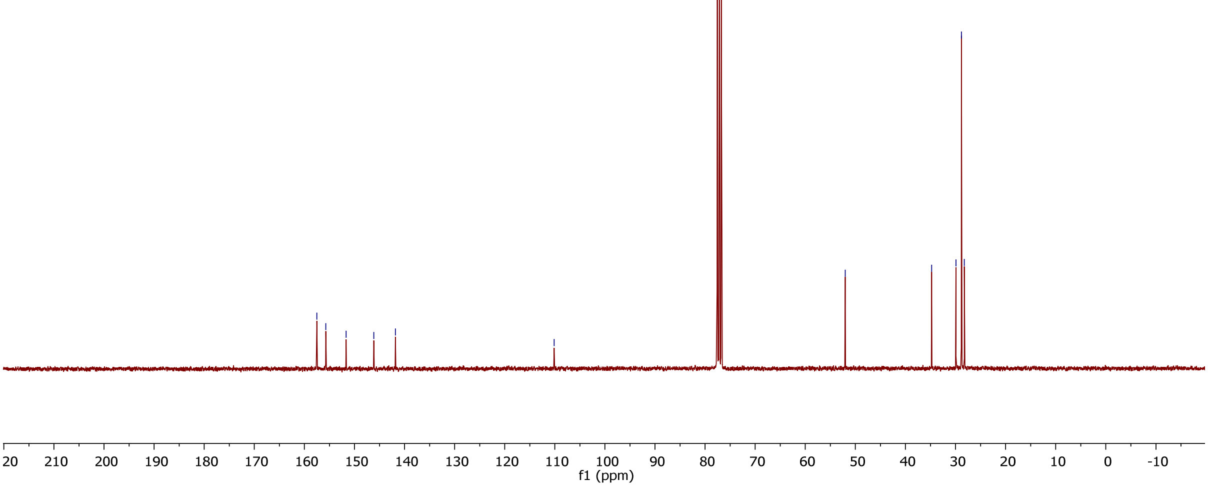

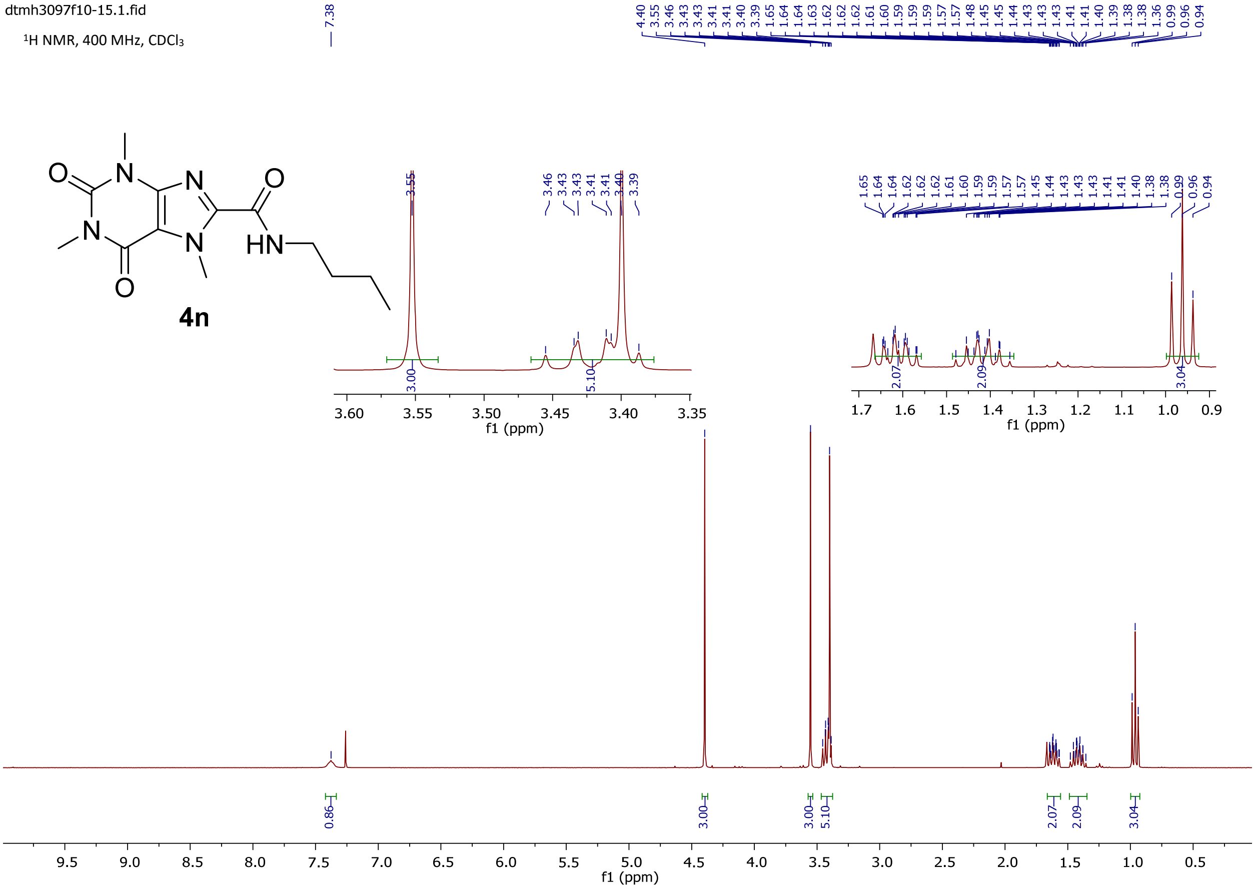
dtmh3097f10-15.1.fid
^{1}H NMR, 400 MHz, $CDCl_3$
7.38
4.40
3.55
3.46
3.43
3.43
3.41
3.41
3.40
3.39
1.65
1.64
1.64
1.63
1.62
1.62
1.62
1.61
1.60
1.59
1.59
1.59
1.57
1.57
1.48
1.45
1.45
1.44
1.43
1.43
1.43
1.41
1.41
1.40
1.39
1.38
1.38
1.36
0.99
0.96
0.94
4n
3.00
5.10
2.07
2.09
3.04
0.86
f1 (ppm)

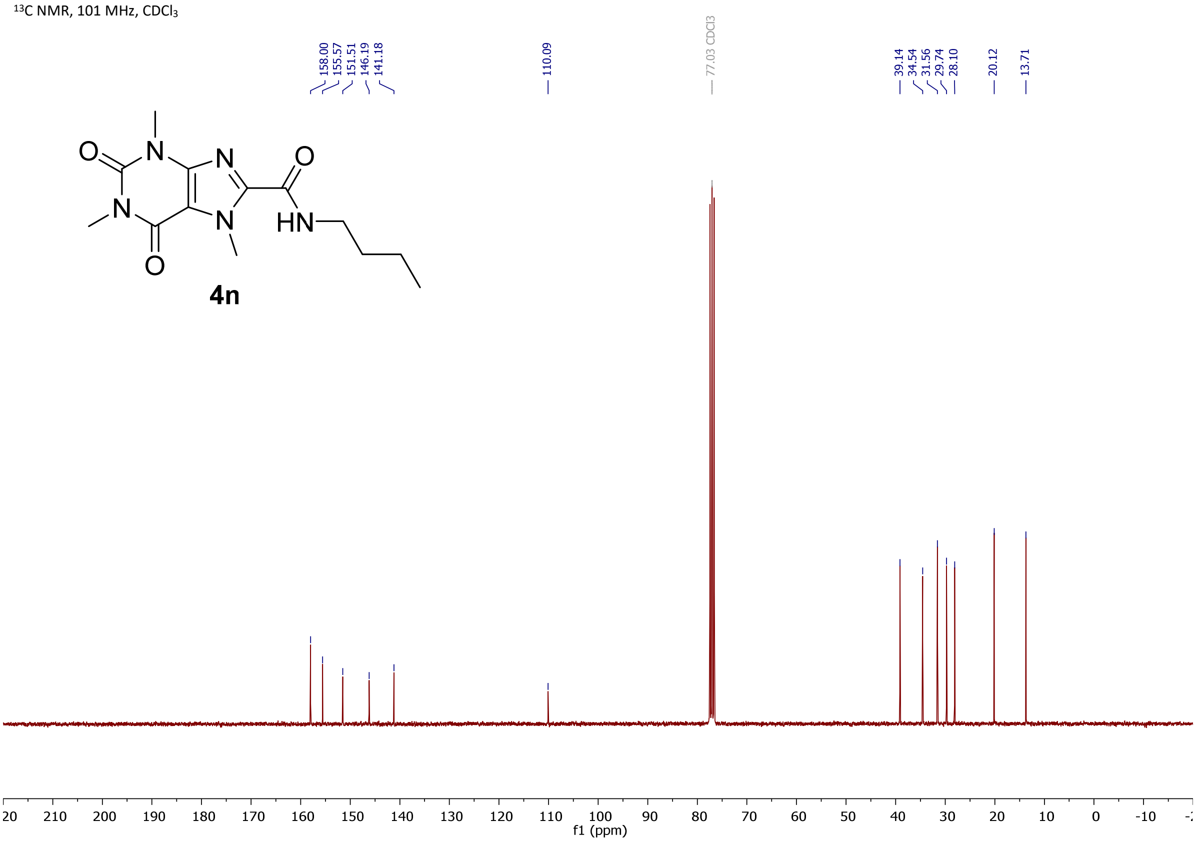
^{13}C NMR, 101 MHz, $CDCl_3$
158.00
155.57
151.51
146.19
141.18
110.09
77.03 CDCl3
39.14
34.54
31.56
29.74
28.10
20.12
13.71
4n
f1 (ppm)

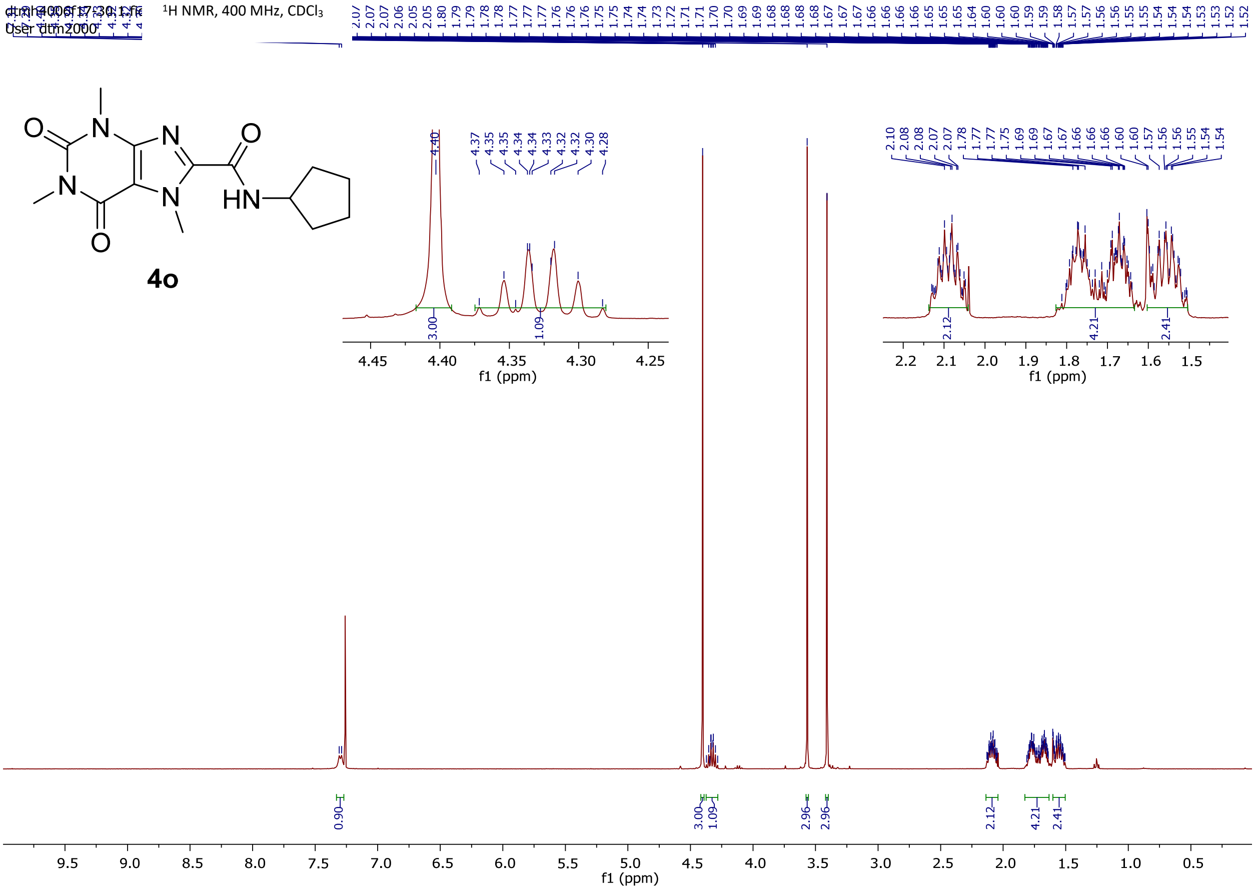

1H NMR, 400 MHz, CDCl3
4o
f1 (ppm)

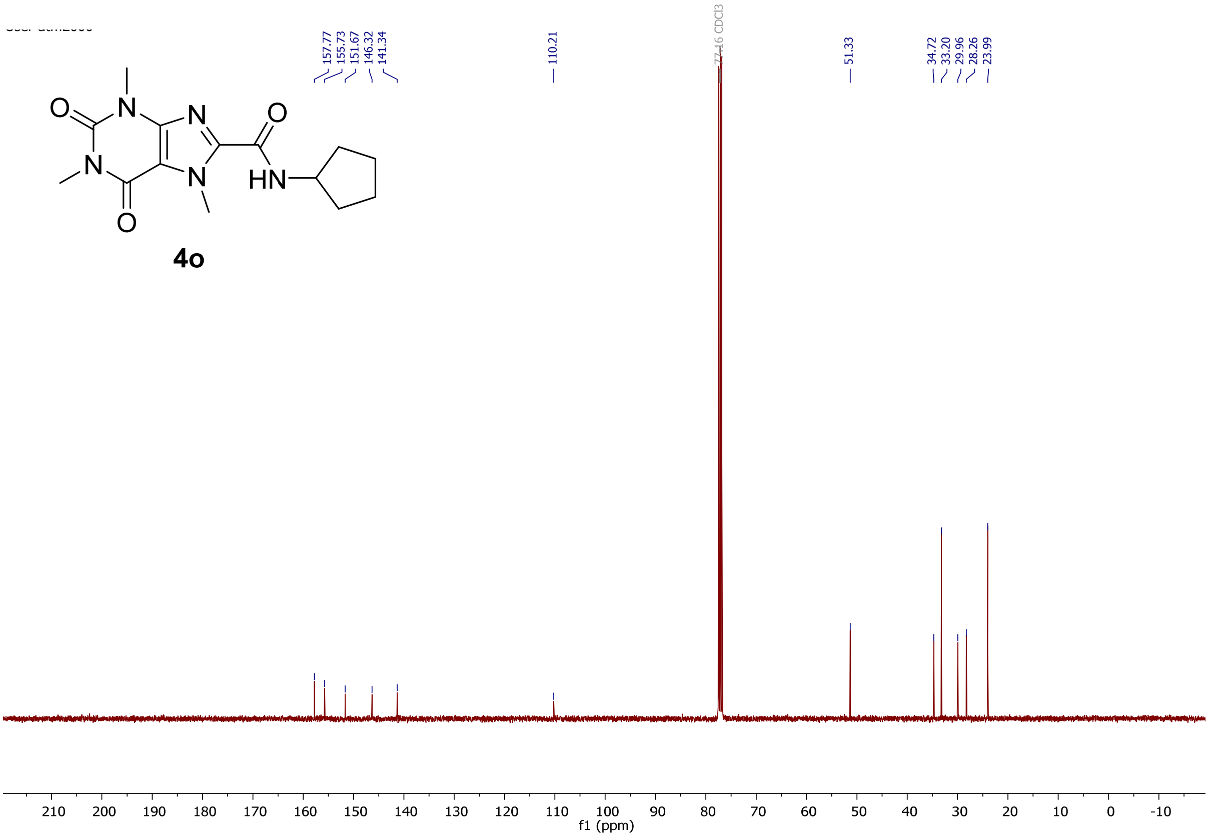

157.77
155.73
151.67
146.32
141.34
110.21
77.16 CDCl3
51.33
34.72
33.20
29.96
28.26
23.99
4o
f1 (ppm)

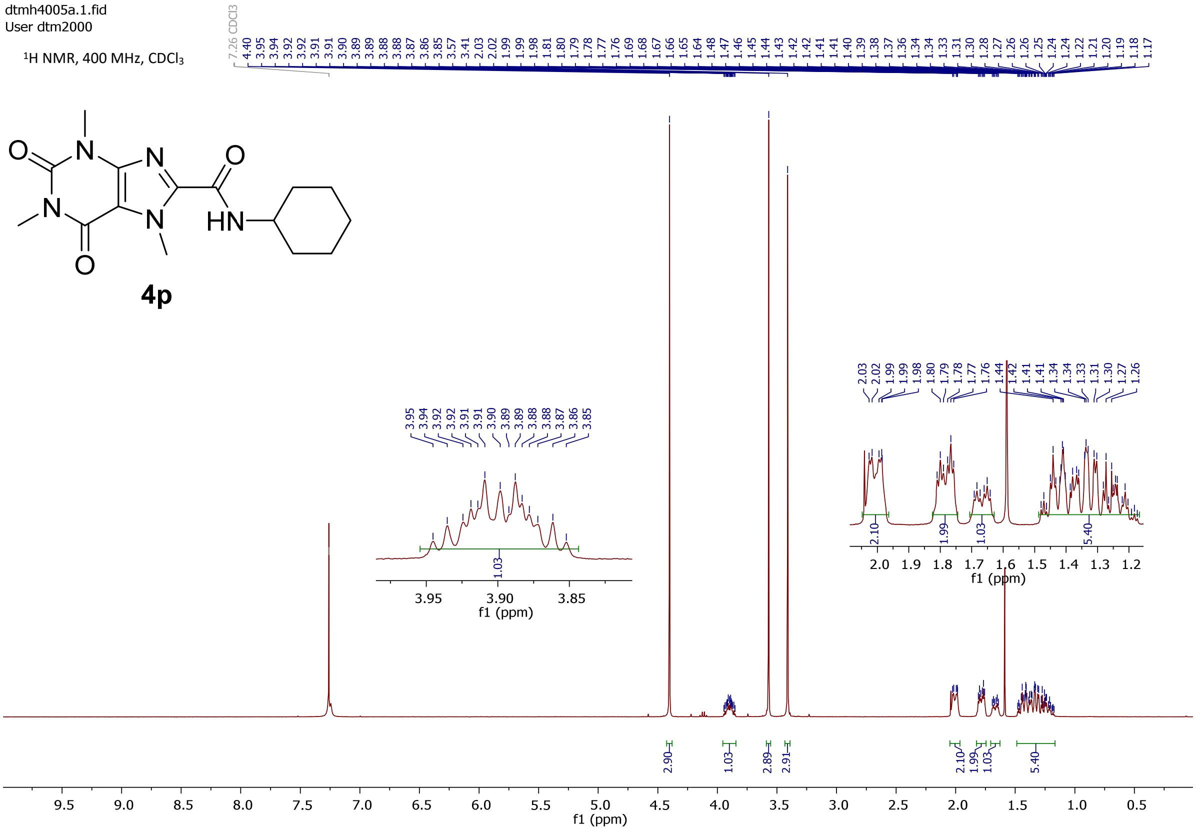
dtmh4005a.1.fid
User dtm2000
¹H NMR, 400 MHz, CDCl₃
7.26 CDCl3
4.40 3.95 3.94 3.92 3.92 3.91 3.91 3.90 3.89 3.89 3.88 3.88 3.87 3.86 3.85 3.57 3.41 2.03 2.02 1.99 1.99 1.99 1.98 1.81 1.80 1.79 1.79 1.78 1.77 1.76 1.69 1.68 1.67 1.66 1.65 1.64 1.48 1.47 1.46 1.45 1.44 1.43 1.42 1.42 1.41 1.41 1.40 1.39 1.38 1.37 1.36 1.34 1.34 1.33 1.31 1.31 1.30 1.28 1.27 1.26 1.26 1.25 1.24 1.24 1.22 1.21 1.20 1.19 1.18 1.17
O N N O N N HN O O
4p
2.90 1.03 2.89 2.91 2.10 1.99 1.03 5.40
f1 (ppm)

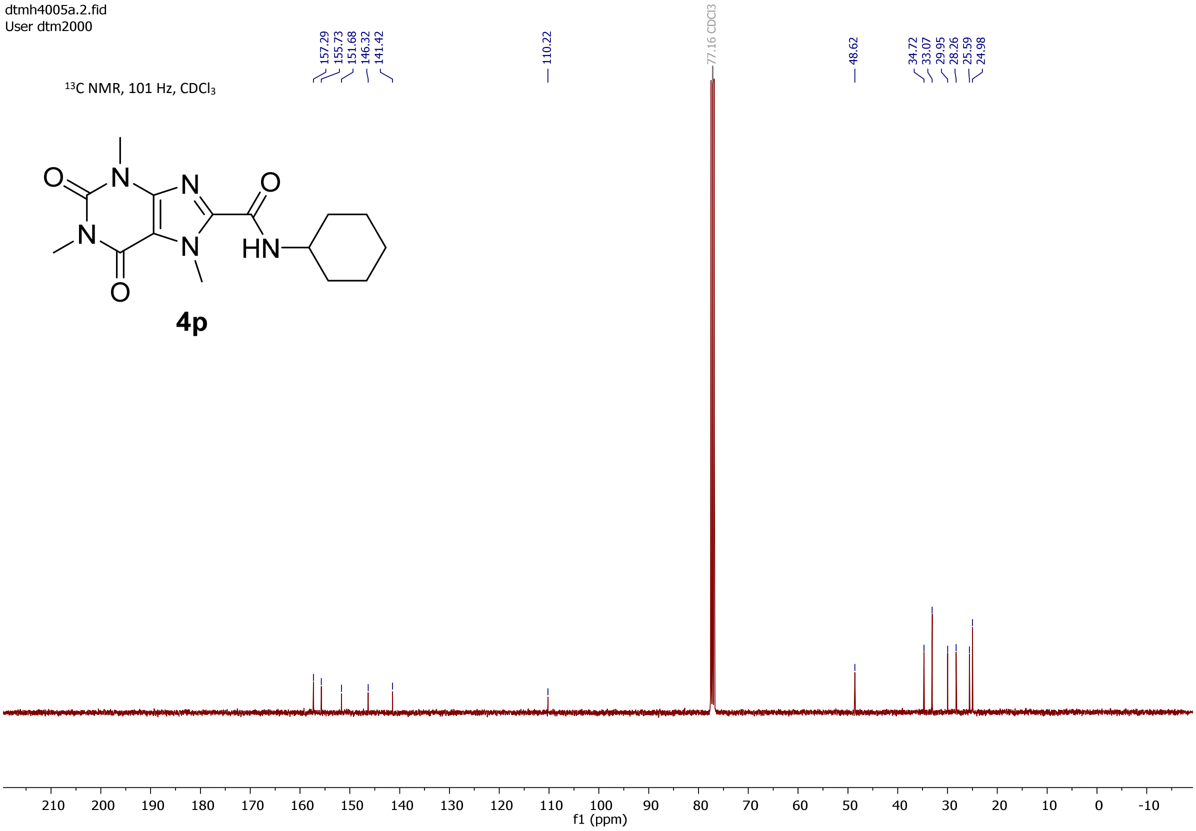
dtmh4005a.2.fid
User dtm2000
¹³C NMR, 101 Hz, CDCl₃
157.29 155.73 151.68 146.32 141.42
110.22
77.16 CDCl3
48.62
34.72 33.07 29.95 28.26 25.59 24.98
O N N O N N HN O O
4p
f1 (ppm)

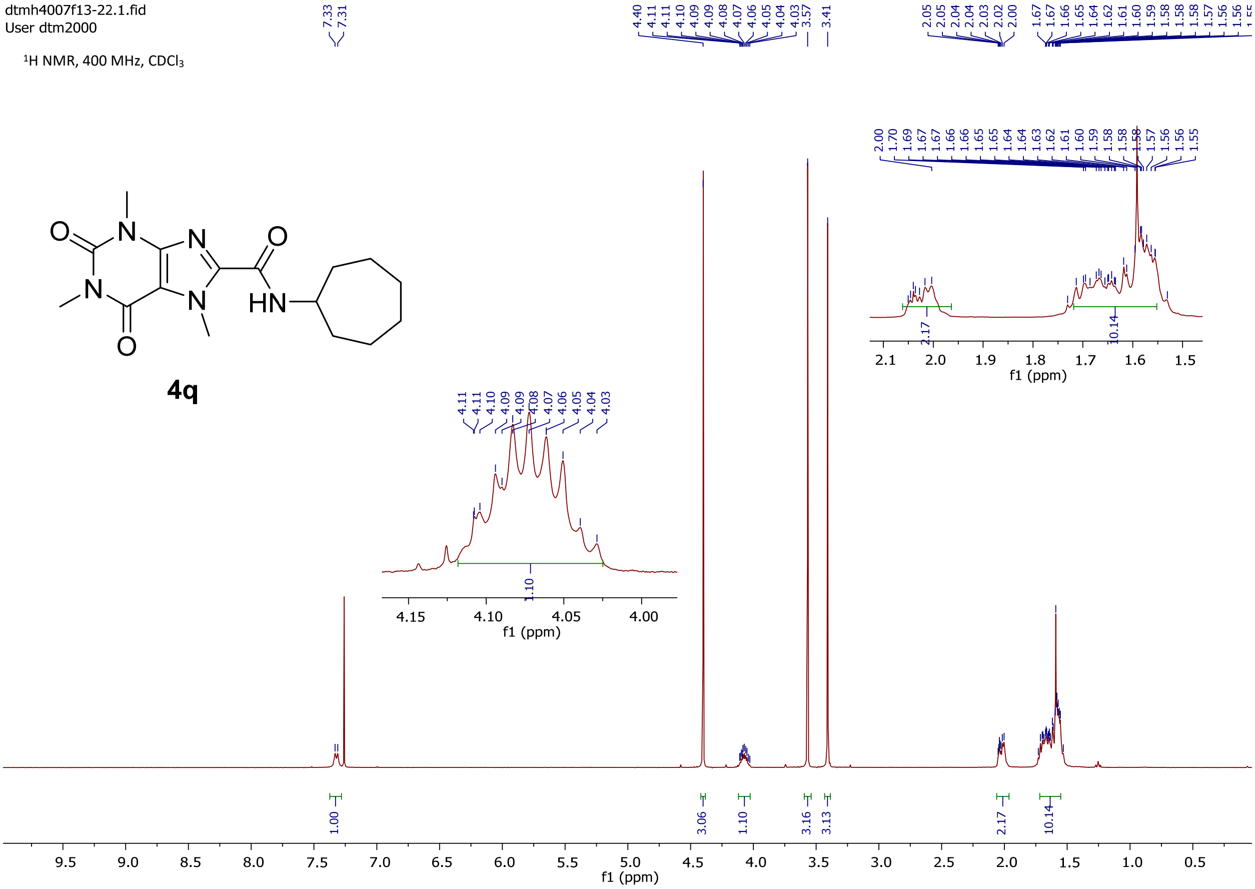
dtmh4007f13-22.1.fid
User dtm2000
^{1}H NMR, 400 MHz, CDCl$_3$
4q
f1 (ppm)

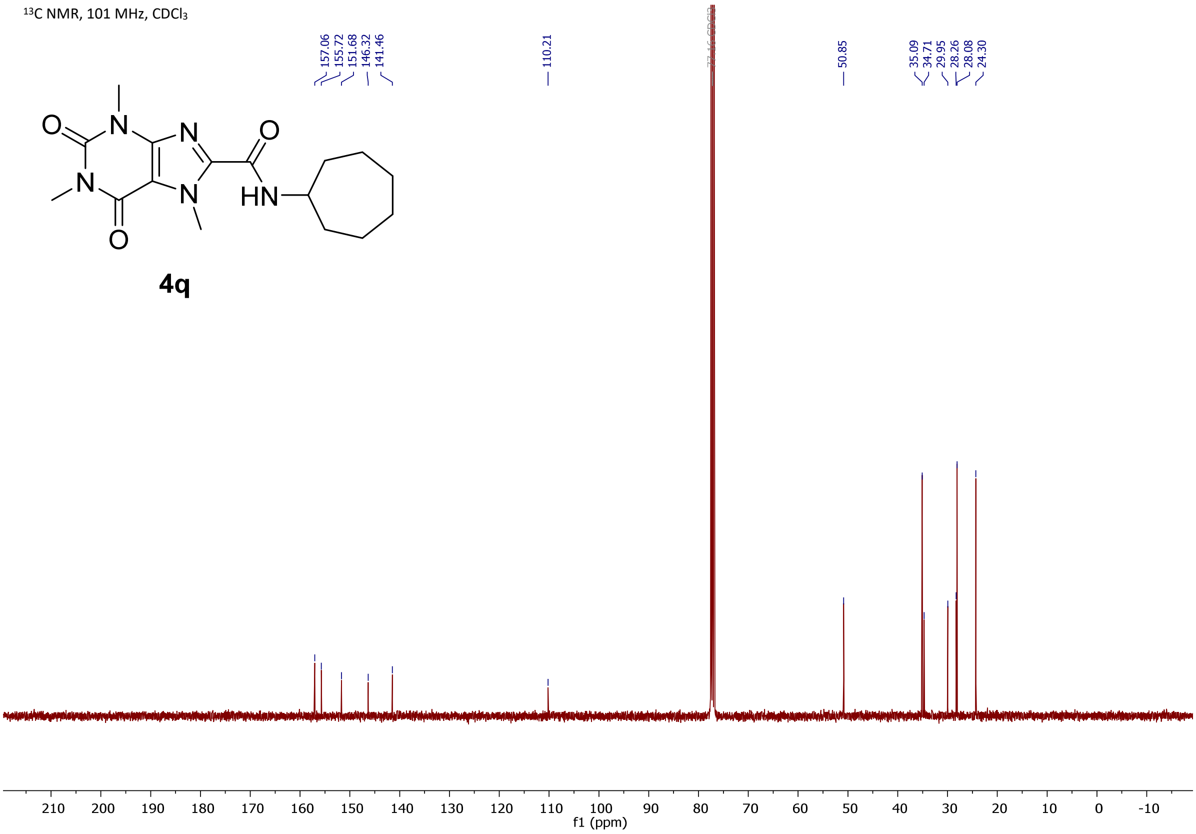
^{13}C NMR, 101 MHz, CDCl$_3$
157.06
155.72
151.68
146.32
141.46
110.21
50.85
35.09
34.71
29.95
28.26
28.08
24.30
4q
f1 (ppm)

dtmh3093a.1.fid ^{1}H NMR, 400 MHz, $CDCl_3$

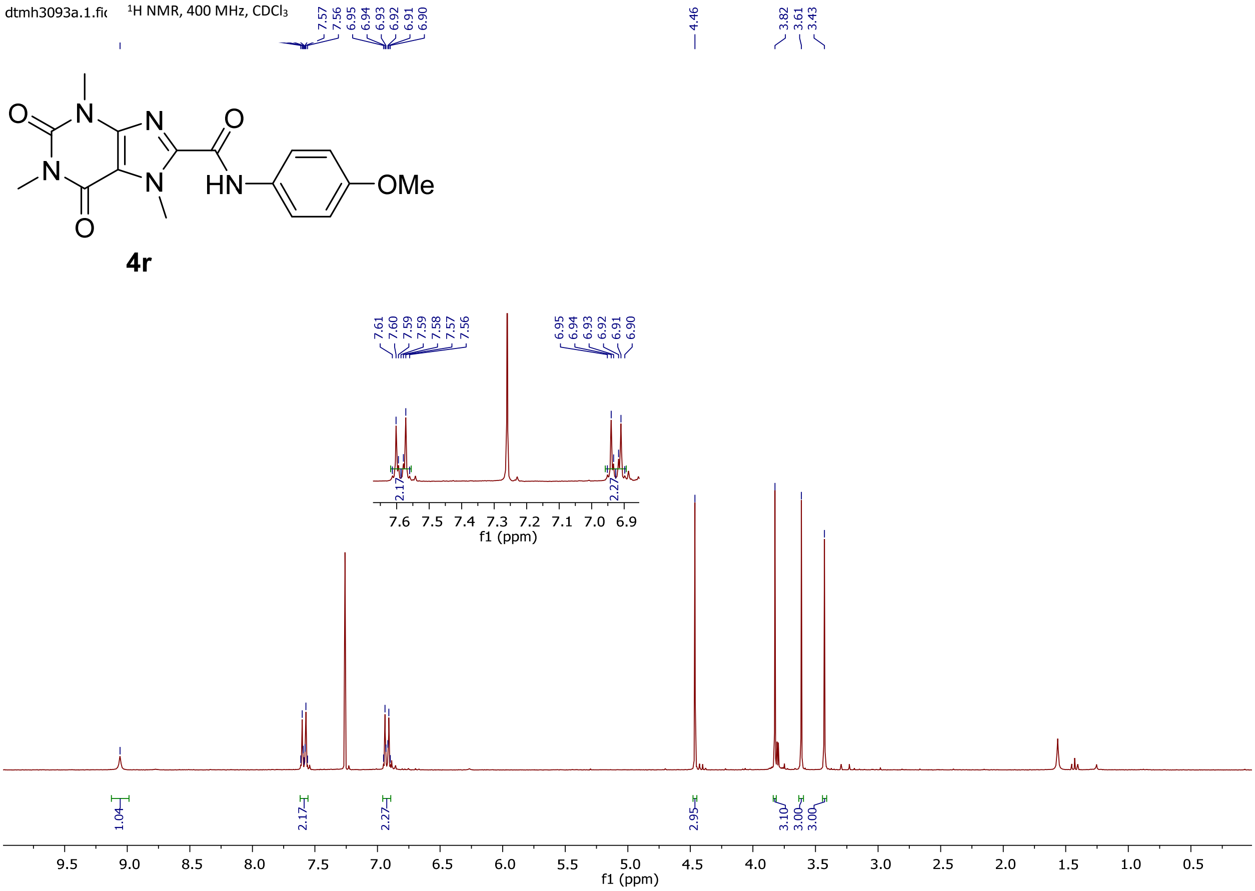

^{13}C NMR, 101 MHz, $CDCl_3$

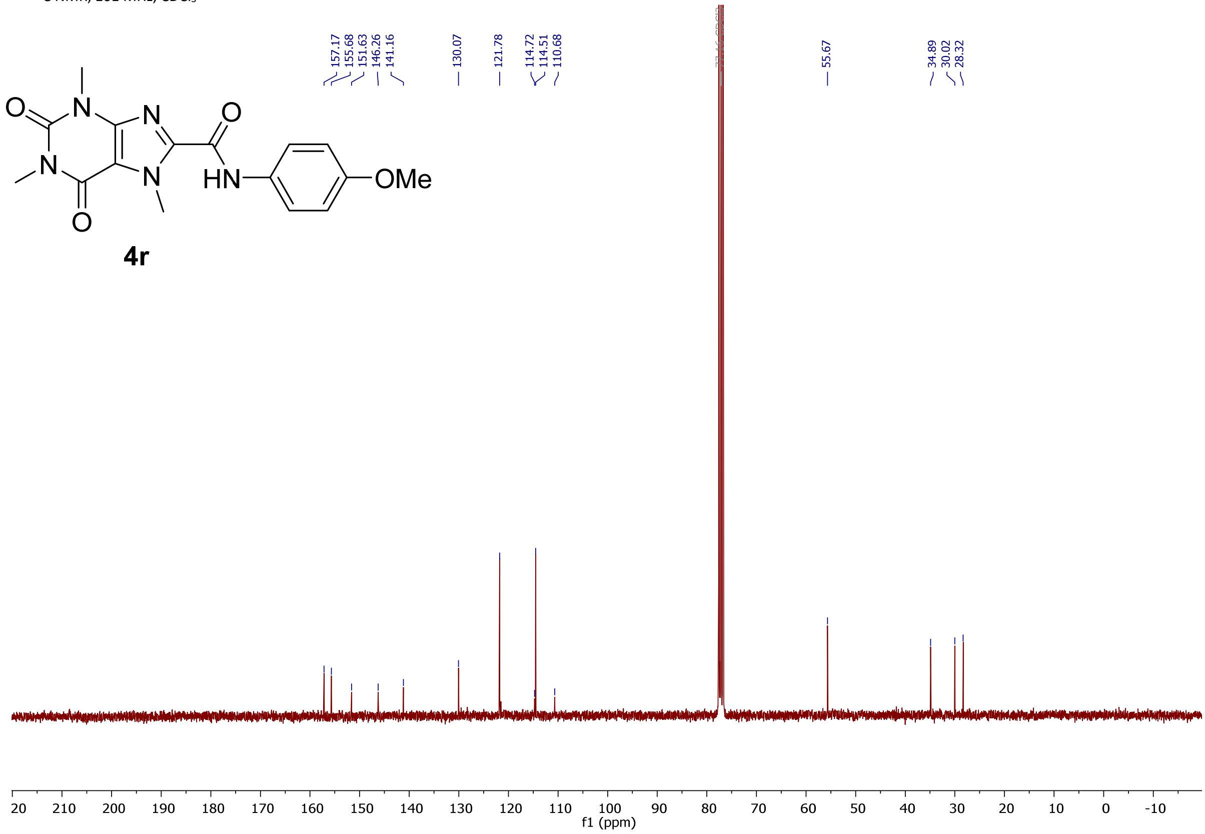

dtmh4016f12-18[illegible]1.fid

^{1}H NMR, 400 MHz, $CDCl_3$

4s

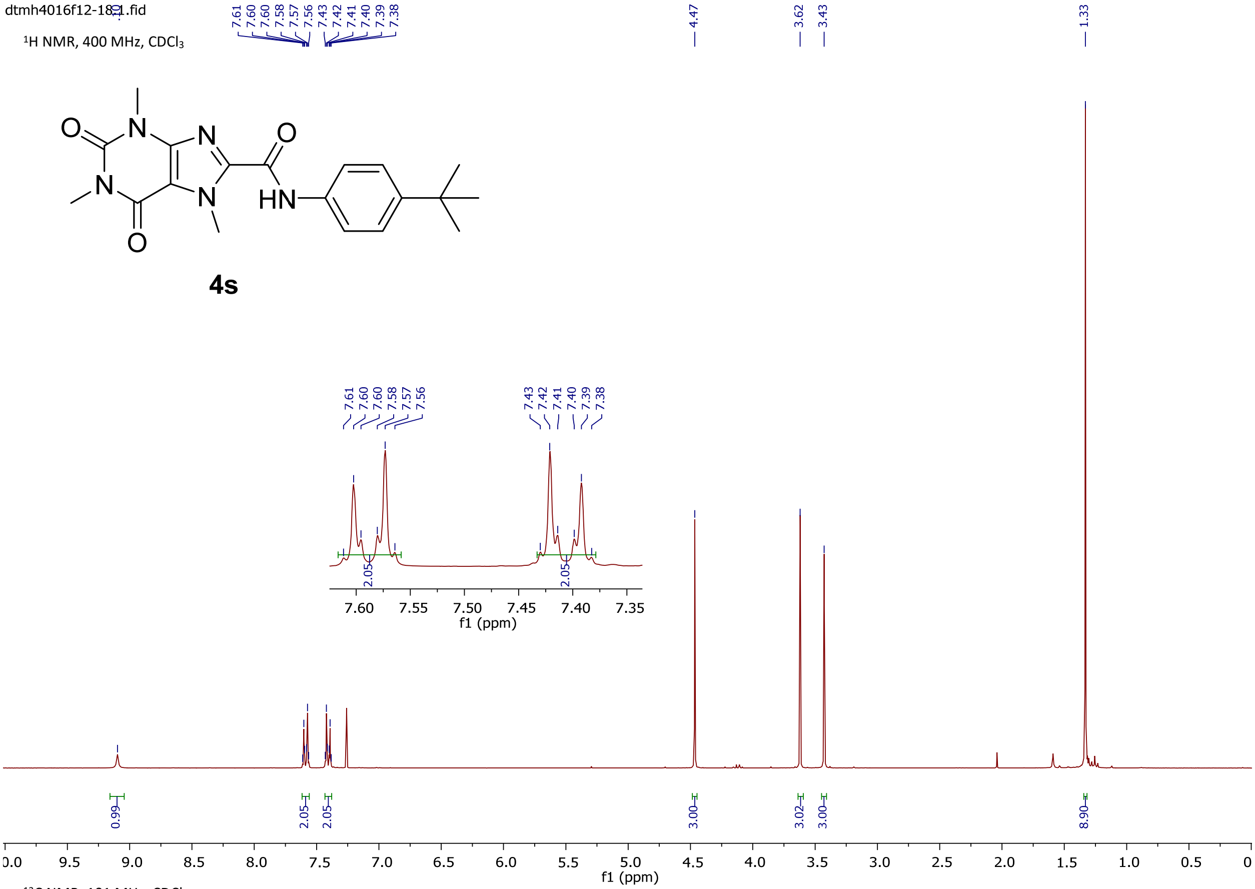

^{13}C NMR, 101 MHz, $CDCl_3$

4s

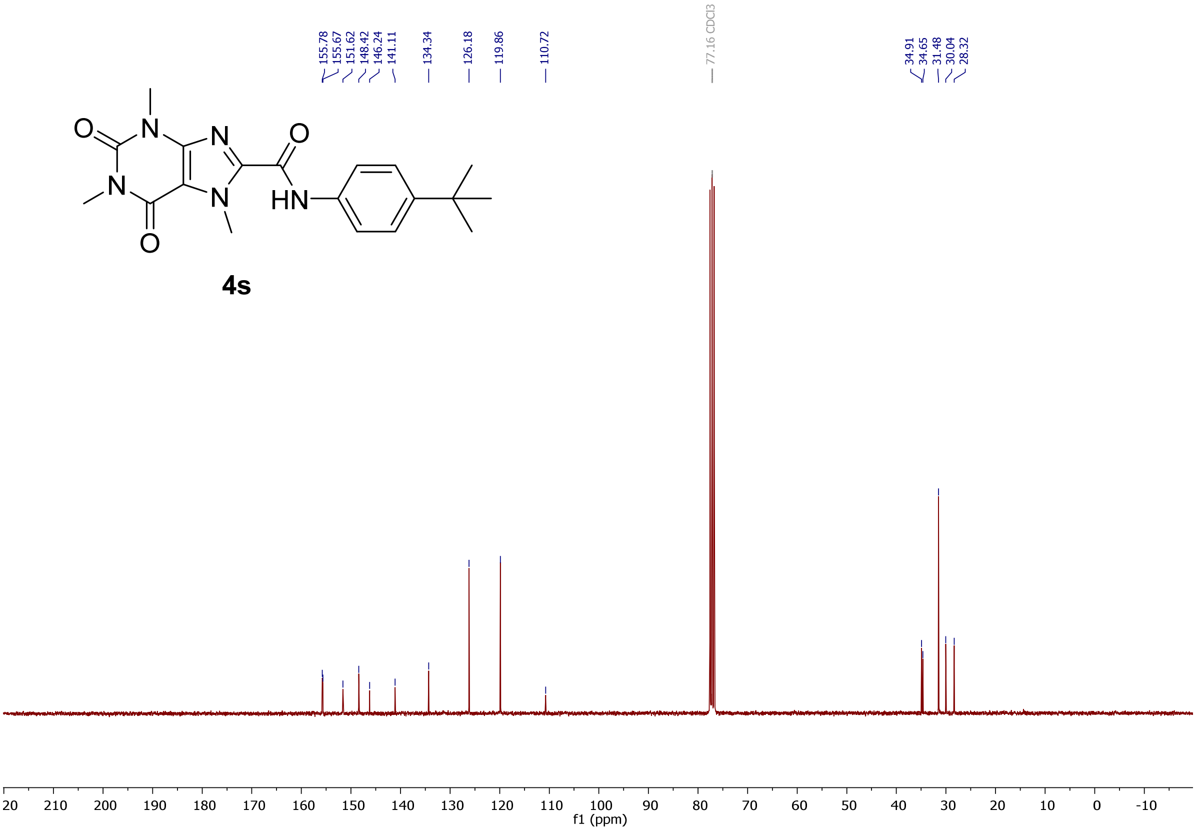

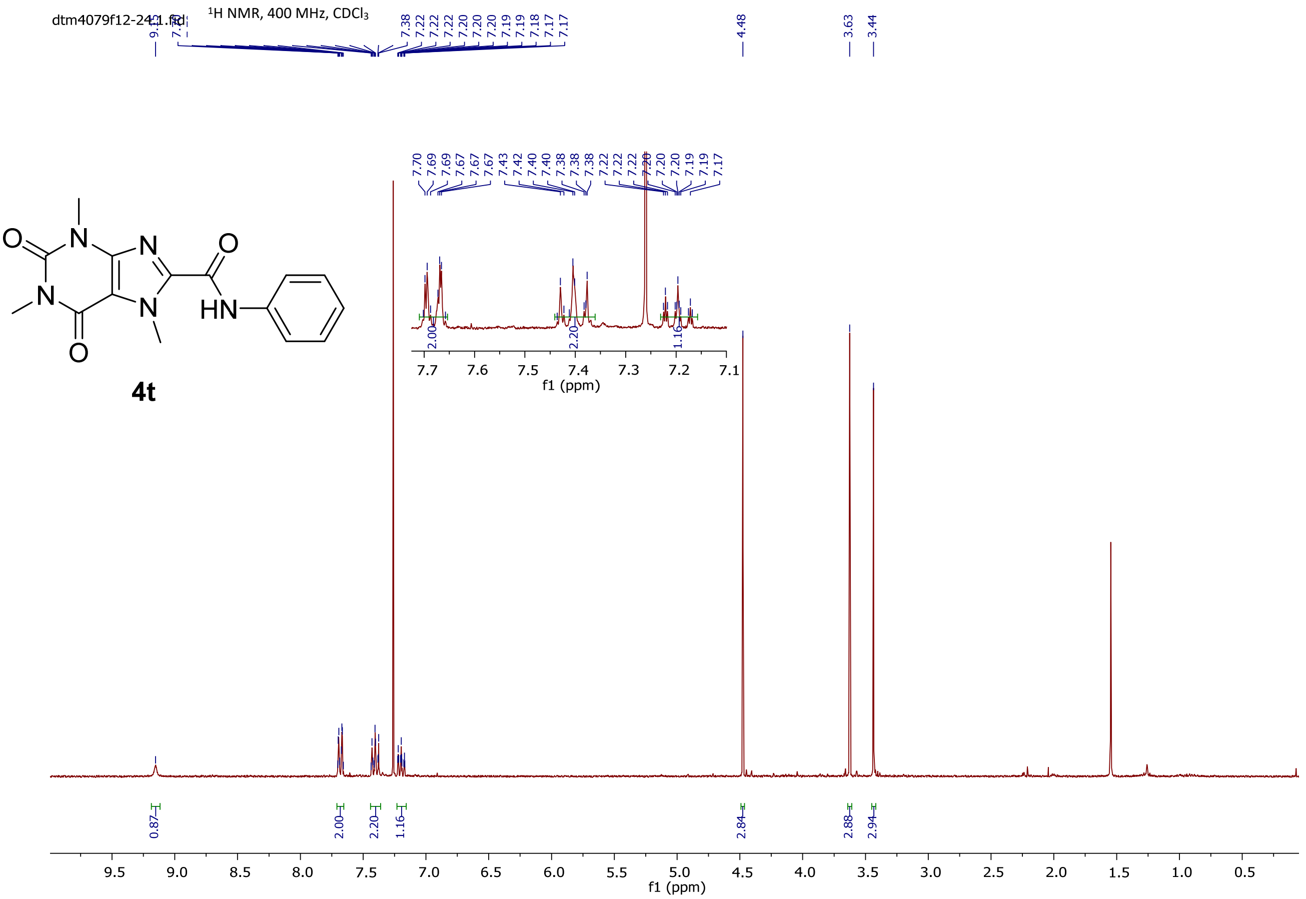
dtm4079f12-241.fid
1H NMR, 400 MHz, CDCl3
9.14
7.38
7.22
7.22
7.22
7.20
7.20
7.20
7.19
7.19
7.18
7.17
7.17
4.48
3.63
3.44
7.70
7.69
7.69
7.67
7.67
7.67
7.43
7.42
7.40
7.38
7.38
7.38
7.22
7.22
7.22
7.20
7.20
7.20
7.19
7.19
7.17
2.00
2.20
1.16
7.7
7.6
7.5
7.4
7.3
7.2
7.1
f1 (ppm)
O
N
N
O
N
HN
N
O
4t
0.87
2.00
2.20
1.16
2.84
2.88
2.94
9.5
9.0
8.5
8.0
7.5
7.0
6.5
6.0
5.5
5.0
4.5
4.0
3.5
3.0
2.5
2.0
1.5
1.0
0.5
f1 (ppm)

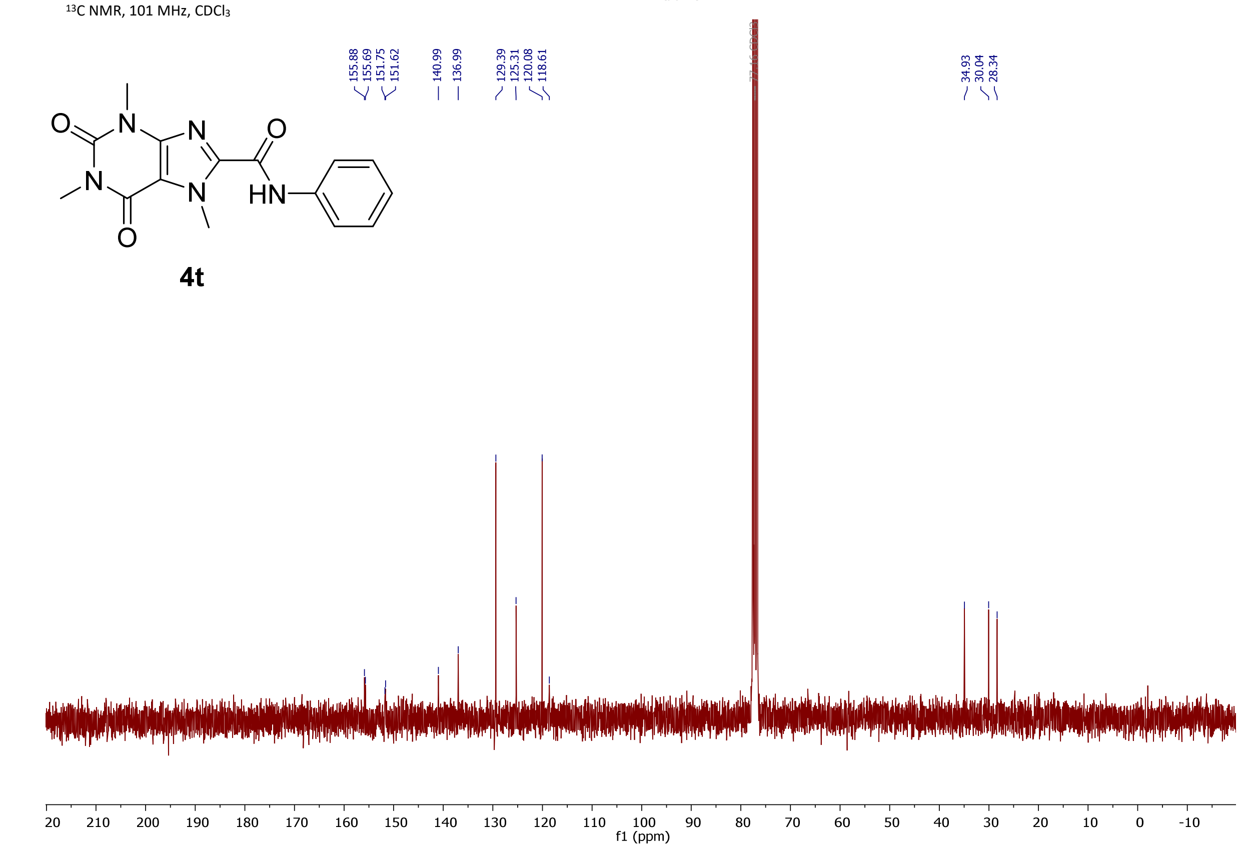
13C NMR, 101 MHz, CDCl3
155.88
155.69
151.75
151.62
140.99
136.99
129.39
125.31
120.08
118.61
34.93
30.04
28.34
O
N
N
O
N
HN
N
O
4t
20
210
200
190
180
170
160
150
140
130
120
110
100
90
80
70
60
50
40
30
20
10
0
-10
f1 (ppm)

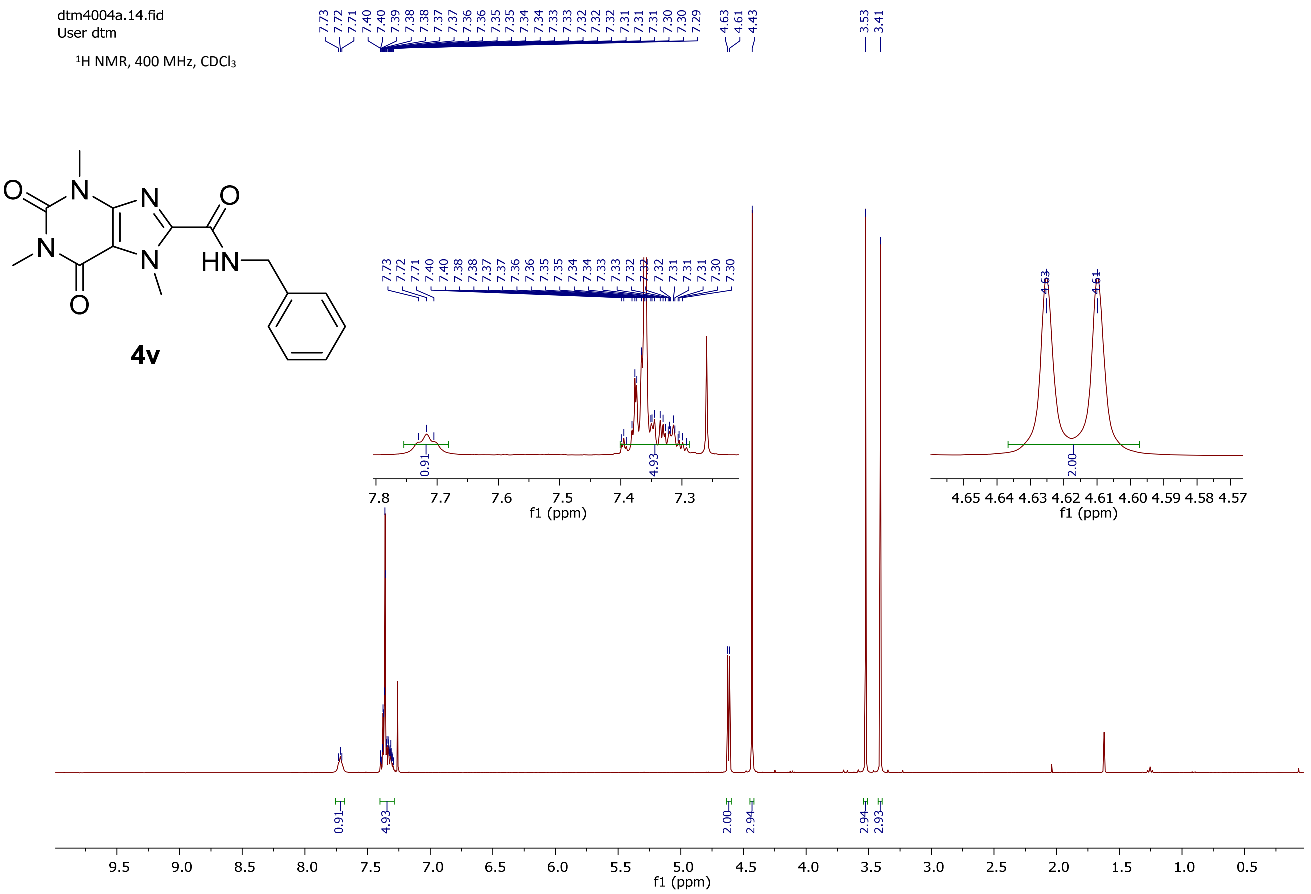
dtm4004a.14.fid
User dtm
1H NMR, 400 MHz, CDCl3
7.73 7.72 7.71 7.40 7.39 7.38 7.38 7.37 7.37 7.36 7.36 7.35 7.35 7.34 7.34 7.33 7.33 7.32 7.32 7.32 7.31 7.31 7.31 7.30 7.30 7.29
4.63 4.61 4.43
3.53 3.41
4v
0.91 4.93 2.00 2.94 2.94 2.93
f1 (ppm)

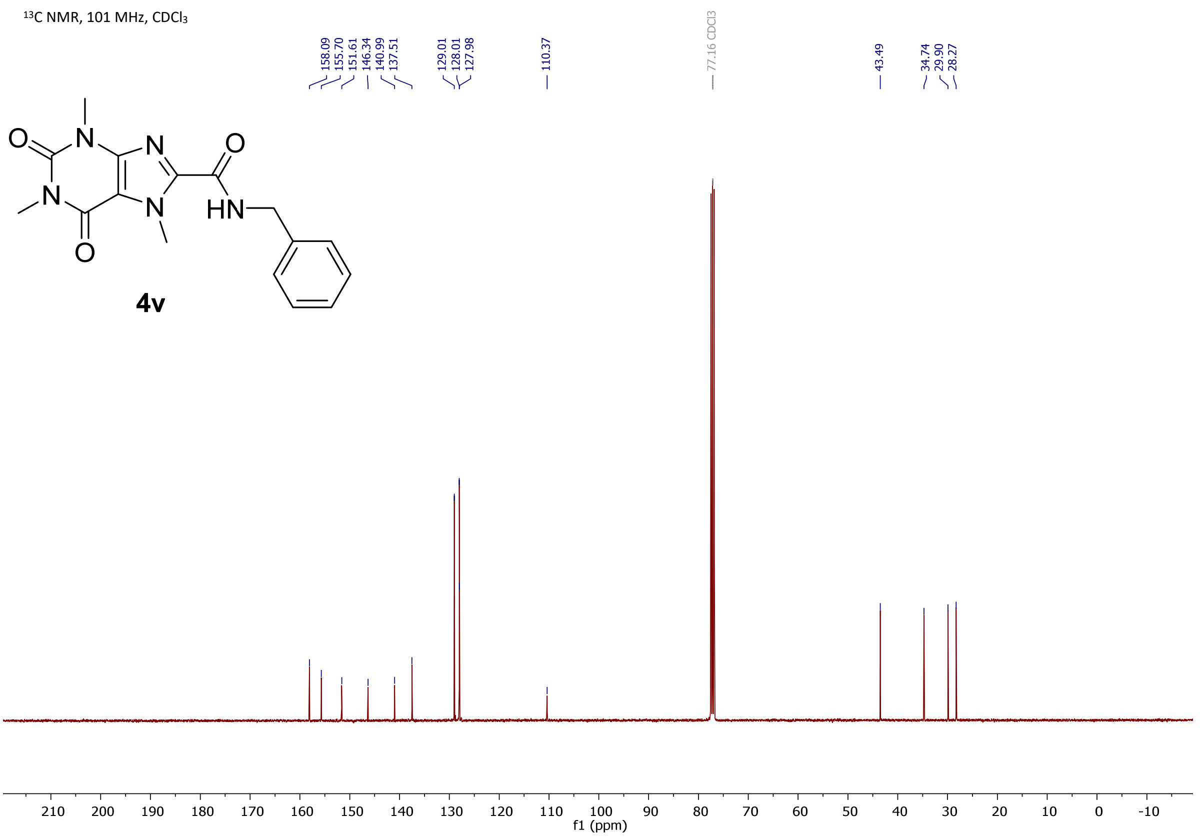
13C NMR, 101 MHz, CDCl3
158.09 155.70 151.61 146.34 140.99 137.51
129.01 128.01 127.98
110.37
77.16 CDCl3
43.49
34.74 29.90 28.27
4v
f1 (ppm)

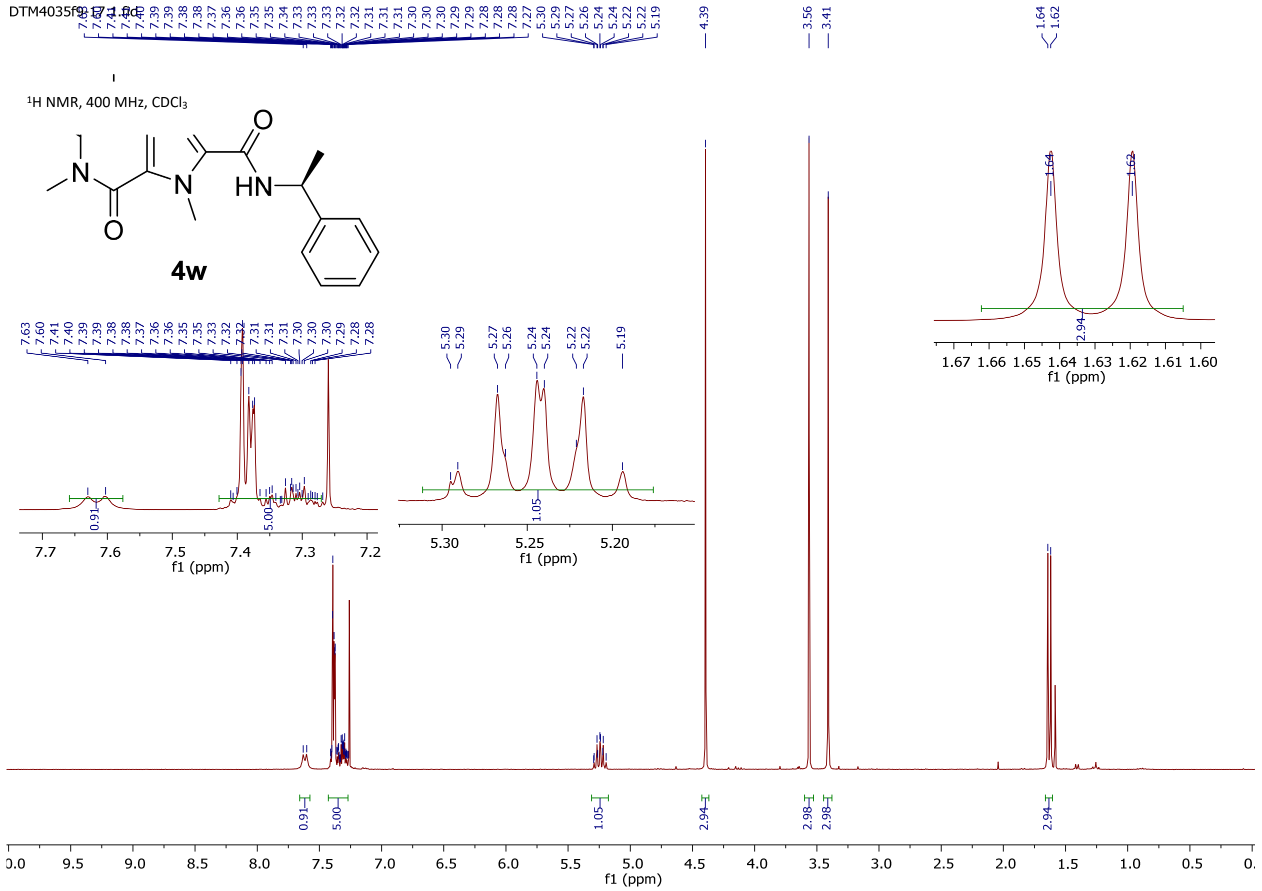

^{1}H NMR, 400 MHz, $CDCl_3$
4w
HN
O
N
f1 (ppm)
4.39
3.56
3.41
1.64
1.62
0.91
5.00
1.05
2.94
2.98
2.98
2.94

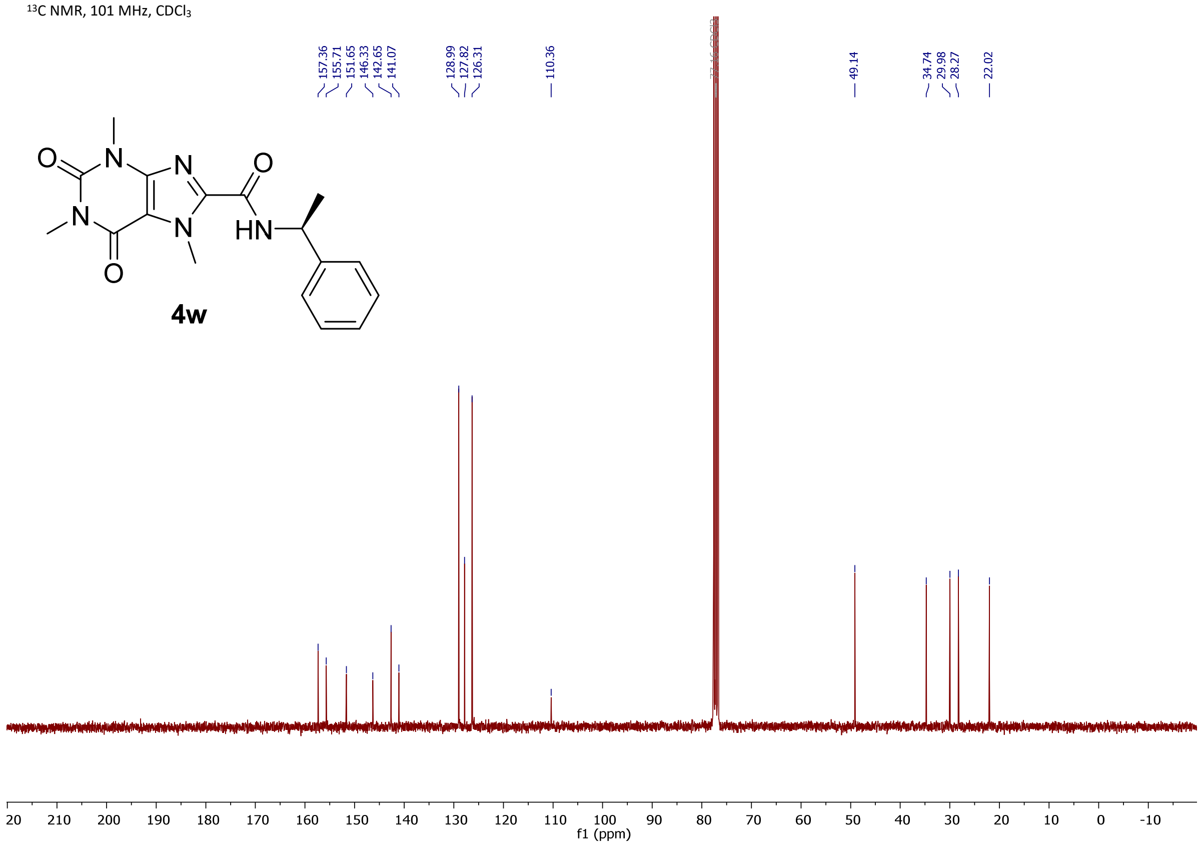

^{13}C NMR, 101 MHz, $CDCl_3$
4w
HN
O
N
157.36
155.71
151.65
146.33
142.65
141.07
128.99
127.82
126.31
110.36
49.14
34.74
29.98
28.27
22.02
f1 (ppm)

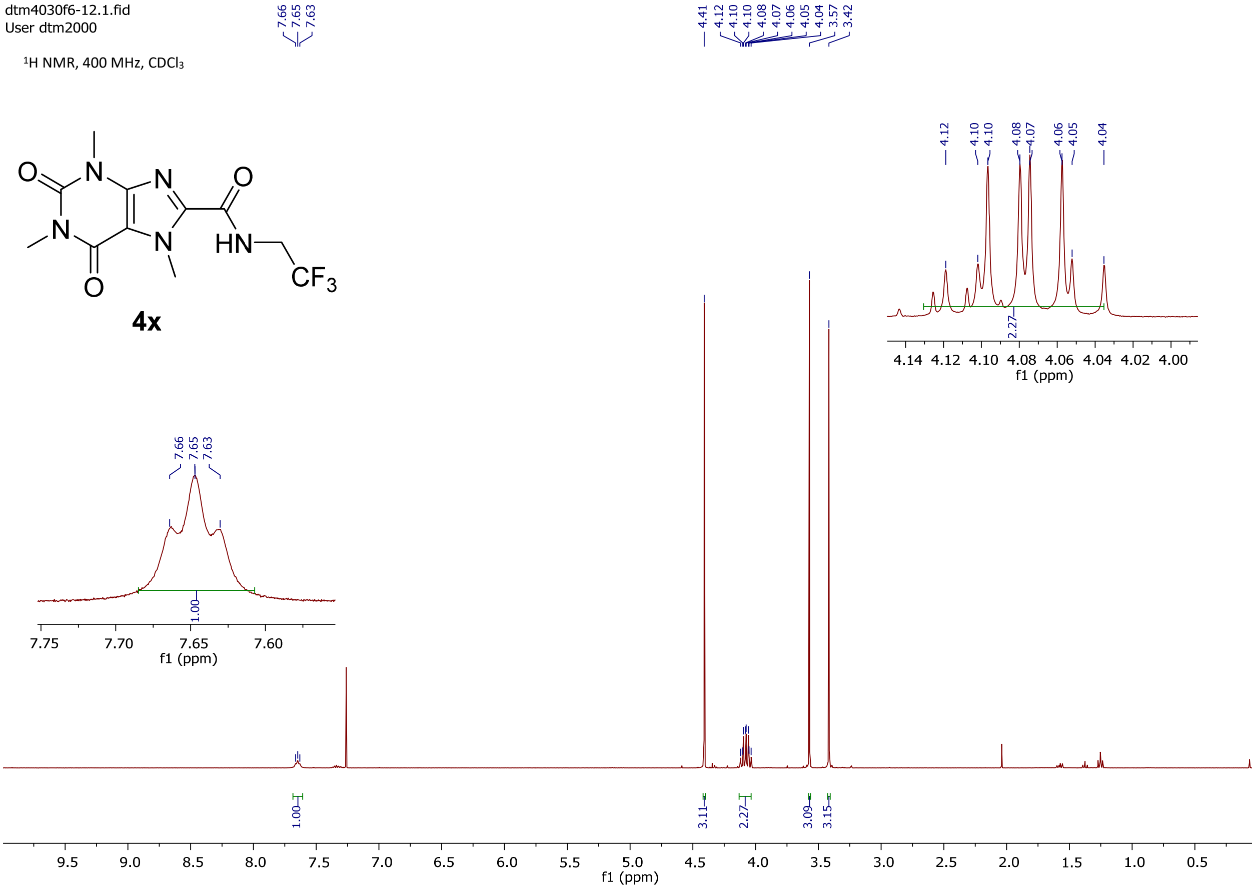
dtm4030f6-12.1.fid
User dtm2000
^{1}H NMR, 400 MHz, $CDCl_3$
7.66
7.65
7.63
4.41
4.12
4.10
4.10
4.08
4.07
4.06
4.05
4.04
3.57
3.42
4x
1.00
7.75 7.70 7.65 7.60
f1 (ppm)
2.27
4.14 4.12 4.10 4.08 4.06 4.04 4.02 4.00
f1 (ppm)
1.00
3.11
2.27
3.09
3.15
9.5 9.0 8.5 8.0 7.5 7.0 6.5 6.0 5.5 5.0 4.5 4.0 3.5 3.0 2.5 2.0 1.5 1.0 0.5
f1 (ppm)

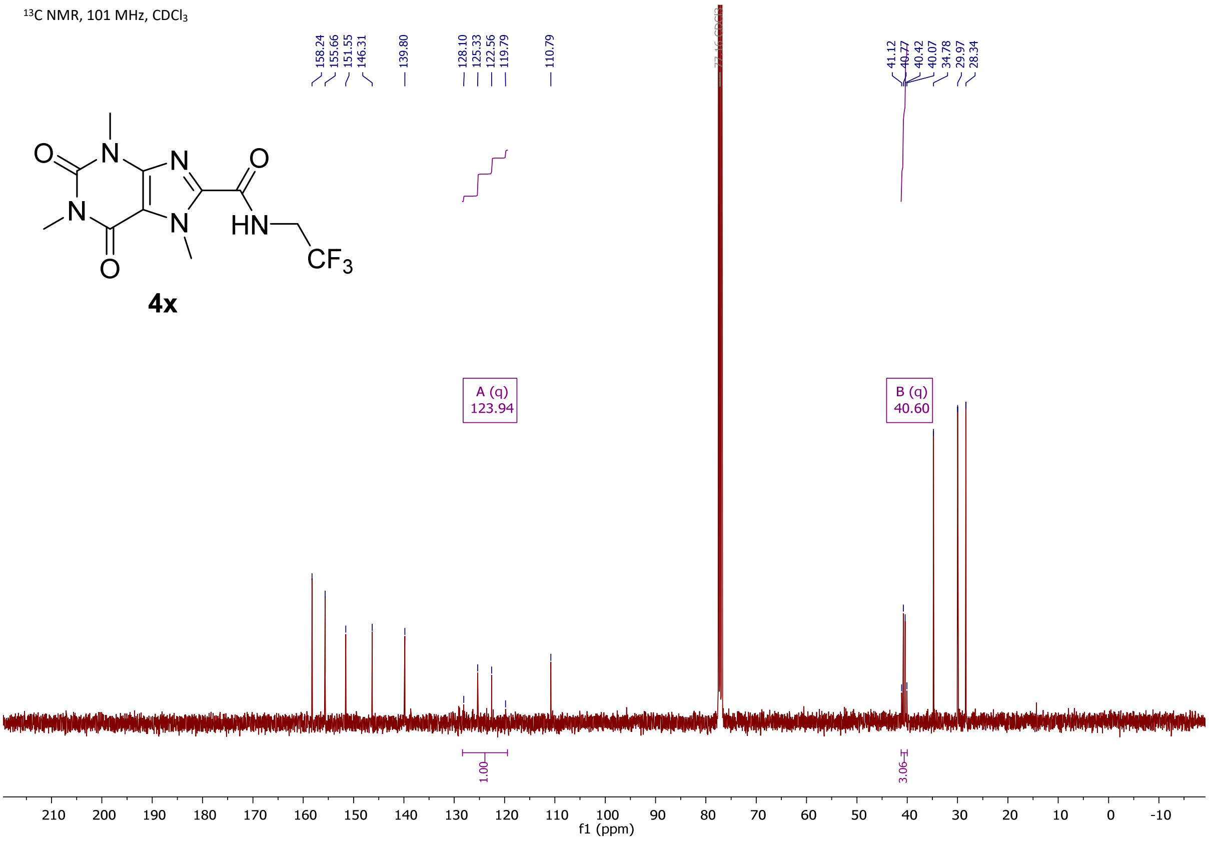
^{13}C NMR, 101 MHz, $CDCl_3$
158.24
155.66
151.55
146.31
139.80
128.10
125.33
122.56
119.79
110.79
41.12
40.77
40.42
40.07
34.78
29.97
28.34
4x
A (q)
123.94
B (q)
40.60
1.00
3.06
210 200 190 180 170 160 150 140 130 120 110 100 90 80 70 60 50 40 30 20 10 0 -10
f1 (ppm)

dtm4030f6-12.4.fid
User dtm2000

^{19}F NMR, 376 MHz, $CDCl_3$

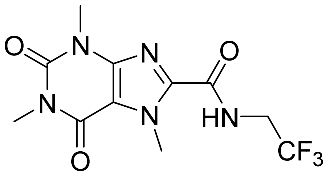

4x

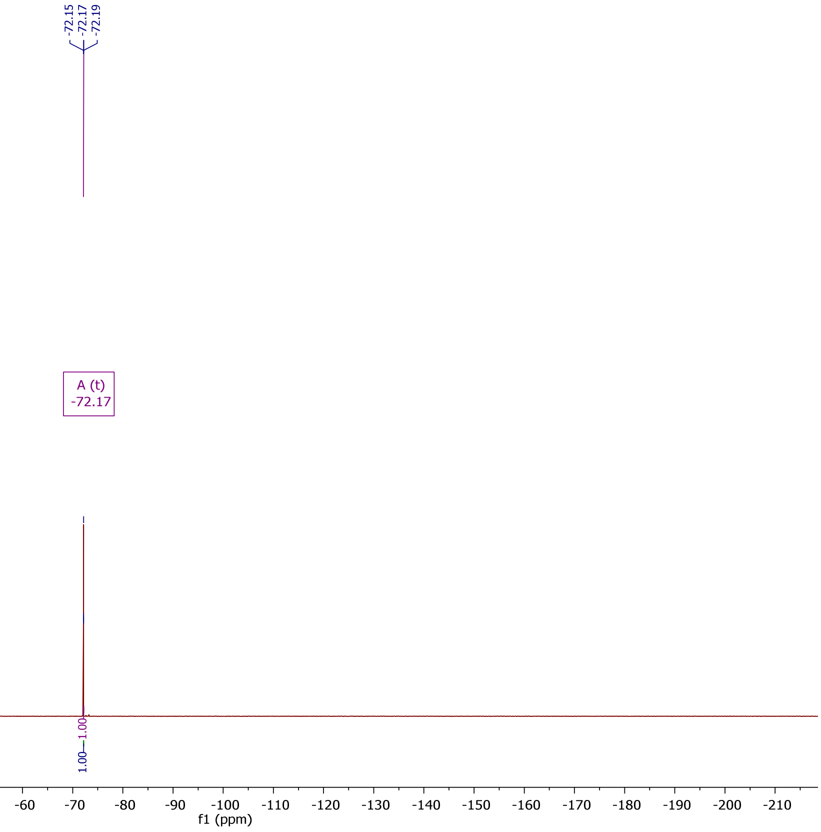

dtm3106a.1.fid

^{1}H NMR, 400 MHz, $CDCl_3$

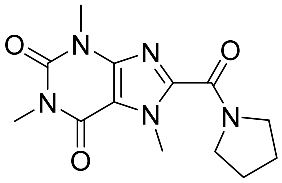

4y

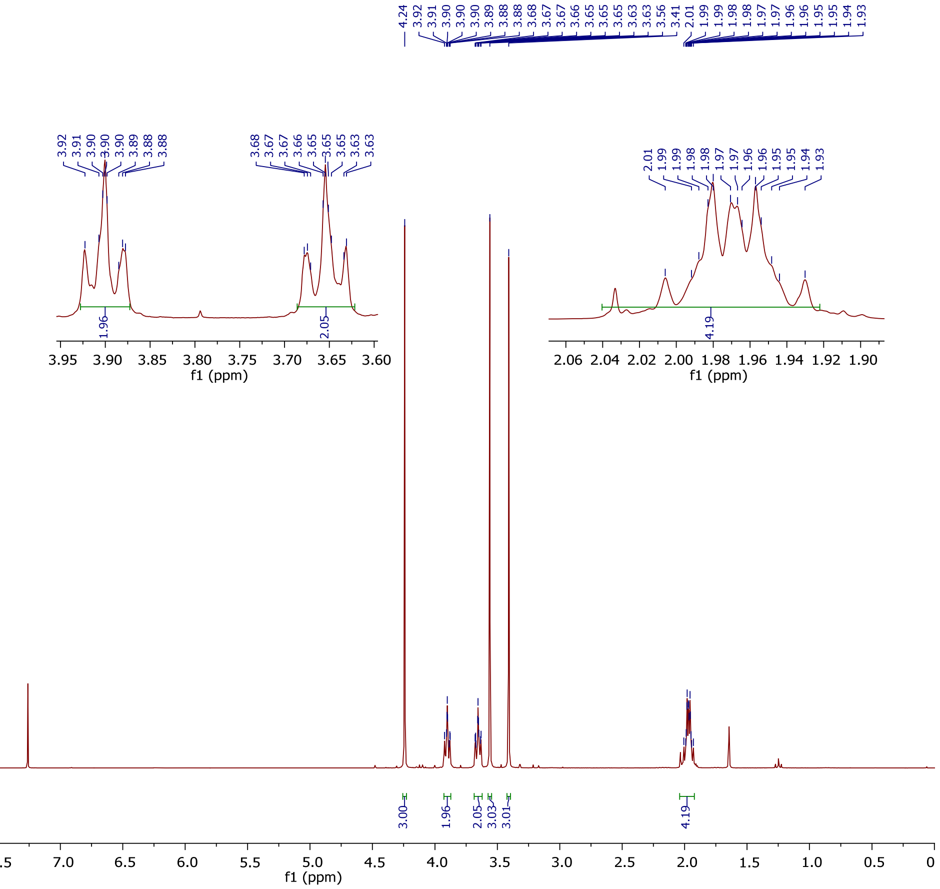

^{13}C NMR, 101 MHz, $CDCl_3$

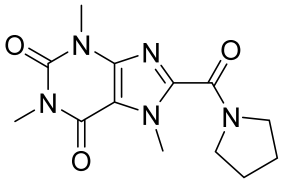

4y

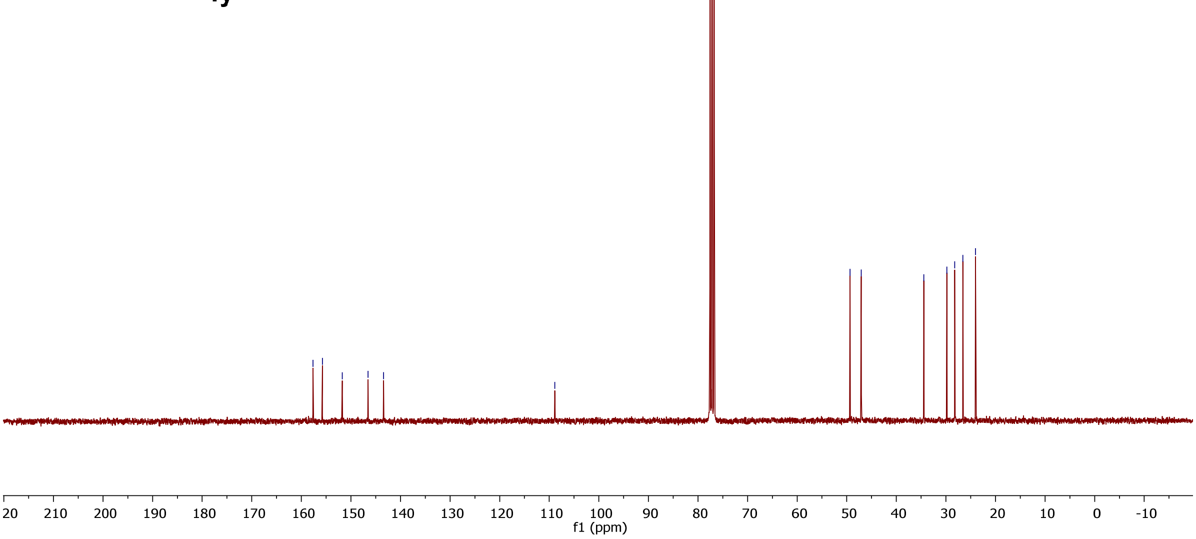

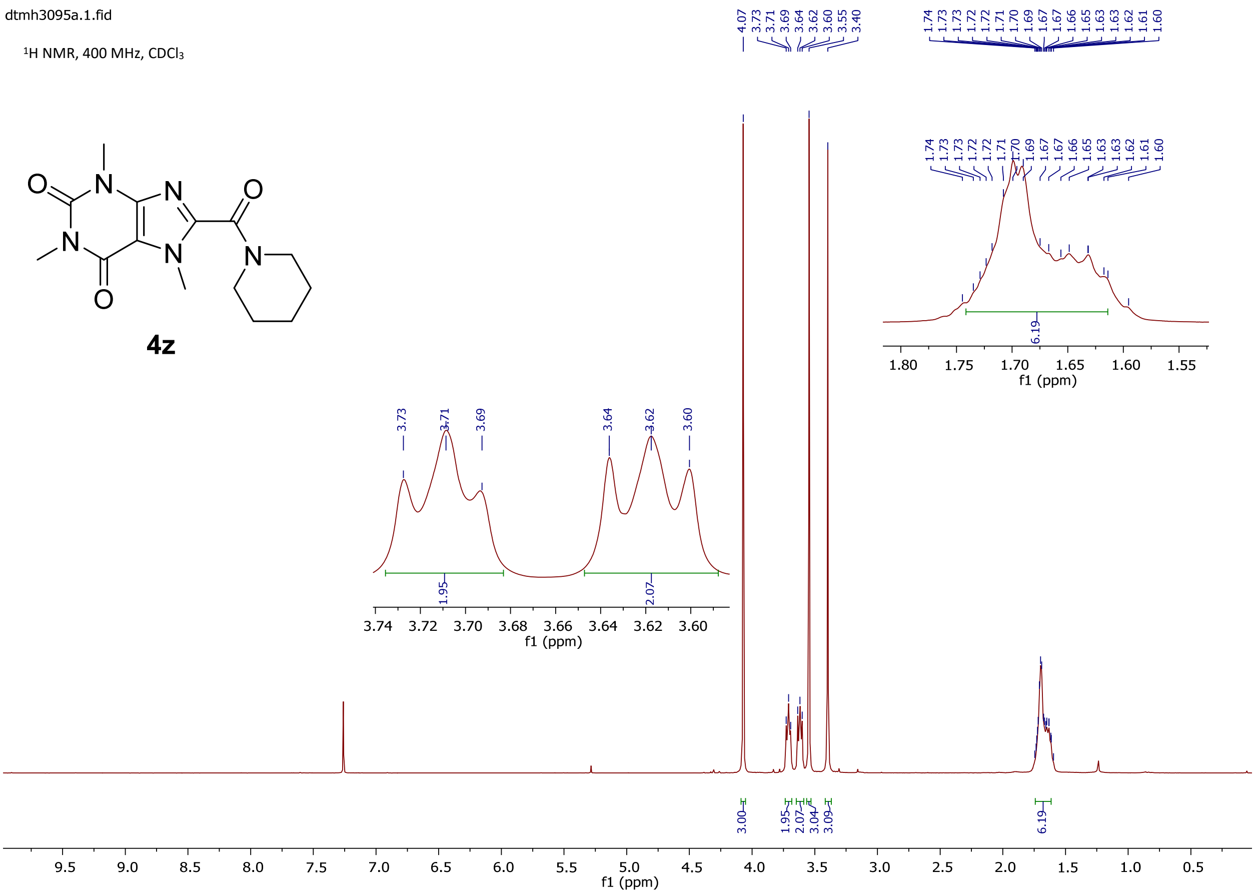
dtmh3095a.1.fid
1H NMR, 400 MHz, CDCl3
4z
4.07 3.73 3.71 3.69 3.64 3.62 3.60 3.55 3.40
1.74 1.73 1.73 1.72 1.72 1.71 1.70 1.69 1.67 1.67 1.66 1.65 1.63 1.63 1.62 1.61 1.60
3.00 1.95 2.07 3.04 3.09 6.19
f1 (ppm)

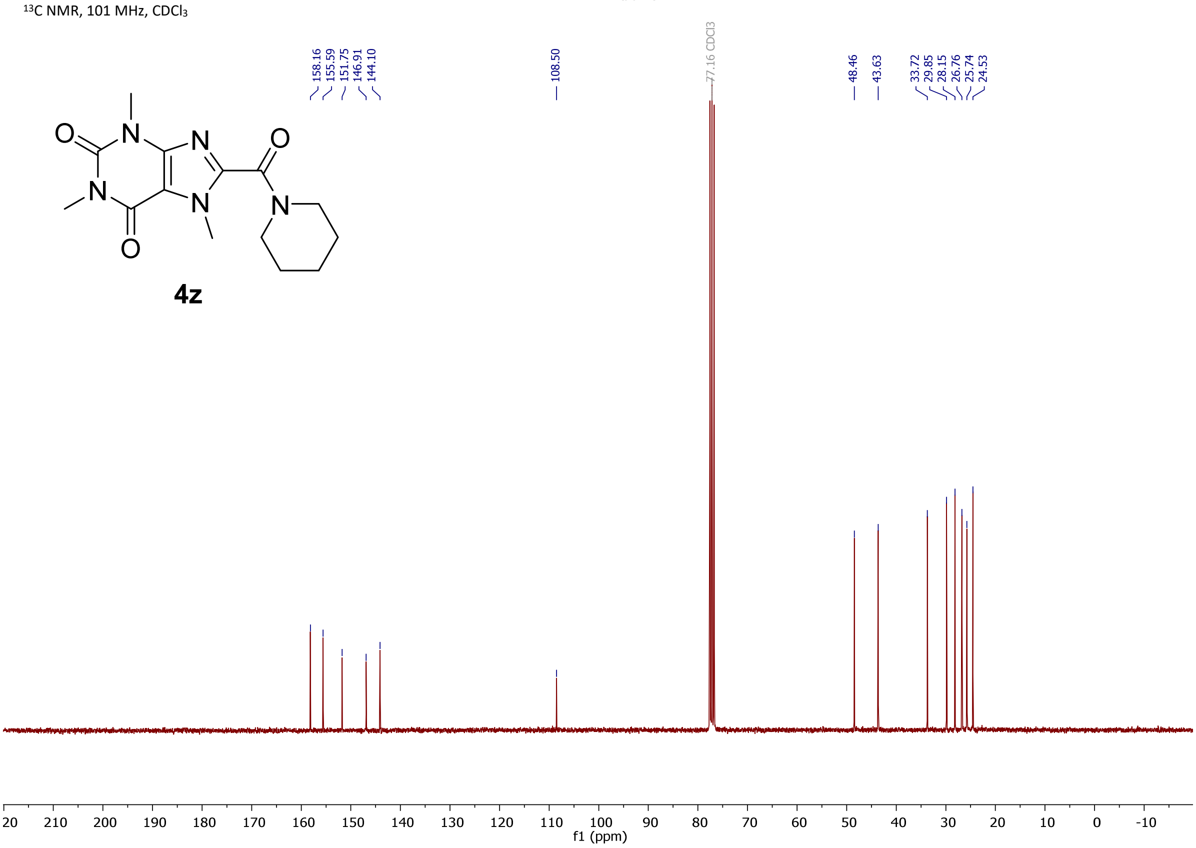
13C NMR, 101 MHz, CDCl3
4z
158.16 155.59 151.75 146.91 144.10
108.50
77.16 CDCl3
48.46 43.63
33.72 29.85 28.15 26.76 25.74 24.53
f1 (ppm)

dtmh3105f10-20.1.fid

^{1}H NMR, 400 MHz, $CDCl_3$

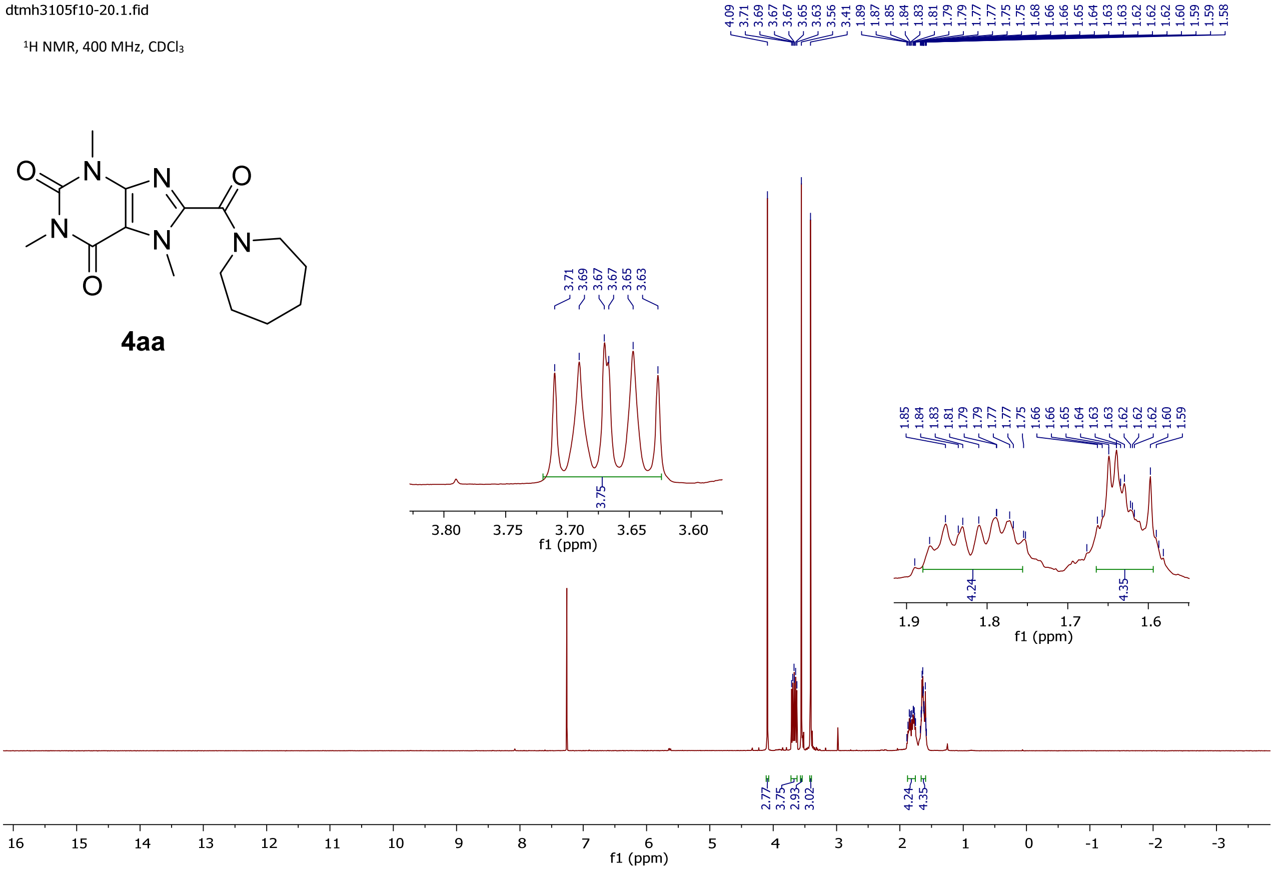

4aa

^{13}C NMR, 101 MHz, $CDCl_3$

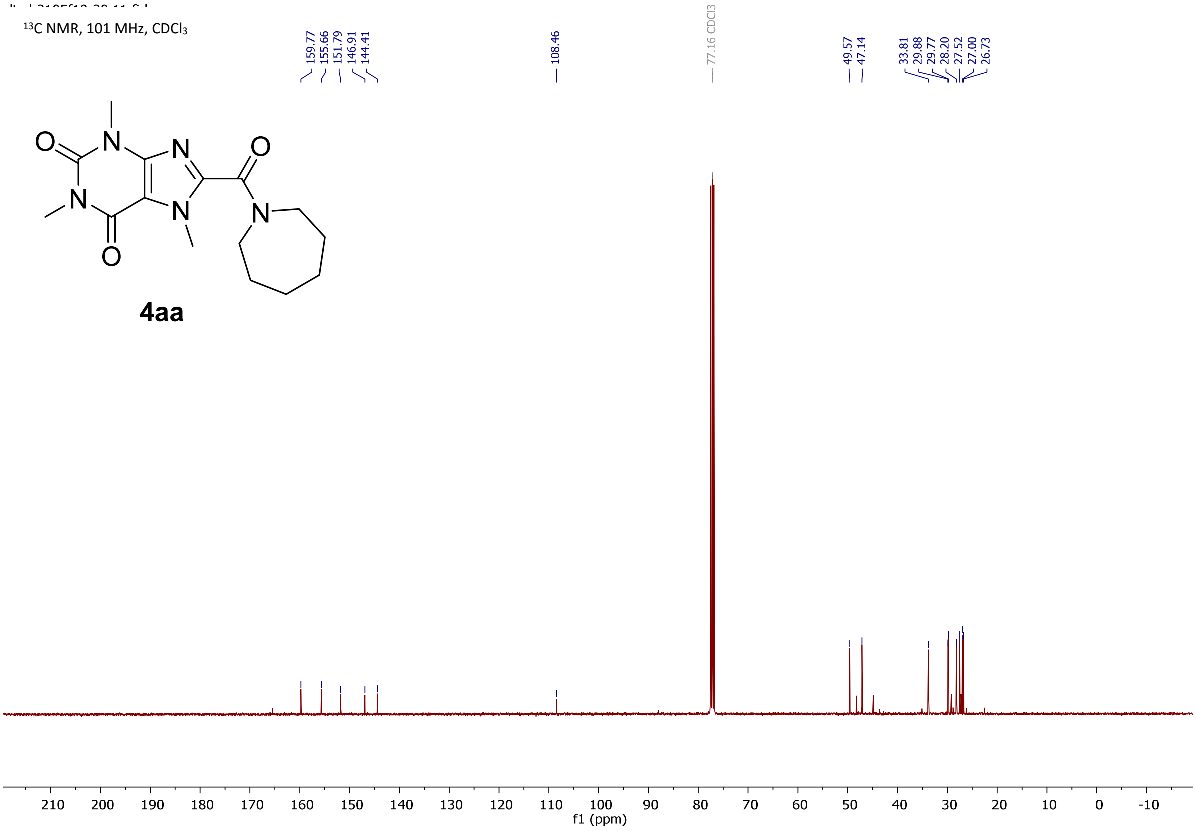

4aa

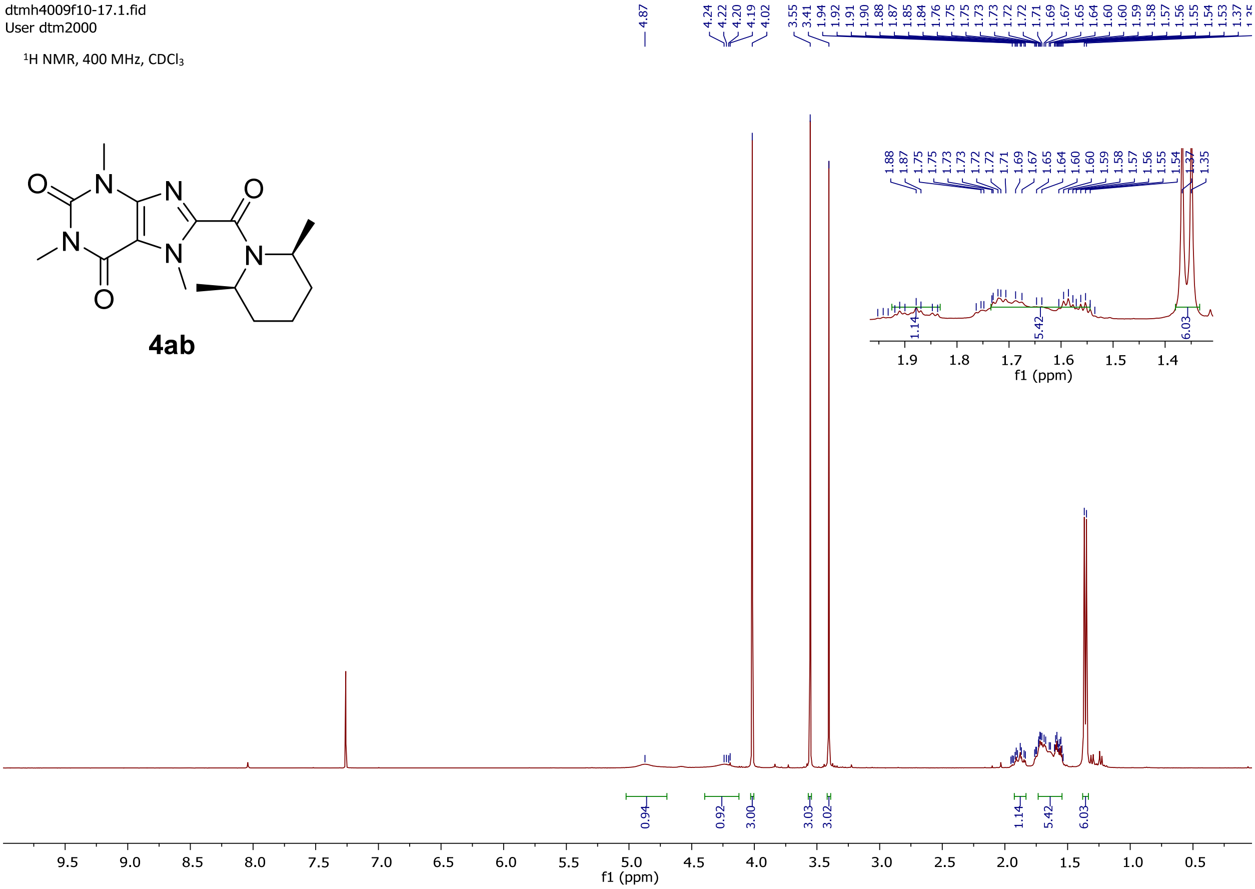
dtmh4009f10-17.1.fid
User dtm2000
¹H NMR, 400 MHz, CDCl₃
4ab
4.87
4.24 4.22 4.20 4.19 4.02
3.55 3.41 1.94 1.92 1.91 1.90 1.88 1.87 1.85 1.84 1.76 1.75 1.75 1.73 1.73 1.72 1.72 1.71 1.69 1.67 1.65 1.64 1.60 1.60 1.59 1.58 1.57 1.56 1.55 1.54 1.53 1.37
0.94 0.92 3.00 3.03 3.02 1.14 5.42 6.03
f1 (ppm)

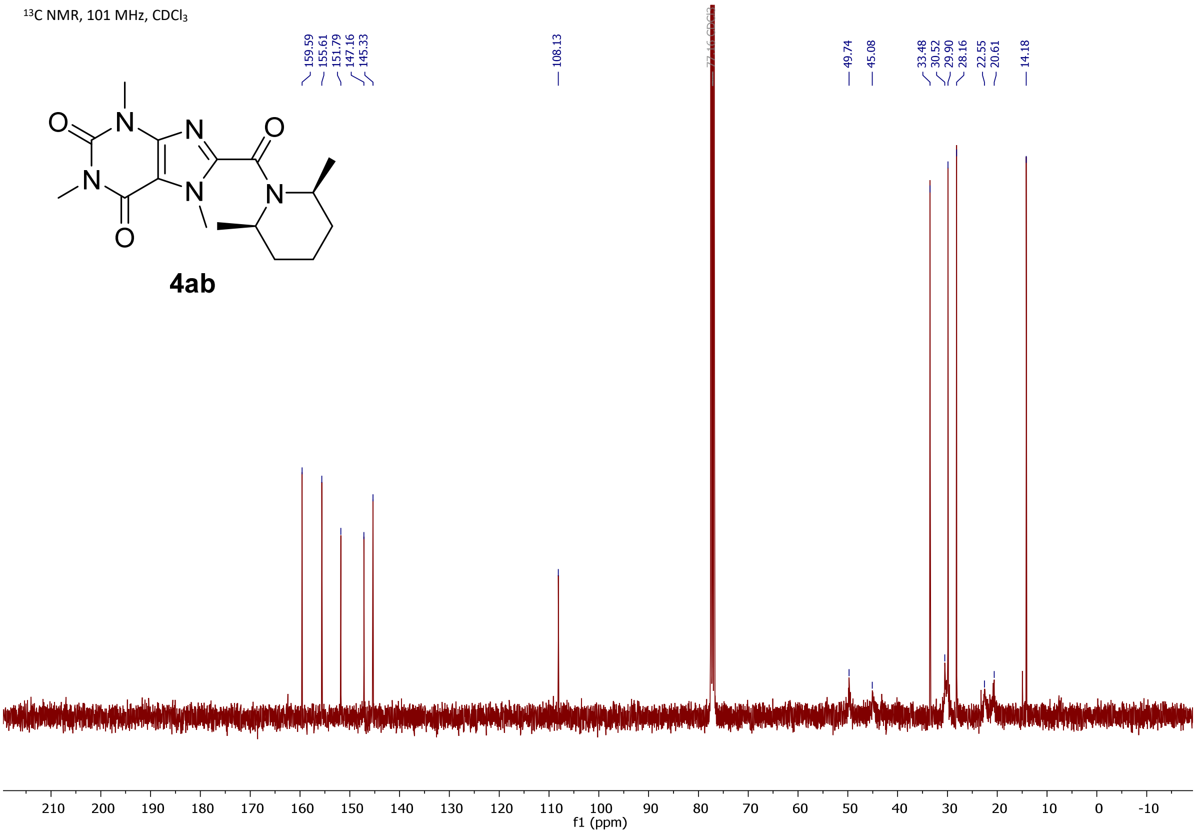
¹³C NMR, 101 MHz, CDCl₃
4ab
159.59 155.61 151.79 147.16 145.33
108.13
49.74 45.08
33.48 30.52 29.90 28.16 22.55 20.61 14.18
f1 (ppm)

DTM4044a.10.fid
User dtm

^{1}H NMR, 400 MHz, $CDCl_3$

4ac

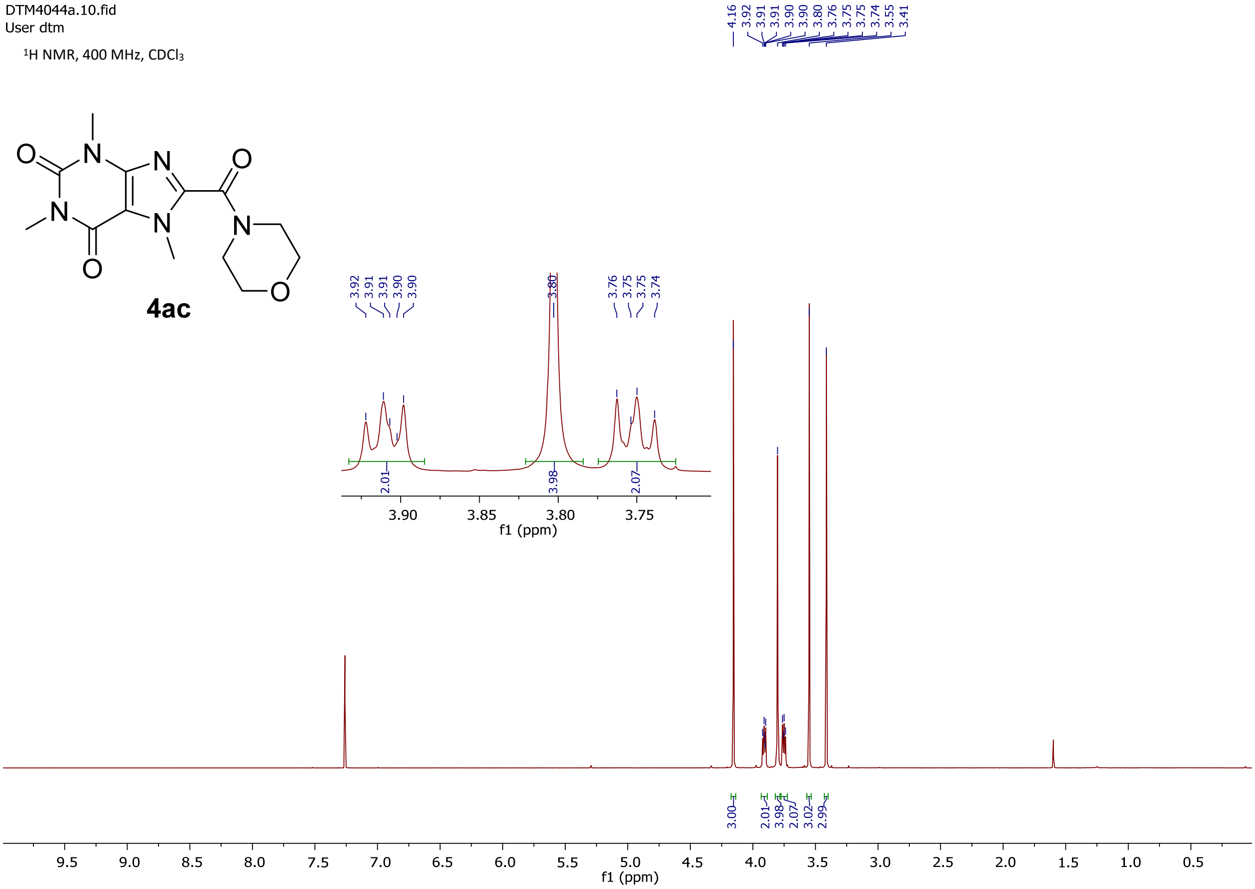

^{13}C NMR, 101 MHz, $CDCl_3$

4ac

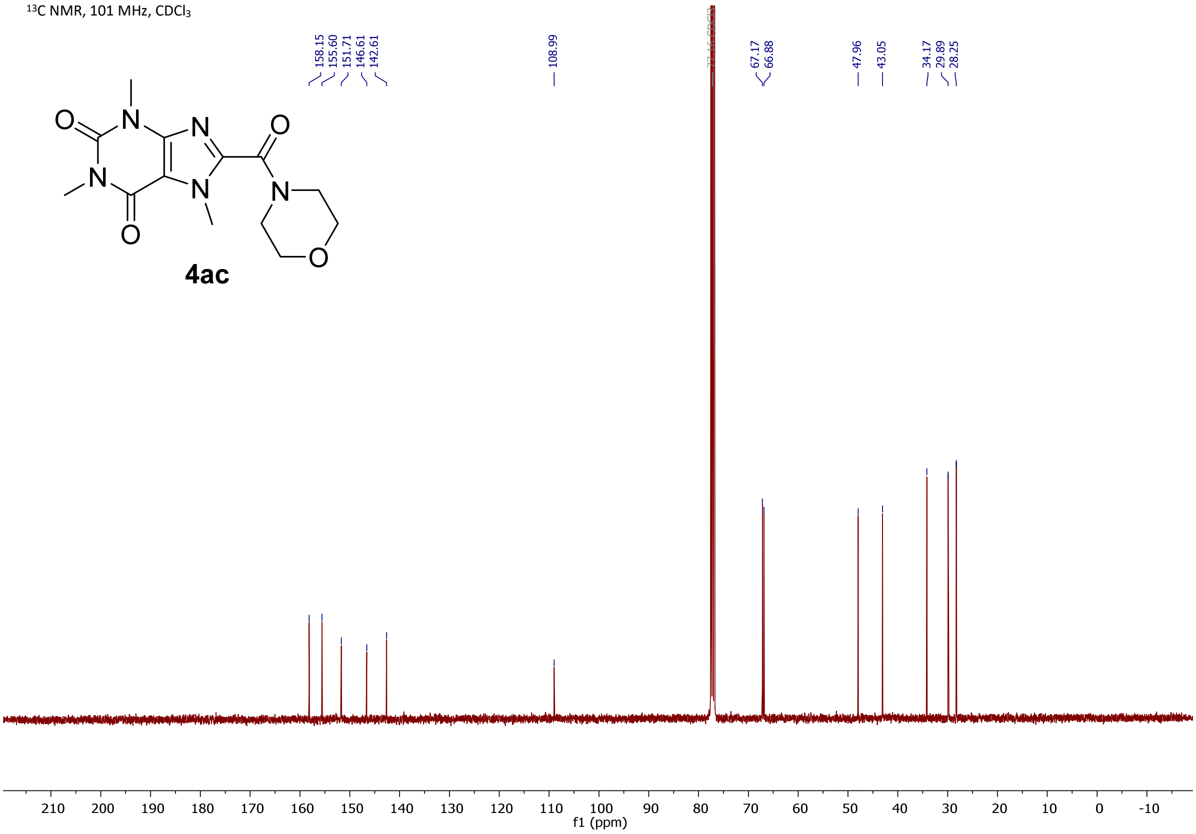

DTM4045a.10.fid
User dtm

1H NMR, 400 MHz, $CDCl_3$

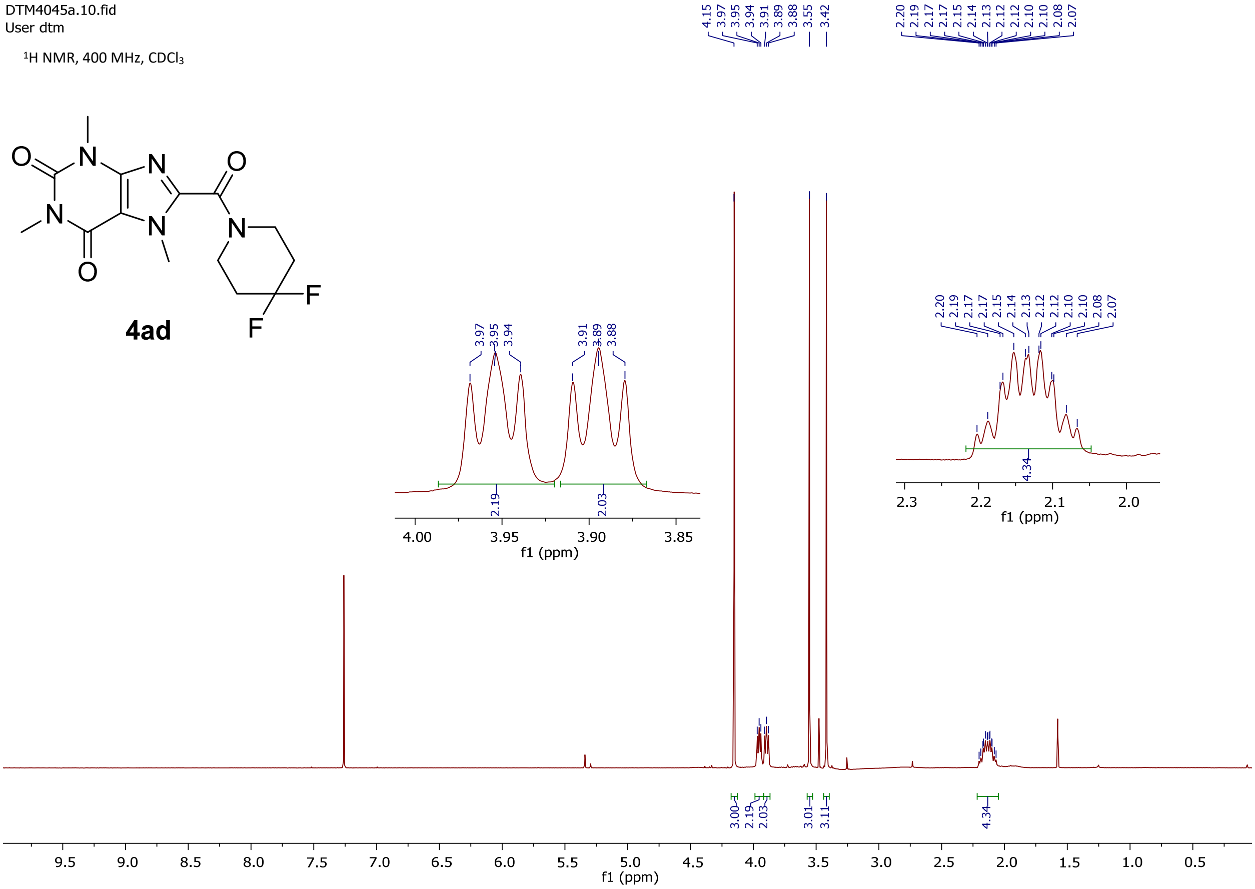

13C NMR, 101 MHz, $CDCl_3$

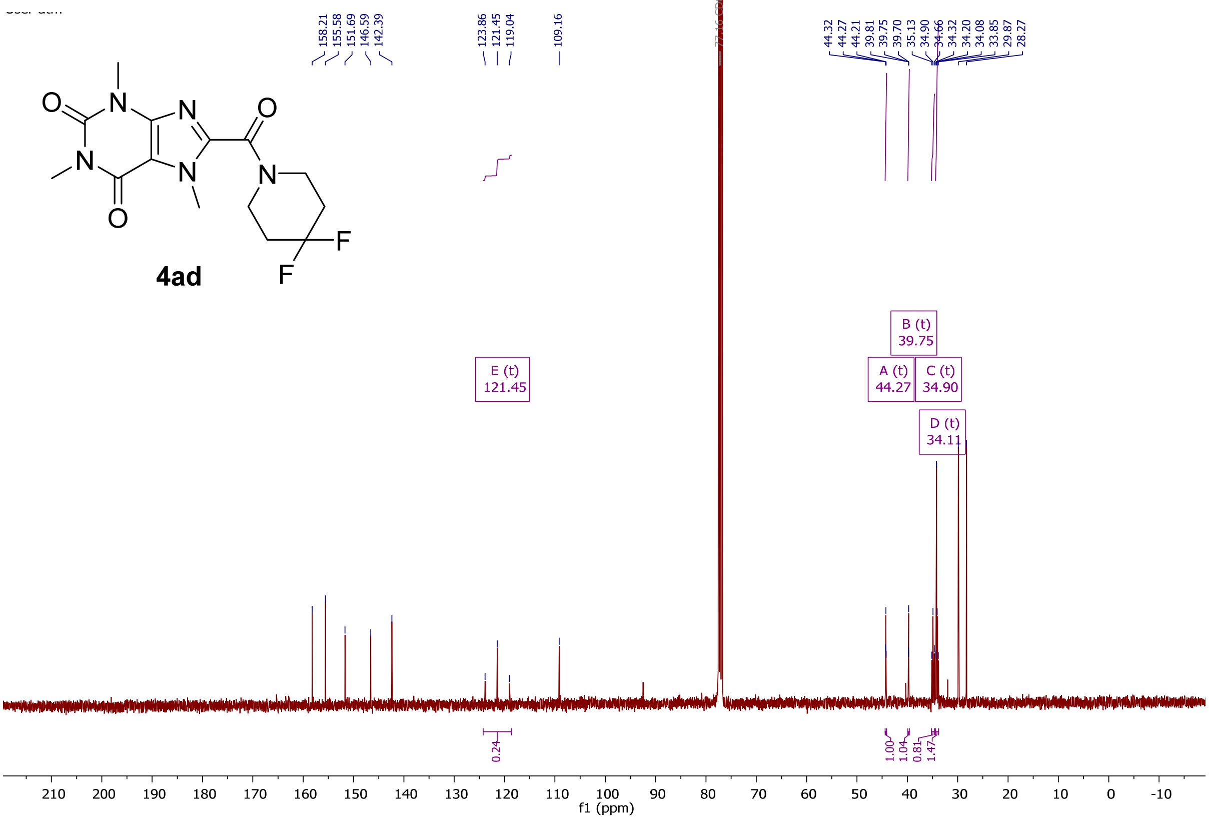

DTM4045a.11.fid
User dtm

^{19}F NMR, 376 MHz, $CDCl_3$

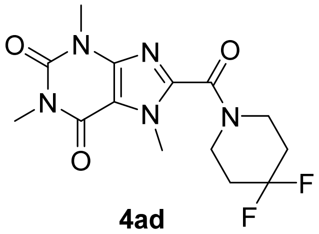

4ad

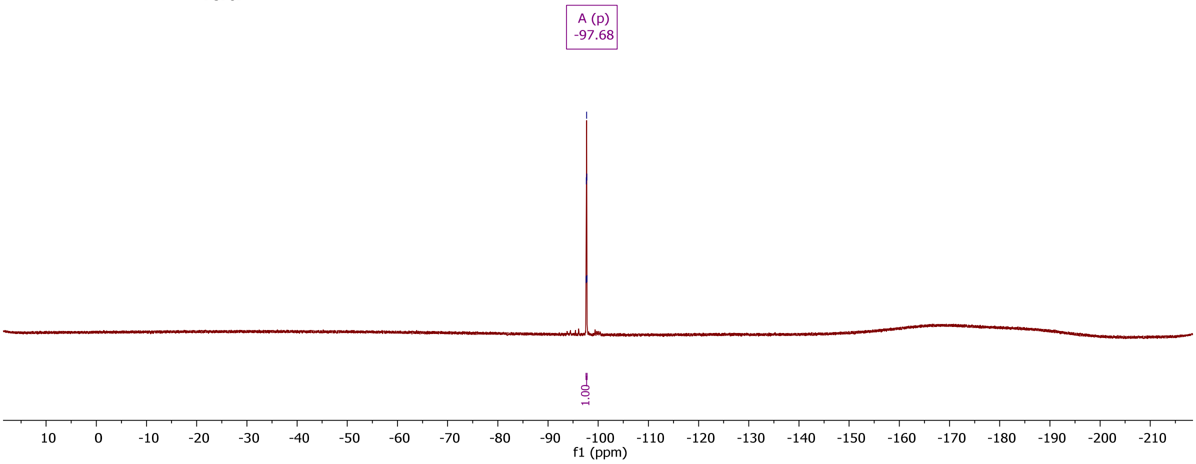

dtm4013a.6.fid

^{1}H NMR, 400 MHz, $CDCl_3$

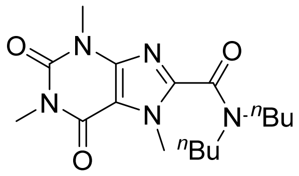

4ae

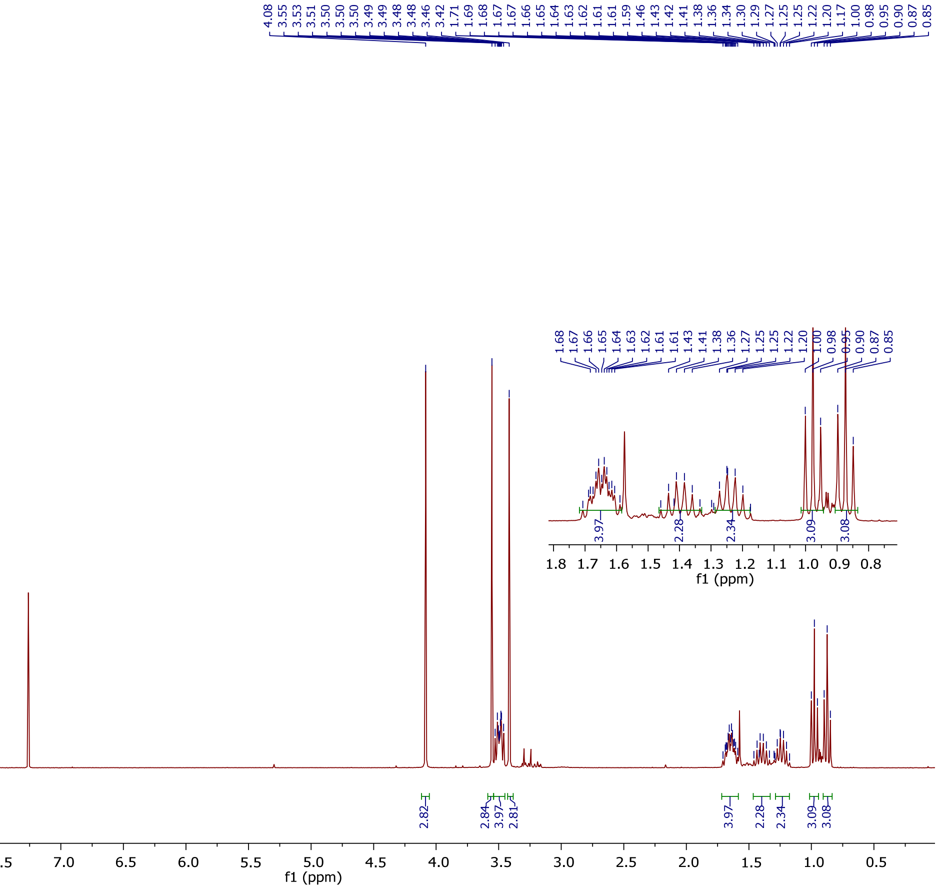

^{13}C NMR, 101 MHz, $CDCl_3$

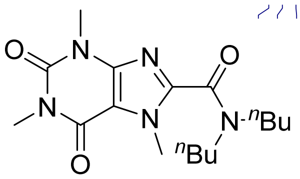

4ae

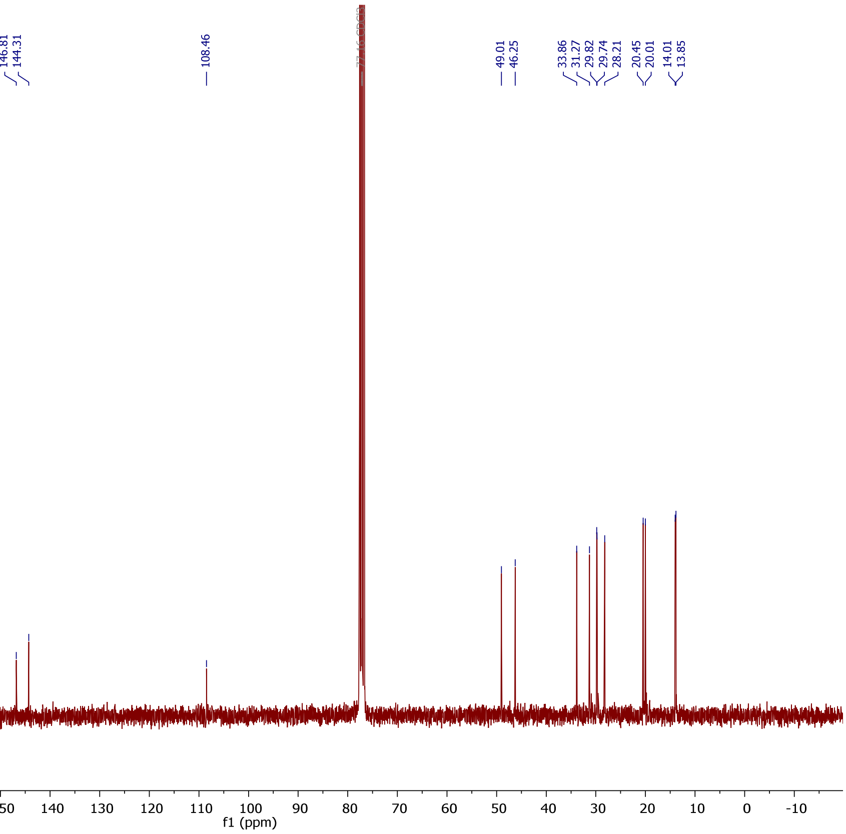

dtm4091a.10.fid
User dtm

^1H NMR, 400 MHz, $CDCl_3$

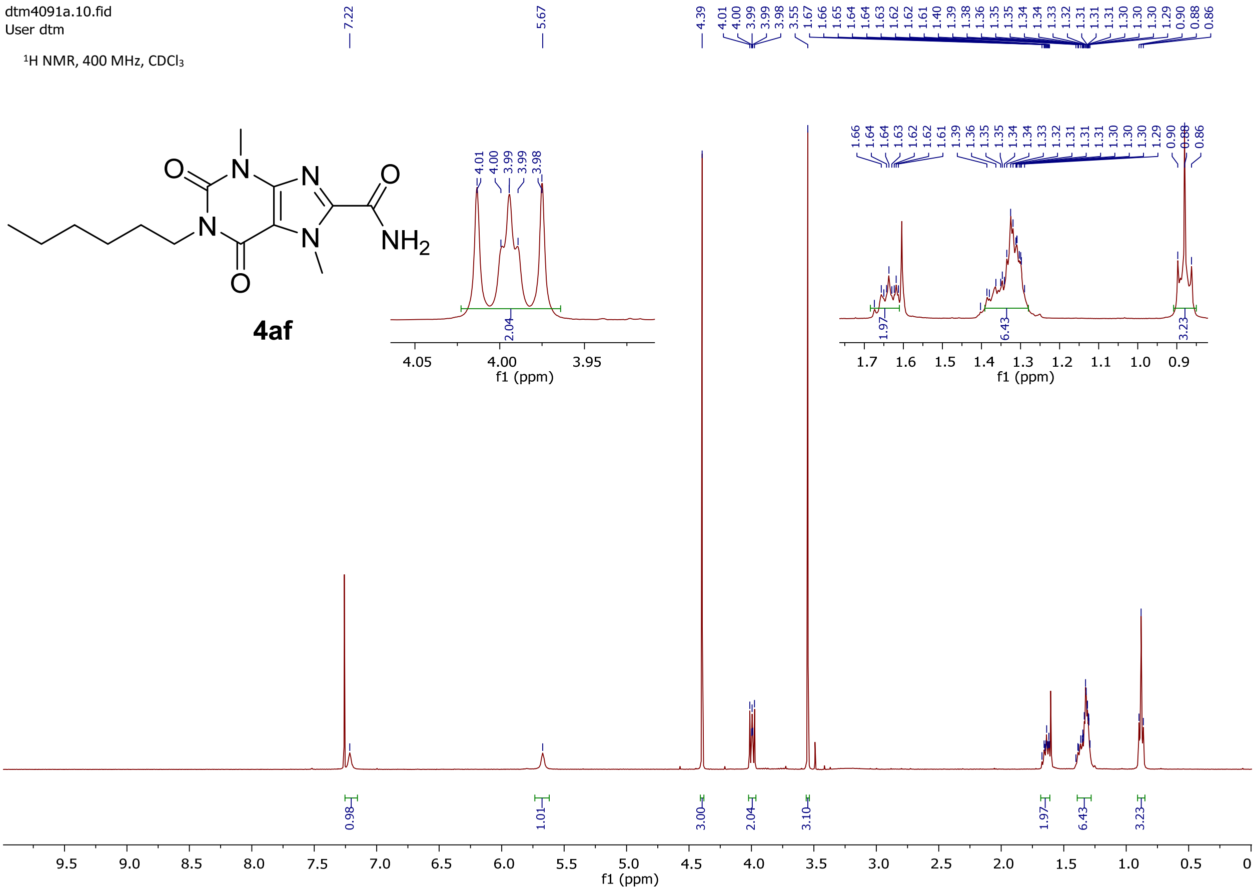

dtm4091a.11.fid
User dtm

^{13}C NMR, 101 MHz, $CDCl_3$

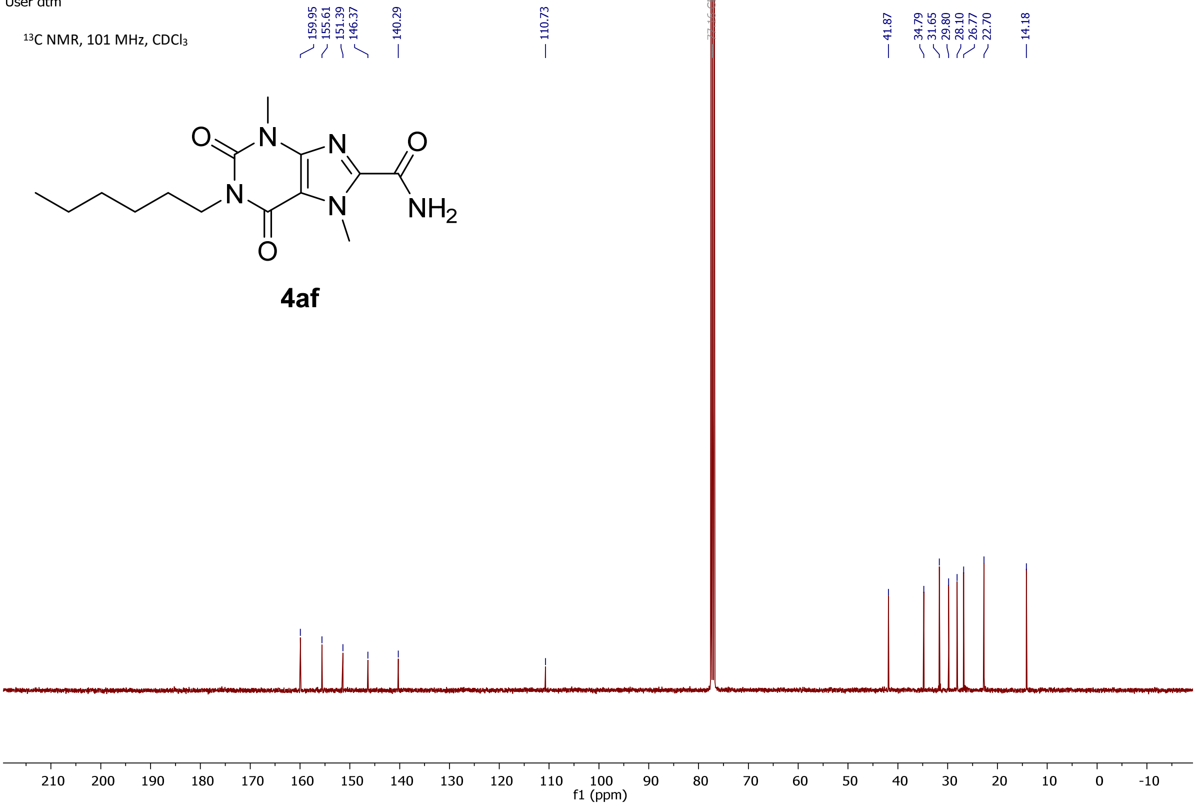

dtm4098a.10.fid
User dtm

^{1}H NMR, 400 MHz, $CDCl_3$

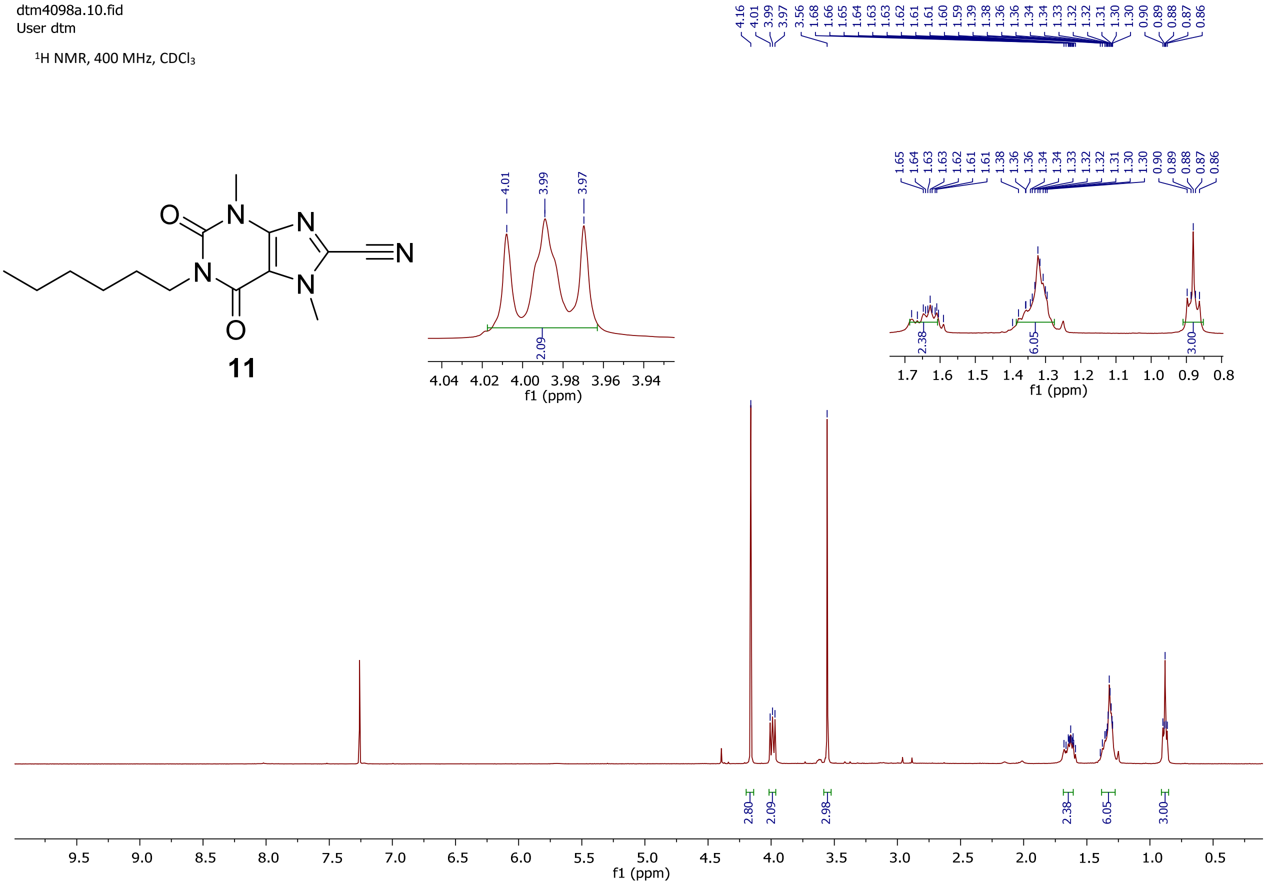

^{13}C NMR, 101 MHz, $CDCl_3$

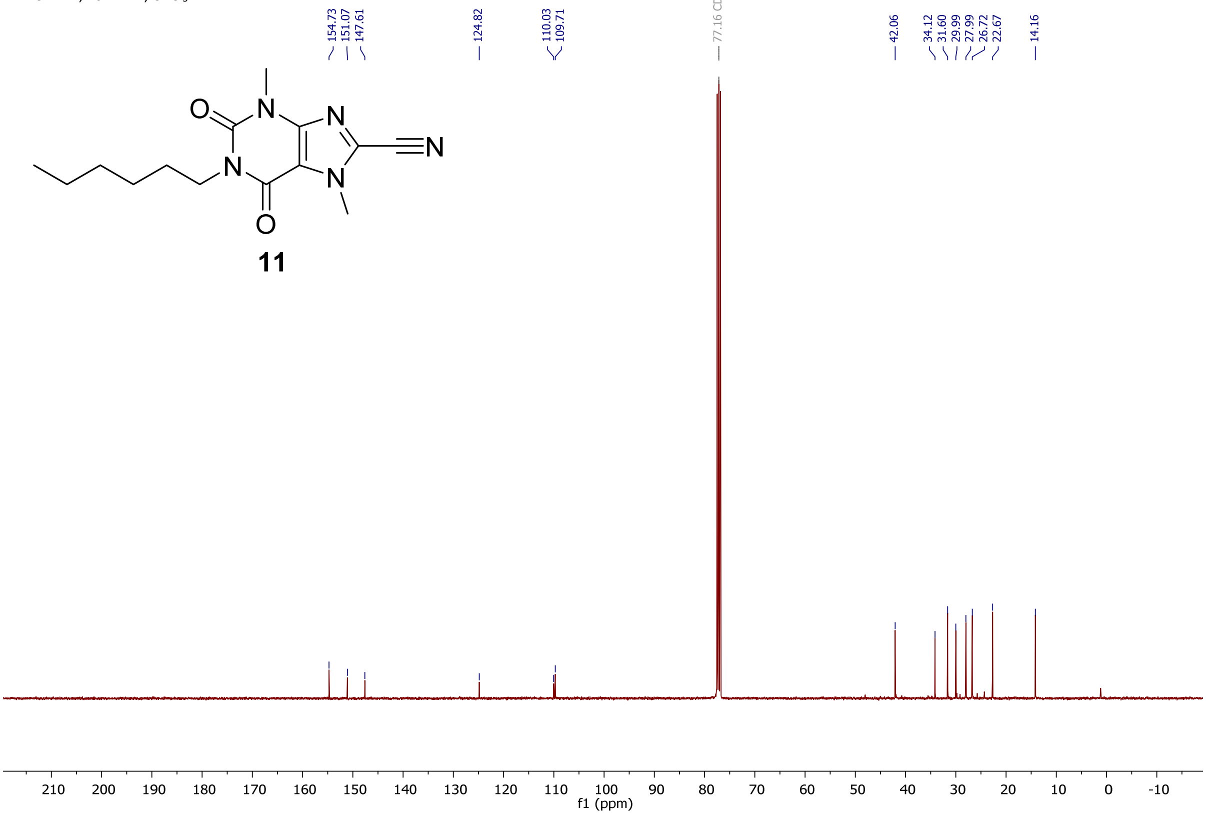

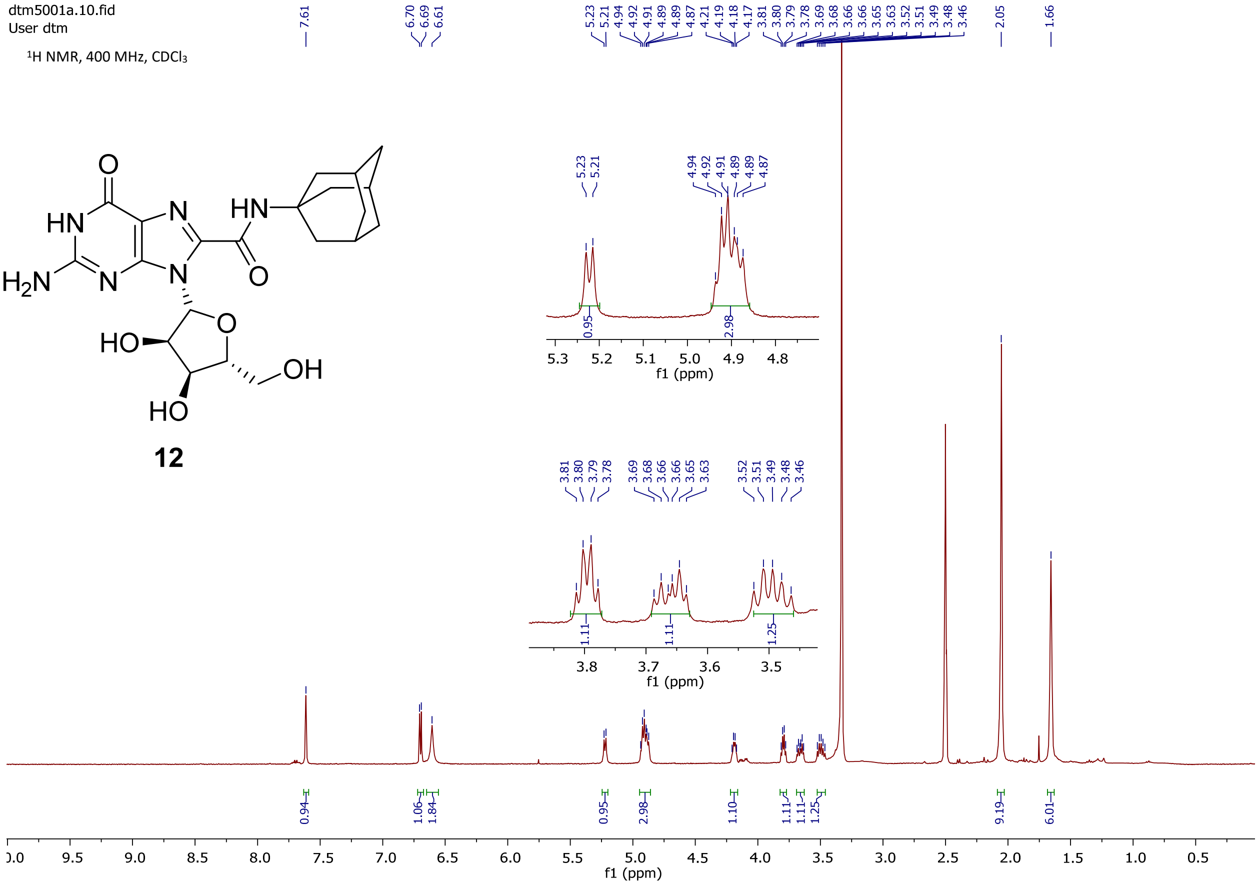
dtm5001a.10.fid
User dtm
^{1}H NMR, 400 MHz, $CDCl_3$
7.61
6.70
6.69
6.61
5.23
5.21
4.94
4.92
4.91
4.89
4.89
4.87
4.21
4.19
4.18
4.17
3.81
3.80
3.79
3.78
3.69
3.68
3.66
3.66
3.65
3.63
3.52
3.51
3.49
3.48
3.46
2.05
1.66
HN
H2N
N
O
HO
OH
12
0.94
1.06
1.84
0.95
2.98
1.10
1.11
1.11
1.25
9.19
6.01
f1 (ppm)

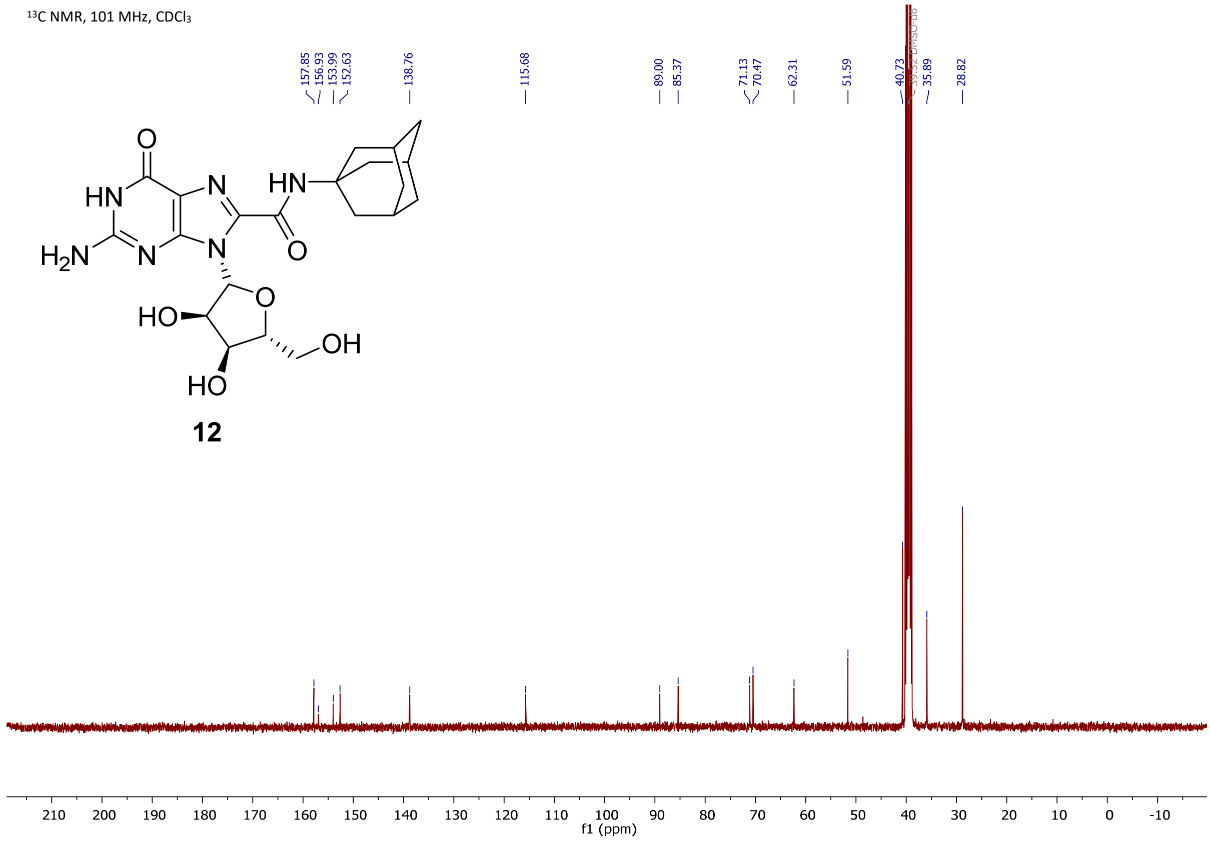
^{13}C NMR, 101 MHz, $CDCl_3$
157.85
156.93
153.99
152.63
138.76
115.68
89.00
85.37
71.13
70.47
62.31
51.59
40.73
39.52 DMSO-d6
35.89
28.82
12
f1 (ppm)

dtm5006a.10.fid
User dtm

^{1}H NMR, 400 MHz, $CDCl_3$

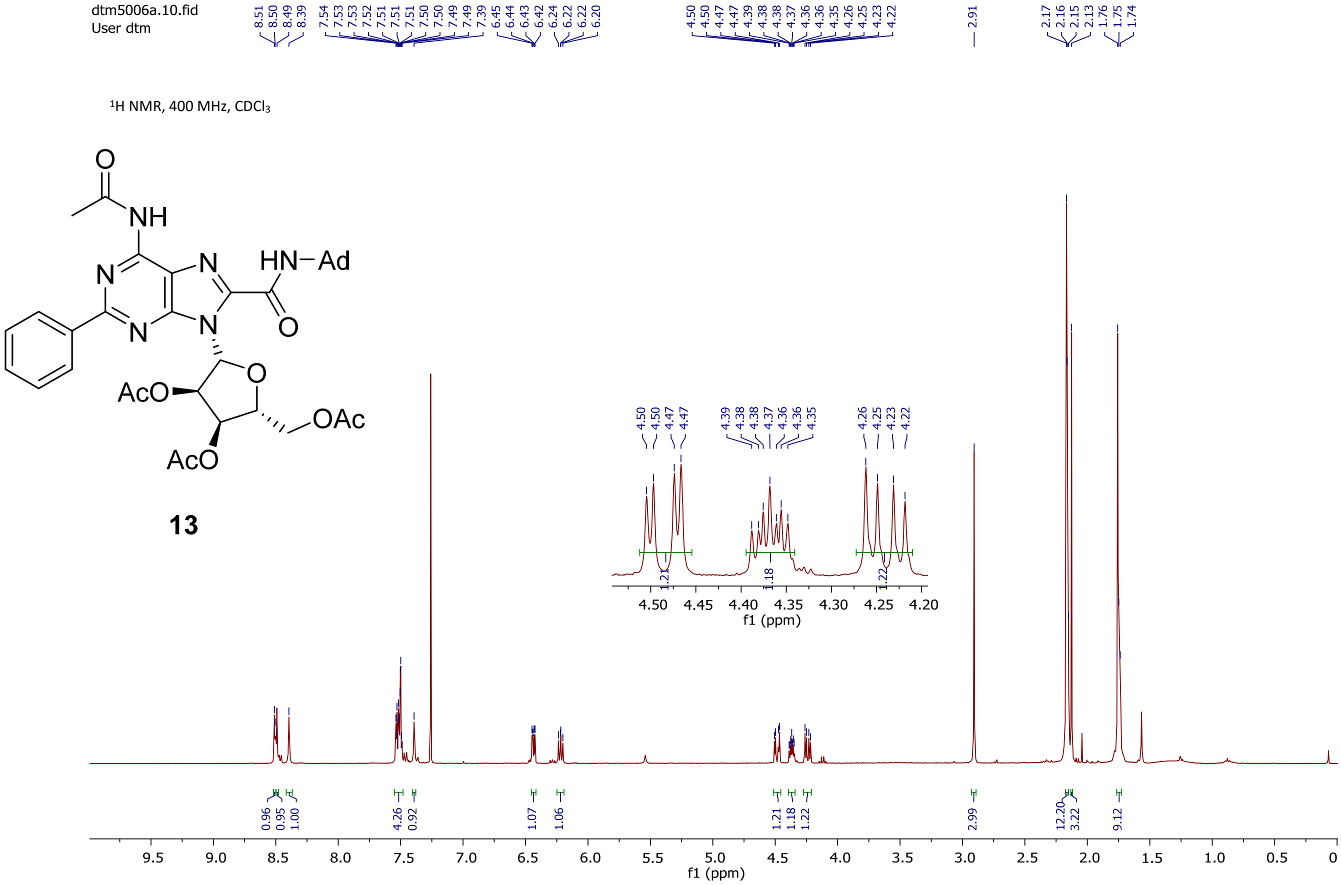

^{13}C NMR, 101 MHz, $CDCl_3$

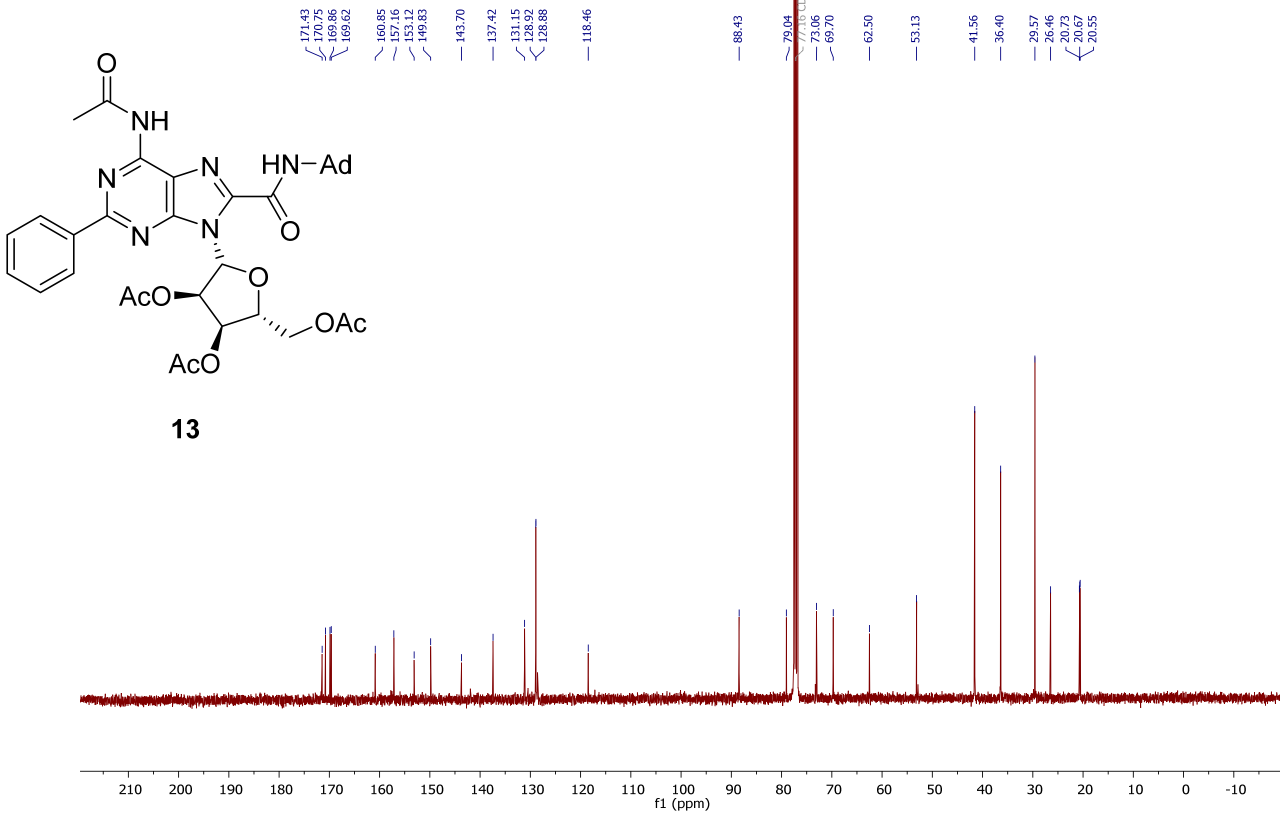

8. References

1. Mooney, D. T.; Donkin, B. D. T.; Demirel, N.; Moore, P. R.; Lee, A.-L., Direct C–H Functionalization of Phenanthrolines: Metal- and Light-Free Dicarbamoylations. *J. Org. Chem* **2021,** *86*, 17282-17293.
2. M. T. Westwood; C. J. C. Lamb; D. R. Sutherland; Lee, A., Metal-, Photocatalyst- and Light-Free Direct C-H Acylation and Carbamoylation of Heterocycles. *Org. Lett.* **2019,** *21*, 7119-7123.
3. Pawar, G. G.; Robert, F.; Grau, E.; Cramail, H.; Landais, Y., Visible-Light Photocatalyzed Oxidative Decarboxylation of Oxamic Acids: a Green Route to Urethanes and Ureas. *Chem. Commun.* **2018,** *54*, 9337-9340.
4. Zhao, M. M.; McNamara, J. M.; Ho, G.-J.; Emerson, K. M.; Song, Z. J.; Tschaen, D. M.; Brands, K. M. J.; Dolling, U.-H.; Grabowski, E. J. J.; Reider, P. J.; Cottrell, I. F.; Ashwood, M. S.; Bishop, B. C., Practical Asymmetric Synthesis of Aprepitant, a Potent Human NK-1 Receptor Antagonist, via a Stereoselective Lewis Acid-Catalyzed Trans Acetalization Reaction. *J. Org. Chem* **2002,** *67*, 6743-6747.
5. Girijavallabhan, V.; Arasappan, A.; Bennett, F.; Chen, K.; Dang, Q.; Huang, Y.; Kerekes, A.; Nair, L.; Pissarnitski, D.; Verma, V.; Alvarez, C.; Chen, P.; Cole, D.; Esposite, S.; Huang, Y.; Hong, Q.; Liu, Z.; Pan, W.; Pu, H.; Rossman, R.; Truong, Q.; Vibulbhan, B.; Wang, J.; Zhao, Z.; Olsen, D.; Stamford, A.; Bogen, S.; Njoroge, F. G., 2′-Modified Guanosine Analogs for the Treatment of HCV. *Nucleosides, Nucleotides & Nucleic Acids* **2016,** *35*, 277-294.
6. Tararov, V. I.; Kolyachkina, S. V.; Alexeev, C. S.; Mikhailov, S. N., N6-Acetyl-2′,3′,5′-tri-O-acetyladenosine; A Convenient, ‘Missed Out’ Substrate for Regioselective N6-Alkylations. *Synthesis* **2011,** *2011*, 2483-2489.
7. Kong, X.; Zuo, H.; Huang, H.-D.; Zhang, Q.; Chen, J.; He, C.; Hu, Y., STING as an Emerging Therapeutic Target for Drug Discovery: Perspectives from the Global Patent Landscape. *J. Adv. Res.* **2022**. DOI: https://doi.org/10.1016/j.jare.2022.05.006
